# A comprehensive analysis on the safety of two biologics dupilumab and omalizumab

**DOI:** 10.3389/fmed.2024.1435370

**Published:** 2024-08-08

**Authors:** Yu Xiao, Wanying Yang, Muyang Wang

**Affiliations:** Department of Dermatology, The People’s Hospital of Longhua, Shenzhen, Guangdong, China

**Keywords:** biologics, dupilumab, omalizumab, safety, serious adverse events, atopic dermatitis, chronic spontaneous urticaria

## Abstract

Dupilumab was approved for the treatment of several dermatologic immune-mediated inflammatory diseases, such as atopic dermatitis and bullous pemphigoid; whereas omalizumab is the first biological agent which was approved to treat chronic spontaneous urticaria. None of the published meta-analyses has provided the sufficient data regarding the safety of these two biologics, especially regarding their potential serious adverse events (SAEs). The aim of this study was, to comprehensively evaluate the safety of the two biologics dupilumab and omalizumab. In this study, we included 32 randomized trials, and performed meta-analyses on 113 types of SAEs regarding dupilumab and 61 types of SAEs regarding omalizumab. We identified that: (1) use of dupilumab was significantly associated with the lower incidence of atopic dermatitis, while use of omalizumab was significantly associated with the lower incidence of asthma; and (2) use of dupilumab was not significantly associated with the incidences of 112 other kinds of SAEs including various infectious diseases, while use of omalizumab was not significantly associated with the incidences of 60 other kinds of SAEs including various infectious diseases. This meta-analysis for the first time assessed the association between use of dupilumab or omalizumab and incidences of various SAEs, and identified that neither dupilumab use nor omalizumab use was associated with the increased risks of any SAEs including various infectious diseases. These findings further confirm the general safety of the two biologics dupilumab and omalizumab. This informs clinicians that there is no need to worry too much about the safety issues of these two biologics.

## Introduction

Dupilumab is a newly developed monoclonal antibody and is able to block signaling of IL-4 and IL-13, both of which are crucial cytokines in the T2 response (1). This biologic agent has been approved for the treatment of atopic dermatitis, prurigo nodularis and bullous pemphigoid (2). Meanwhile, dupilumab has been demonstrated to be effective for the treatment of concomitant atopic dermatitis and chronic rhinosinusitis with nasal polyposis (3). Moreover, the new possible indications of dupilumab are increasingly explored, and those include but are not limited to various skin diseases, such as nummular eczema, chronic hand eczema, allergic contact dermatitis, and alopecia areata (4). On the other hand, omalizumab is a recombinant humanized monoclonal antibody that selectively binds to free IgE and inhibits the binding of IgE to FcεRI on mast cells and basophils. This effect subsequently downregulates the expression of FcεRI on mast cells and basophils (5). Clinical studies show that omalizumab is an effective and well-tolerated treatment for patients with chronic spontaneous urticaria (6, 7). Therefore, omalizumab become the first biological agent which was approved to treat chronic spontaneous urticaria (8, 9).

Several recent meta-analyses (10–13) have confirmed the intermediate effectiveness of dupilumab in treating atopic dermatitis, whereas a recent meta-analysis (14) have revealed the limited effectiveness of dupilumab in treating alopecia areata (in other words, the efficacy of dupilumab in alopecia areata is debated). Moreover, two recent meta-analyses (15, 16) have suggested the obvious effectiveness of omalizumab for the treatment of chronic spontaneous urticaria, whereas a recent meta-analysis (17) have shown the nonsignificant efficacy of omalizumab for the treatment of pemphigoid. The aforementioned meta-analyses have mainly focused on assessing the efficacy of dupilumab and omalizumab on several dermatologic immune-mediated inflammatory diseases, but have provided no data (12–16) or the limited data (10, 11, 17) regarding the safety of these two biologics, especially regarding serious adverse events (SAEs) possibly associated with dupilumab and omalizumab. Therefore, we carried out a meta-analysis, with the purpose of comprehensively characterizing the safety of the two biologics dupilumab and omalizumab. To achieve this purpose, we evaluated the association between use of dupilumab or omalizumab and incidences of various SAEs including but not limited to various infectious diseases.

## Methods

Relevant studies published before May 16, 2024 were searched via PubMed and ClinicalTrials.gov. Studies eligible to be included in this meta-analysis were those randomized controlled trials (RCTs) that enrolled ≥300 participants, compared any of the two biologics (dupilumab and omalizumab) with an active drug, a placebo, or no drug, and reported various SAEs. In this meta-analysis, we included all the RCTs as long as they met the aforementioned criteria, whether the RCTs assessed dermatological diseases or other diseases. We extracted the data of various safety outcomes (i.e., various SAEs) from ClinicalTrials.gov. When extracting data, we gave priority to the data of which the control group was placebo. It meant that: when a study reported both the data of which the control group was a placebo and the data of which the control group was a non-placebo comparator, we only extracted the former data but not the later data. The data of which the study arm was switching from biologics of interest to non-biologics (or from non-biologics to biologics of interest) were excluded. We conducted meta-analyses on various safety outcomes, as long as they were reported by two or more of the included trials. We did meta-analysis based on the trials of dupilumab and omalizumab, respectively, using a fixed-effects model (when I^2^ was less than 50%) or a random-effects model (when I^2^ was great than or equal to 50%). Meta-analysis results were reported by the pooled risk ratios (RRs) and 95% confidence intervals (CIs).

## Results

In this meta-analysis we included a total of 32 RCTs, which consisted of 16 dupilumab RCTs and 16 omalizumab RCTs. The ClinicalTrials.gov registration numbers of included dupilumab trials were NCT01854047, NCT01859988, NCT02260986, NCT02277743, NCT02277769, NCT02395133, NCT02414854, NCT02755649, NCT02898454, NCT02948959, NCT03345914, NCT03633617, NCT03720470, NCT03738397, NCT03930732, and NCT04345367. These 16 trials of dupilumab involved 10616 participants including 6512 dupilumab users and 4104 non-dupilumab users. The ClinicalTrials.gov registration numbers of included omalizumab trials were NCT00079937, NCT00096954, NCT00264849, NCT00314574, NCT00377572, NCT01202903, NCT01264939, NCT01287117, NCT01292473, NCT01430403, NCT01716754, NCT02477332, NCT03328897, NCT03369704, NCT03580356, and NCT03580369. These 16 trials of omalizumab involved 7656 participants including 4141 omalizumab users and 3515 non-omalizumab users.

### Meta-analyses of dupilumab

The forest plots of meta-analyses of dupilumab and 113 safety outcomes are given in Supplementary Figure 1, and the summary results are presented in Supplementary Table 1. Dupilumab use was significantly associated with the lower incidence of one safety outcome, namely, Dermatitis atopic (RR 0.44, 95% CI 0.20–0.99; *P* = 0.047; I^2^ = 0.0%). Moreover, dupilumab use was not significantly associated with the incidences of the other 112 kinds of safety outcomes, including but not limited to various Infections And Infestations, i.e., Pneumonia (RR 0.62, 95% CI 0.31–1.24; *P* = 0.180; I^2^ = 0.0%), Appendicitis (RR 0.79, 95% CI 0.20–3.14; *P* = 0.735; I^2^ = 0.0%), Urinary tract infection (RR 0.94, 95% CI 0.20–4.38; *P* = 0.941; I^2^ = 0.0%), Sepsis (RR 0.17, 95% CI 0.03–1.07; *P* = 0.059; I^2^ = 0.0%), Gastroenteritis (RR 0.49, 95% CI 0.09–2.77; *P* = 0.422; I^2^ = 0.0%), Erysipelas (RR 1.53, 95% CI 0.24–9.70; P = 0.651; I^2^ = 0.0%), Diverticulitis (RR 0.58, 95% CI 0.09–3.70; *P* = 0.568; I^2^ = 0.0%), Cellulitis (RR 1.39, 95% CI 0.22–8.78; *P* = 0.727; I^2^ = 0.0%), Bronchitis (RR 0.62, 95% CI 0.10–3.82; *P* = 0.609; I^2^ = 0.0%), Upper respiratory tract infection (RR 0.71, 95% CI 0.07–6.82; *P* = 0.767; I^2^ = 36.1%), Staphylococcal infection (RR 0.24, 95% CI 0.02–2.29; *P* = 0.215; I^2^ = 0.0%), Septic shock (RR 0.71, 95% CI 0.07–6.84; *P* = 0.769; I^2^ = 0.0%), Pyelonephritis (RR 1.50, 95% CI 0.16–14.37; *P* = 0.726; I^2^ = 0.0%), Pneumonia bacterial (RR 0.51, 95% CI 0.05–4.89; *P* = 0.560; I^2^ = 0.0%), Influenza (RR 0.34, 95% CI 0.04–3.25; *P* = 0.349; I^2^ = 0.0%), Herpes zoster (RR 0.51, 95% CI 0.02–16.49; *P* = 0.704; I^2^ = 57.7%), Epiglottitis (RR 1.53, 95% CI 0.16–14.67; *P* = 0.712; I^2^ = 0.0%), COVID-19 pneumonia (RR 0.39, 95% CI 0.09–1.65; P = 0.199; I^2^ = 0.0%), COVID-19 (RR 0.65, 95% CI 0.17–2.51; *P* = 0.531; I^2^ = 0.0%), Chronic sinusitis (RR 0.50, 95% CI 0.05–4.83; *P* = 0.552; I^2^ = 0.0%), and Abdominal wall abscess (RR 0.86, 95% CI 0.09–8.27; *P* = 0.898; I^2^ = 14.7%).

### Meta-analyses of omalizumab

The forest plots of meta-analyses of omalizumab and 61 safety outcomes are given in Supplementary Figure 2, and the summary results are presented in Supplementary Table 2. Omalizumab use was significantly associated with the lower incidence of one safety outcome, namely, Asthma (RR 0.62, 95% CI 0.45–0.86; *P* = 0.004; I^2^ = 4.8%). Moreover, omalizumab use was not significantly associated with the incidences of the other 60 kinds of safety outcomes, including but not limited to various Infections And Infestations, i.e., Pneumonia (RR 0.45, 95% CI 0.19–1.08; *P* = 0.074; I^2^ = 0.0%), Appendicitis (RR 1.58, 95% CI 0.43–5.82; *P* = 0.493; I^2^ = 0.0%), Urinary tract infection (RR 0.92, 95% CI 0.19–4.39; P = 0.914; I^2^ = 0.0%), Upper respiratory tract infection (RR 0.73, 95% CI 0.15–3.59; *P* = 0.696; I^2^ = 0.0%), Gastroenteritis (RR 1.34, 95% CI 0.27–6.62; P = 0.720; I^2^ = 0.0%), Lower respiratory tract infection (RR 0.43, 95% CI 0.07–2.47; *P* = 0.343; I^2^ = 0.0%), Influenza (RR 0.78, 95% CI 0.13–4.75; *P* = 0.785; I^2^ = 0.0%), Gastroenteritis viral (RR 0.72, 95% CI 0.12–4.12; *P* = 0.708; I^2^ = 0.0%), Bronchitis (RR 0.42, 95% CI 0.08–2.06; *P* = 0.283; I^2^ = 0.0%), Sinusitis (RR 0.70, 95% CI 0.07–6.66; *P* = 0.752; I^2^ = 36.1%), Respiratory tract infection viral (RR 3.14, 95% CI 0.33–30.11; *P* = 0.321; I^2^ = 0.0%), Diverticulitis (RR 1.05, 95% CI 0.13–8.52; *P* = 0.962; I^2^ = 0.0%), and Cellulitis (RR 0.53, 95% CI 0.06–5.05; *P* = 0.579; I^2^ = 0.0%).

## Discussion

This is the first meta-analysis that comprehensively assessed the association between use of dupilumab or omalizumab and incidences of various SAEs including various infectious diseases. By performing meta-analyses on 113 safety outcomes regarding dupilumab and 61 safety outcomes regarding omalizumab, we produced two key findings as follows. First, use of dupilumab was significantly associated with the lower incidence of atopic dermatitis, while use of omalizumab was significantly associated with the lower incidence of asthma. Second, use of dupilumab was not significantly associated with the incidences of 112 other kinds of SAEs including various infectious diseases, while use of omalizumab was not significantly associated with the incidences of 60 other kinds of SAEs including various infectious diseases.

Four meta-analyses (10–13) identified that dupilumab was effective in treating atopic dermatitis, while two meta-analyses (18, 19) showed that omalizumab was effective in treating asthma. These findings are consistent with the first finding in our meta-analysis. On the other hand, use of dupilumab was not associated with the increased risk of skin infections in Marko et al.’s meta-analysis (20), and dupilumab could reduce new/worsened allergy events versus placebo in Geba et al.’s meta-analysis (21). Moreover, Lin et al.’s meta-analysis (22) demonstrated that omalizumab led to the lower incidences of safety concerns as compared with the other immunosuppressants; and Xu et al.’s meta-analysis (23) demonstrated that omalizumab versus standard treatment had a higher safety profile due to having fewer adverse reactions. It is worth mentioning that dupilumab was observed to be also effective and safety for the treatment of adult atopic dermatitis in special populations (24). On the basis of these previous findings, the second finding in our meta-analysis further confirm the general safety of the two biologics (i.e., dupilumab and omalizumab).

This meta-analysis has two advantages. First, this meta-analysis analyzed a great many SAEs outcomes, which succeeded in fully assessing the safety of the two biologics dupilumab and omalizumab. Second, there was no heterogeneity or only a little heterogeneity observed in most of the outcomes analyzed in this meta-analysis. In contrast, this meta-analysis has two main disadvantages. First, some SAEs outcomes had relatively lower incidences, which resulted in relatively wider 95% CIs of RRs. Those wider 95% CIs meant insufficient statistical power. Therefore, future studies are needed to confirm those results that had wider 95% CIs. Second, since the adequate data were not available, we failed to conduct subgroup analyses according to disease type and drug dose. Therefore, further conducting relevant subgroup analyses in future studies is useful to confirm and expand upon our findings.

## Conclusion

This meta-analysis for the first time assessed the association between use of dupilumab or omalizumab and incidences of various SAEs, and identified that neither dupilumab use nor omalizumab use was associated with the increased risks of any SAEs including but not limited to various infectious diseases. These findings further confirm the general safety of the two biologics dupilumab and omalizumab. In spite of this, the monitoring for adverse events should be constant and clinicians should be vigilant about it.

## Data availability statement

The original contributions presented in this study are included in this article/Supplementary material, further inquiries can be directed to the corresponding author.

## Author contributions

YX: Conceptualization, Data curation, Formal analysis, Writing – original draft, Writing – review and editing. WY: Data curation, Formal analysis, Writing – original draft, Writing – review and editing. MW: Data curation, Writing – original draft, Writing – review and editing.

## Supplementary material

Supplementary Figure 1 Forest plots of dupilumab.

Supplementary Figure 2 Forest plots of omalizumab.

Supplementary Table 1 Meta-analyses of dupilumab and 113 safety outcomes.

Supplementary Table 2 Meta-analyses of omalizumab and 61 safety outcomes.
