# Supplementary material for: A comprehensive analysis on the safety of two biologics dupilumab and omalizumab
Source: Front Med (Lausanne). 2024 Aug 8;11:1435370. doi: 10.3389/fmed.2024.1435370 (PMC11338893; doi:10.3389/fmed.2024.1435370)
Supplement: Supplementary Figure 1 — Forest plots of dupilumab. [file Image_1.PDF]

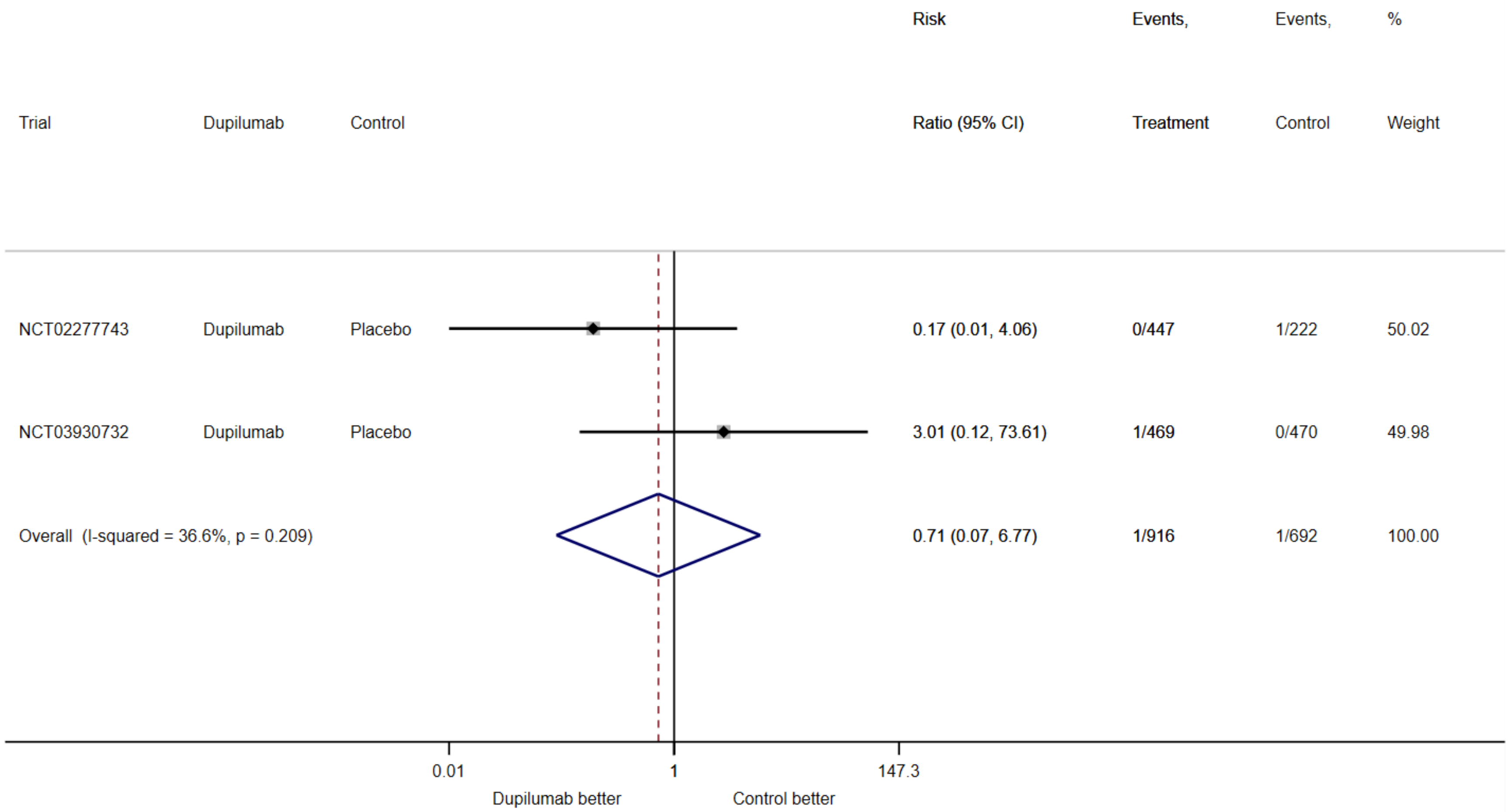

1_Blood And Lymphatic System Disorders-Figure S1 Meta-analysis of Dupilumab and Anaemia

Trial	Dupilumab	Control	Risk Ratio (95% CI)	Events, Treatment	Events, Control	% Weight
NCT01854047	Dupilumab	Placebo	0.78 (0.03, 19.04)	1/611	0/158	24.32
NCT02414854	Dupilumab	Placebo	1.51 (0.06, 36.94)	1/1263	0/634	24.27
NCT02898454	Dupilumab	Placebo	1.52 (0.06, 37.09)	1/297	0/150	24.34
NCT02948959	Dupilumab	Placebo	2.48 (0.12, 51.33)	2/271	0/134	27.07
Overall (I-squared = 0.0%, p = 0.966)			1.47 (0.30, 7.12)	5/2442	0/1076	100.00

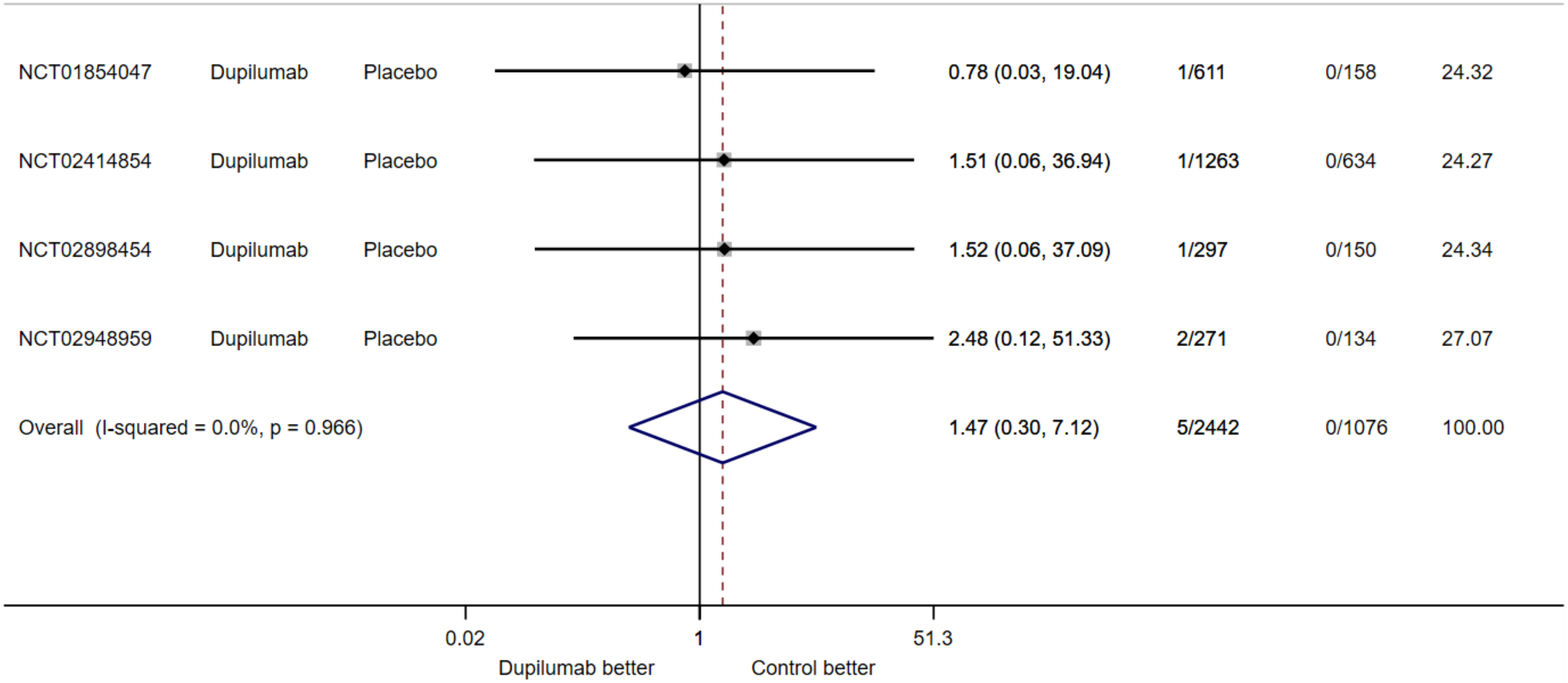

1_Blood And Lymphatic System Disorders-Figure S2 Meta-analysis of Dupilumab and Eosinophilia

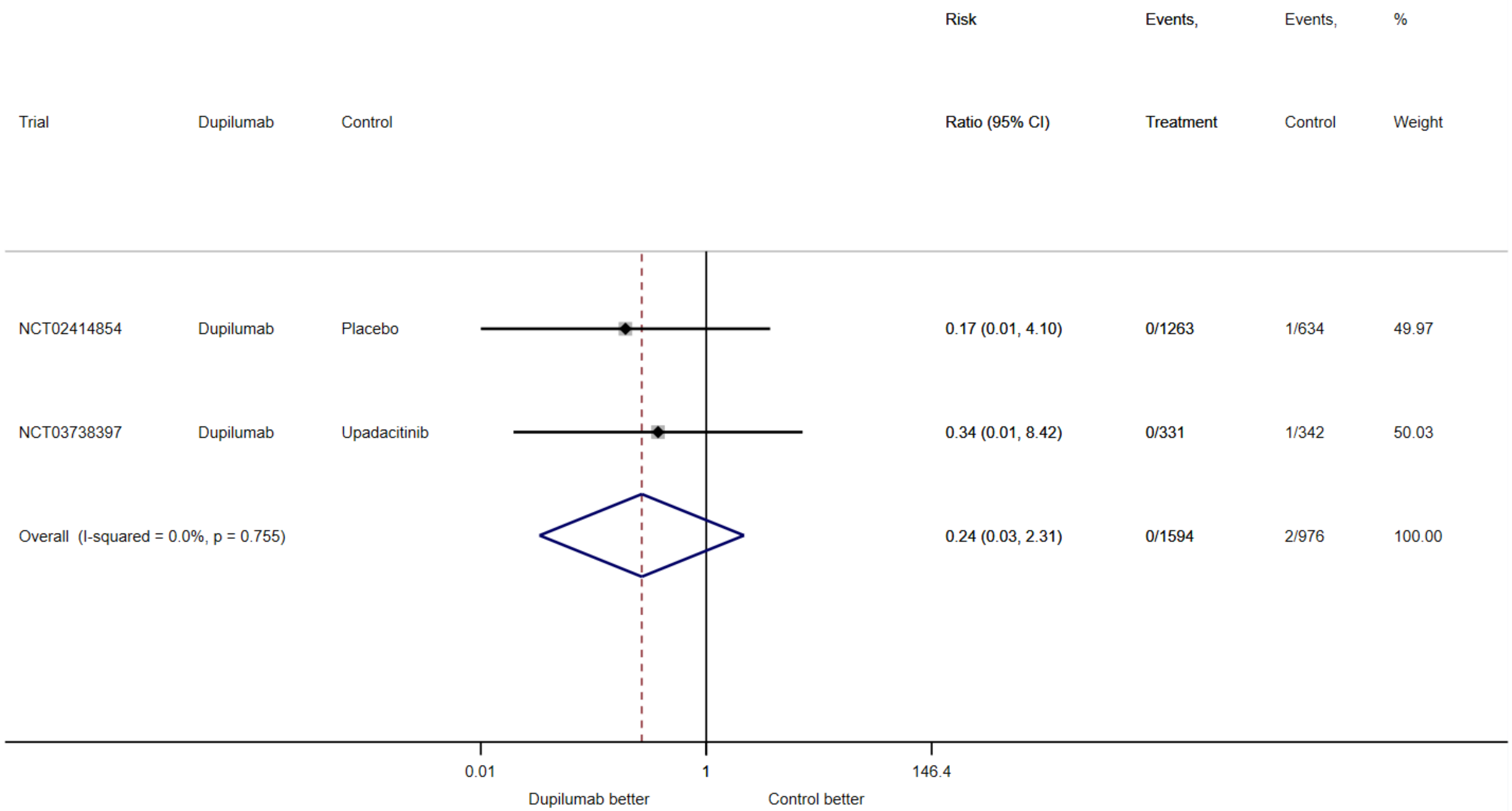

1_Blood And Lymphatic System Disorders-Figure S3 Meta-analysis of Dupilumab and Neutropenia

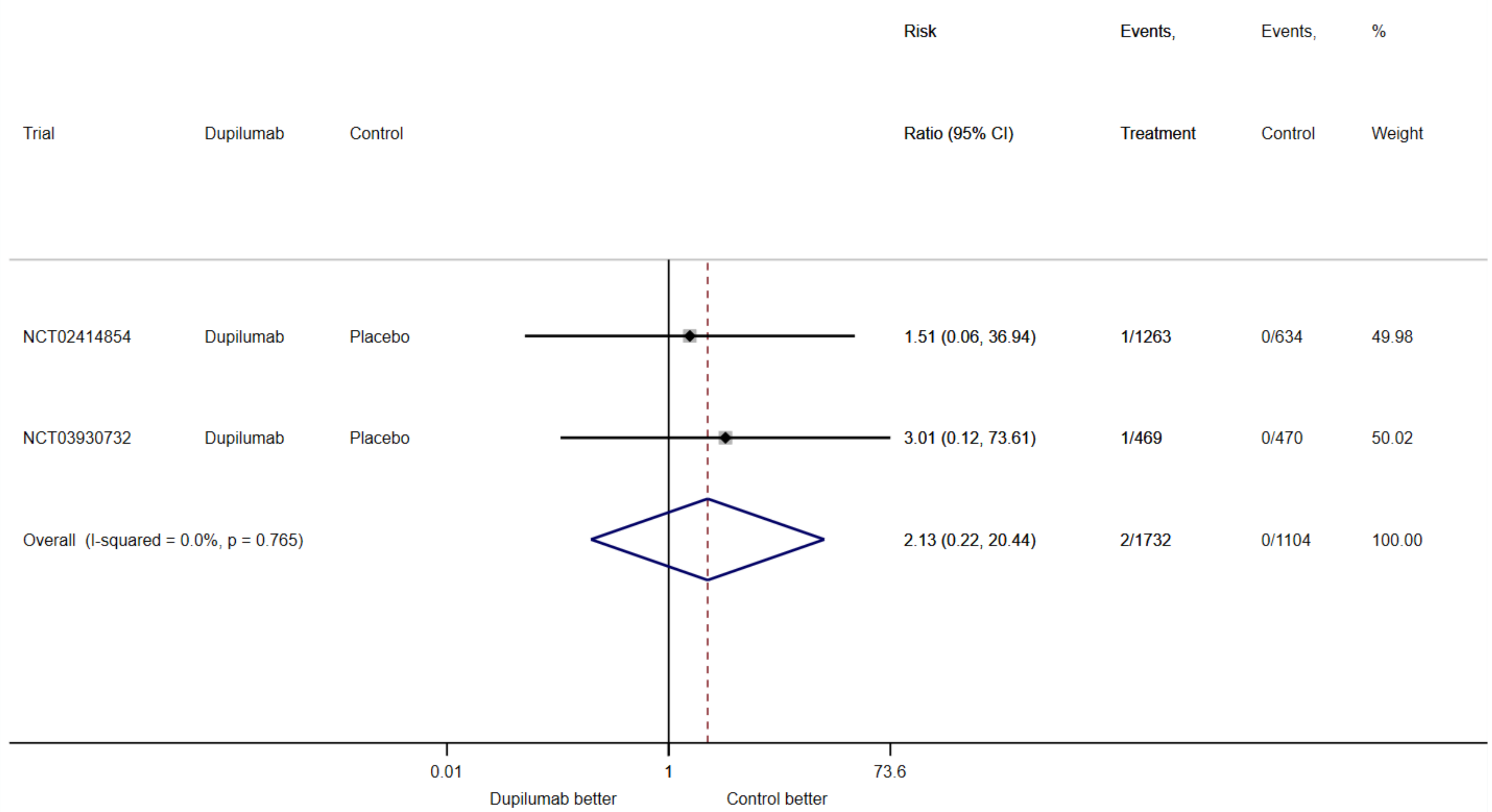

2_Cardiac Disorders-Figure S1 Meta-analysis of Dupilumab and Acute coronary syndrome

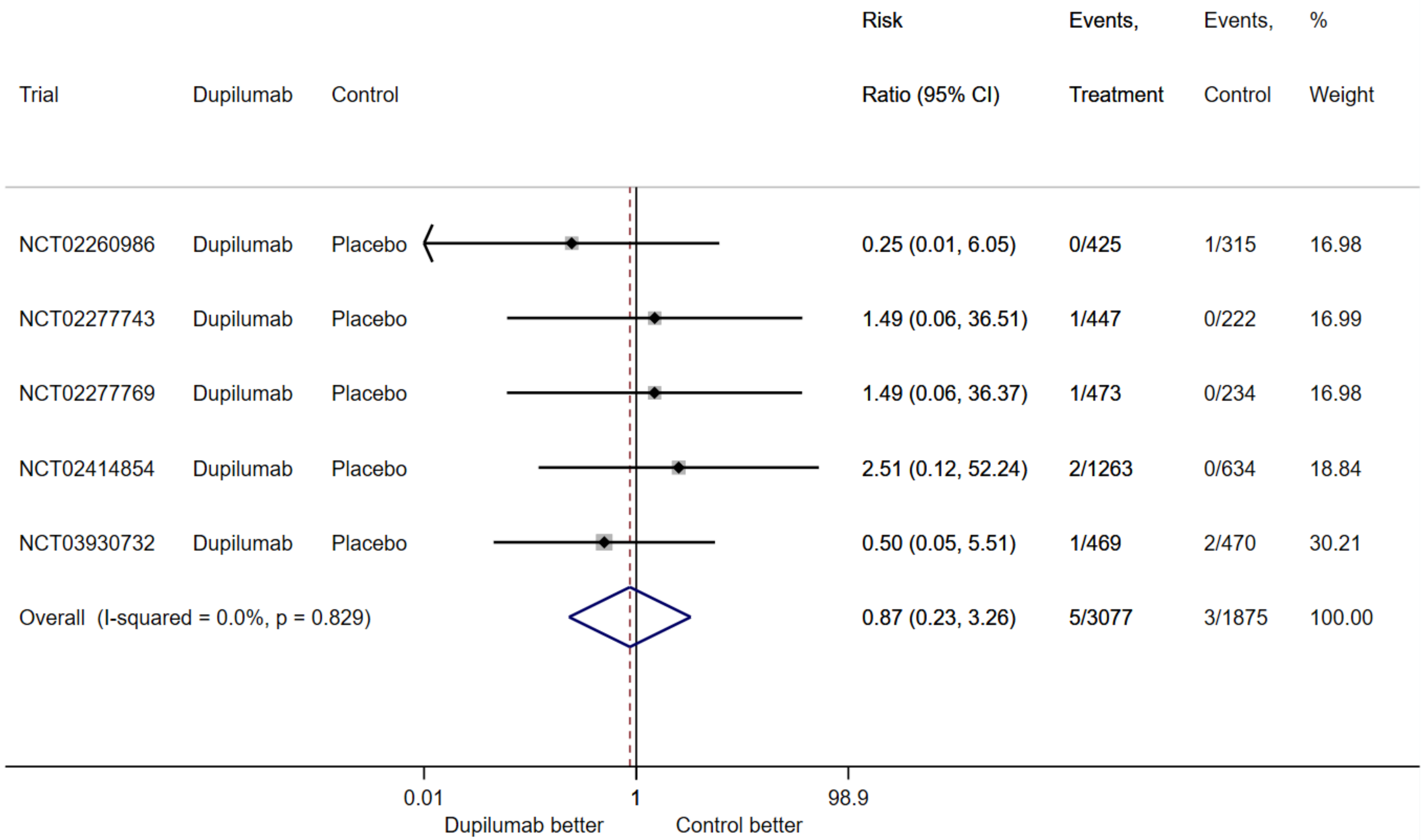

2_Cardiac Disorders-Figure S2 Meta-analysis of Dupilumab and Acute myocardial infarction

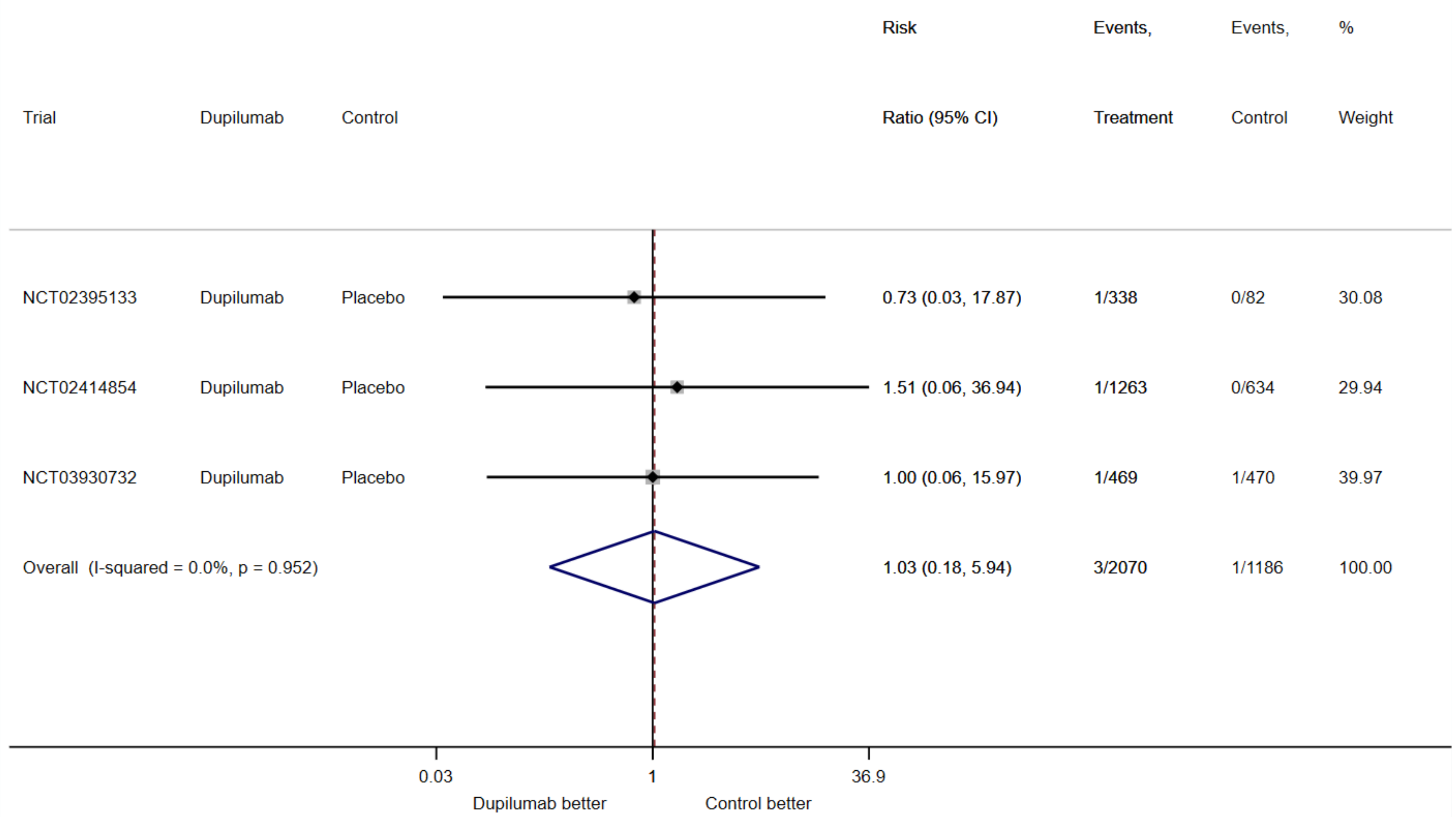

2_Cardiac Disorders-Figure S3 Meta-analysis of Dupilumab and Atrial fibrillation

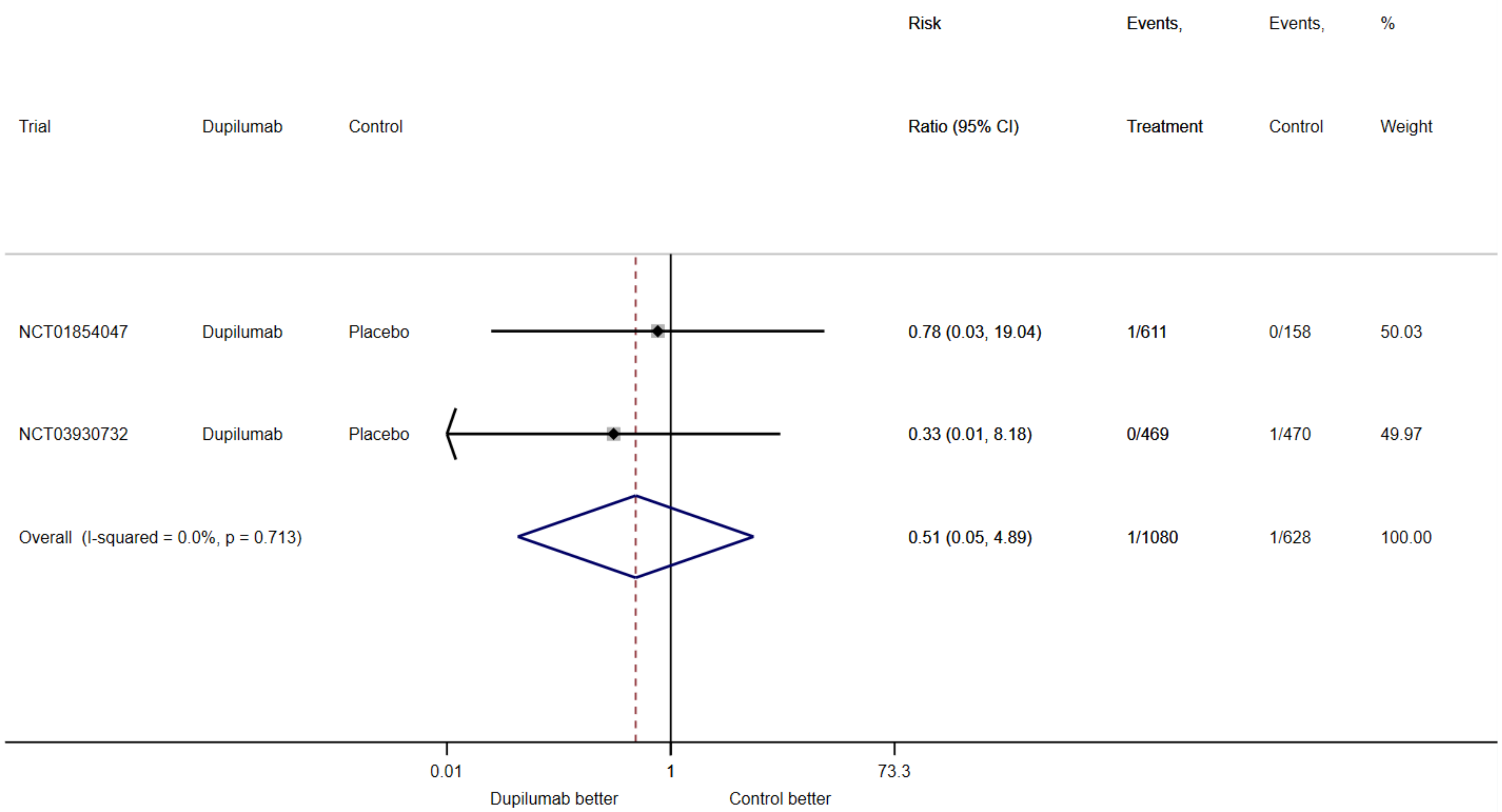

2_Cardiac Disorders-Figure S4 Meta-analysis of Dupilumab and Atrioventricular block complete

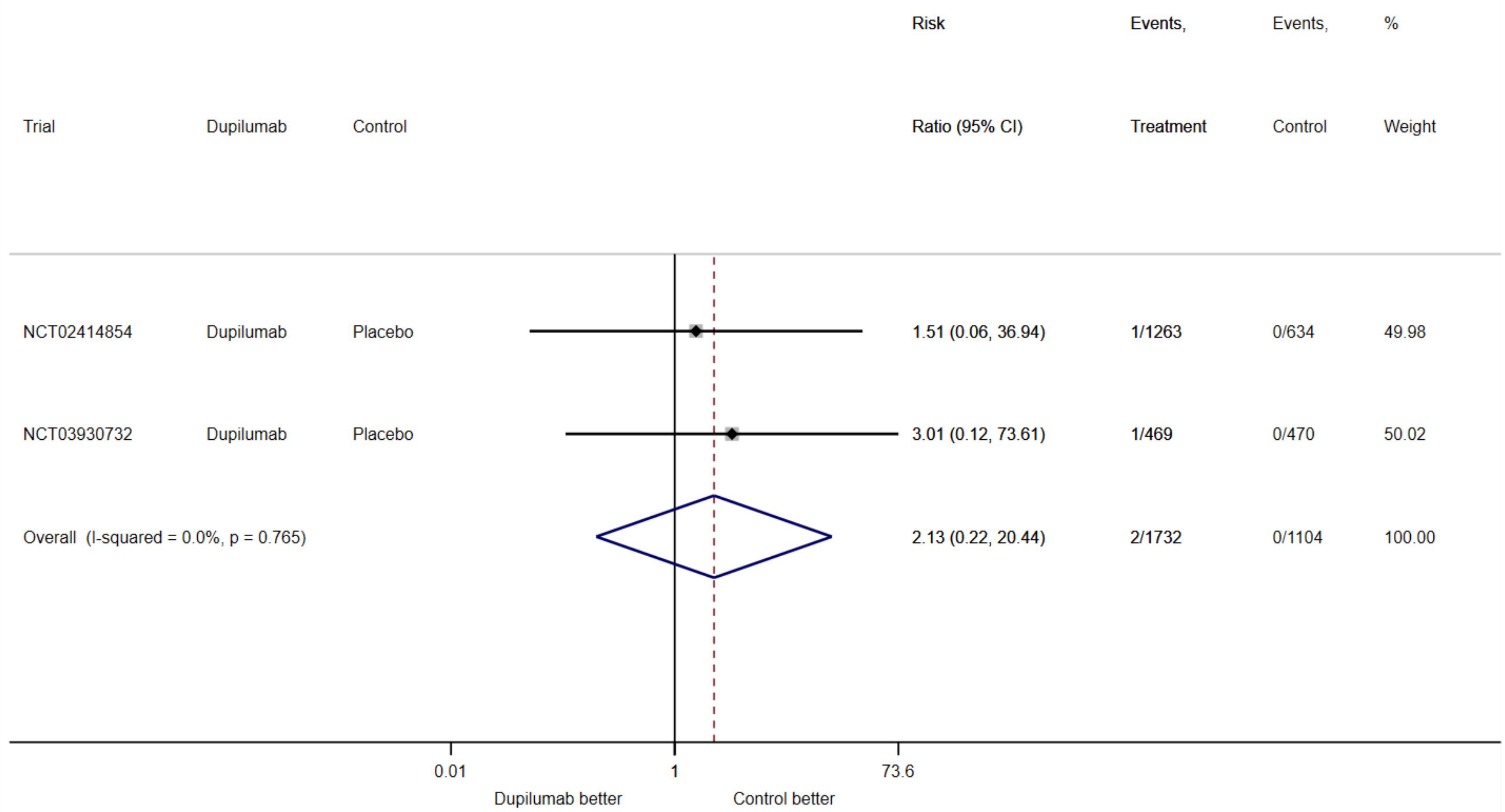

2_Cardiac Disorders-Figure S5 Meta-analysis of Dupilumab and Atrioventricular block second degree

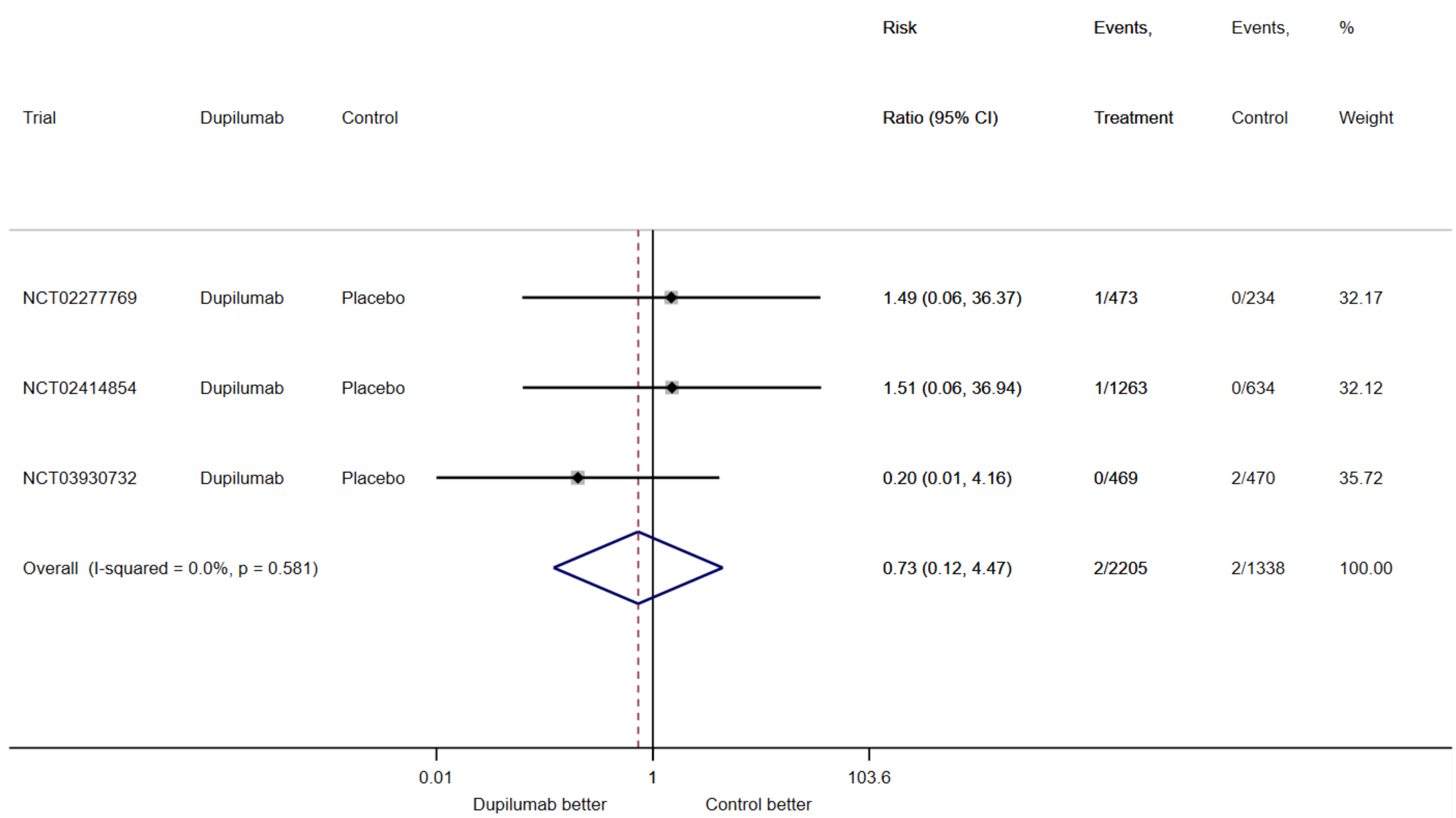

2_Cardiac Disorders-Figure S6 Meta-analysis of Dupilumab and Cardiac failure congestive

Trial	Dupilumab	Control	Risk Ratio (95% CI)	Events, Treatment	Events, Control	% Weight
NCT02414854	Dupilumab	Placebo	2.51 (0.12, 52.24)	2/1263	0/634	52.60
NCT04345367	Dupilumab	Abrocitinib	0.33 (0.01, 8.09)	0/365	1/362	47.40
Overall (I-squared = 0.0%, p = 0.367)			0.96 (0.11, 8.68)	2/1628	1/996	100.00

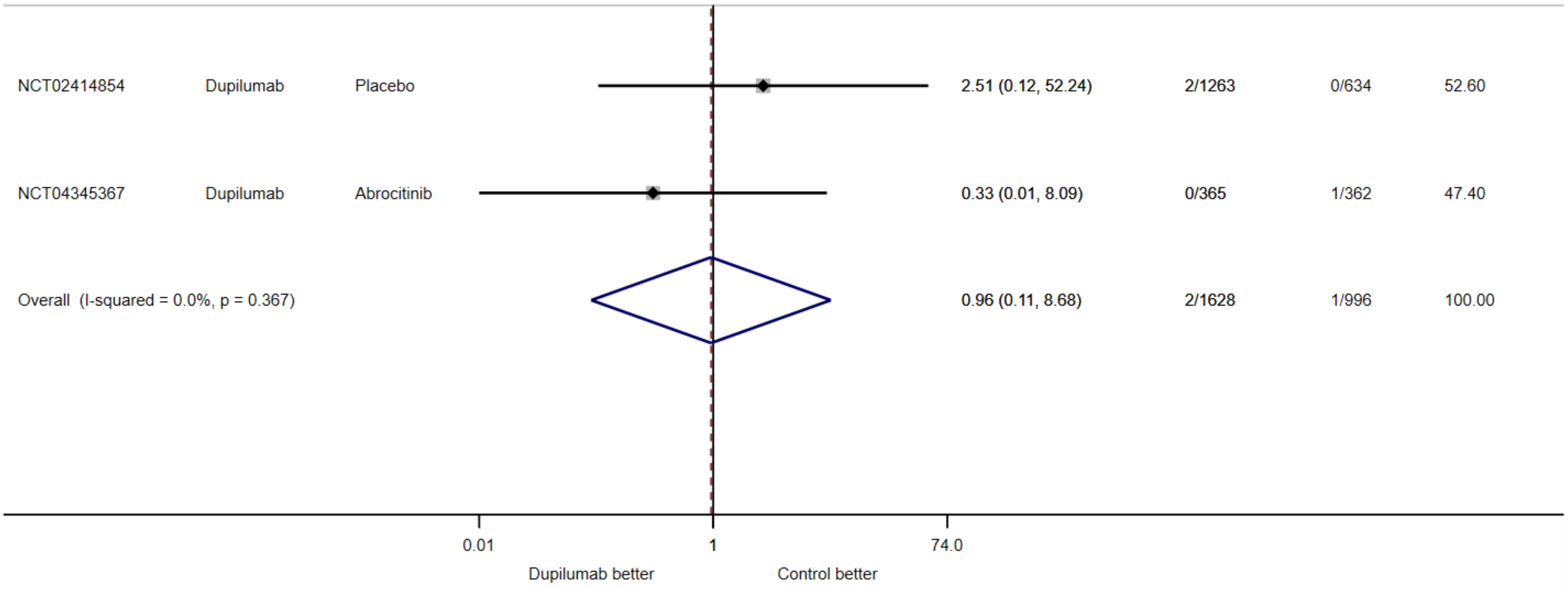

2_Cardiac Disorders-Figure S7 Meta-analysis of Dupilumab and Cardio-respiratory arrest

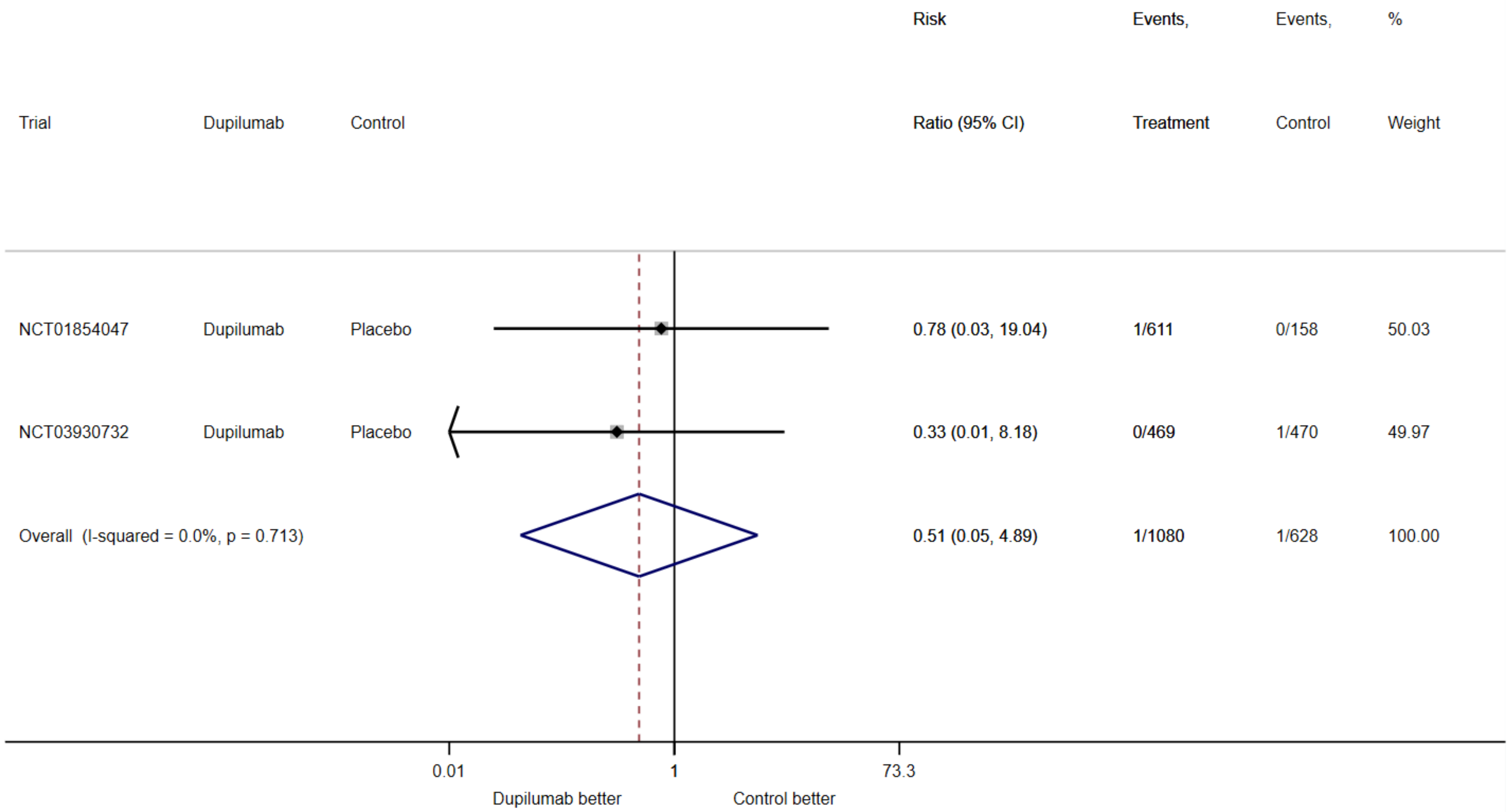

2_Cardiac Disorders-Figure S8 Meta-analysis of Dupilumab and Cor pulmonale acute

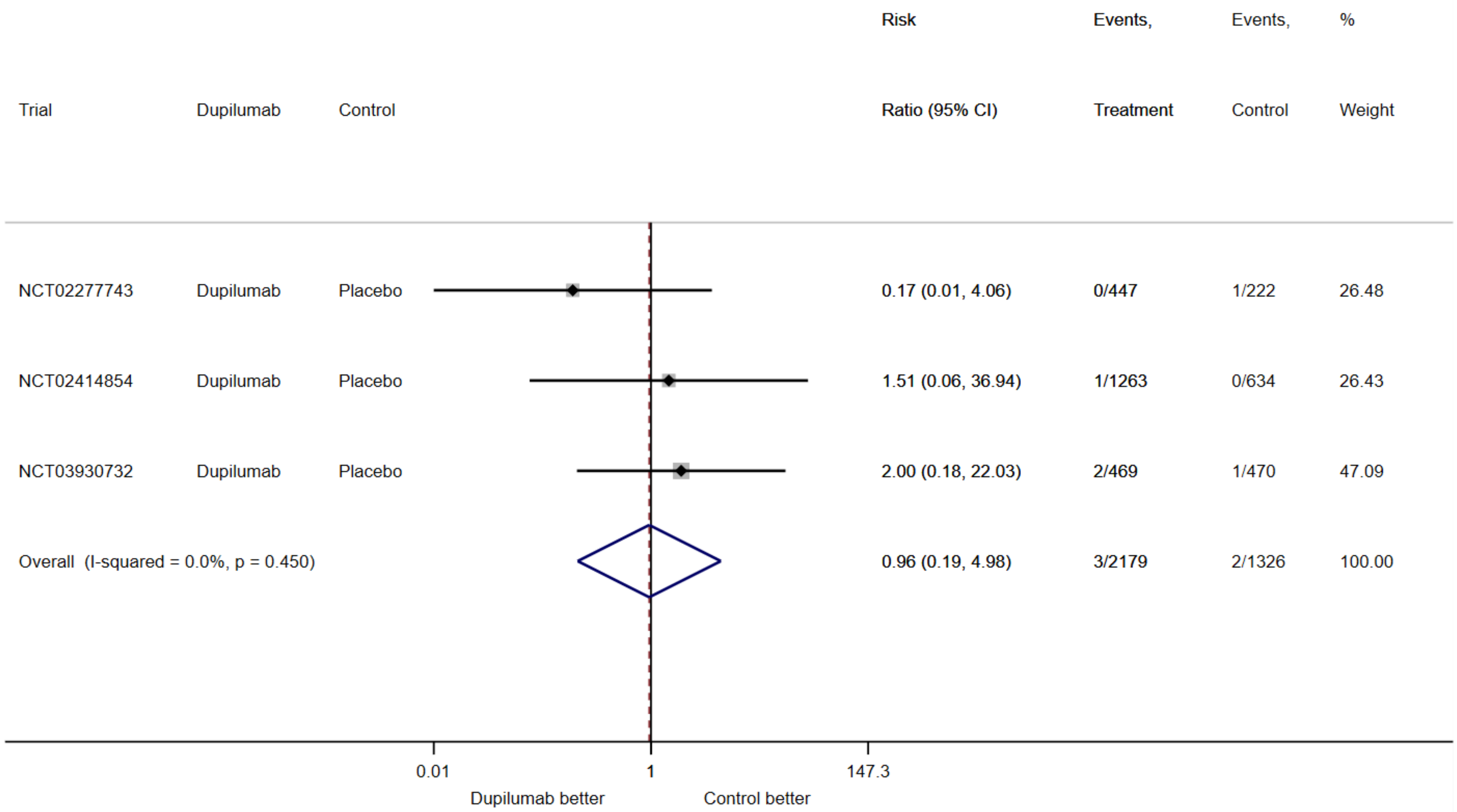

2_Cardiac Disorders-Figure S9 Meta-analysis of Dupilumab and Coronary artery disease

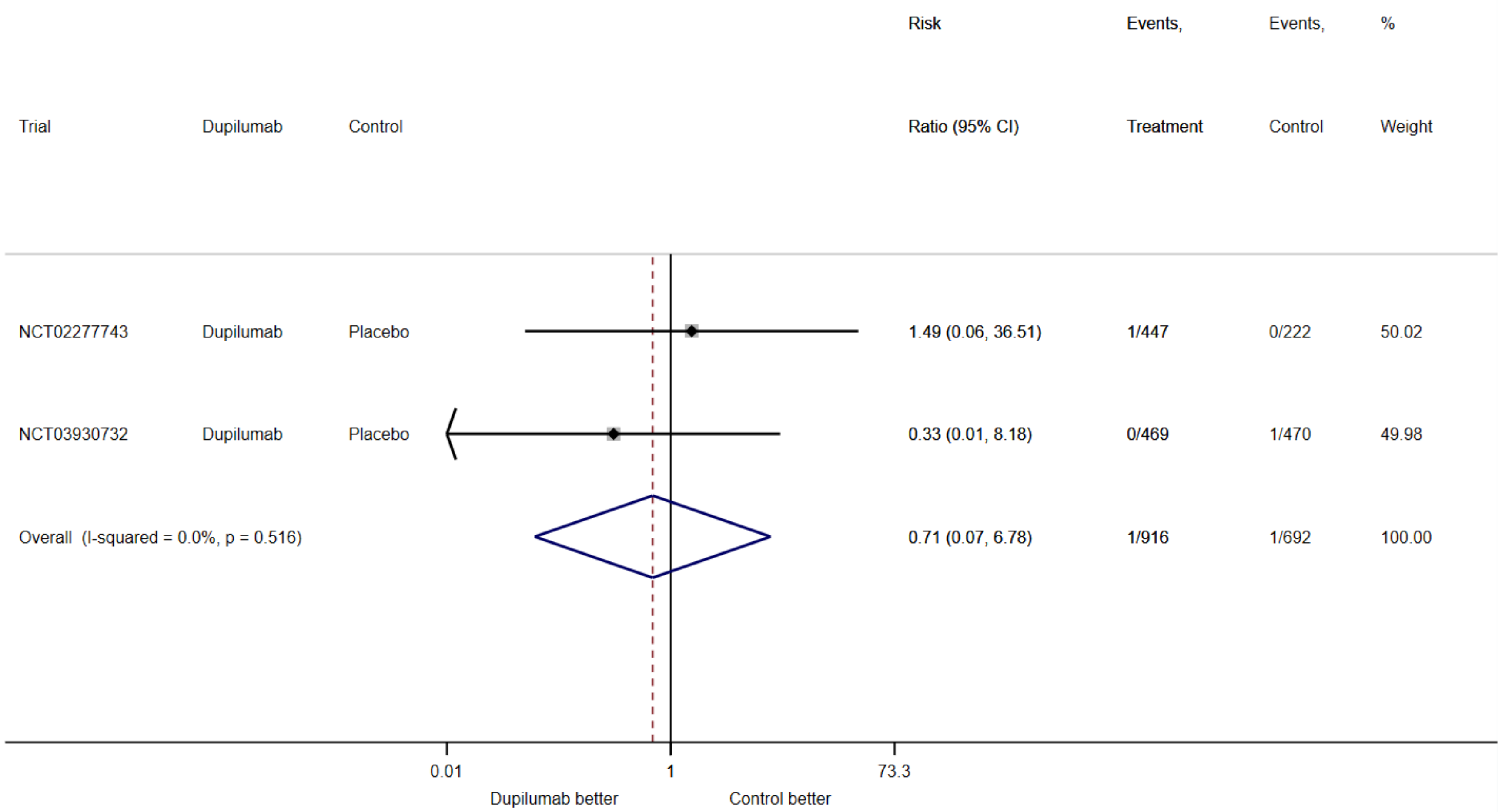

2_Cardiac Disorders-Figure S10 Meta-analysis of Dupilumab and Myocardial infarction

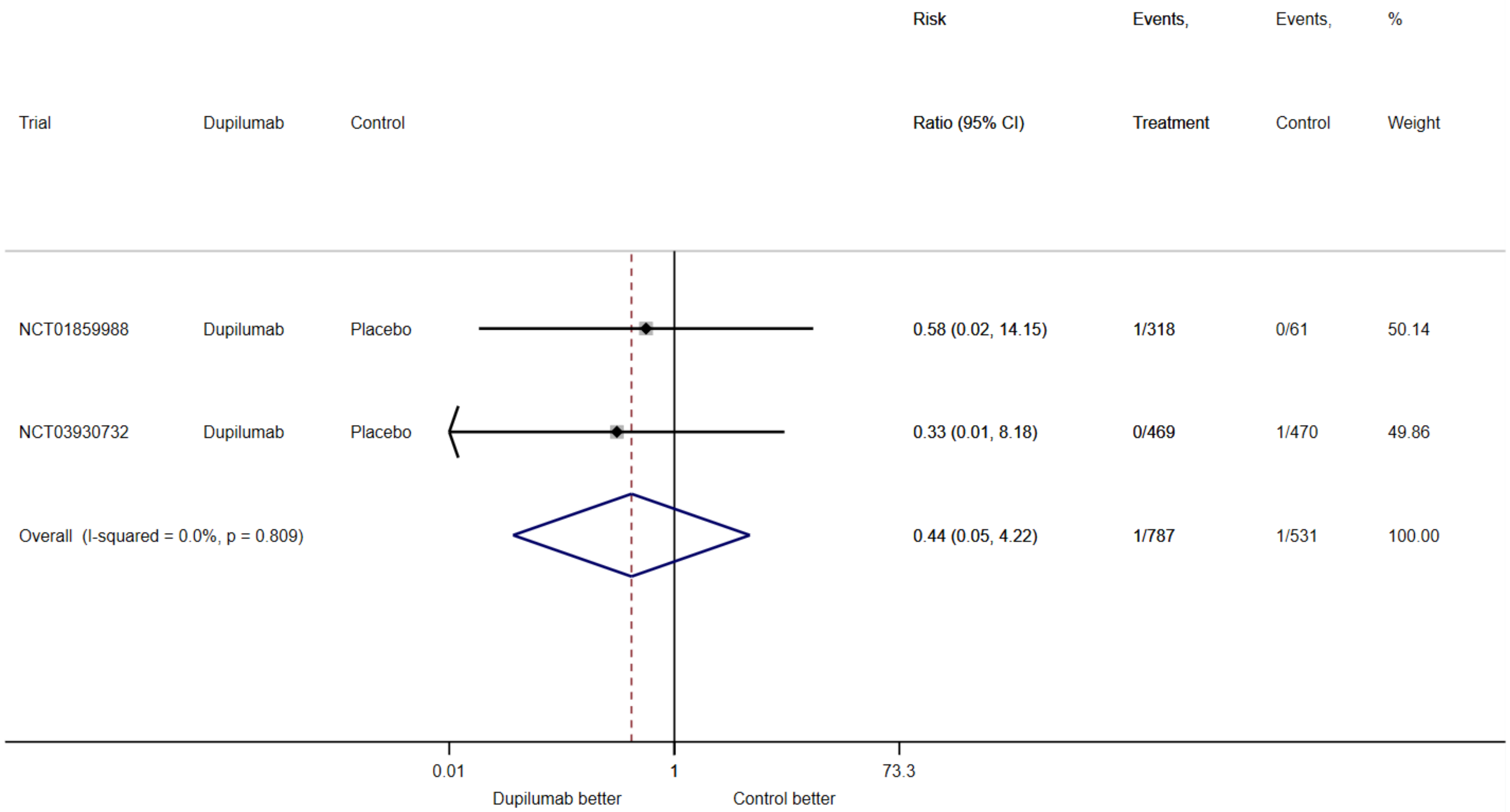

2_Cardiac Disorders-Figure S11 Meta-analysis of Dupilumab and Tachycardia

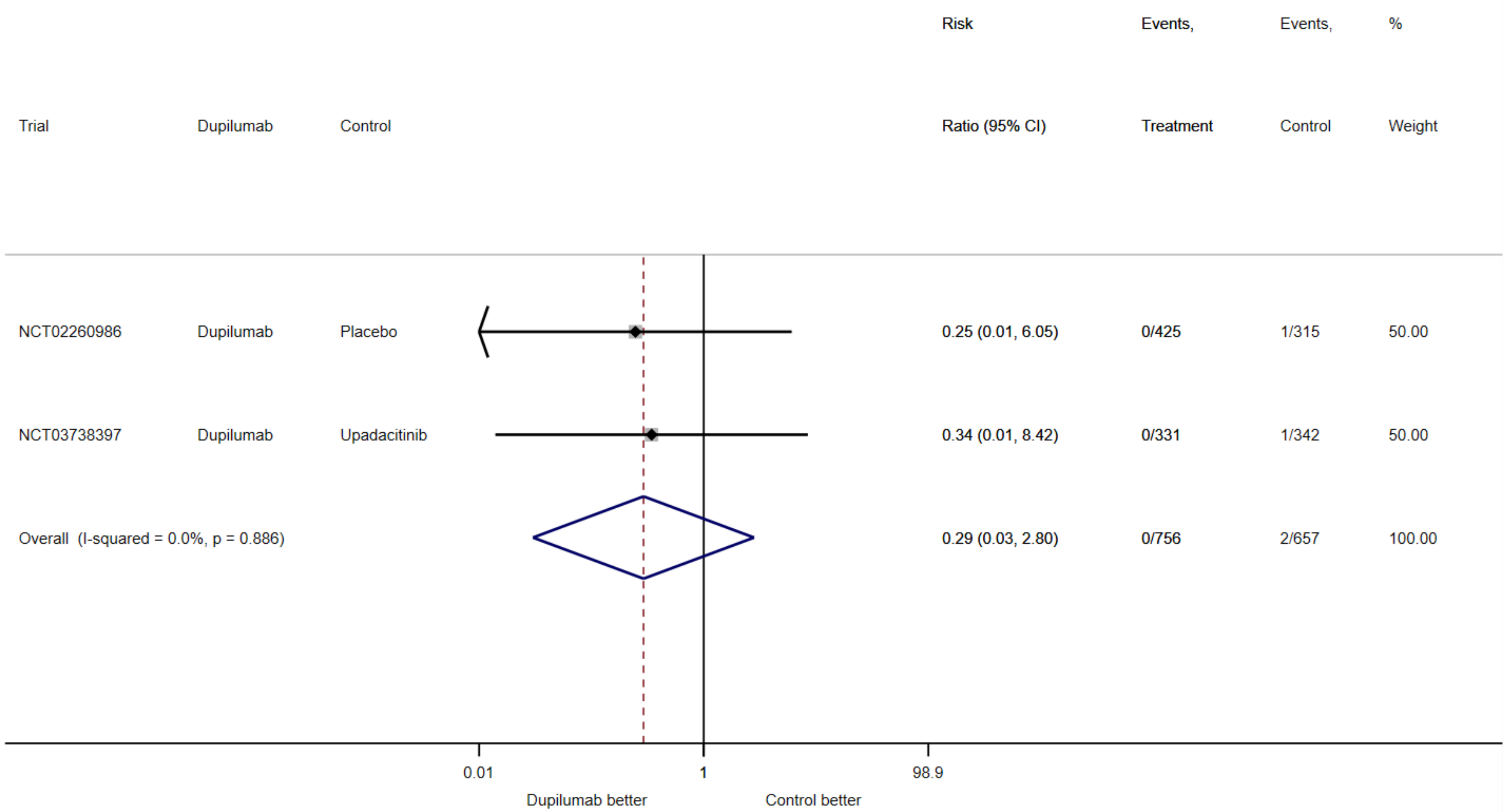

3_Eye Disorders-Figure S1 Meta-analysis of Dupilumab and Glaucoma

Trial	Dupilumab	Control	Risk Ratio (95% CI)	Events, Treatment	Events, Control	% Weight
NCT02277769	Dupilumab	Placebo	1.49 (0.06, 36.37)	1/473	0/234	16.66
NCT02414854	Dupilumab	Placebo	1.51 (0.06, 36.94)	1/1263	0/634	16.64
NCT02898454	Dupilumab	Placebo	0.17 (0.01, 4.12)	0/297	1/150	16.69
NCT03633617	Dupilumab	Placebo	1.74 (0.07, 42.26)	1/203	0/117	16.71
NCT03720470	Dupilumab	Placebo/PF-04965842	0.82 (0.03, 20.00)	0/242	1/595	16.66
NCT03930732	Dupilumab	Placebo	0.33 (0.01, 8.18)	0/469	1/470	16.65
Overall (I-squared = 0.0%, p = 0.888)			0.75 (0.20, 2.77)	3/2947	3/2200	100.00

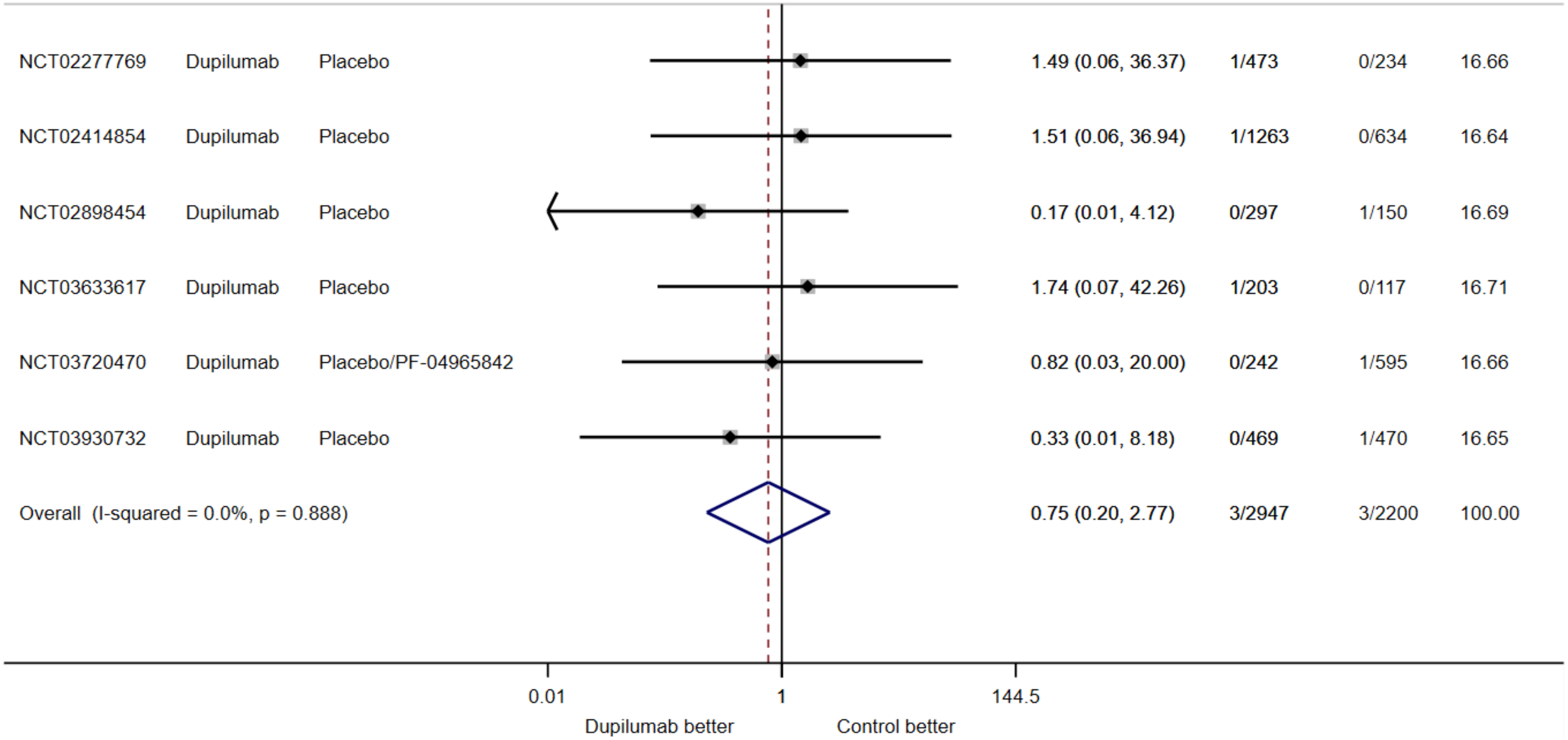

4_Gastrointestinal Disorders-Figure S1 Meta-analysis of Dupilumab and Abdominal pain

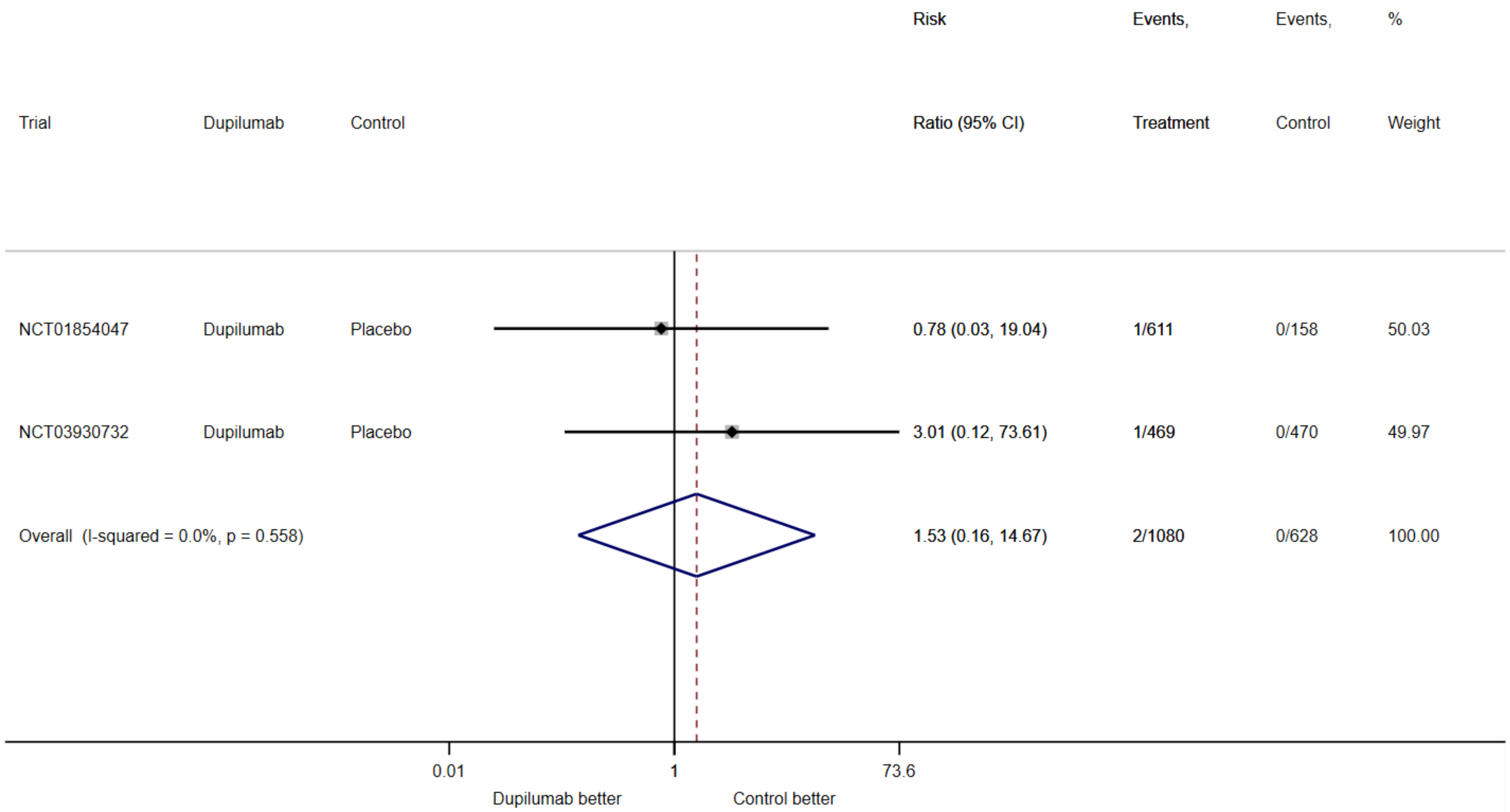

4_Gastrointestinal Disorders-Figure S2 Meta-analysis of Dupilumab and Colitis

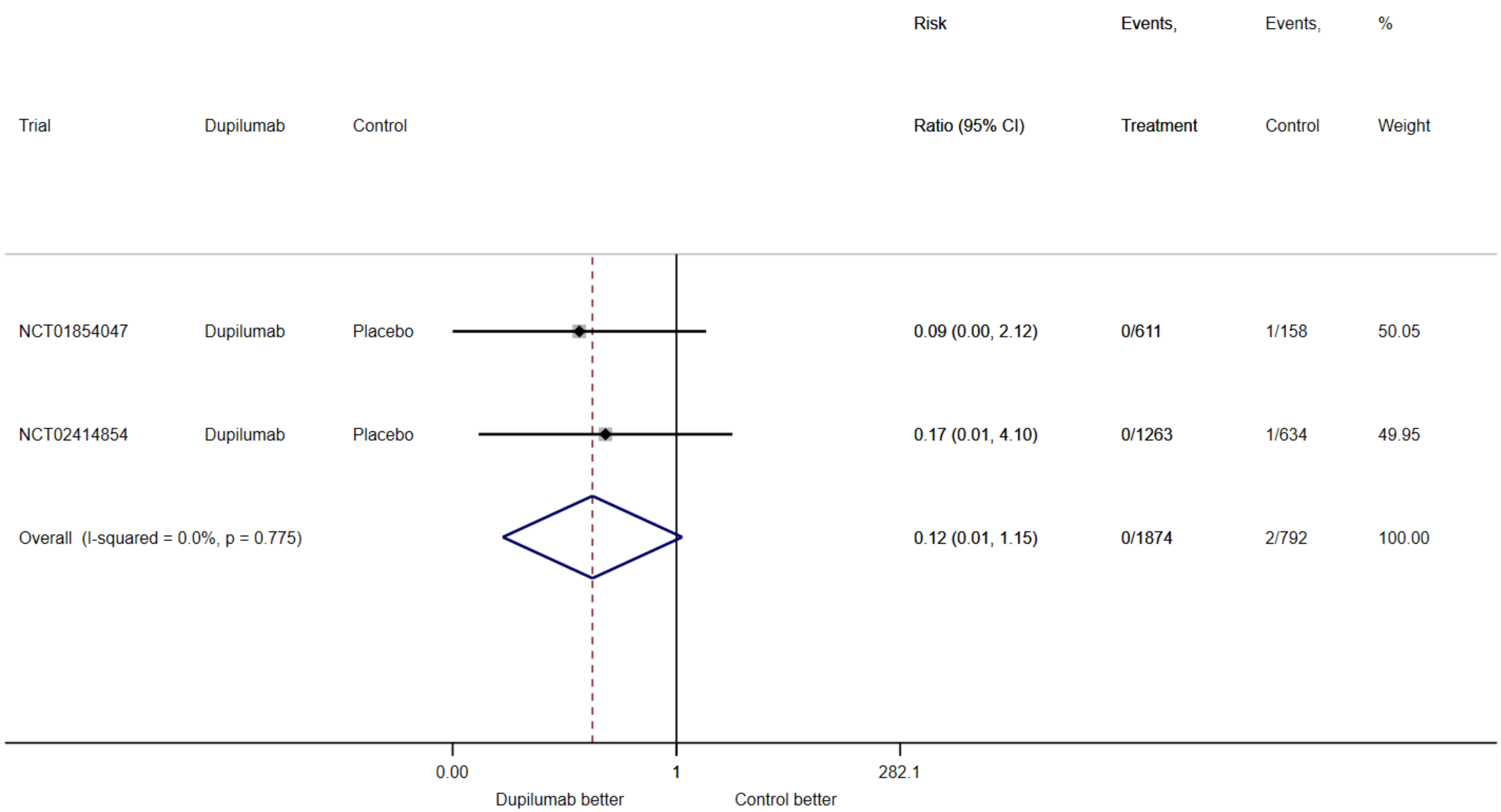

4_Gastrointestinal Disorders-Figure S3 Meta-analysis of Dupilumab and Large intestine polyp

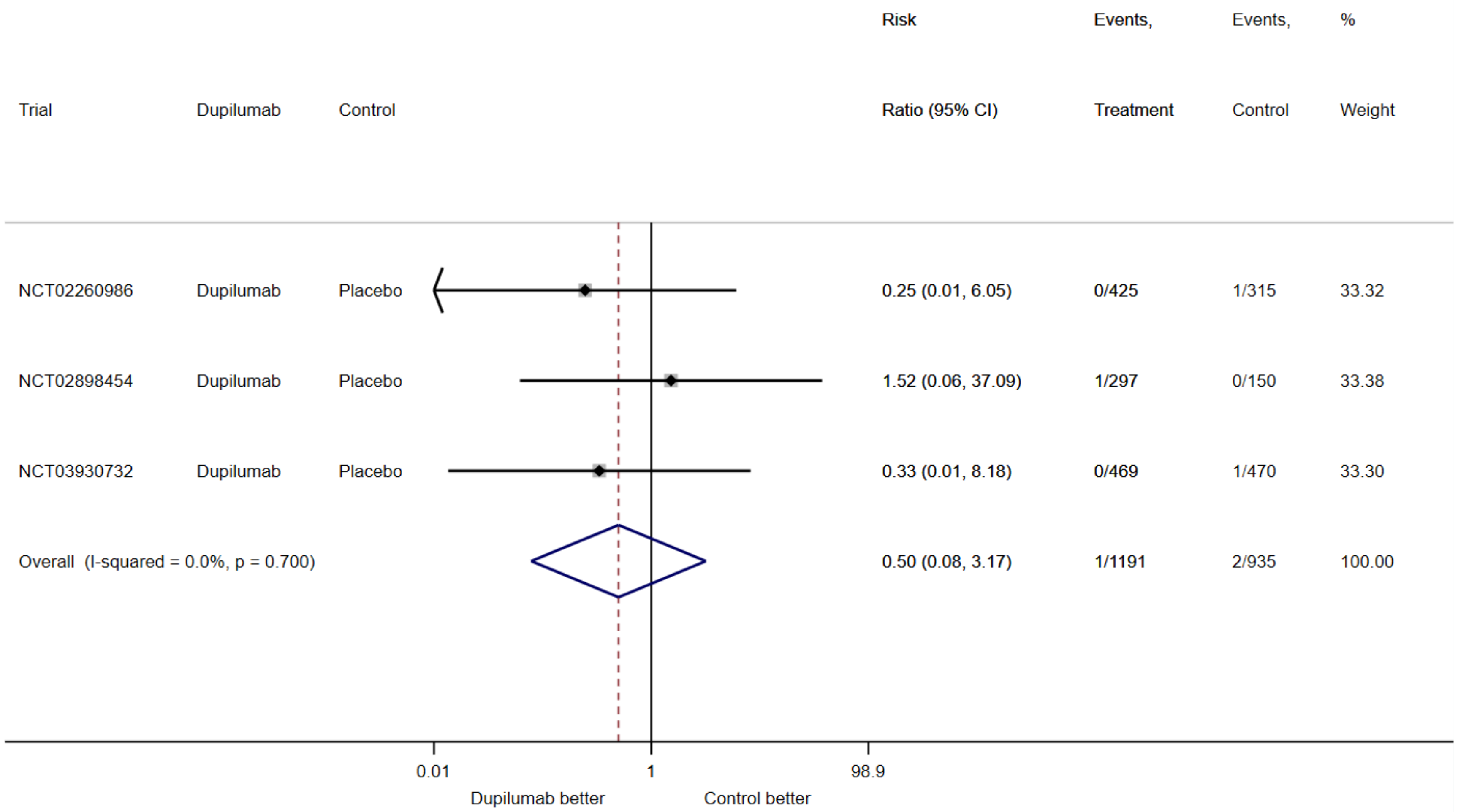

4_Gastrointestinal Disorders-Figure S4 Meta-analysis of Dupilumab and Pancreatitis

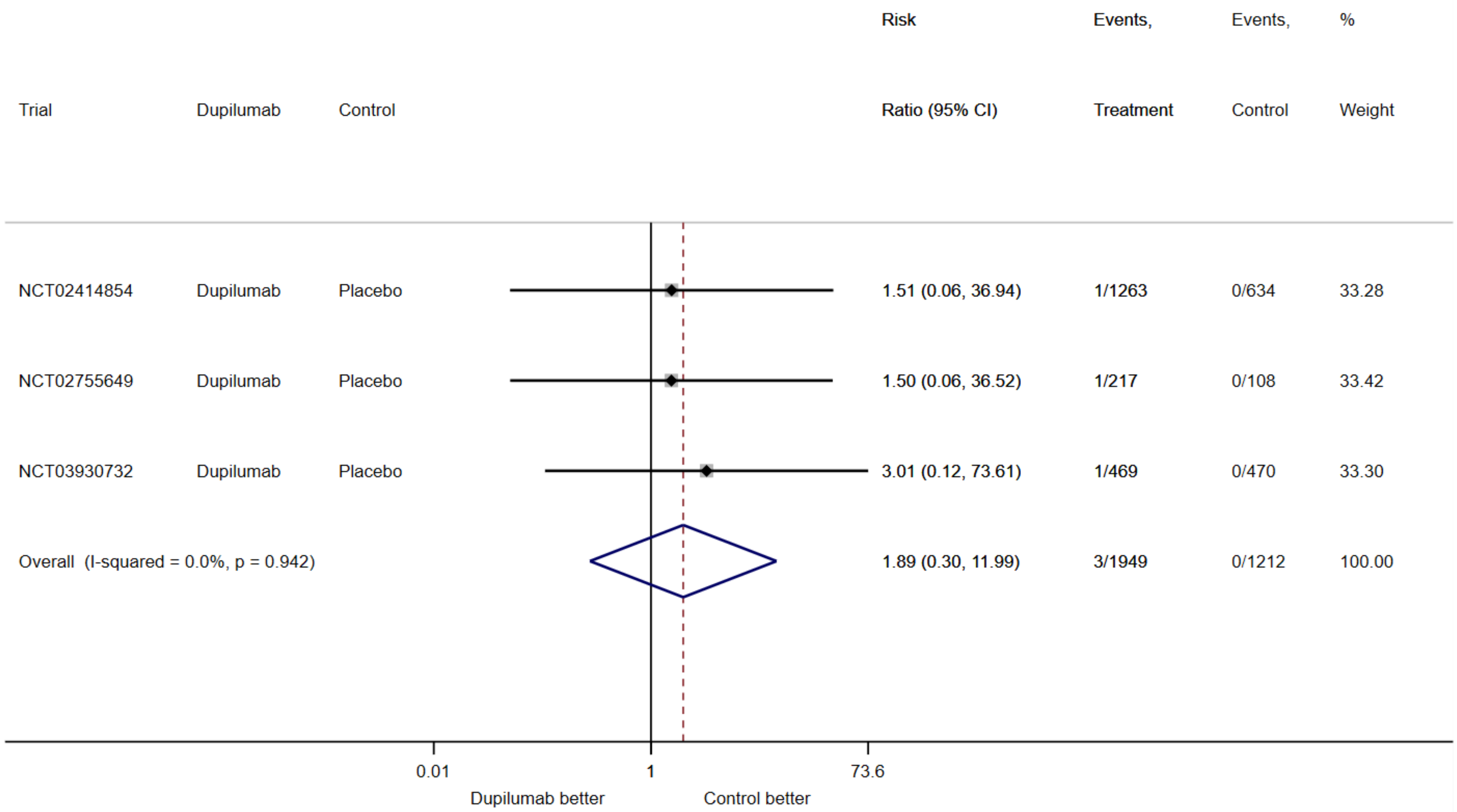

4_Gastrointestinal Disorders-Figure S5 Meta-analysis of Dupilumab and Pancreatitis acute

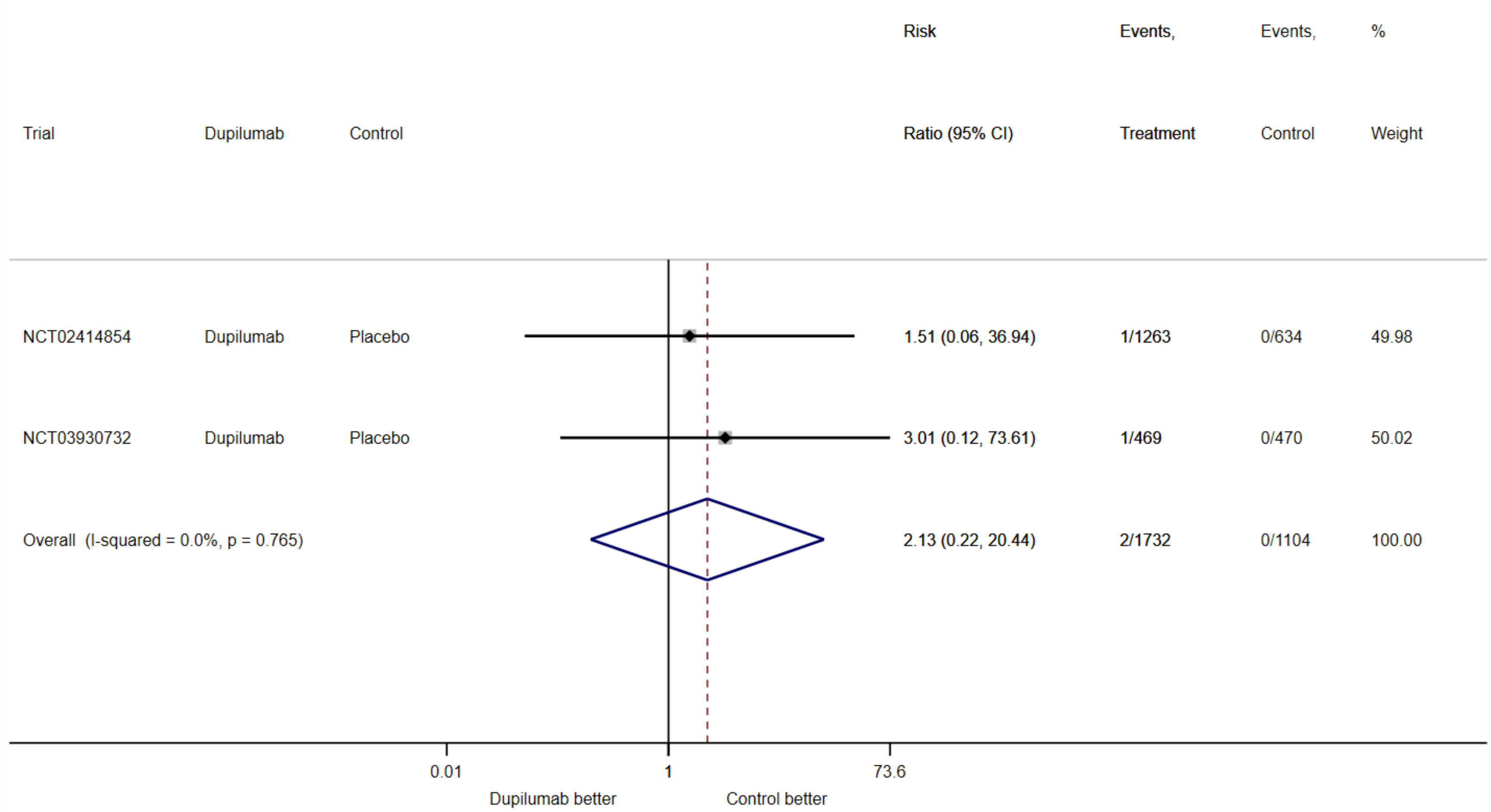

4_Gastrointestinal Disorders-Figure S6 Meta-analysis of Dupilumab and Umbilical hernia

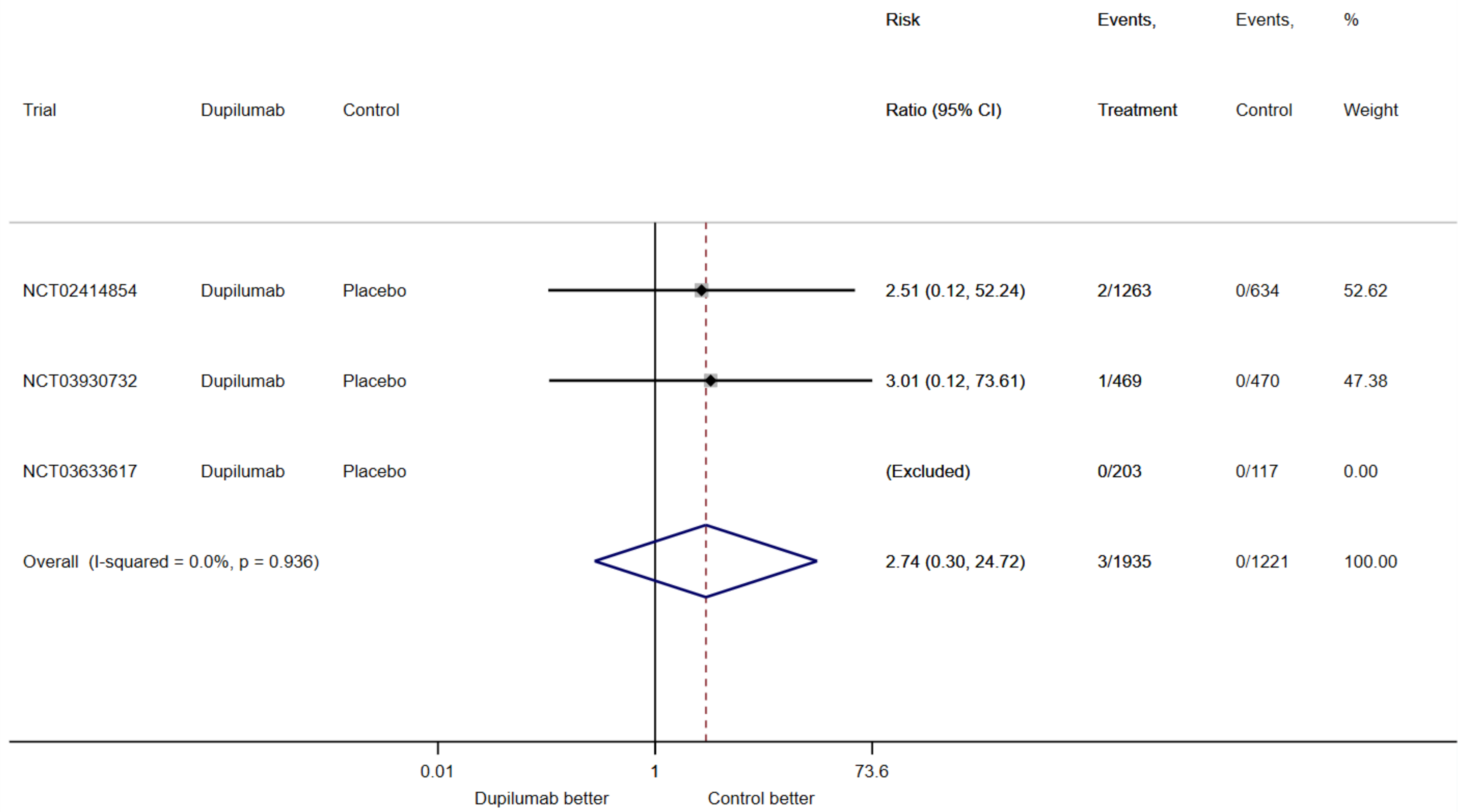

5_General Disorders-Figure S1 Meta-analysis of Dupilumab and Chest pain

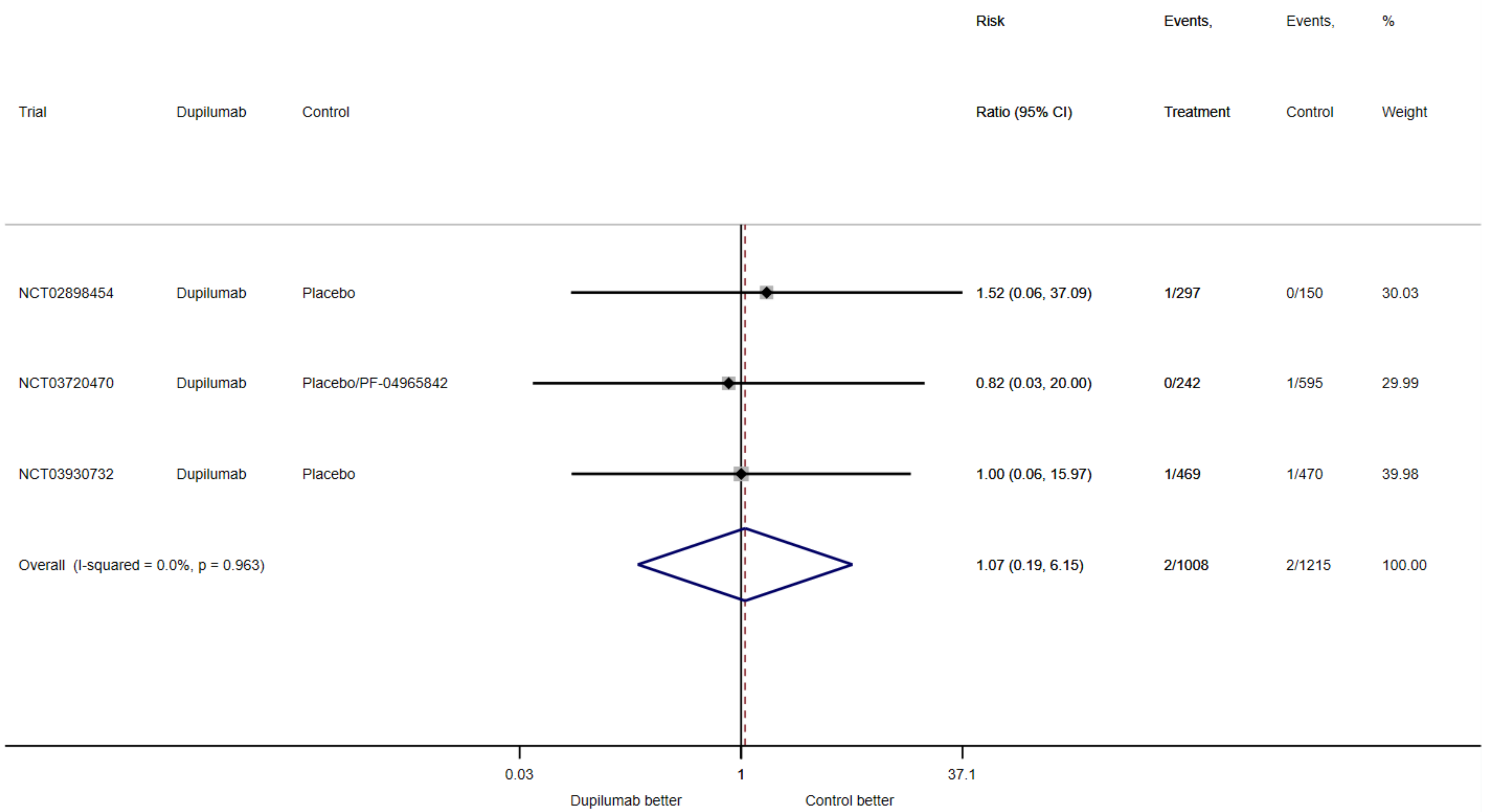

5_General Disorders-Figure S2 Meta-analysis of Dupilumab and Pyrexia

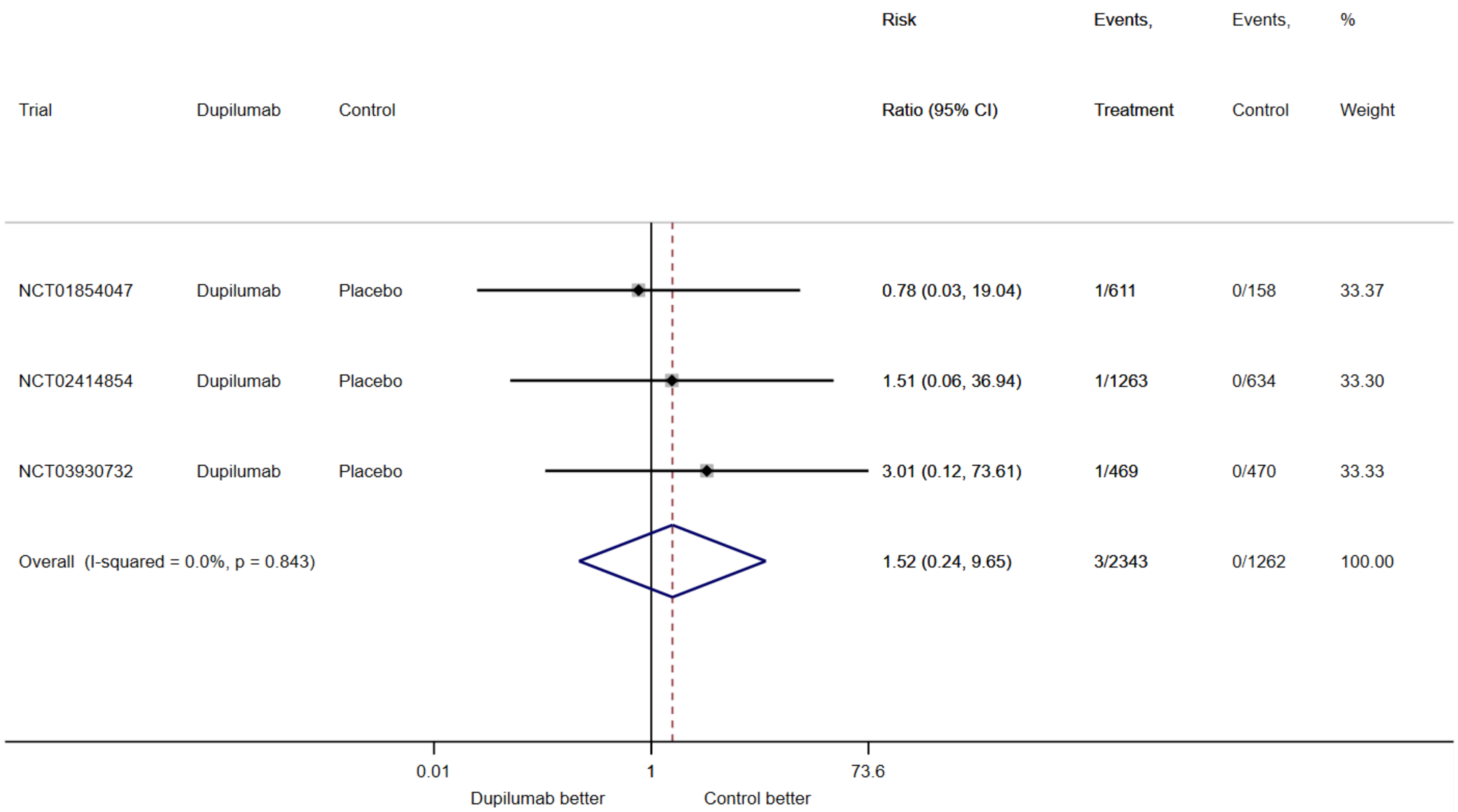

6_Hepatobiliary Disorders-Figure S1 Meta-analysis of Dupilumab and Cholecystitis

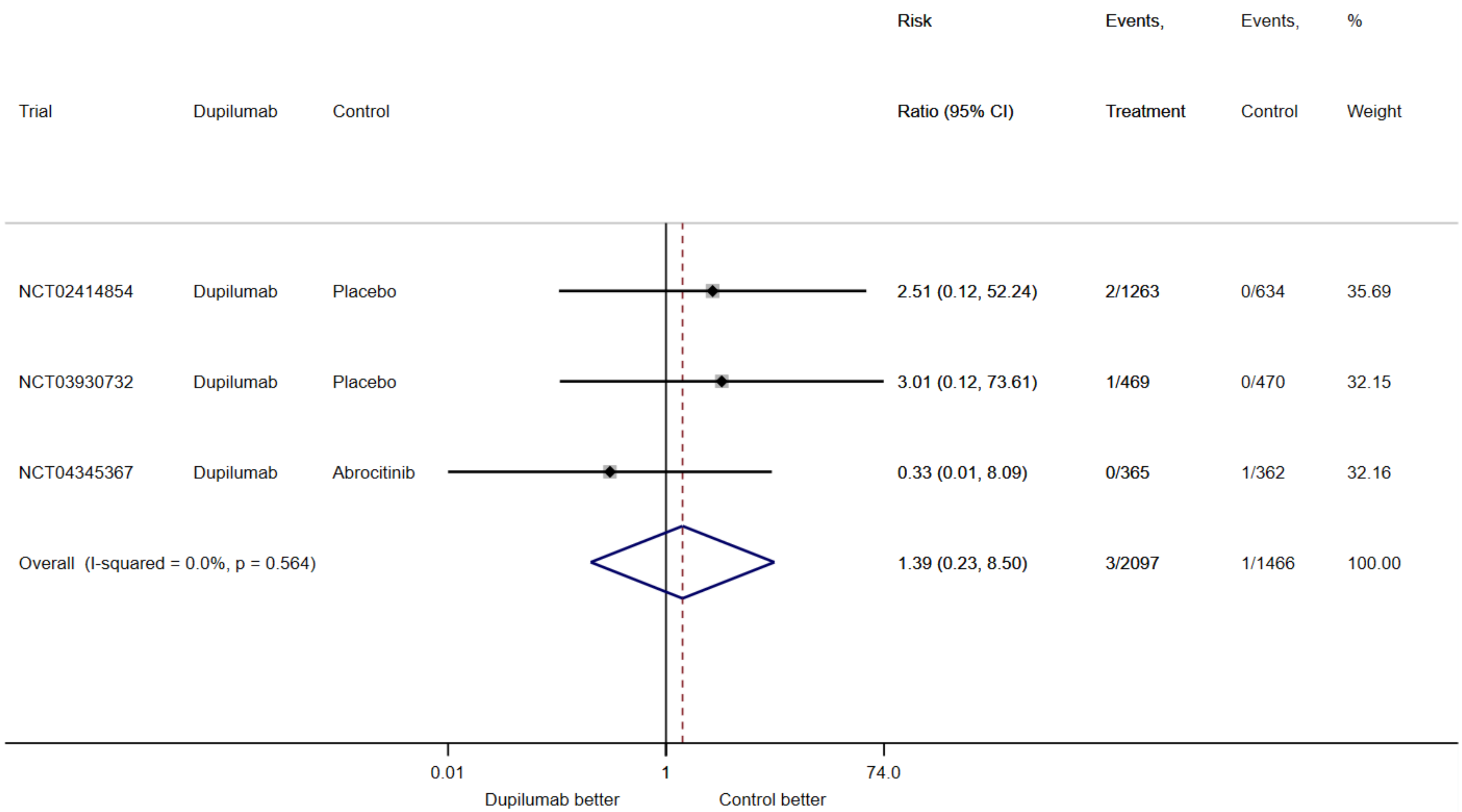

6_Hepatobiliary Disorders-Figure S2 Meta-analysis of Dupilumab and Cholecystitis acute

Trial	Dupilumab	Control	Risk Ratio (95% CI)	Events, Treatment	Events, Control	% Weight
NCT02260986	Dupilumab	Placebo	2.23 (0.09, 54.45)	1/425	0/315	25.00
NCT02414854	Dupilumab	Placebo	1.51 (0.06, 36.94)	1/1263	0/634	24.97
NCT02898454	Dupilumab	Placebo	0.17 (0.01, 4.12)	0/297	1/150	25.04
NCT03930732	Dupilumab	Placebo	3.01 (0.12, 73.61)	1/469	0/470	24.99
Overall (I-squared = 0.0%, p = 0.589)			1.14 (0.23, 5.64)	3/2454	1/1569	100.00

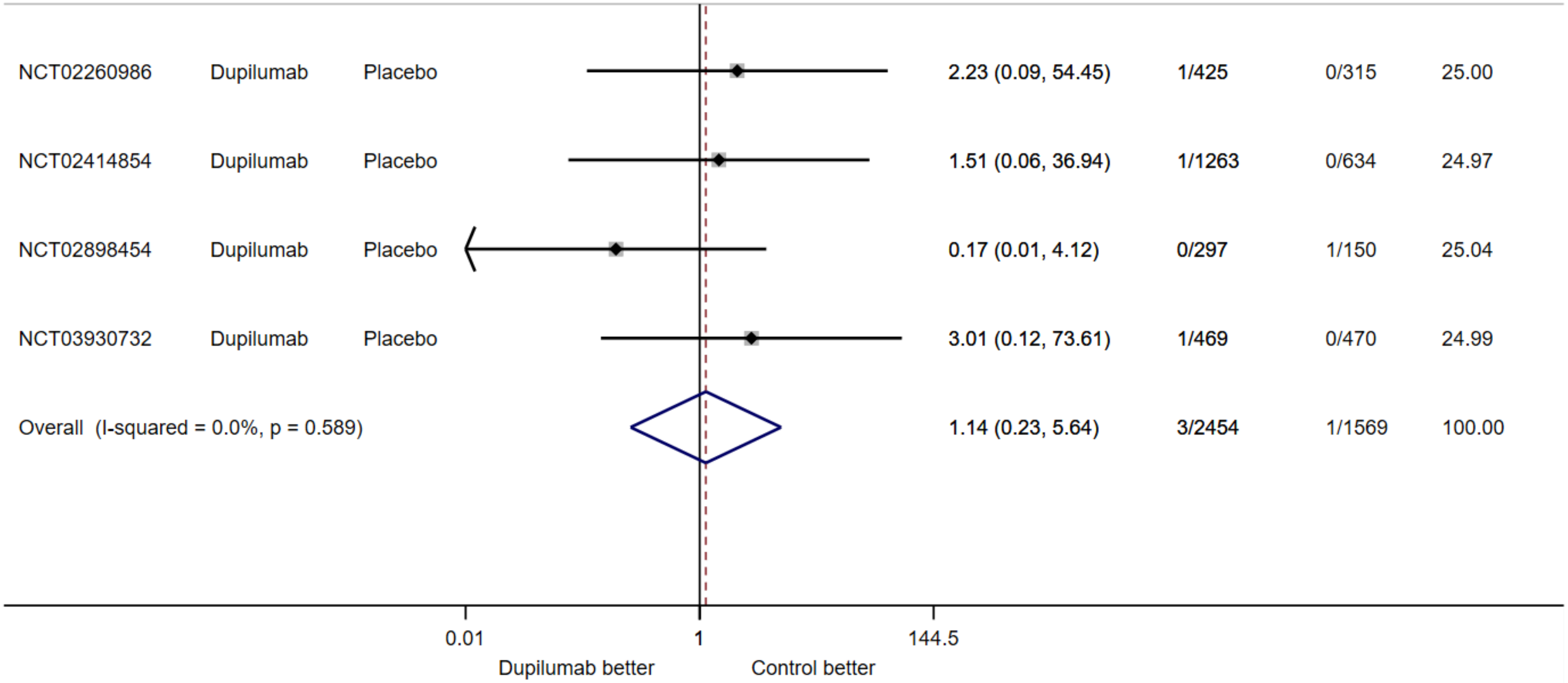

6_Hepatobiliary Disorders-Figure S3 Meta-analysis of Dupilumab and Cholelithiasis

Trial	Dupilumab	Control	Risk Ratio (95% CI)	Events, Treatment	Events, Control	% Weight
NCT01854047	Dupilumab	Placebo	0.78 (0.03, 19.04)	1/611	0/158	16.37
NCT02395133	Dupilumab	Placebo	0.73 (0.03, 17.87)	1/338	0/82	16.41
NCT02414854	Dupilumab	Placebo	2.51 (0.12, 52.24)	2/1263	0/634	18.15
NCT02948959	Dupilumab	Placebo	0.17 (0.01, 4.03)	0/271	1/134	16.38
NCT03720470	Dupilumab	Placebo/PF-04965842	0.82 (0.03, 20.00)	0/242	1/595	16.35
NCT03930732	Dupilumab	Placebo	0.33 (0.01, 8.18)	0/469	1/470	16.34
Overall (I-squared = 0.0%, p = 0.892)			0.65 (0.18, 2.37)	4/3194	3/2073	100.00

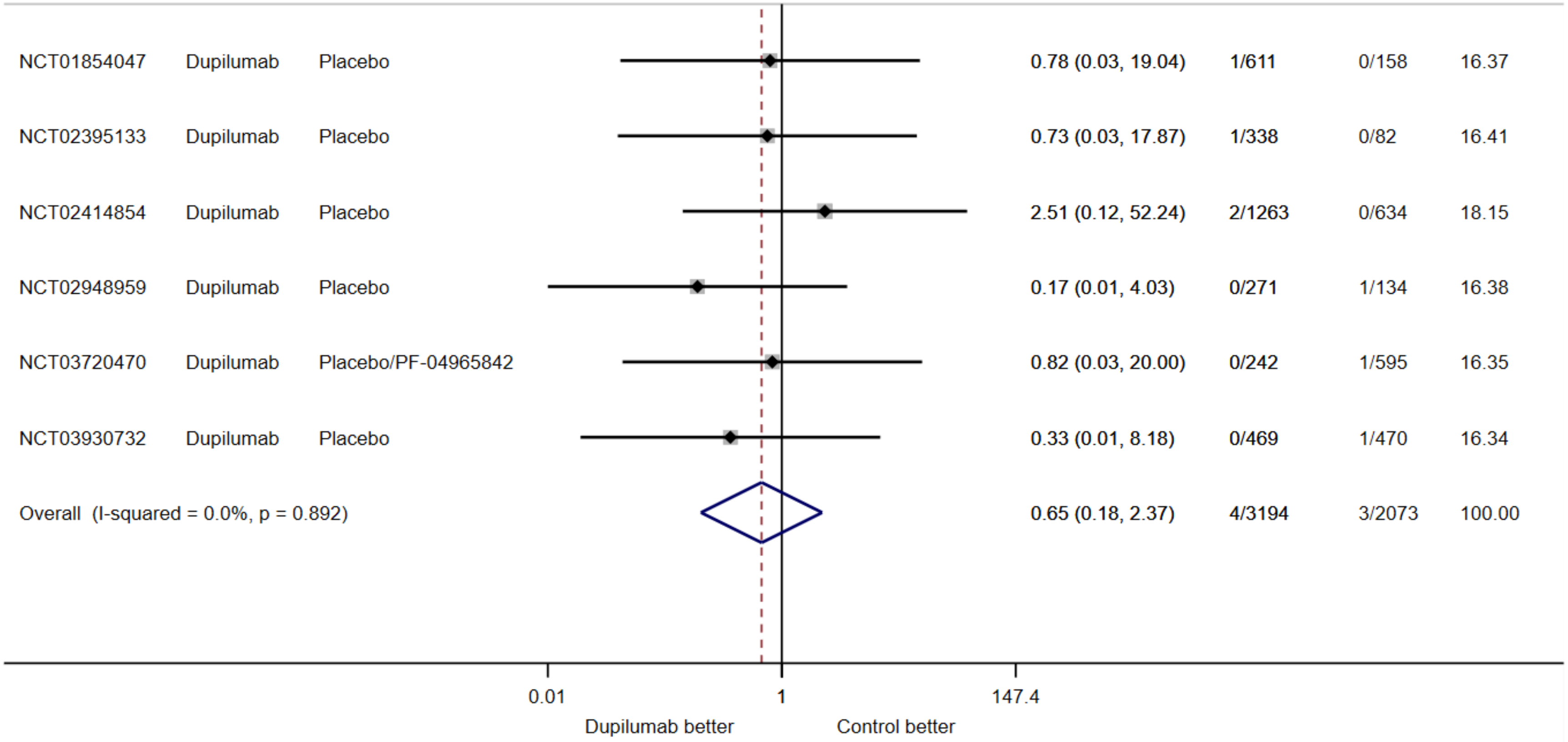

7_Immune System Disorders-Figure S1 Meta-analysis of Dupilumab and Anaphylactic reaction

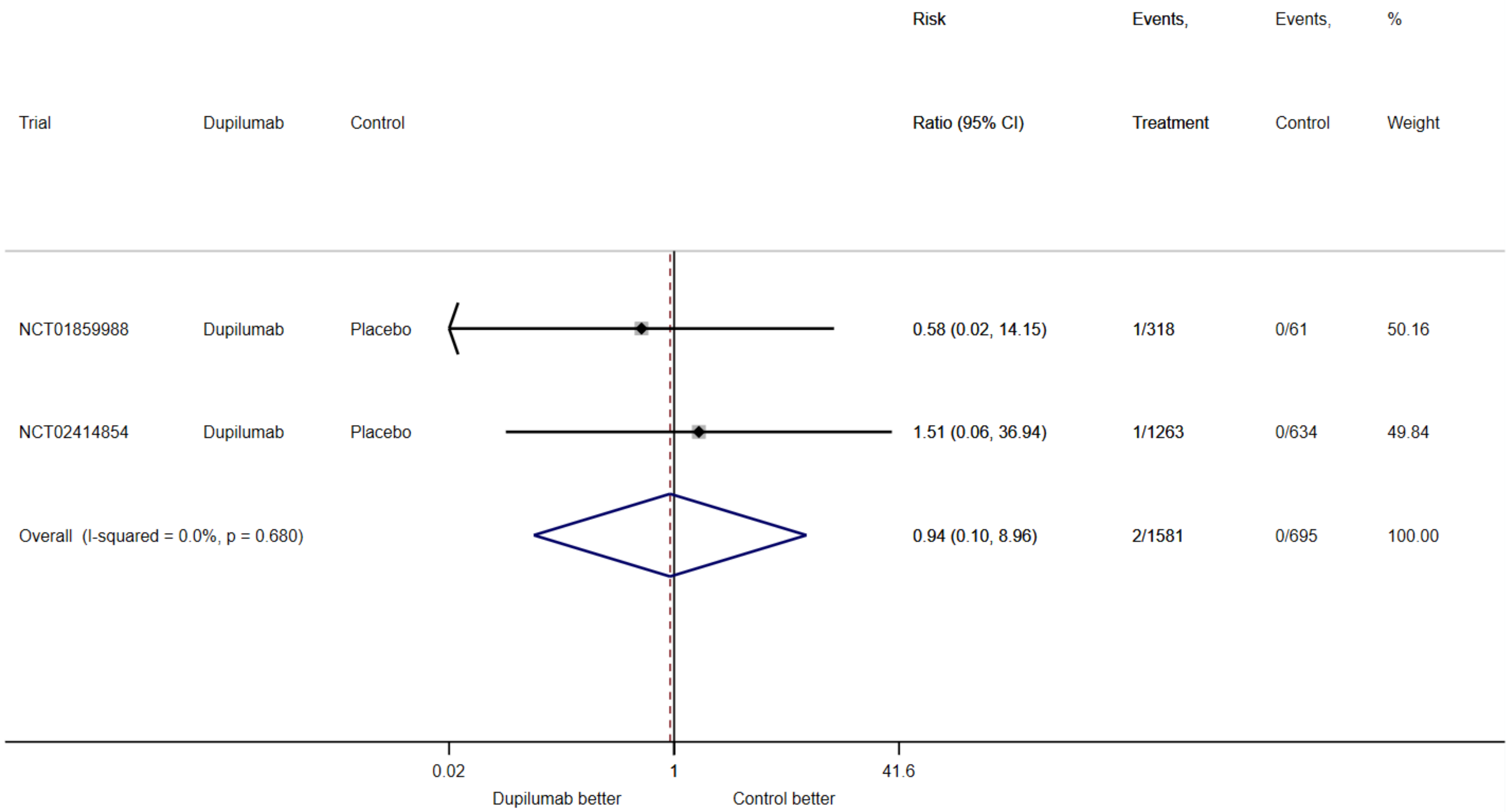

7_Immune System Disorders-Figure S2 Meta-analysis of Dupilumab and Anaphylactic shock

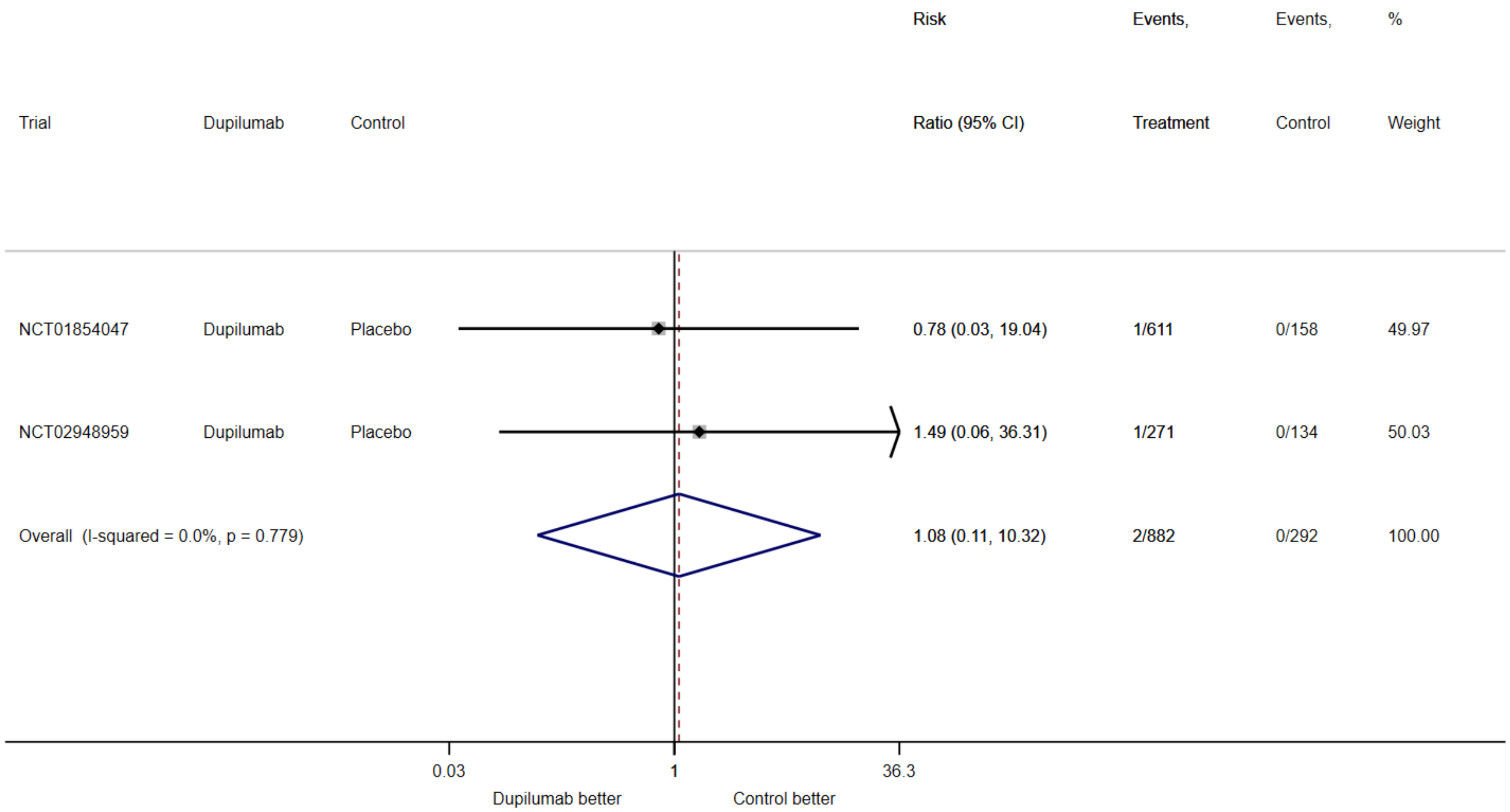

7_Immune System Disorders-Figure S3 Meta-analysis of Dupilumab and Drug hypersensitivity

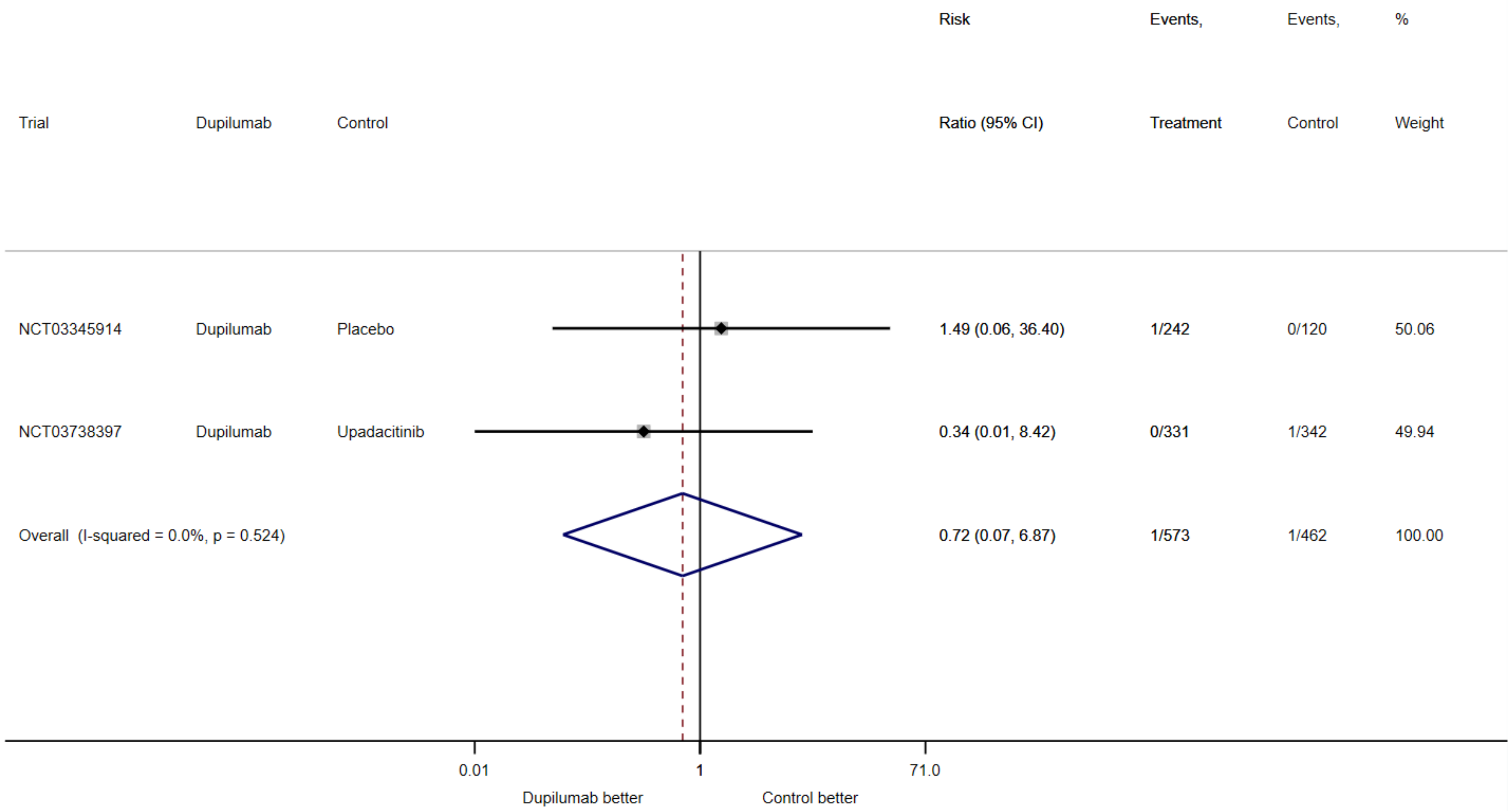

7_Immune System Disorders-Figure S4 Meta-analysis of Dupilumab and Food allergy

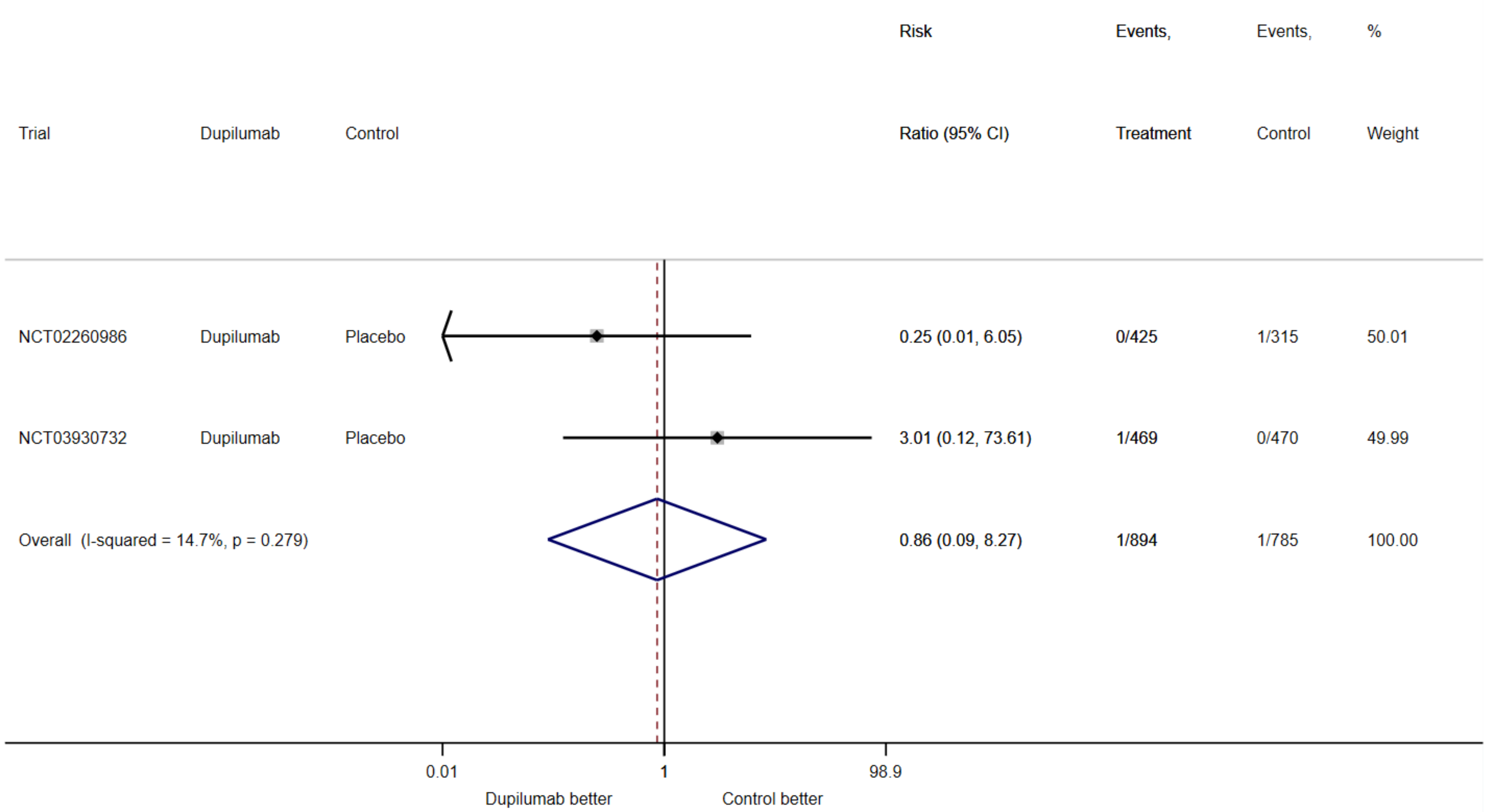

8_Infections And Infestations-Figure S1 Meta-analysis of Dupilumab and Abdominal wall abscess

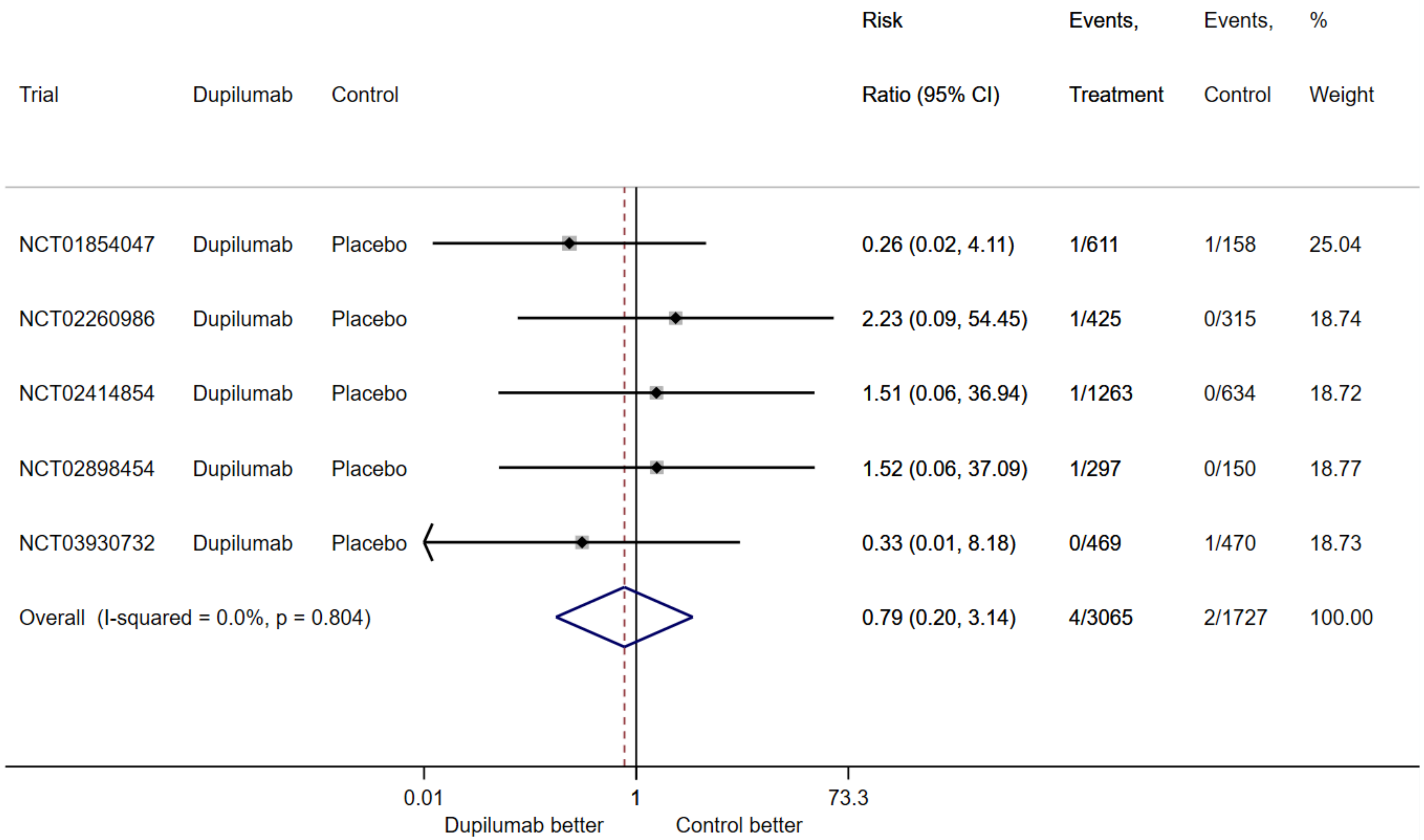

8_Infections And Infestations-Figure S2 Meta-analysis of Dupilumab and Appendicitis

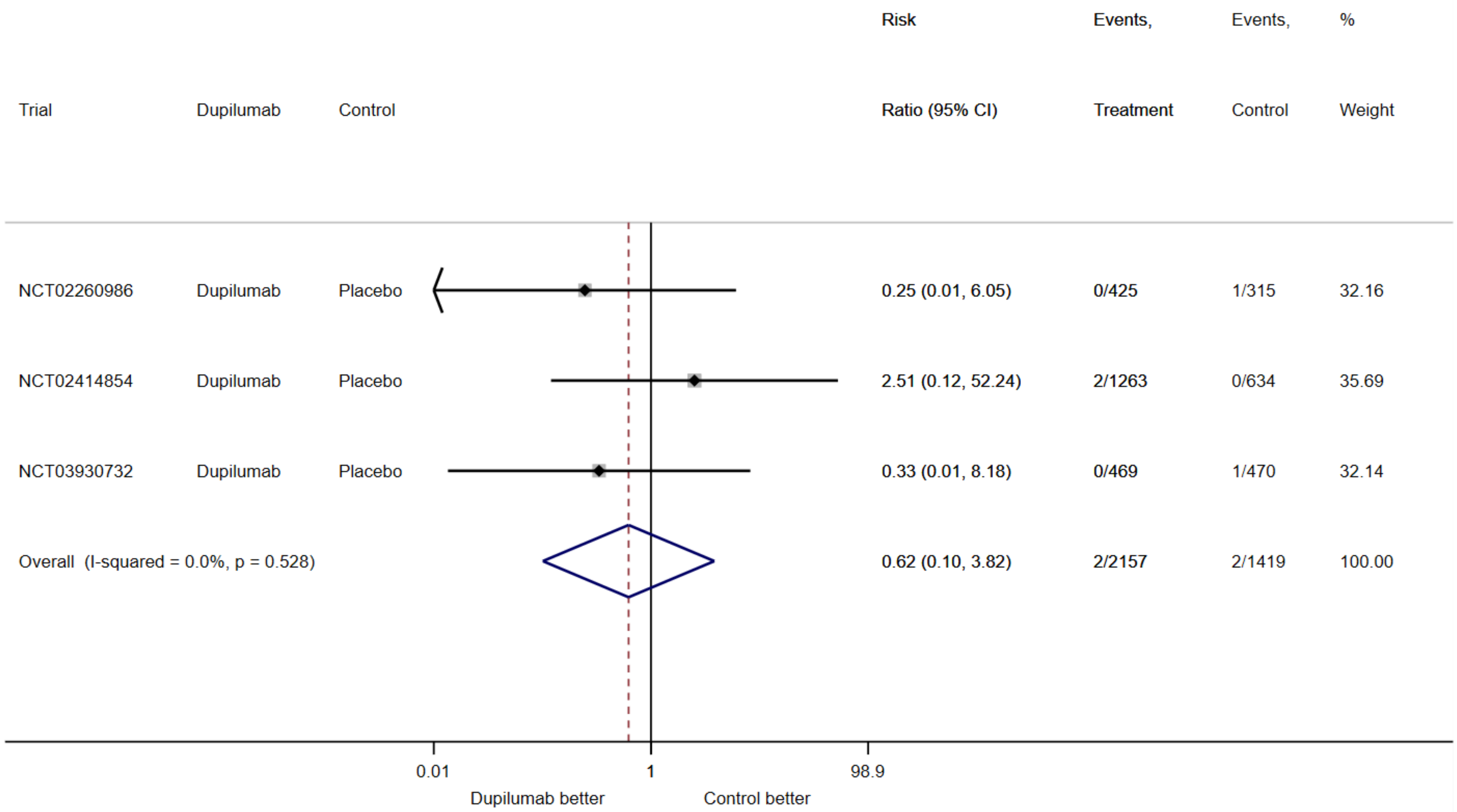

8_Infections And Infestations-Figure S3 Meta-analysis of Dupilumab and Bronchitis

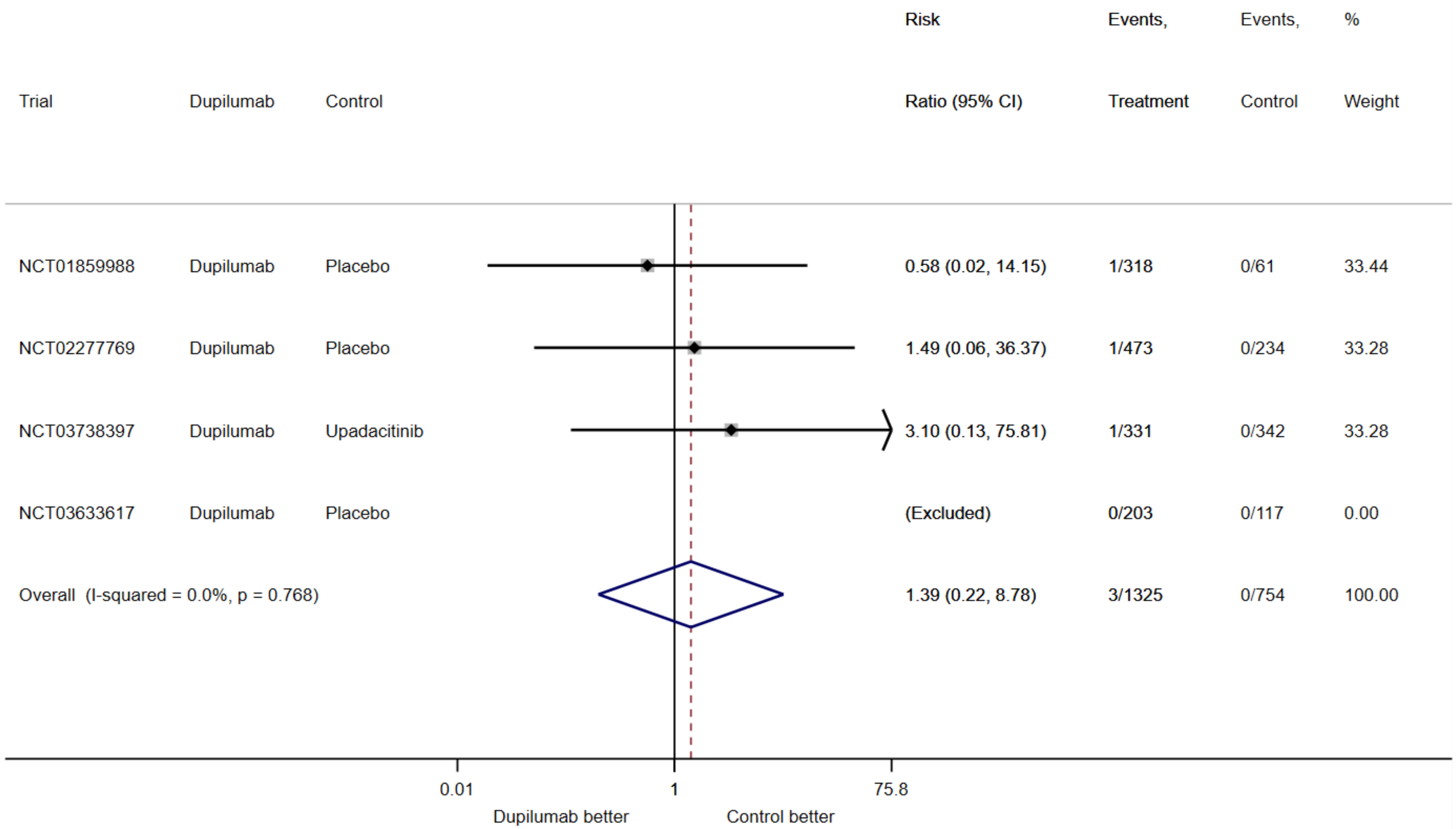

8_Infections And Infestations-Figure S4 Meta-analysis of Dupilumab and Cellulitis

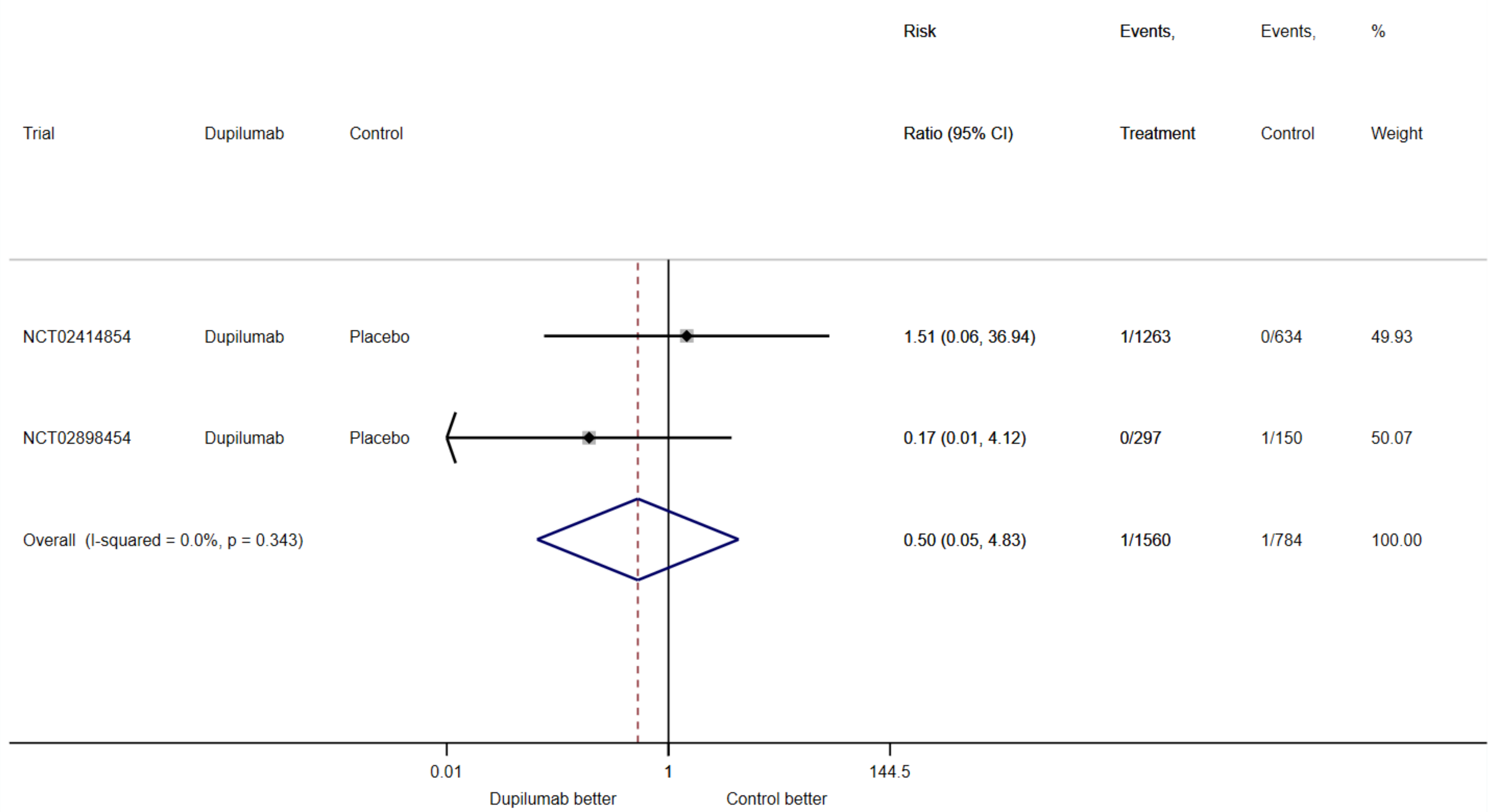

8_Infections And Infestations-Figure S5 Meta-analysis of Dupilumab and Chronic sinusitis

Trial	Dupilumab	Control	Risk Ratio (95% CI)	Events, Treatment	Events, Control	% Weight
NCT03930732	Dupilumab	Placebo	0.75 (0.17, 3.34)	3/469	4/470	82.13
NCT04345367	Dupilumab	Abrocitinib	0.33 (0.01, 8.09)	0/365	1/362	17.87
Overall (I-squared = 0.0%, p = 0.648)			0.65 (0.17, 2.51)	3/834	5/832	100.00

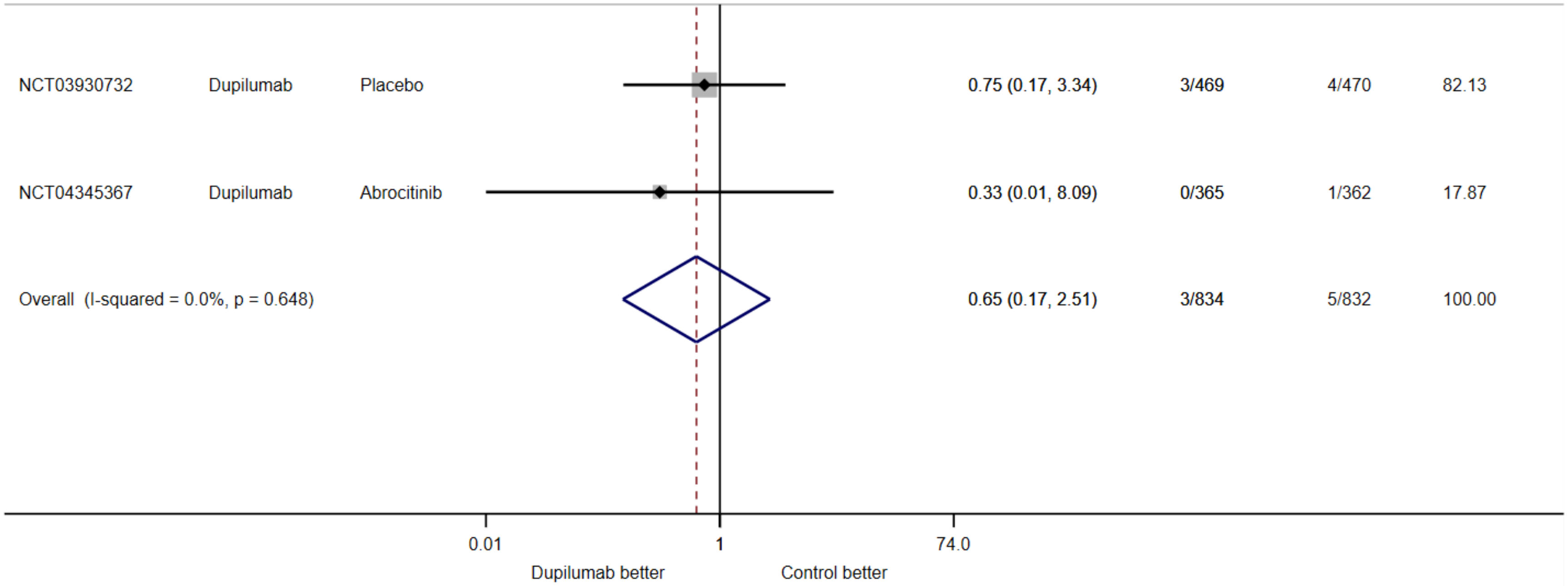

8_Infections And Infestations-Figure S6 Meta-analysis of Dupilumab and Covid-19

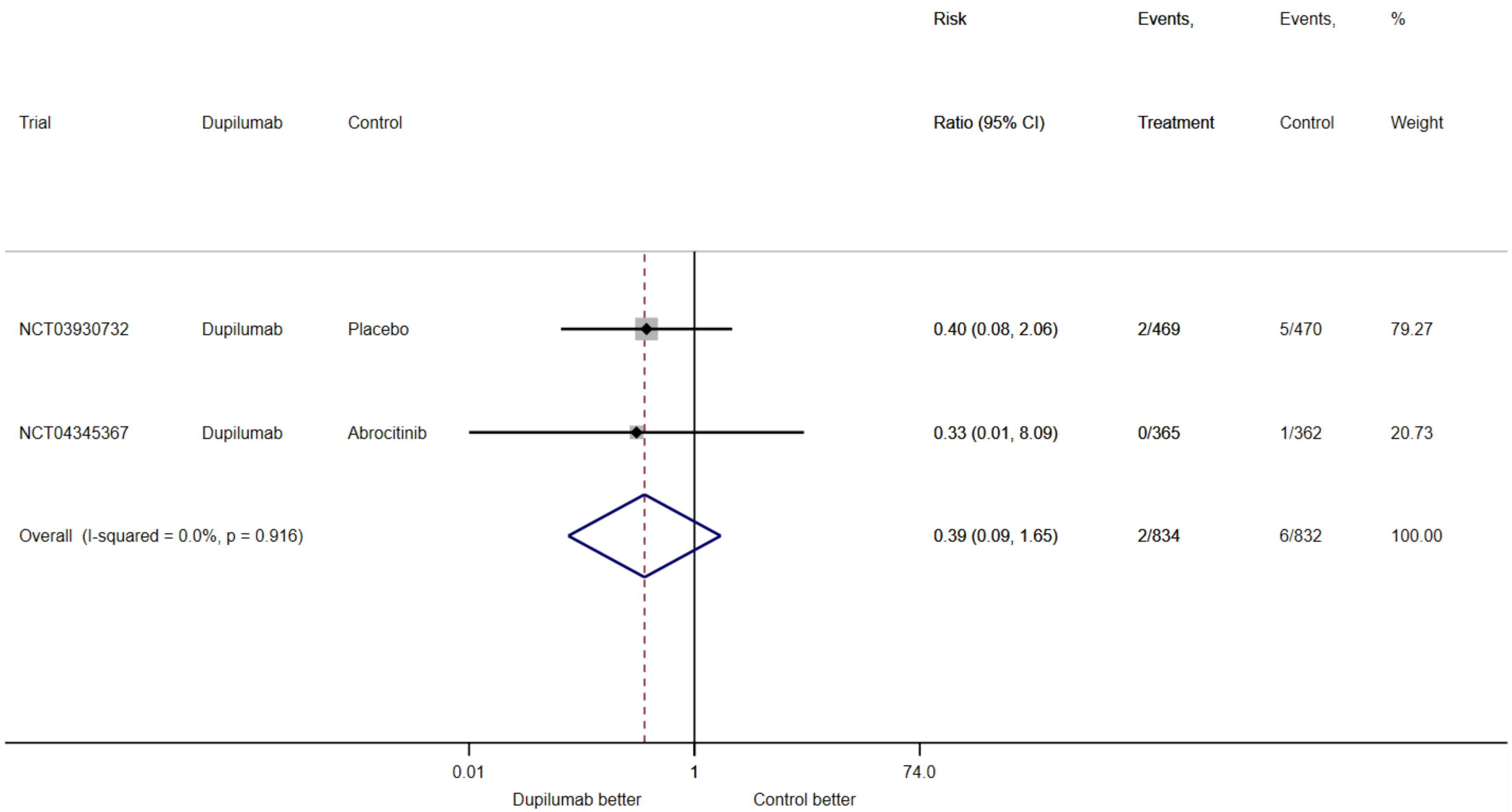

8_Infections And Infestations-Figure S7 Meta-analysis of Dupilumab and Covid-19 pneumonia

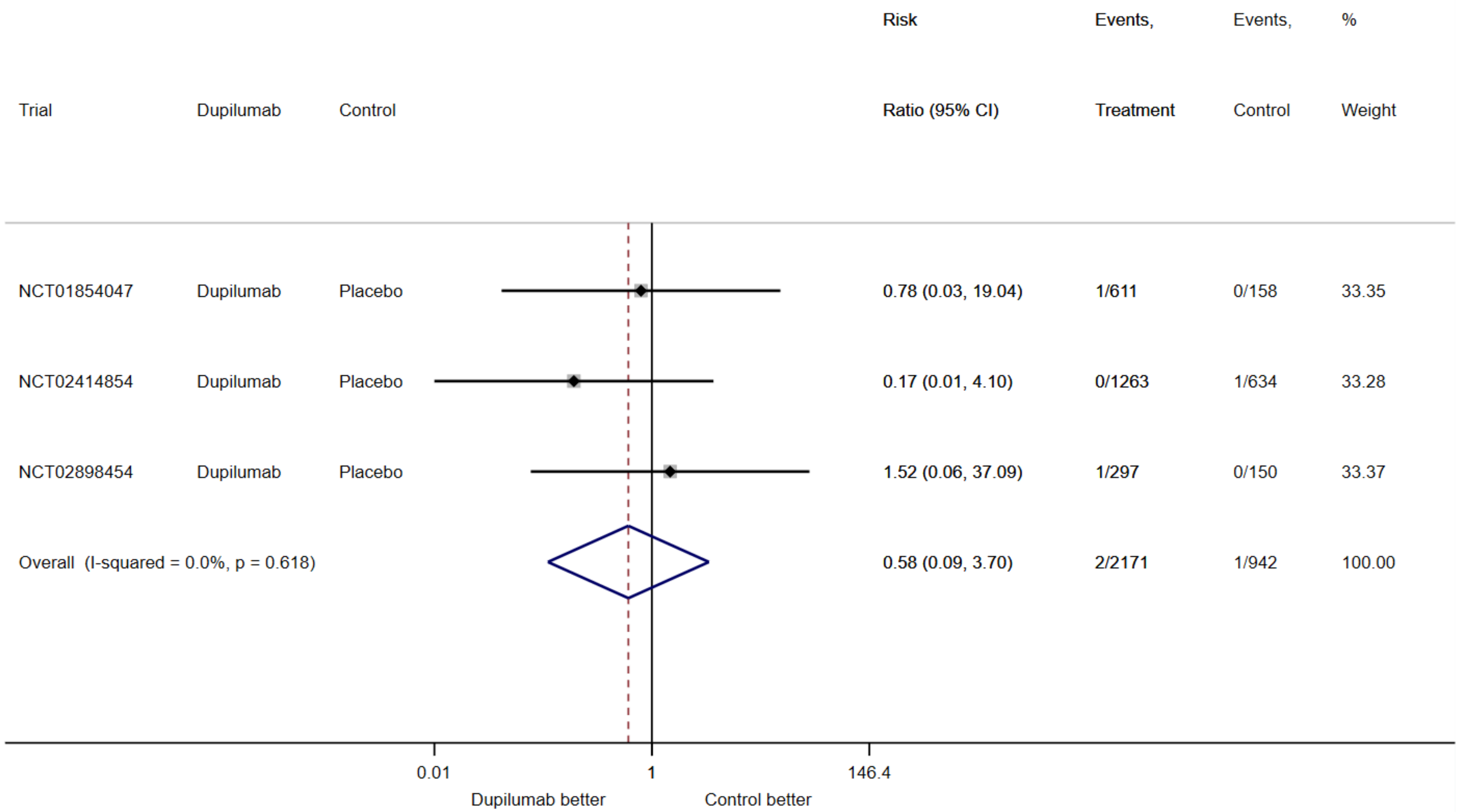

8_Infections And Infestations-Figure S8 Meta-analysis of Dupilumab and Diverticulitis

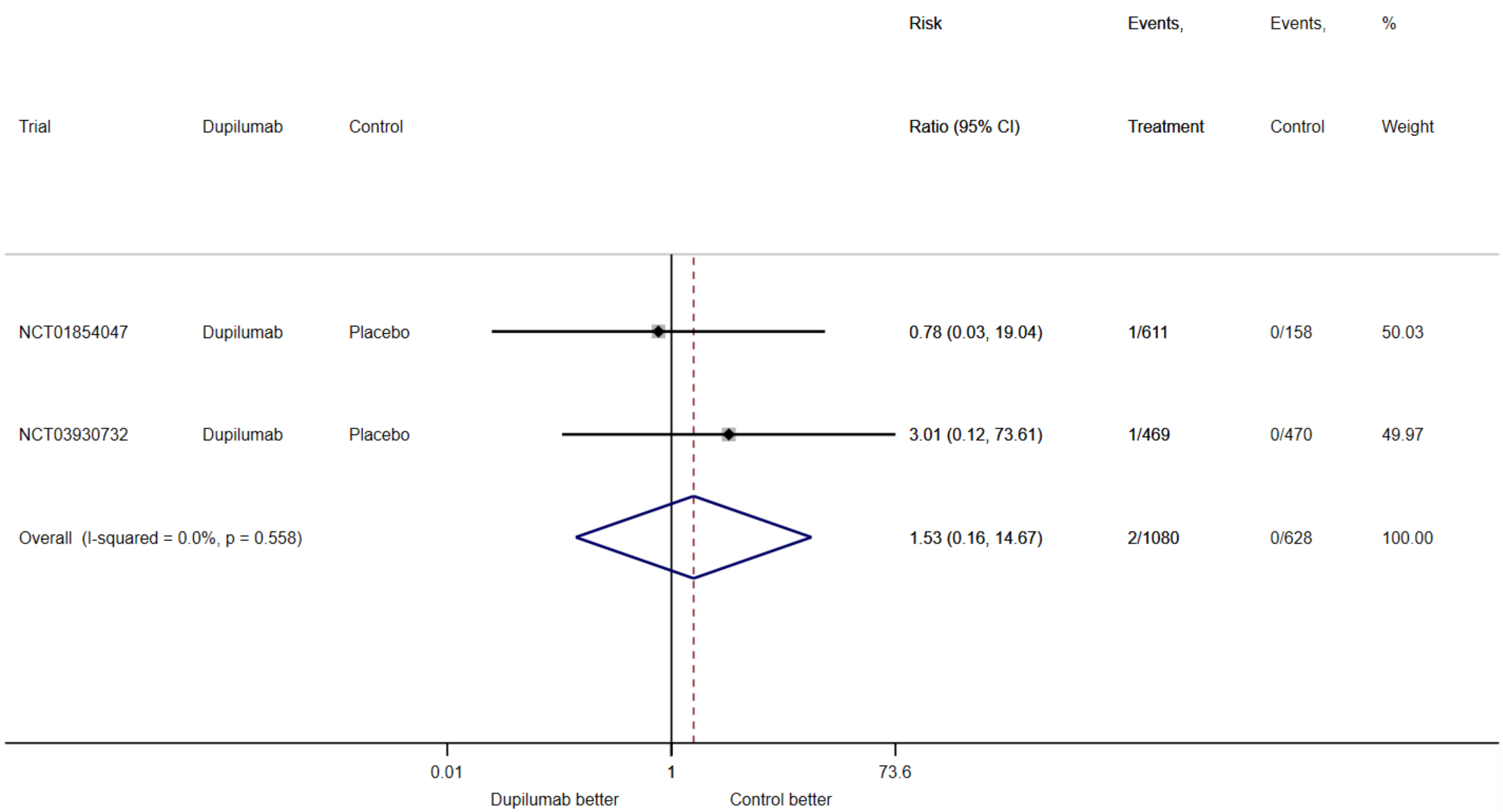

8_Infections And Infestations-Figure S9 Meta-analysis of Dupilumab and Epiglottitis

Trial	Dupilumab	Control	Risk Ratio (95% CI)	Events, Treatment	Events, Control	% Weight
NCT01854047	Dupilumab	Placebo	0.78 (0.03, 19.04)	1/611	0/158	33.35
NCT02277769	Dupilumab	Placebo	1.49 (0.06, 36.37)	1/473	0/234	33.33
NCT03738397	Dupilumab	Upadacitinib	3.10 (0.13, 75.81)	1/331	0/342	33.32
Overall (I-squared = 0.0%, p = 0.836)			1.53 (0.24, 9.70)	3/1415	0/734	100.00

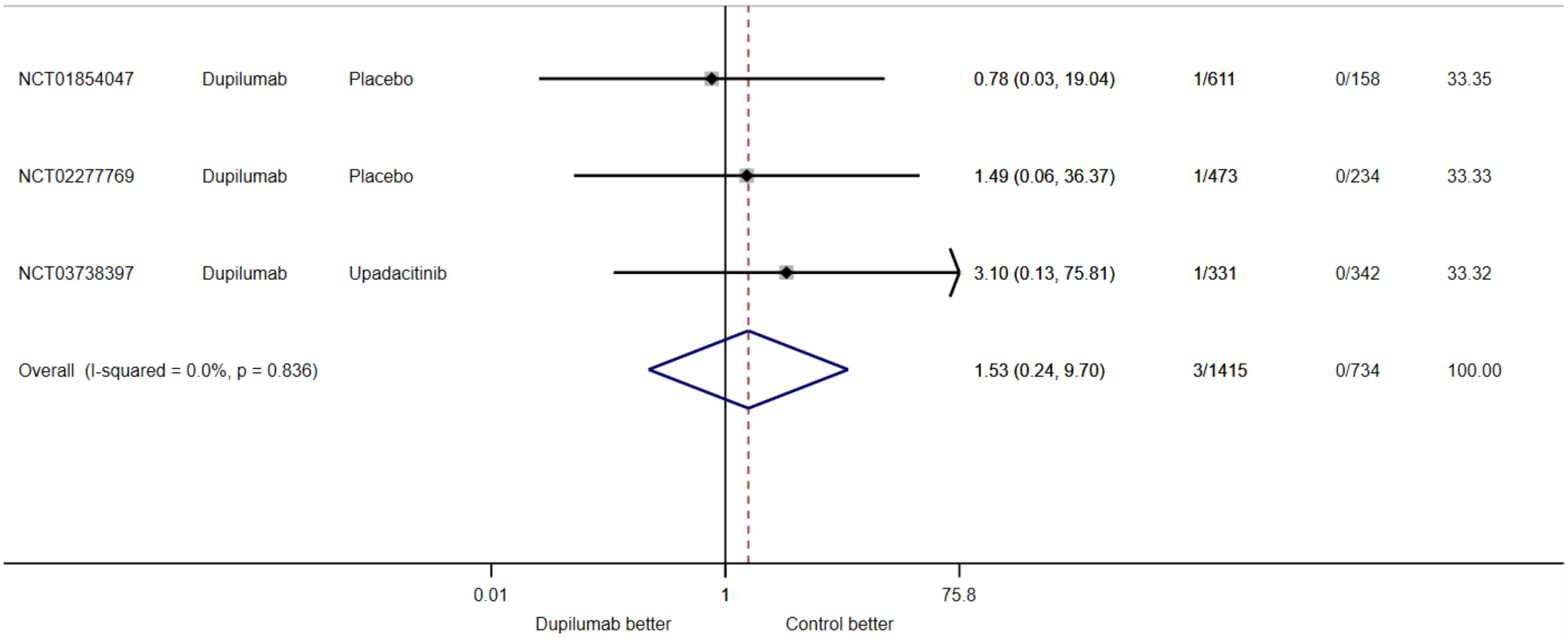

8_Infections And Infestations-Figure S10 Meta-analysis of Dupilumab and Erysipelas

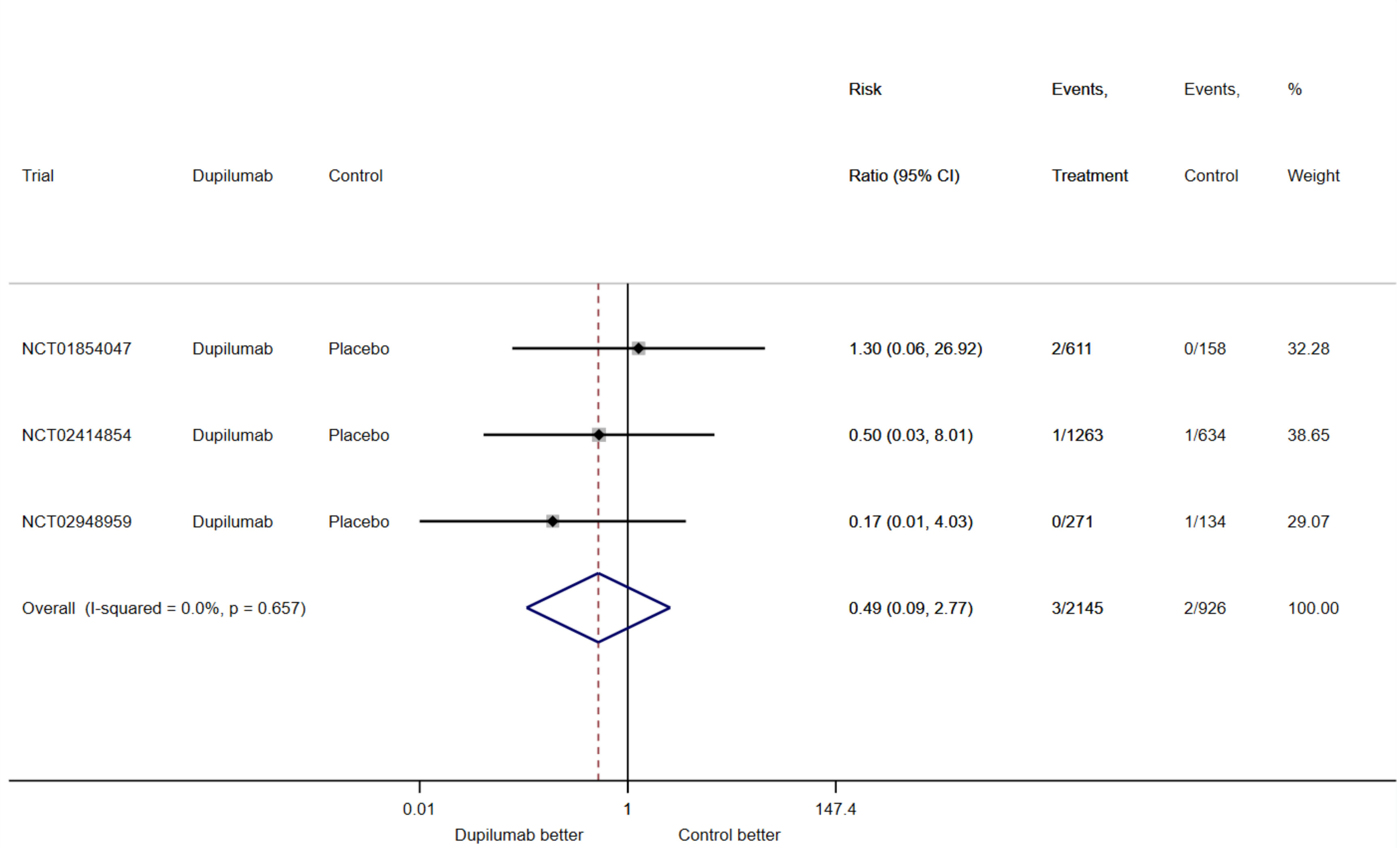

8_Infections And Infestations-Figure S11 Meta-analysis of Dupilumab and Gastroenteritis

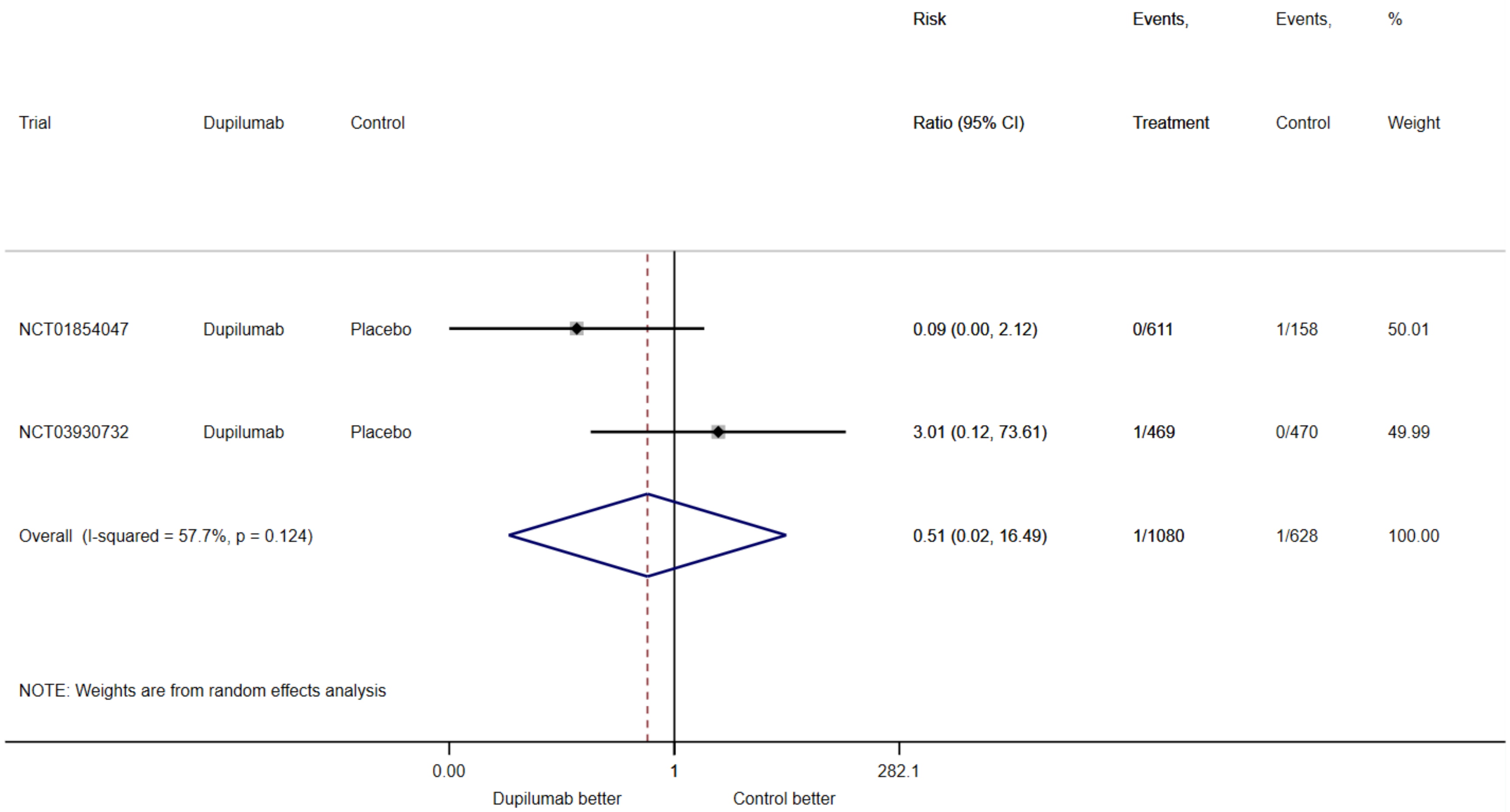

8_Infections And Infestations-Figure S12 Meta-analysis of Dupilumab and Herpes zoster

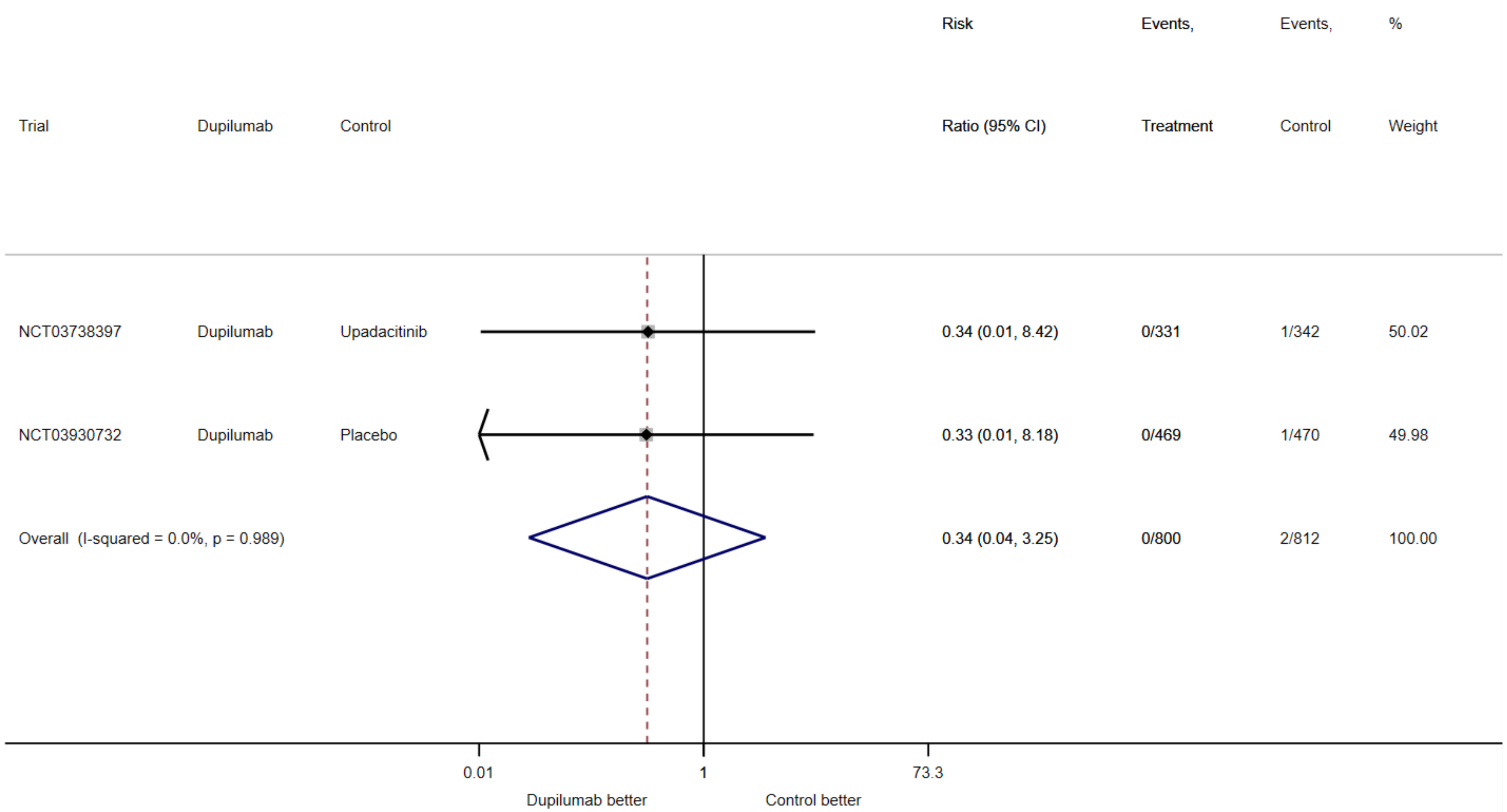

8_Infections And Infestations-Figure S13 Meta-analysis of Dupilumab and Influenza

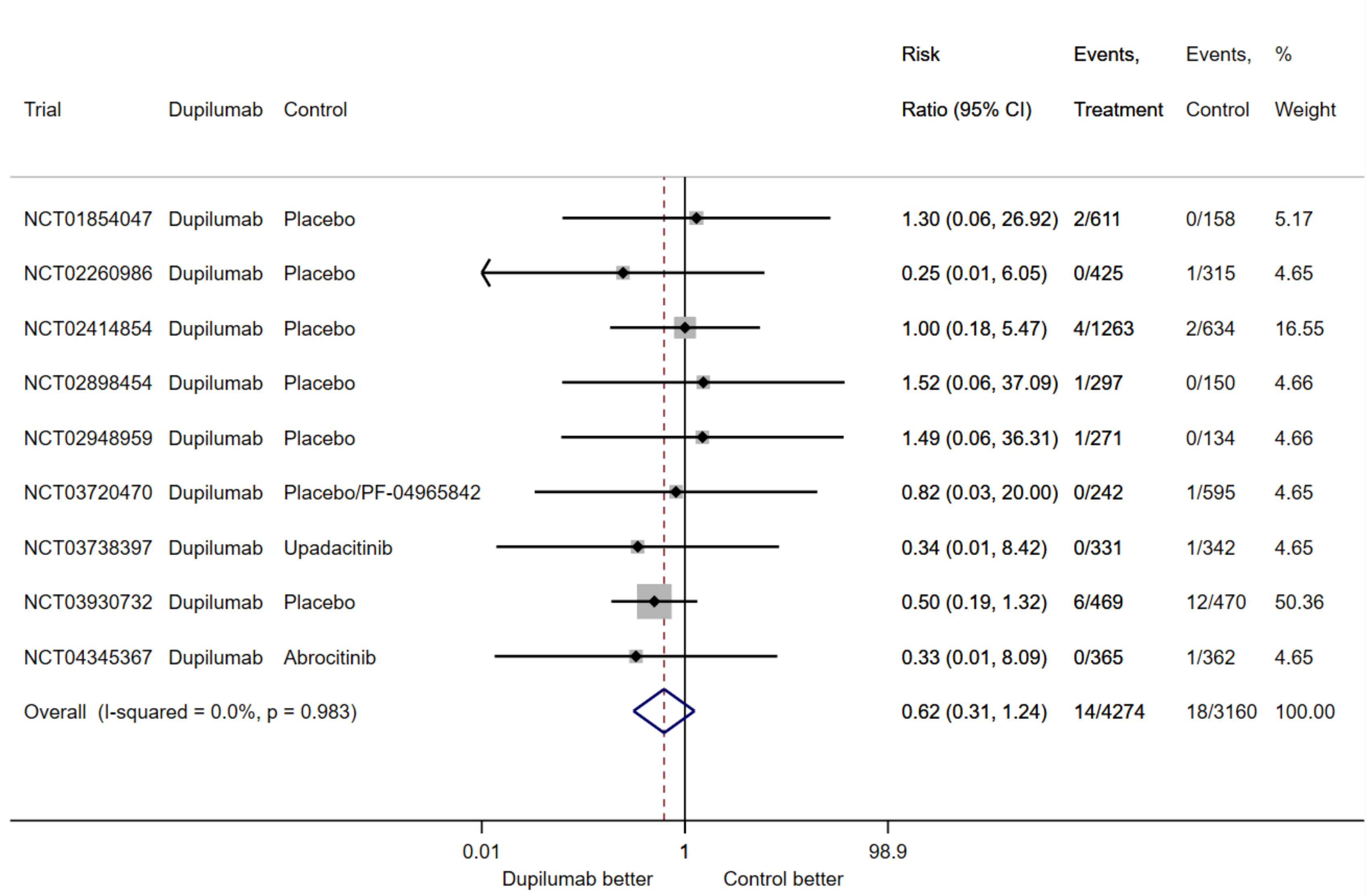

8_Infections And Infestations-Figure S14 Meta-analysis of Dupilumab and Pneumonia

Trial	Dupilumab	Control	Risk Ratio (95% CI)	Events, Treatment	Events, Control	% Weight
NCT01854047	Dupilumab	Placebo	0.78 (0.03, 19.04)	1/611	0/158	50.03
NCT03930732	Dupilumab	Placebo	0.33 (0.01, 8.18)	0/469	1/470	49.97
Overall (I-squared = 0.0%, p = 0.713)			0.51 (0.05, 4.89)	1/1080	1/628	100.00

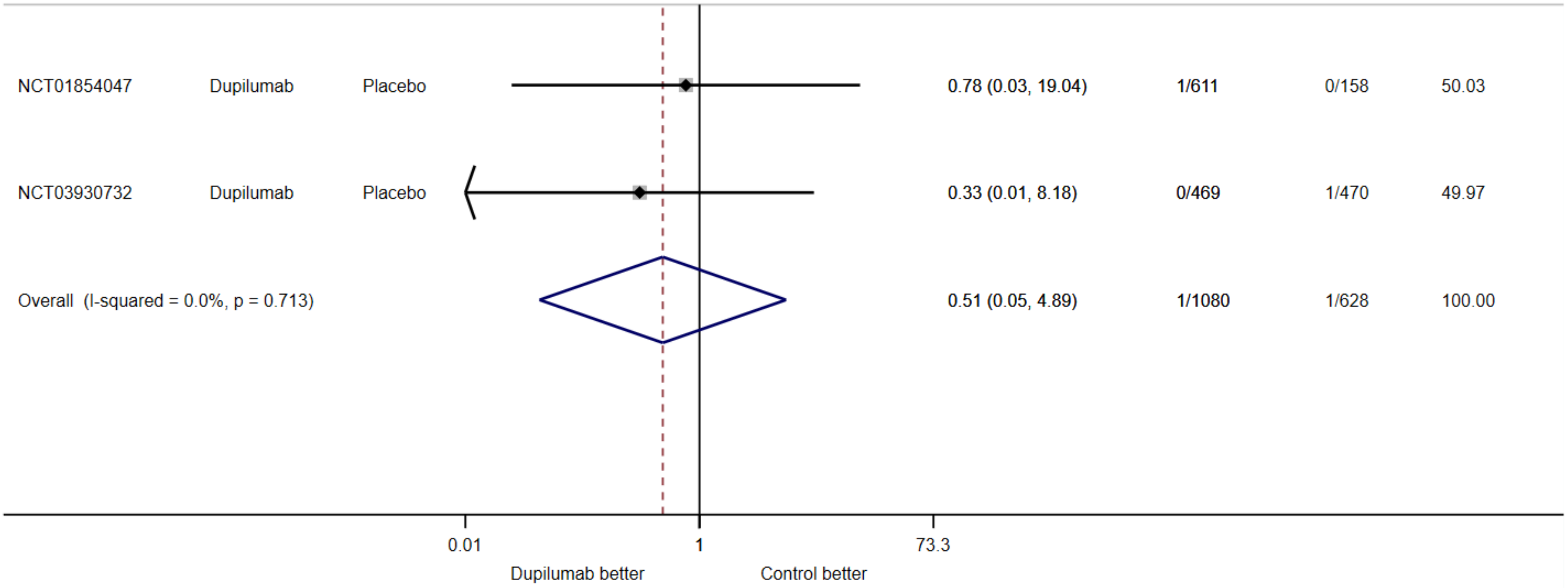

8_Infections And Infestations-Figure S15 Meta-analysis of Dupilumab and Pneumonia bacterial

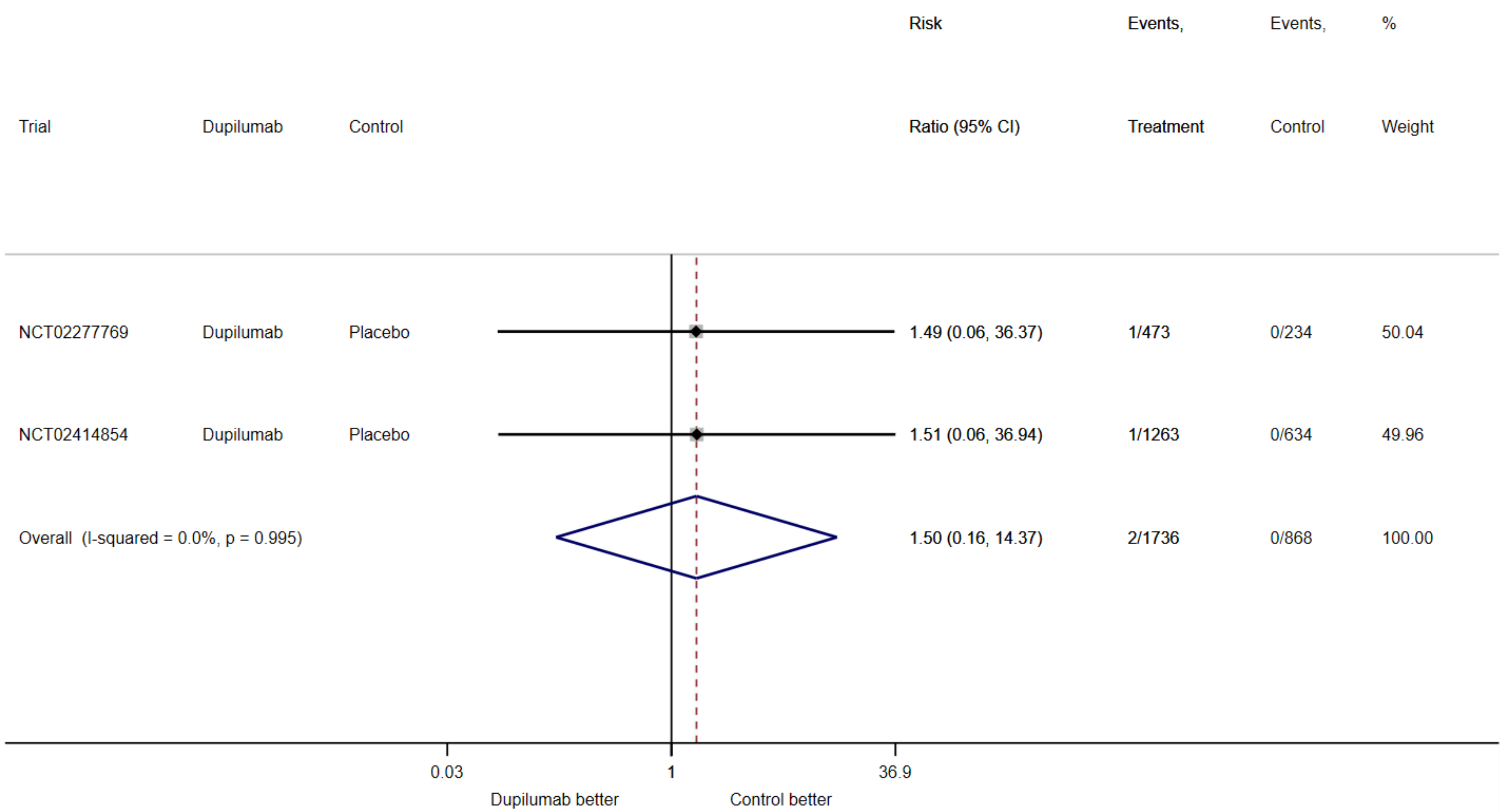

8_Infections And Infestations-Figure S16 Meta-analysis of Dupilumab and Pyelonephritis

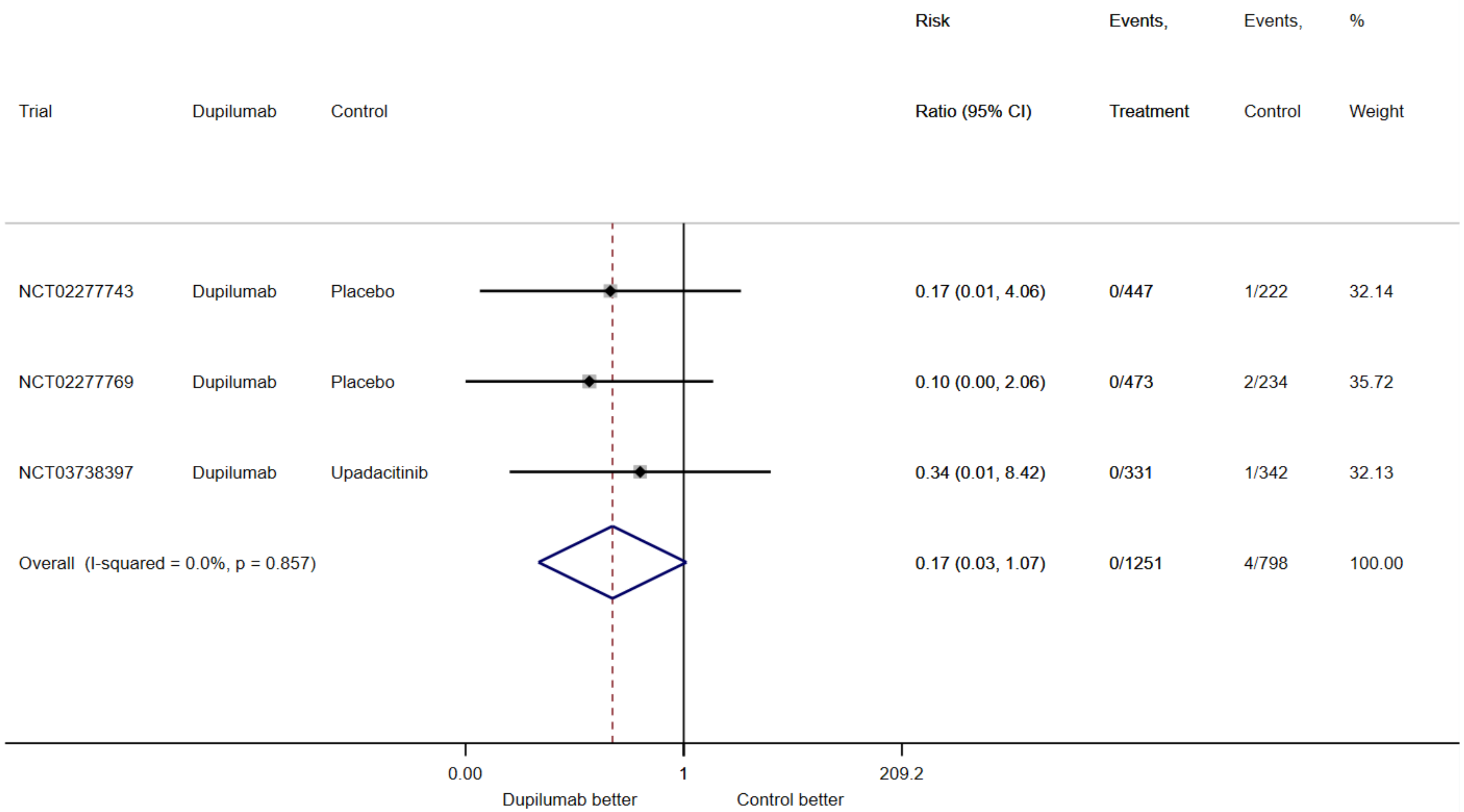

8_Infections And Infestations-Figure S17 Meta-analysis of Dupilumab and Sepsis

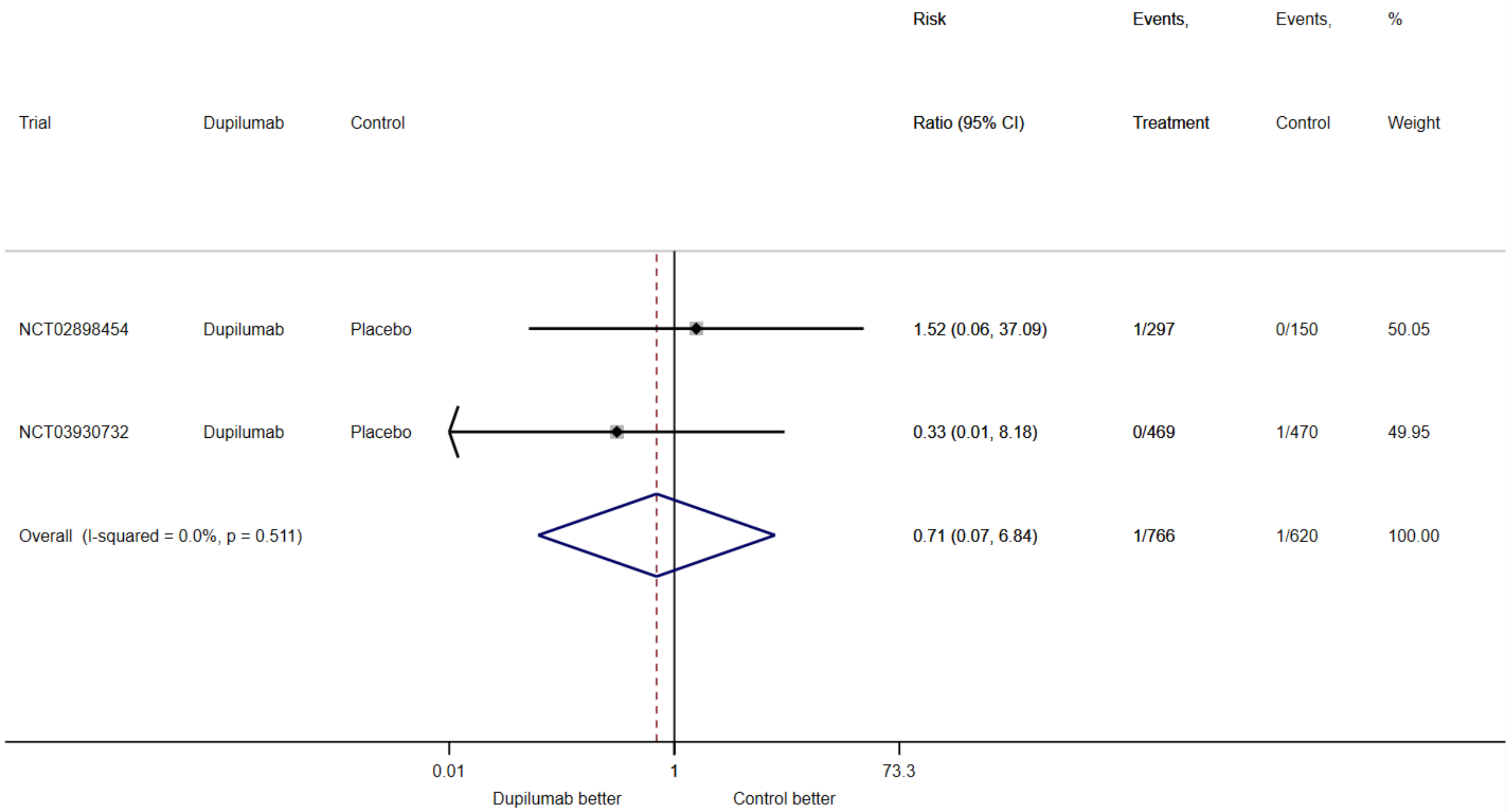

8_Infections And Infestations-Figure S18 Meta-analysis of Dupilumab and Septic shock

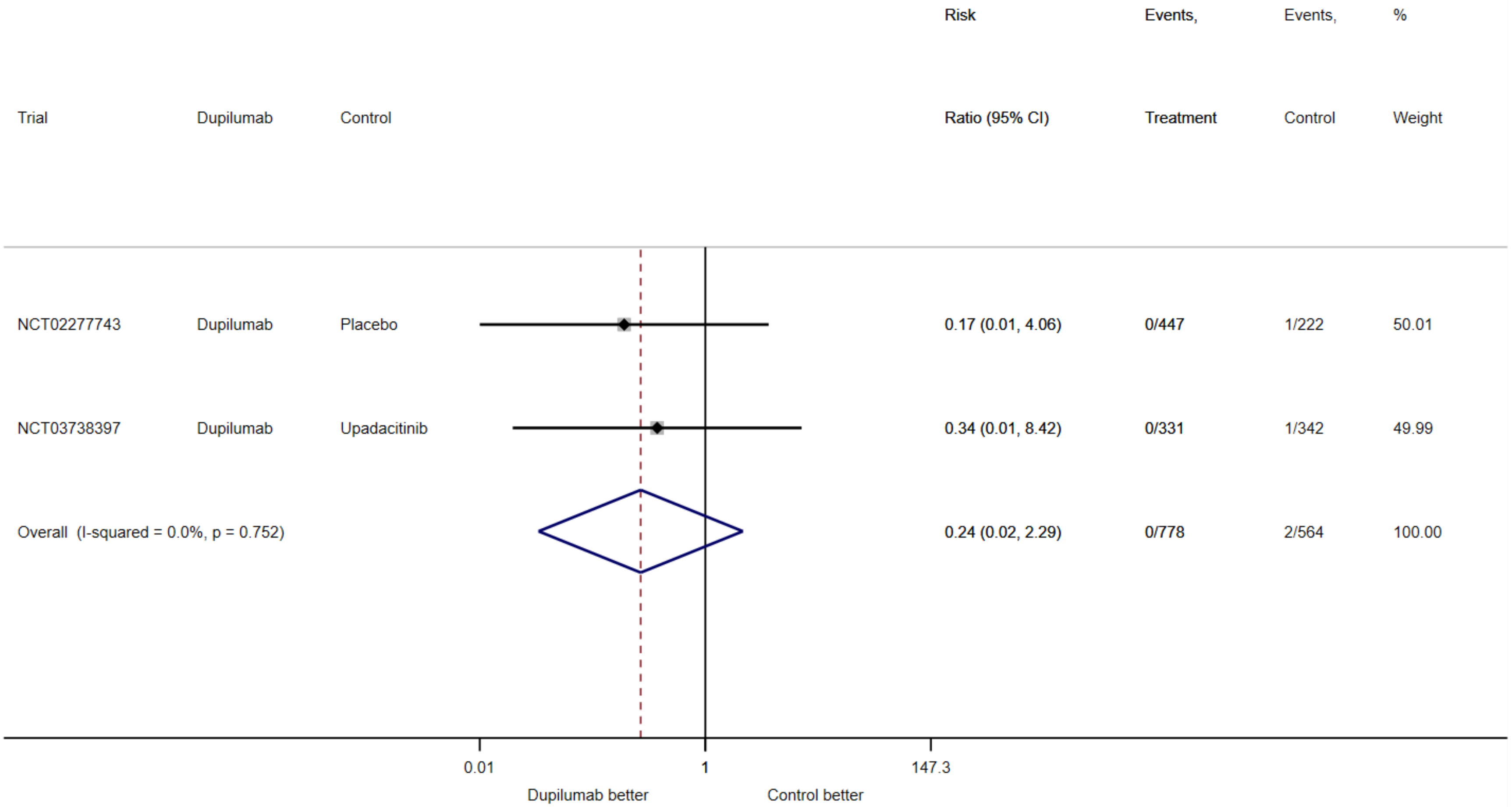

8_Infections And Infestations-Figure S19 Meta-analysis of Dupilumab and Staphylococcal infection

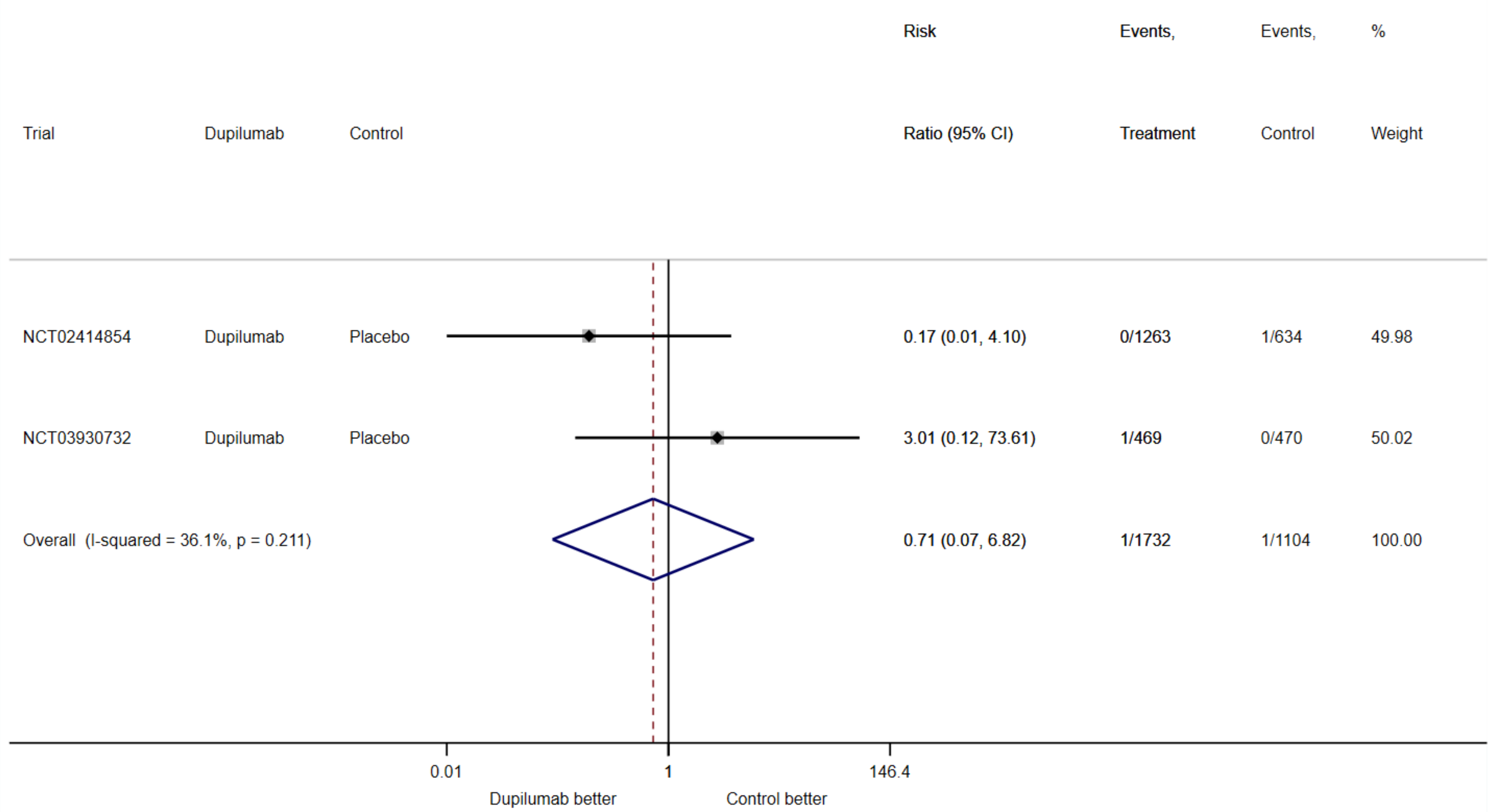

8_Infections And Infestations-Figure S20 Meta-analysis of Dupilumab and Upper respiratory tract infection

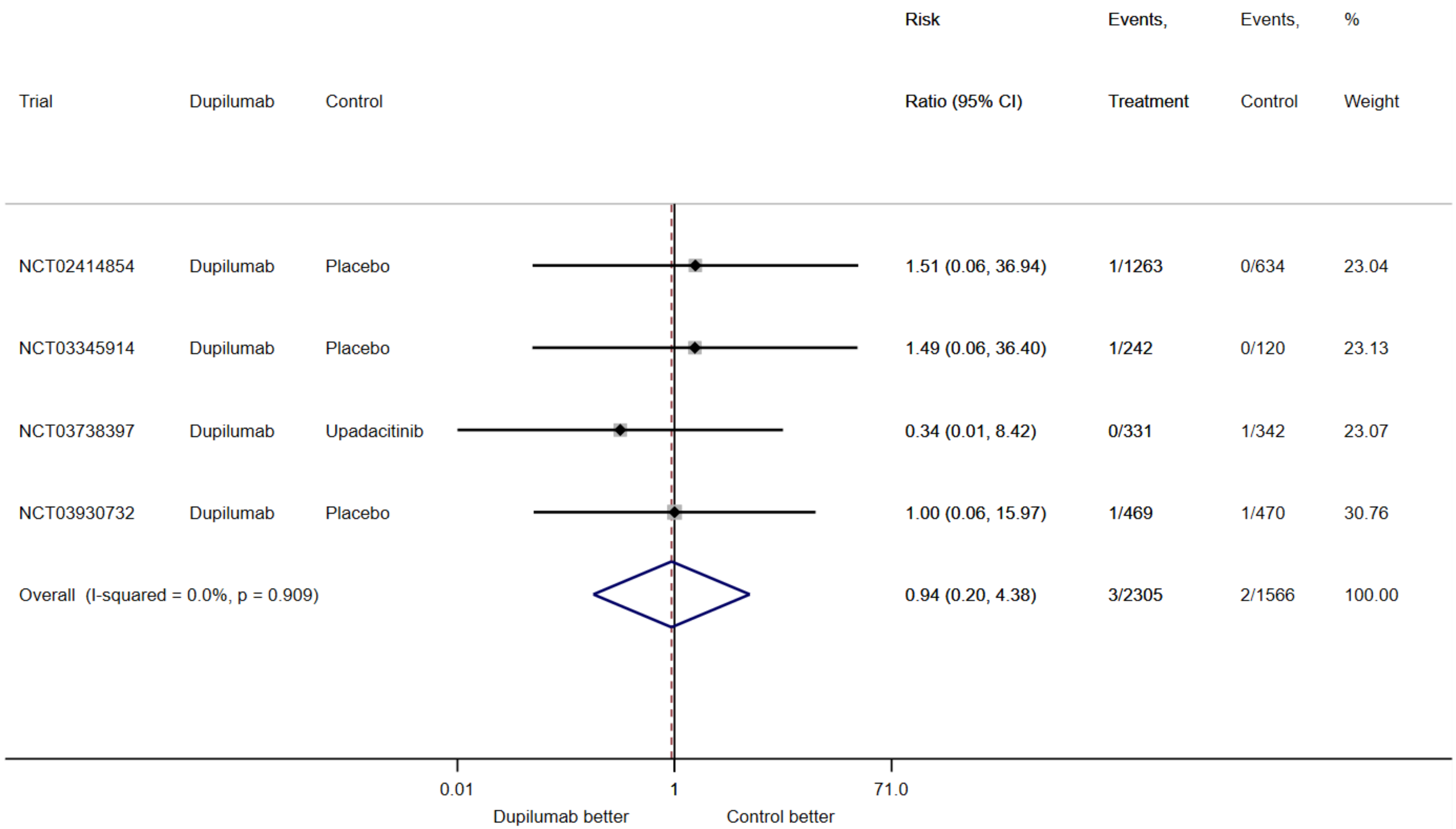

8_Infections And Infestations-Figure S21 Meta-analysis of Dupilumab and Urinary tract infection

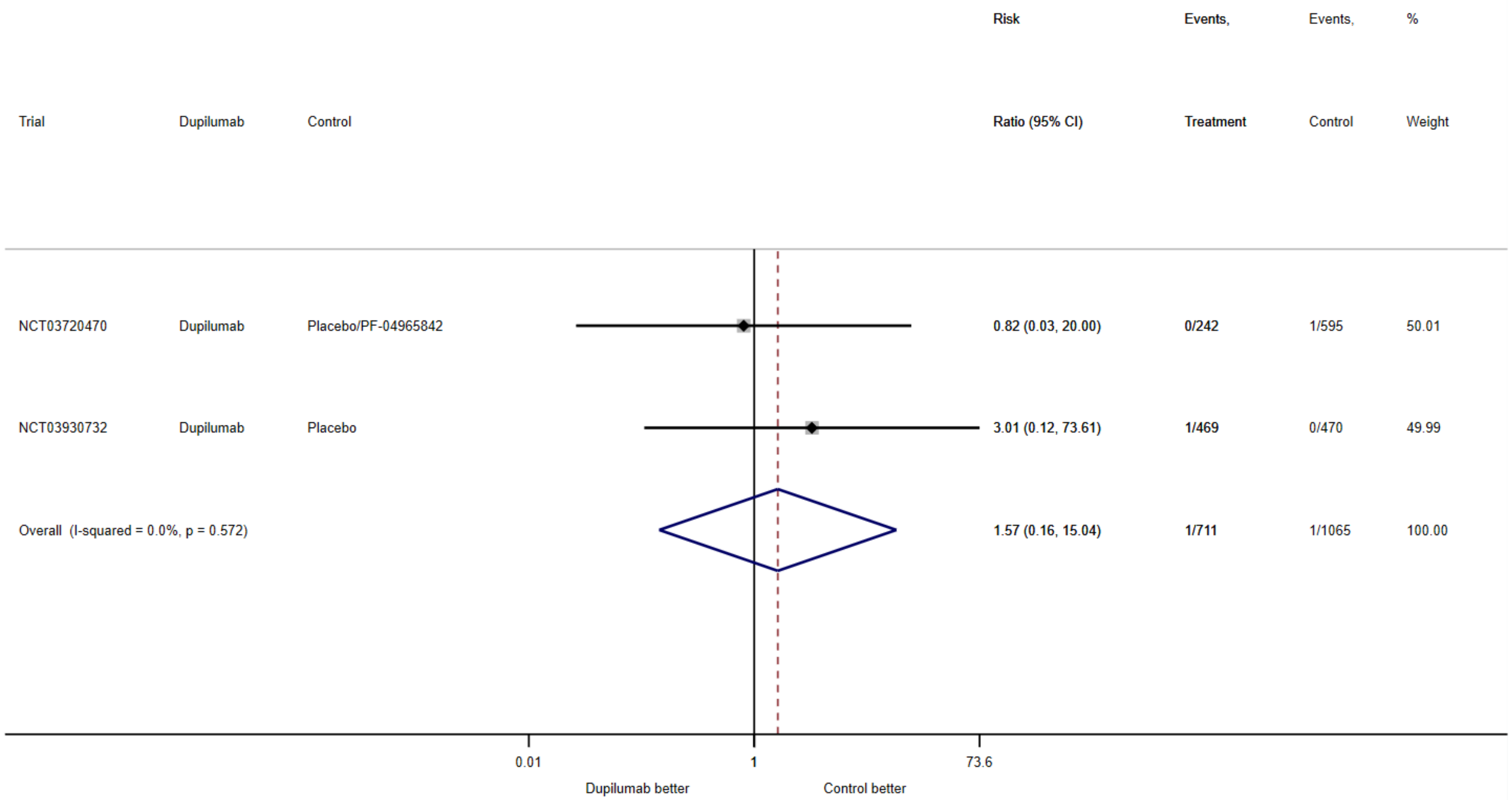

9_Injury, Poisoning And Procedural Complications-Figure S1 Meta-analysis of Dupilumab and Ankle fracture

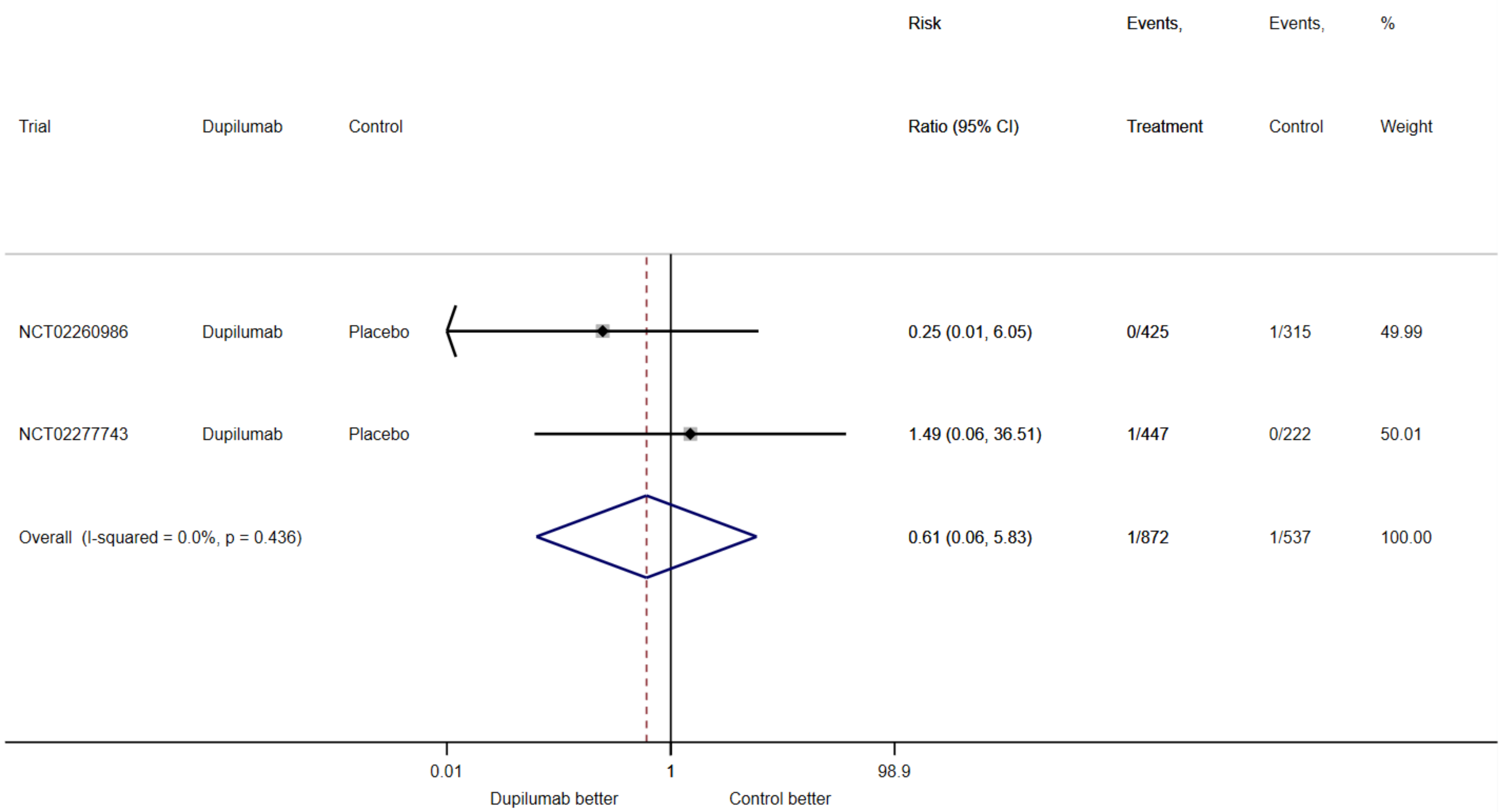

9_Injury, Poisoning And Procedural Complications-Figure S2 Meta-analysis of Dupilumab and Clavicle fracture

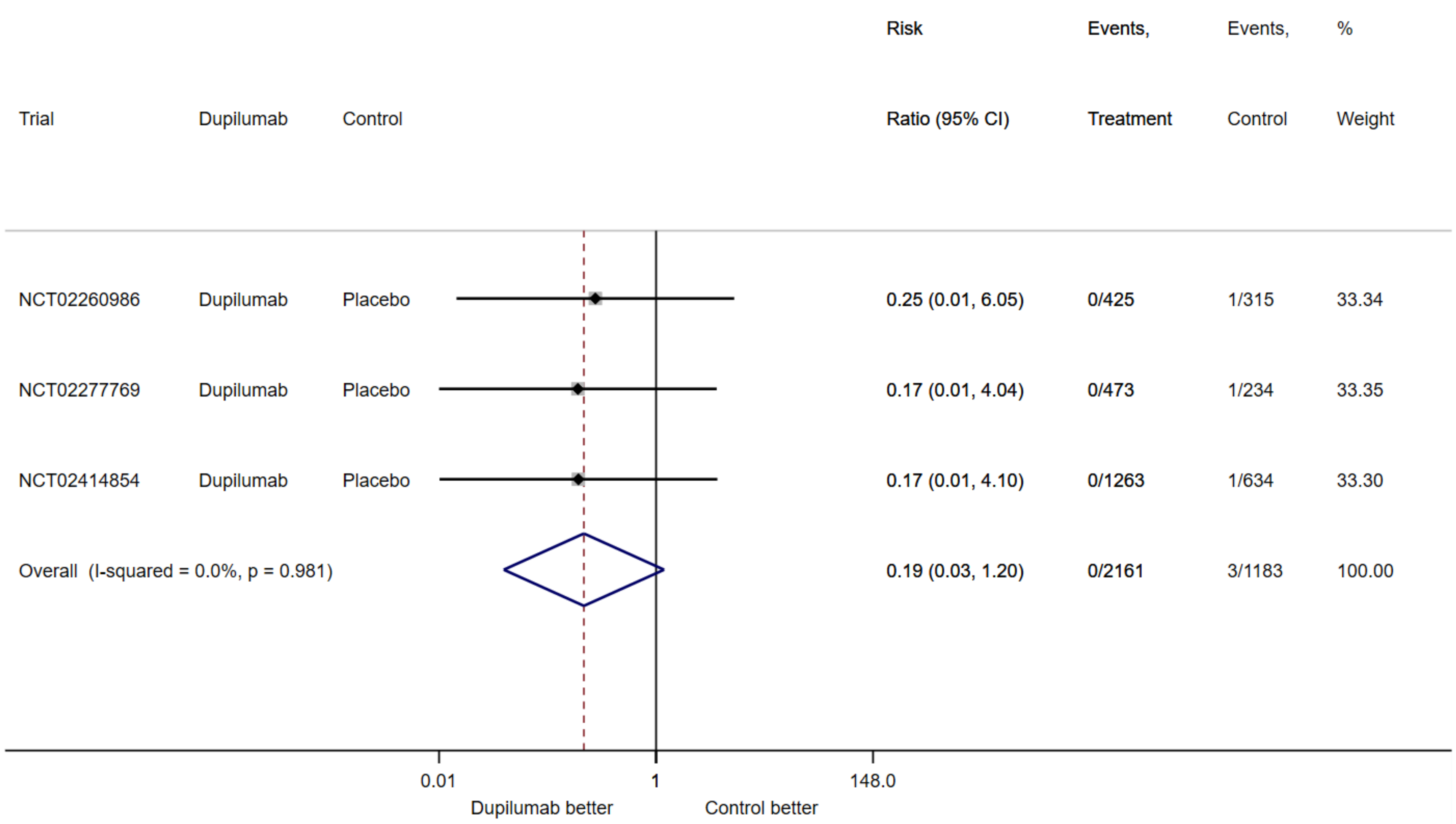

9_Injury, Poisoning And Procedural Complications-Figure S3 Meta-analysis of Dupilumab and Concussion

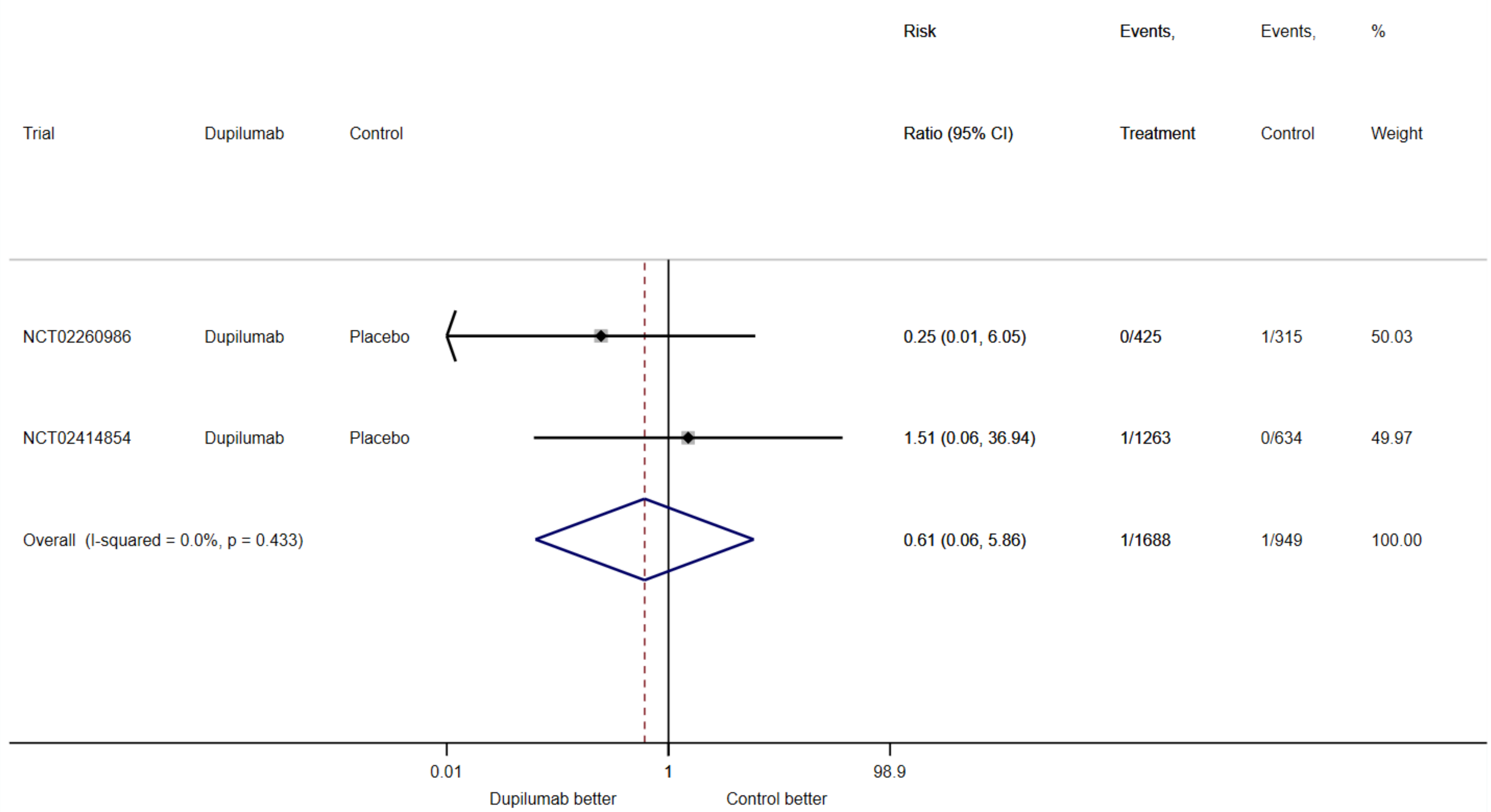

9_Injury, Poisoning And Procedural Complications-Figure S4 Meta-analysis of Dupilumab and Contusion

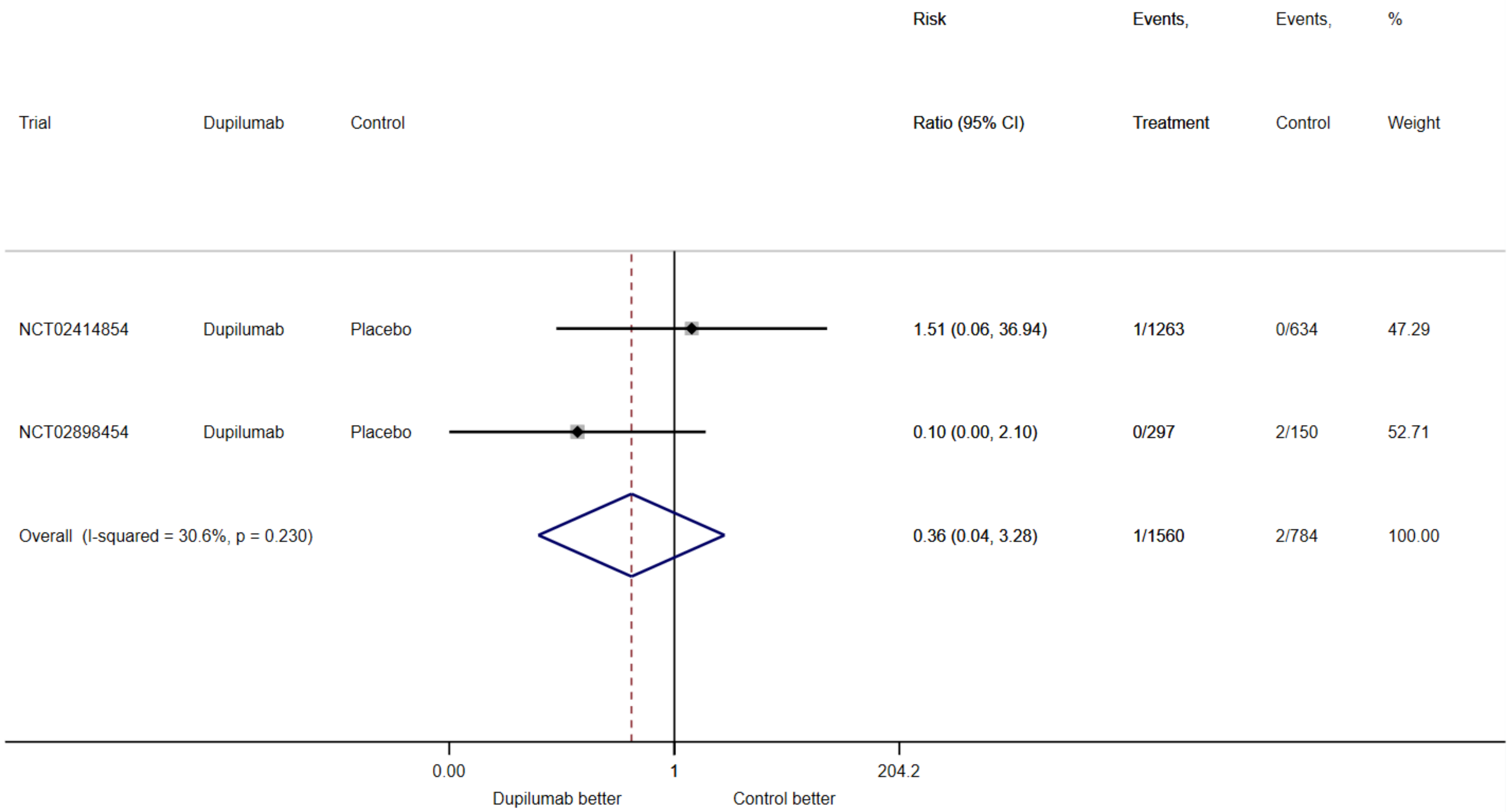

9_Injury, Poisoning And Procedural Complications-Figure S5 Meta-analysis of Dupilumab and Facial bones fracture

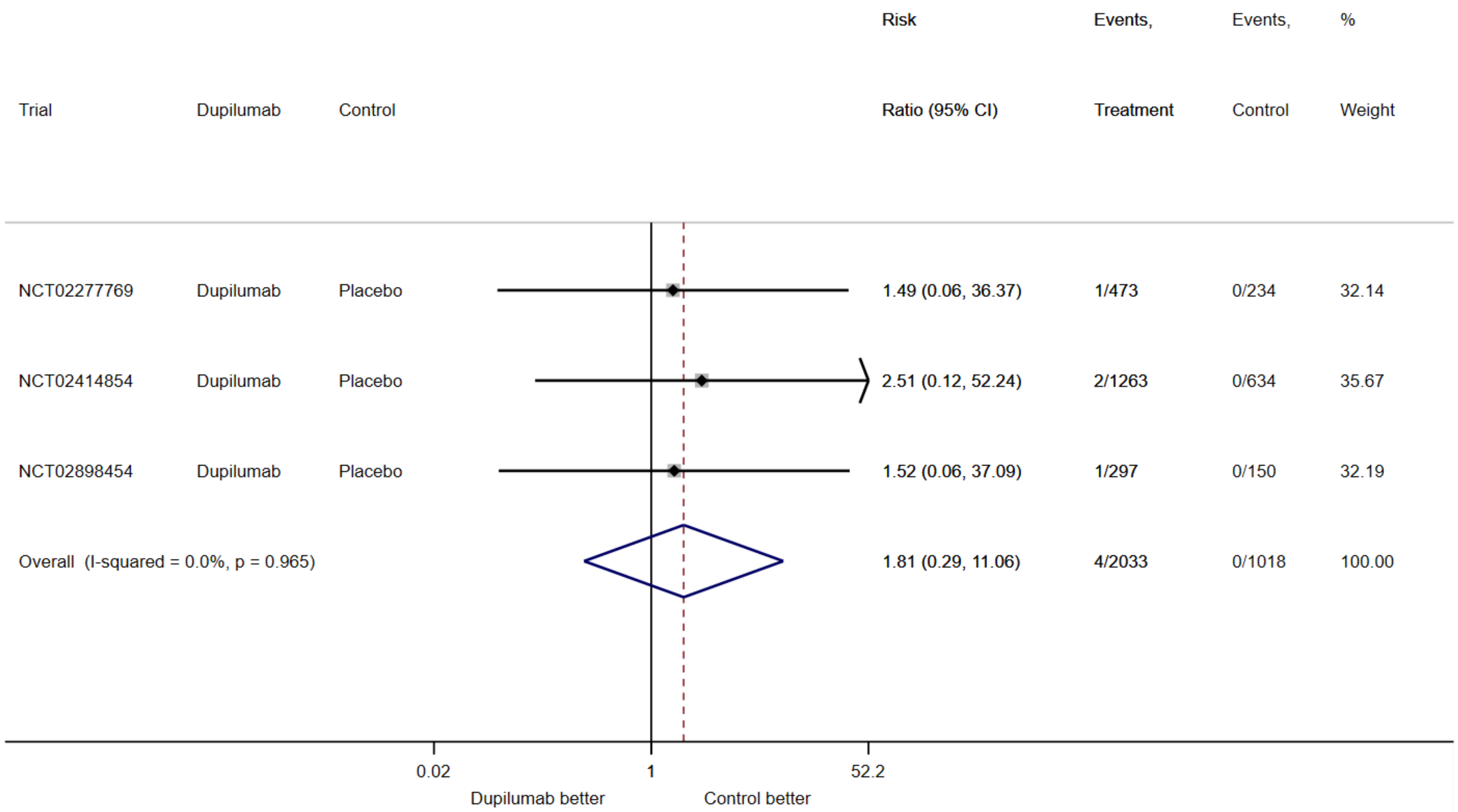

9_Injury, Poisoning And Procedural Complications-Figure S6 Meta-analysis of Dupilumab and Fall

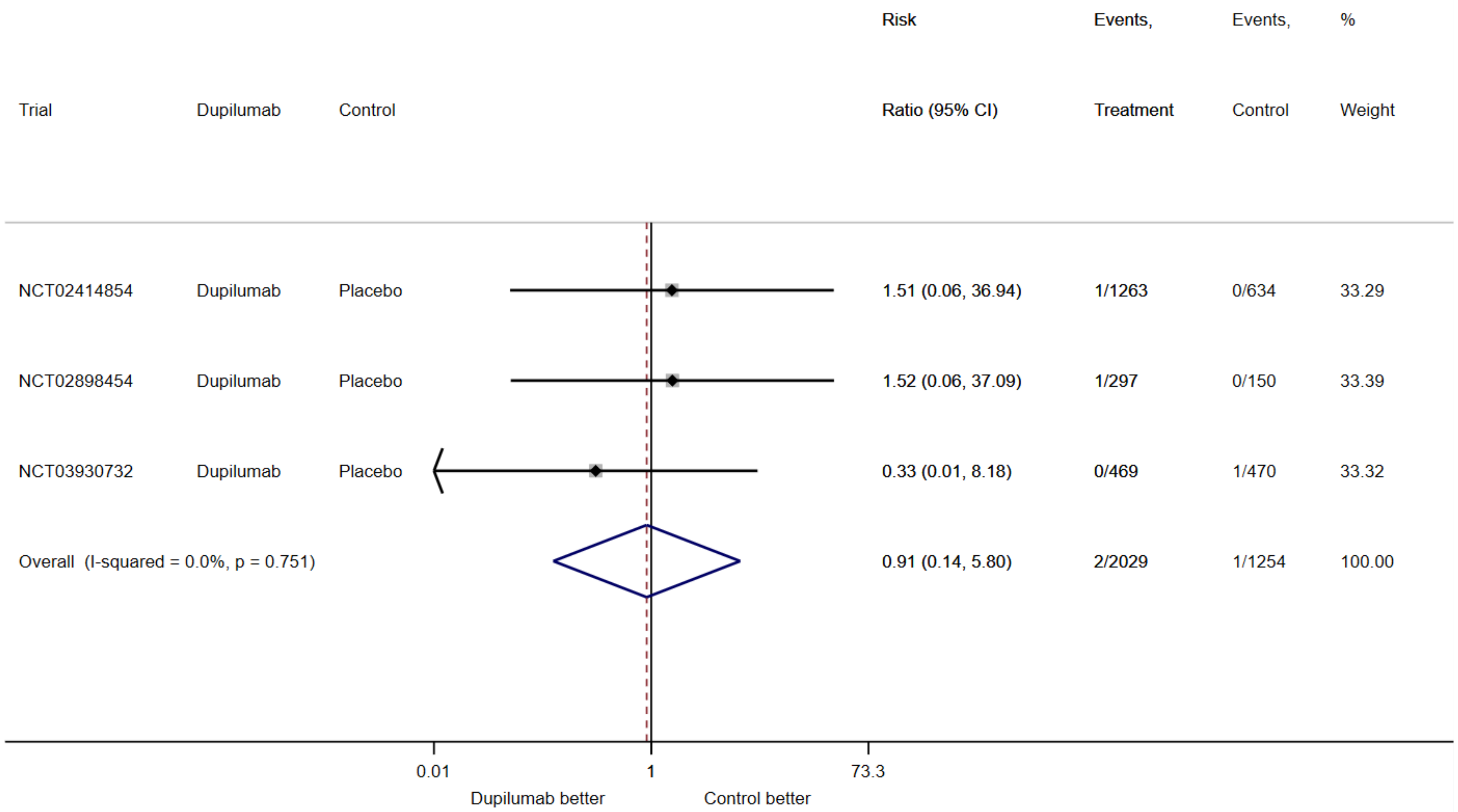

9_Injury, Poisoning And Procedural Complications-Figure S7 Meta-analysis of Dupilumab and Femur fracture

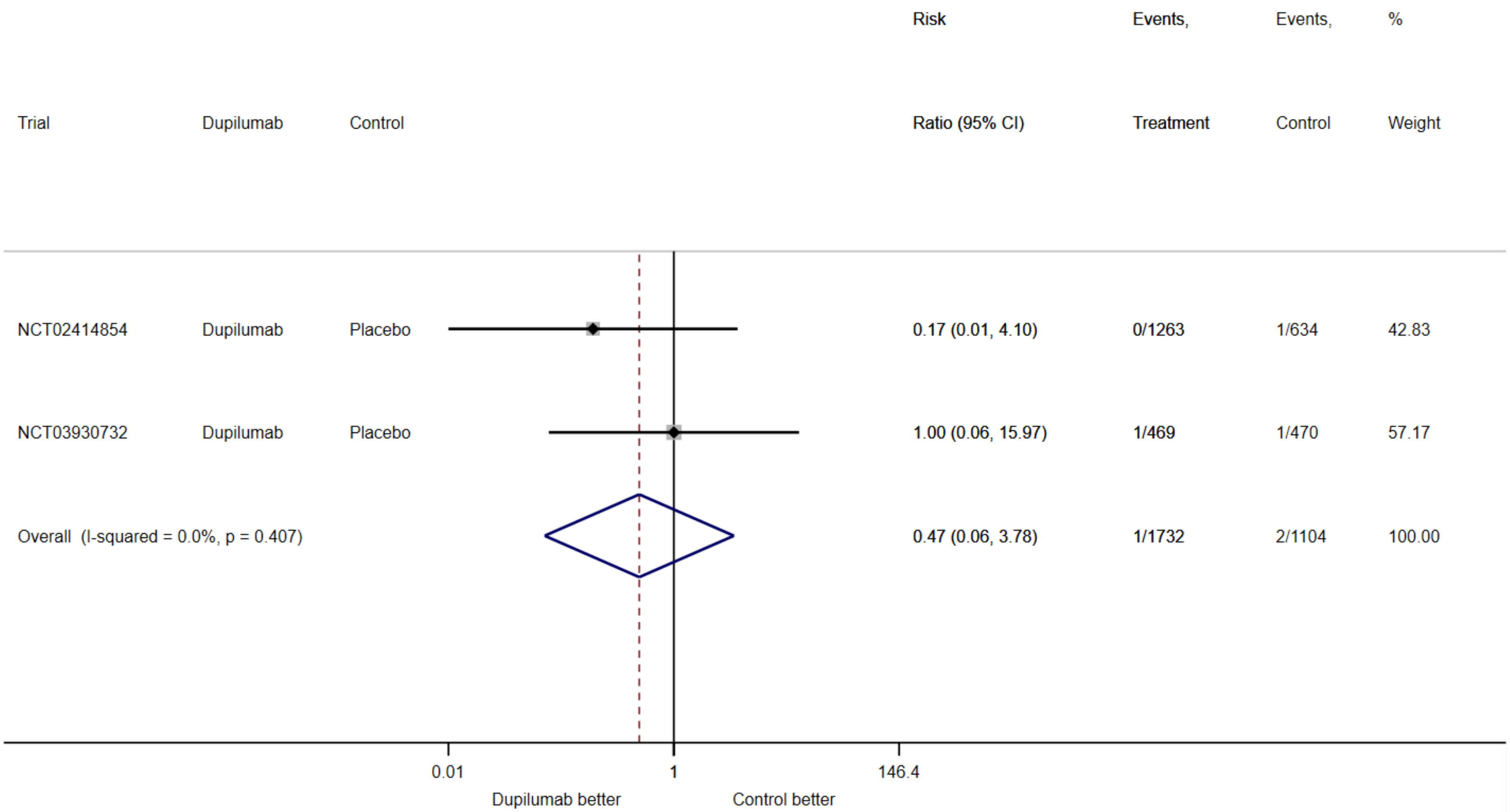

9_Injury, Poisoning And Procedural Complications-Figure S8 Meta-analysis of Dupilumab and Fibula fracture

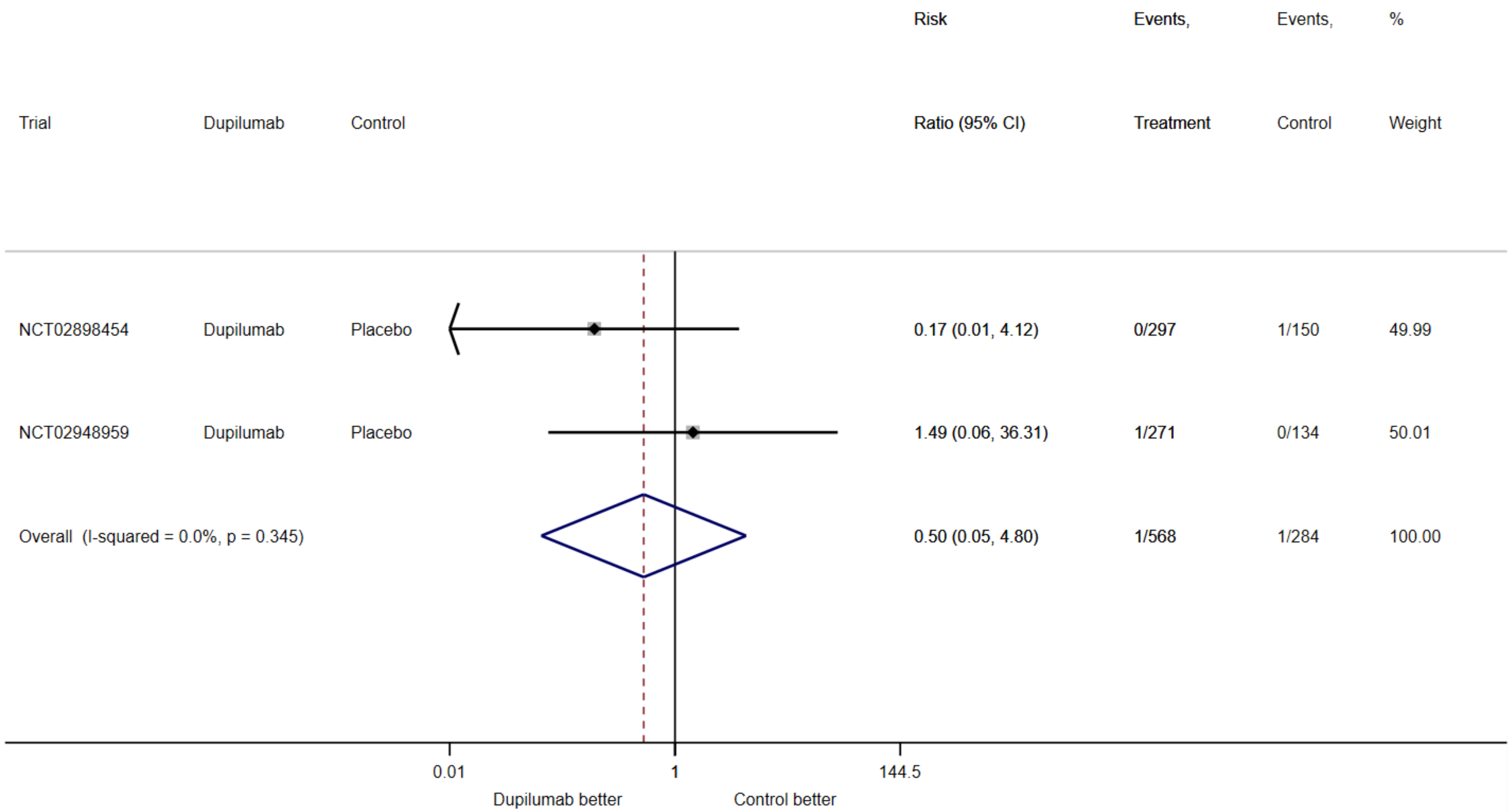

9_Injury, Poisoning And Procedural Complications-Figure S9 Meta-analysis of Dupilumab and Hand fracture

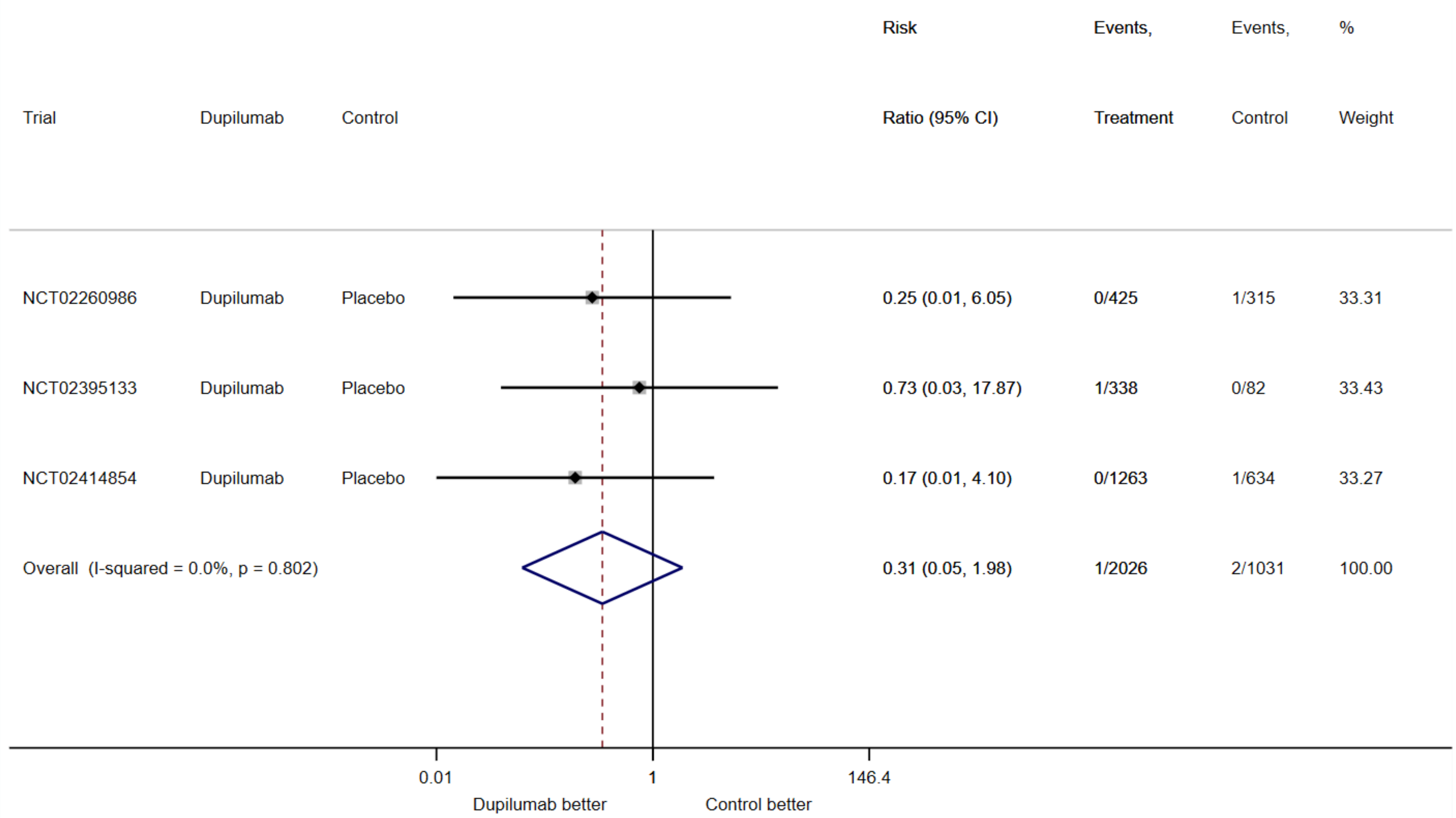

9_Injury, Poisoning And Procedural Complications-Figure S10 Meta-analysis of Dupilumab and Ligament rupture

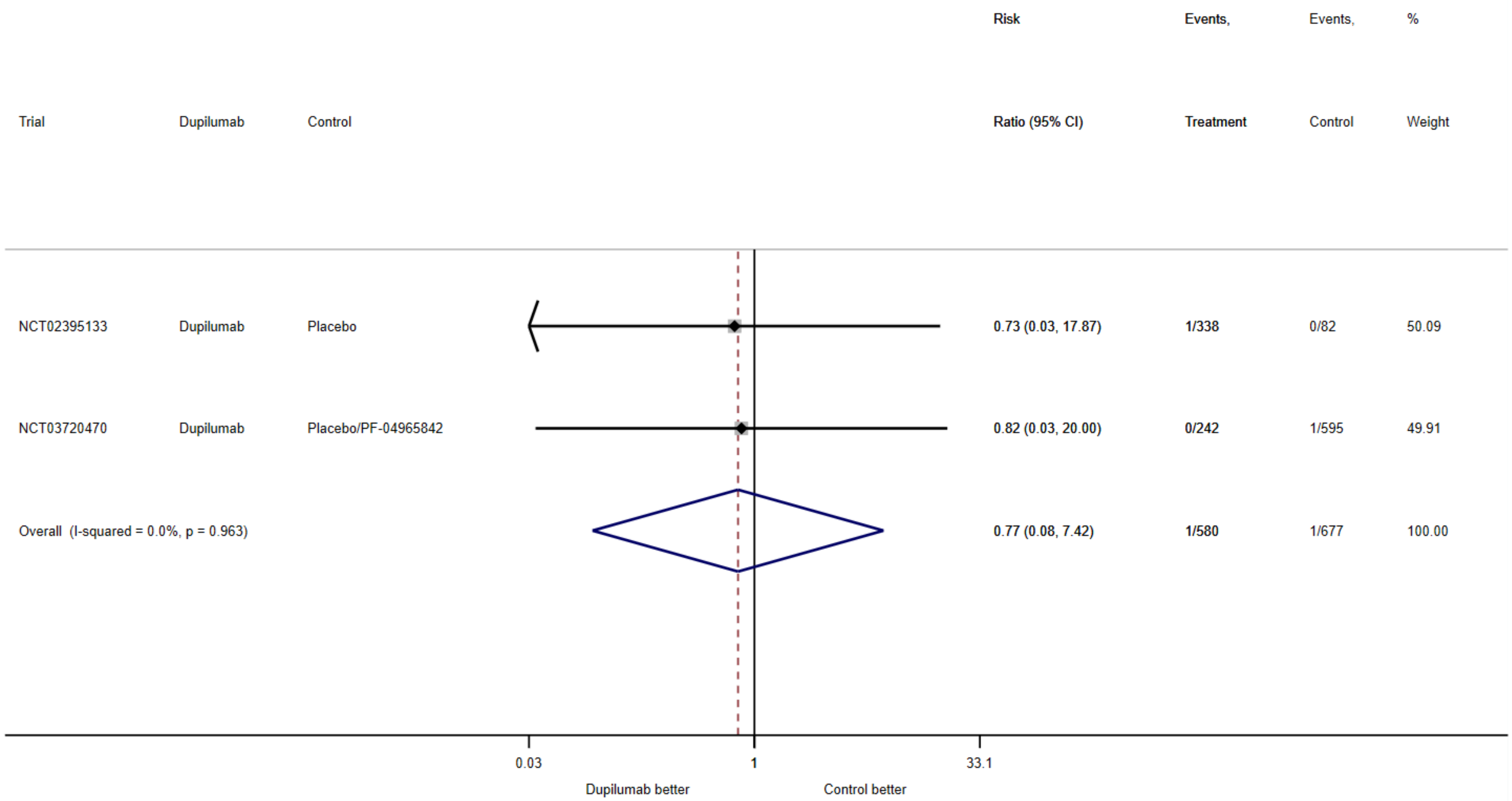

9_Injury, Poisoning And Procedural Complications-Figure S11 Meta-analysis of Dupilumab and Muscle injury

Trial	Dupilumab	Control	Risk Ratio (95% CI)	Events, Treatment	Events, Control	% Weight
NCT02414854	Dupilumab	Placebo	0.17 (0.01, 4.10)	0/1263	1/634	42.83
NCT03930732	Dupilumab	Placebo	1.00 (0.06, 15.97)	1/469	1/470	57.17
Overall (I-squared = 0.0%, p = 0.407)			0.47 (0.06, 3.78)	1/1732	2/1104	100.00

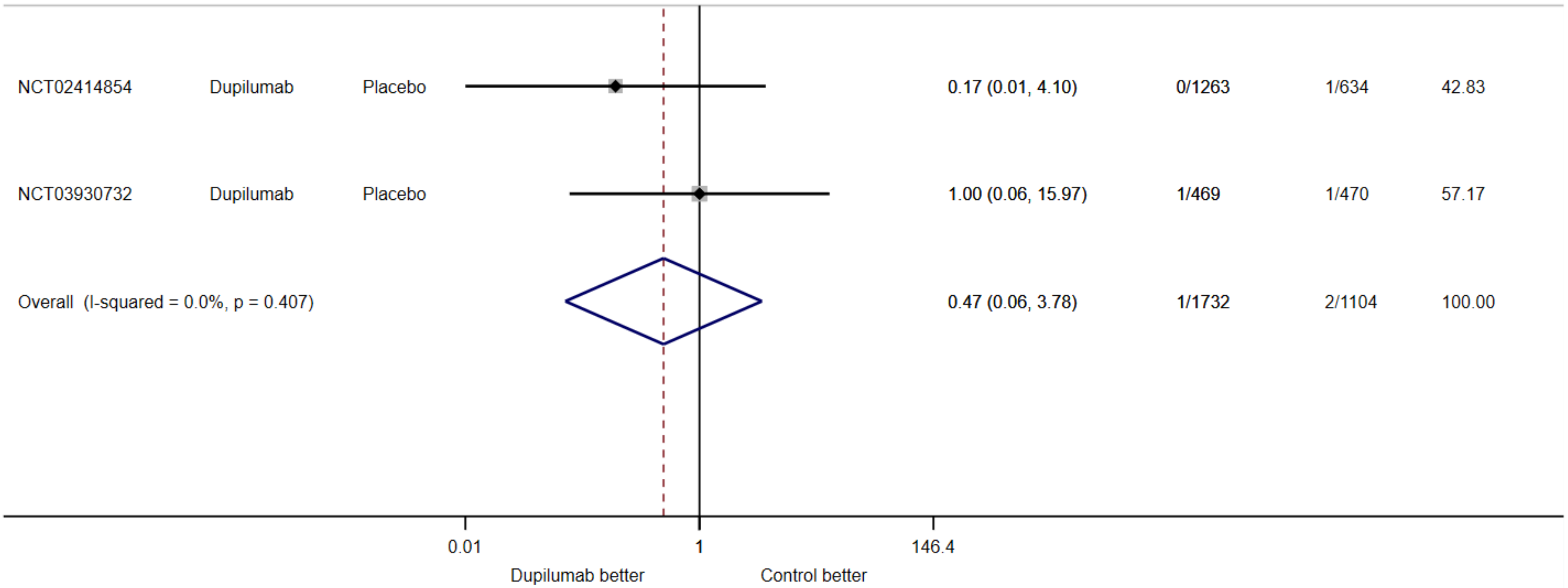

9_Injury, Poisoning And Procedural Complications-Figure S12 Meta-analysis of Dupilumab and Pneumothorax traumatic

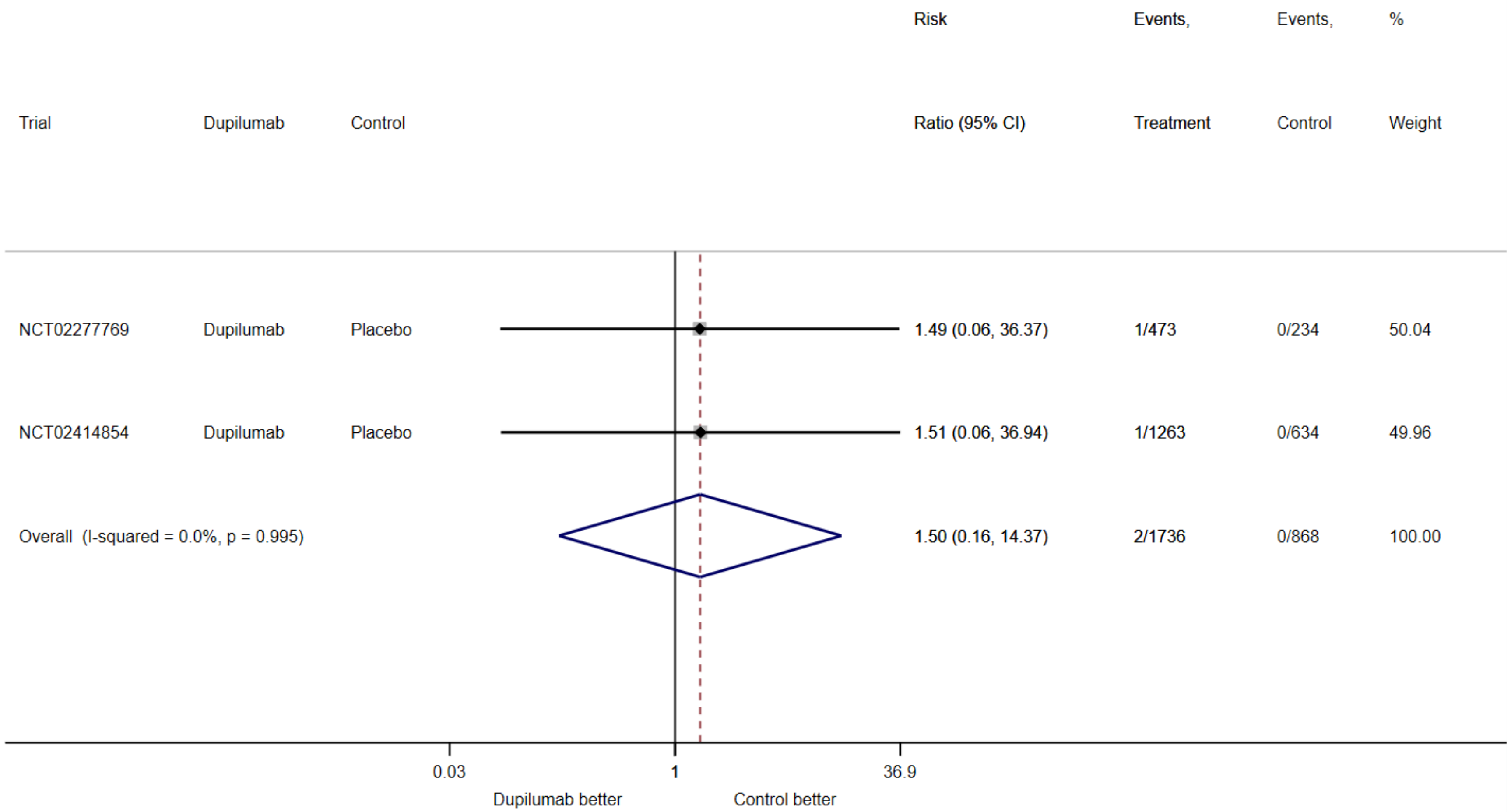

9_Injury, Poisoning And Procedural Complications-Figure S13 Meta-analysis of Dupilumab and Radius fracture

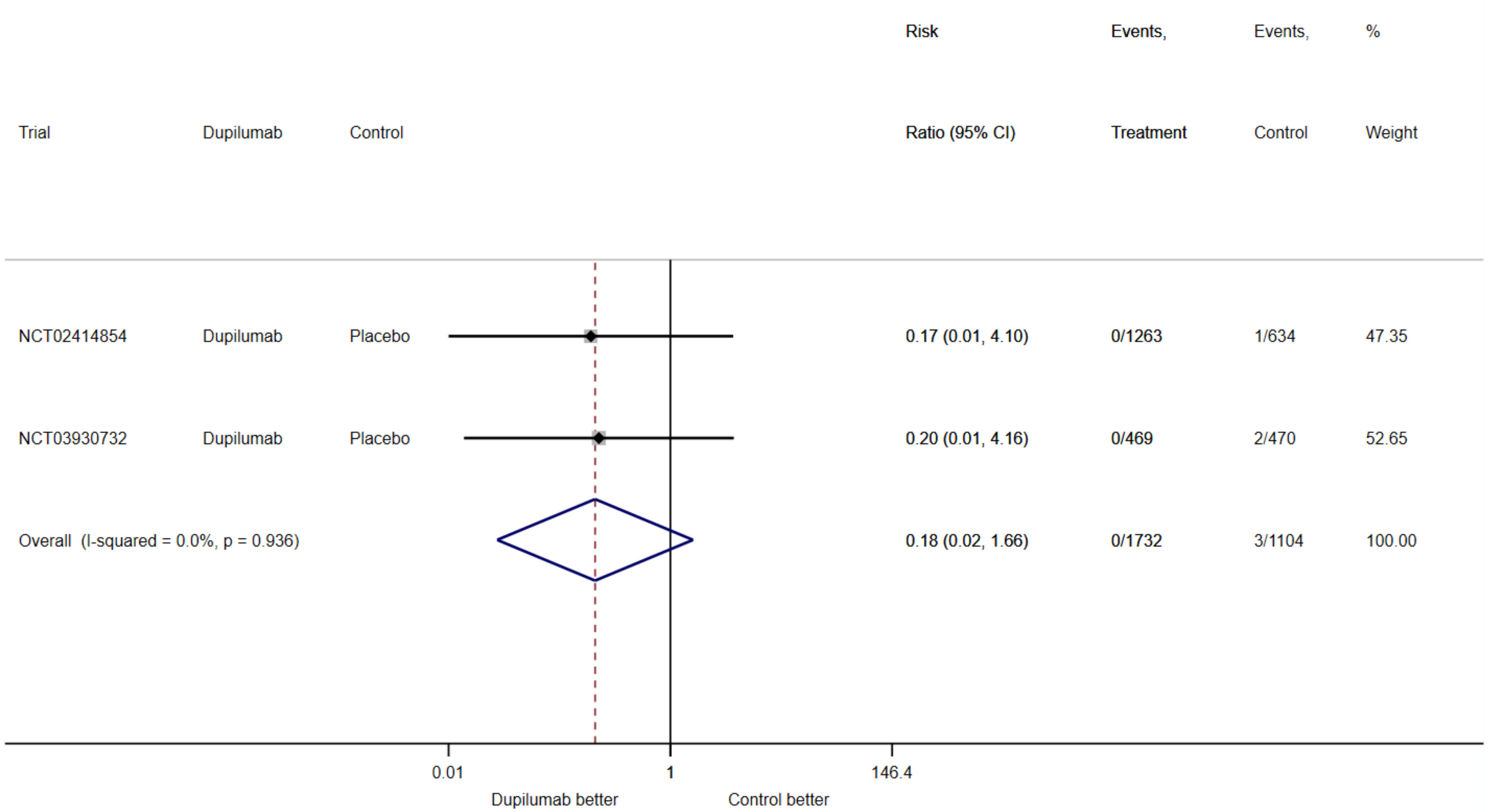

9_Injury, Poisoning And Procedural Complications-Figure S14 Meta-analysis of Dupilumab and Rib fracture

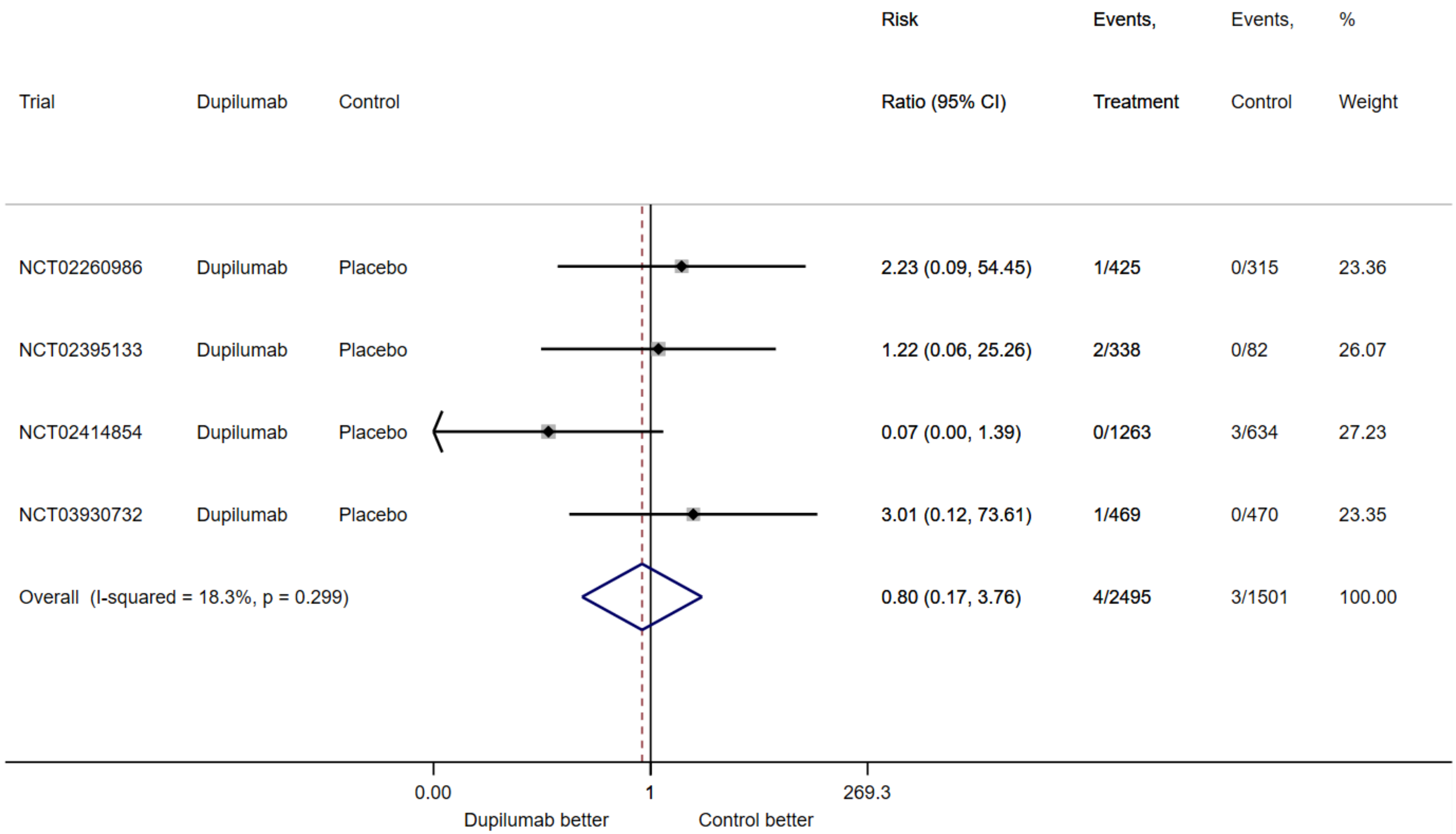

9_Injury, Poisoning And Procedural Complications-Figure S15 Meta-analysis of Dupilumab and Road traffic accident

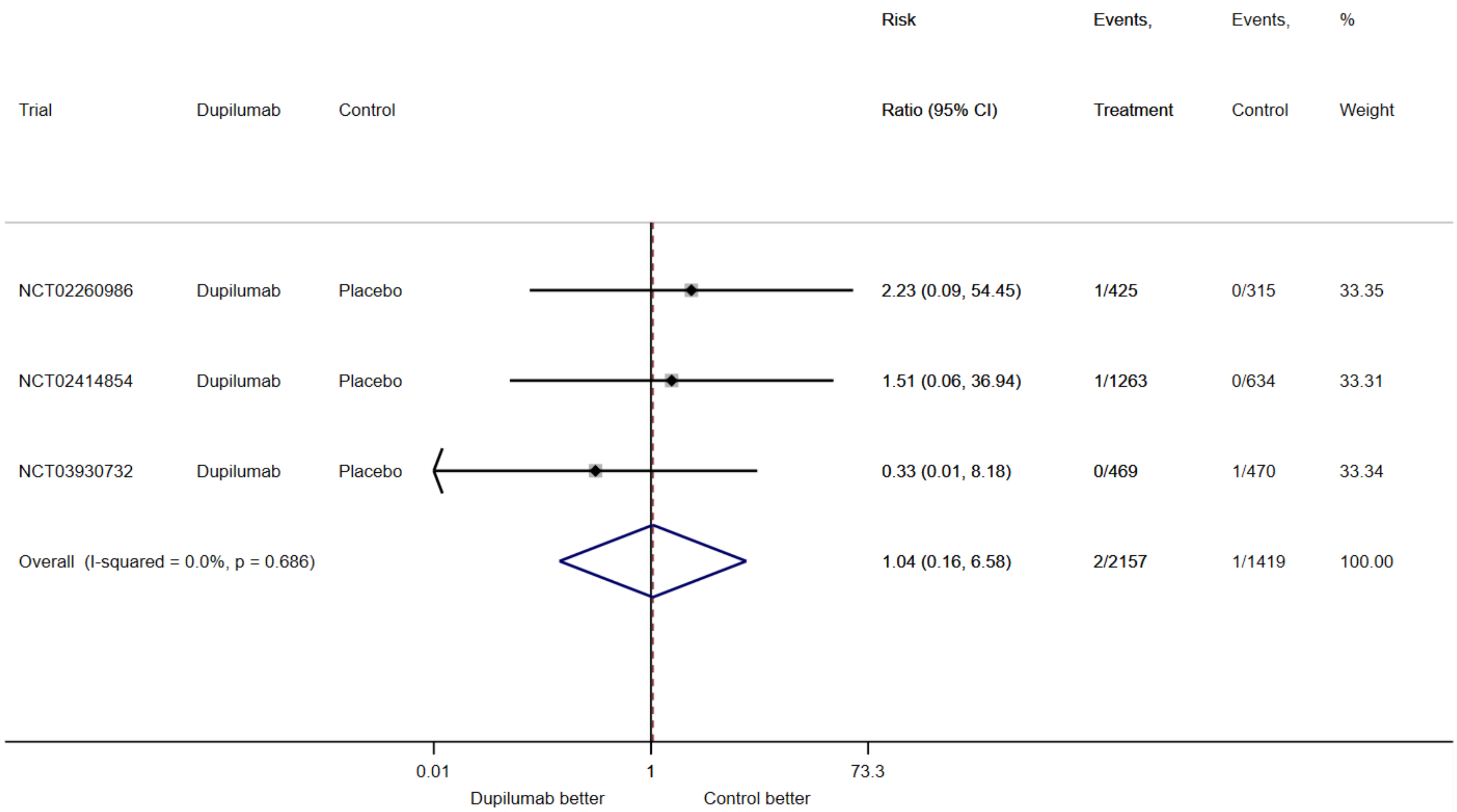

9_Injury, Poisoning And Procedural Complications-Figure S16 Meta-analysis of Dupilumab and Spinal compression fracture

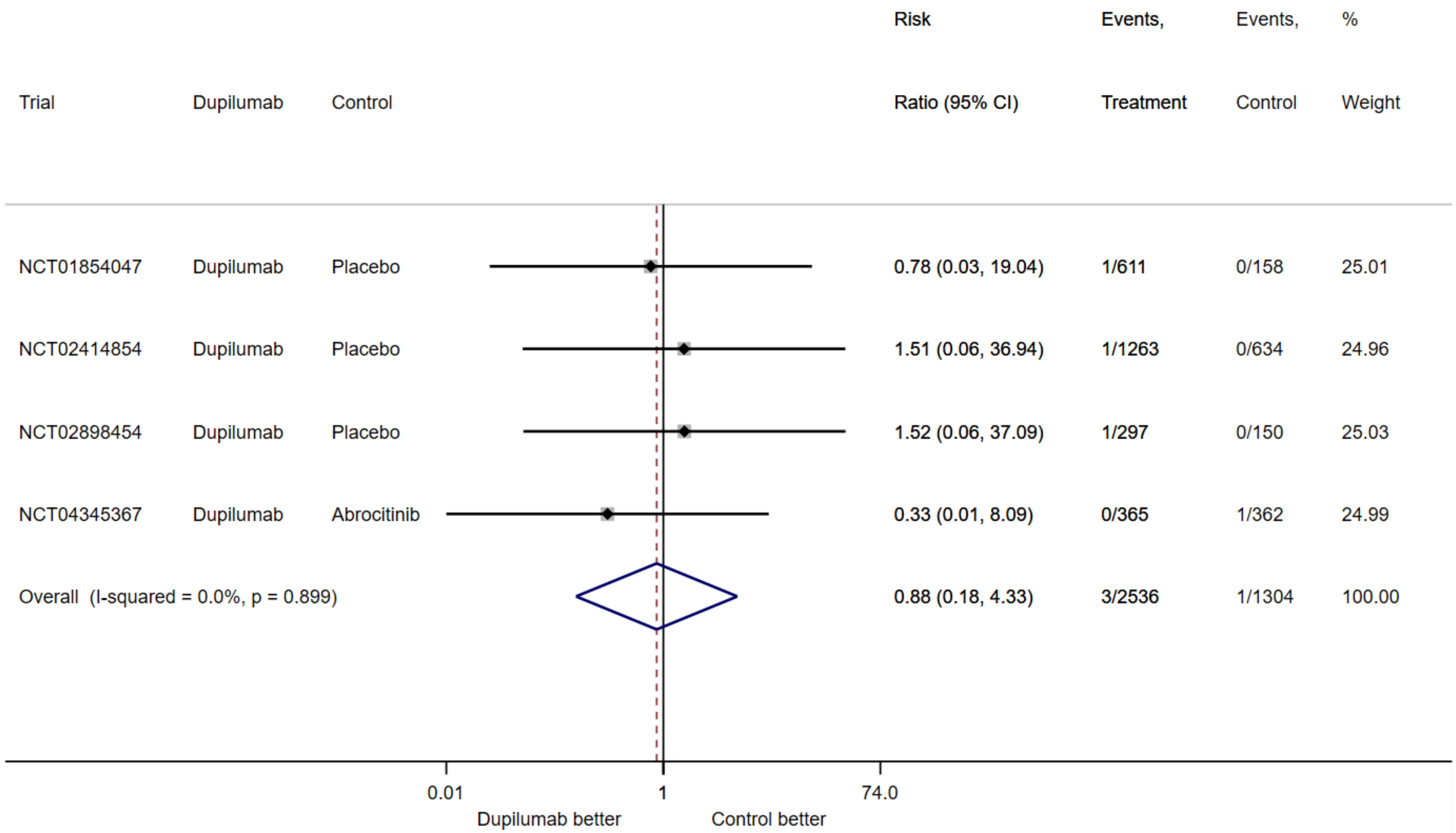

9_Injury, Poisoning And Procedural Complications-Figure S17 Meta-analysis of Dupilumab and Upper limb fracture

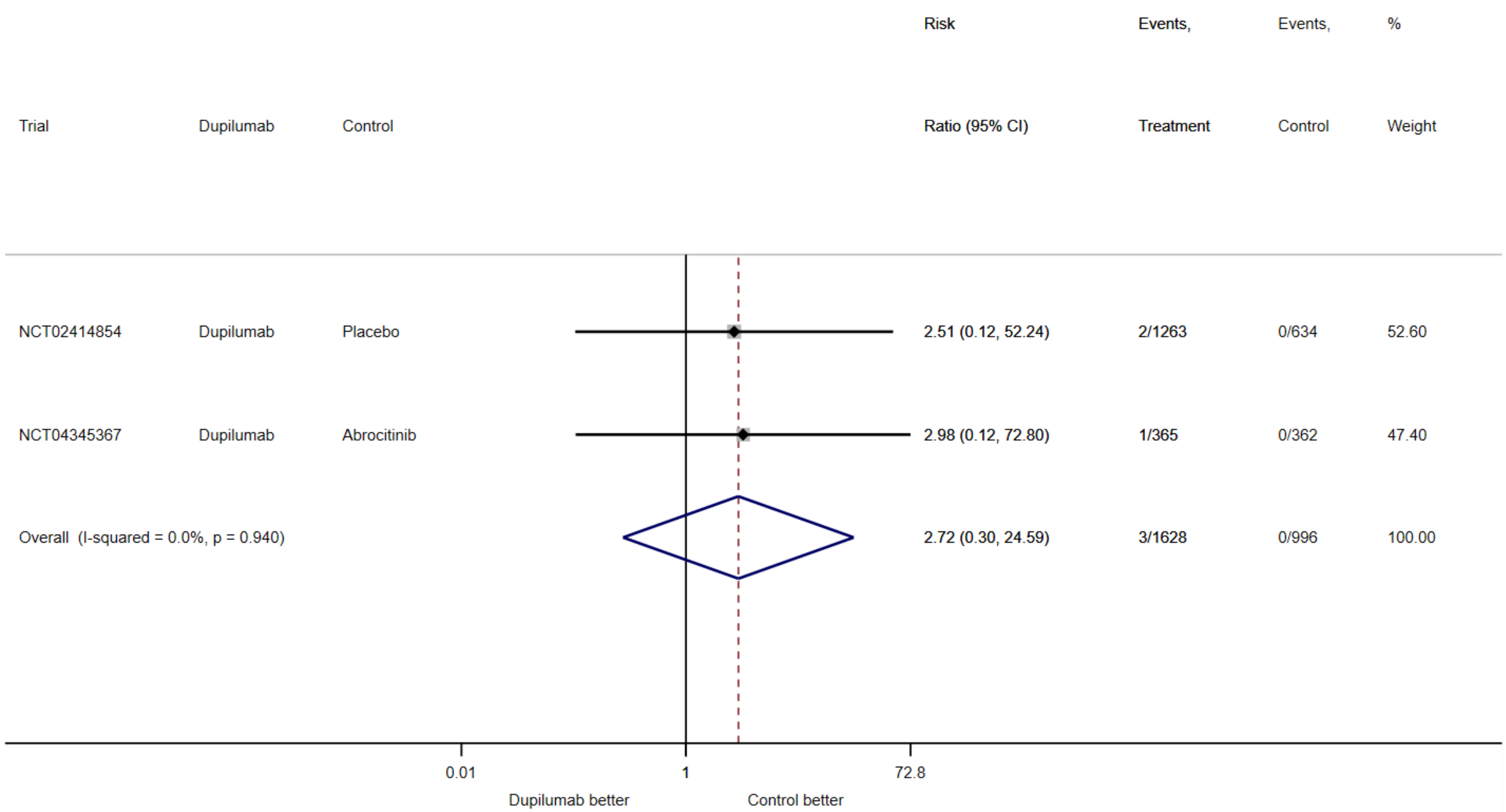

10_Investigations-Figure S1 Meta-analysis of Dupilumab and Alanine aminotransferase increased

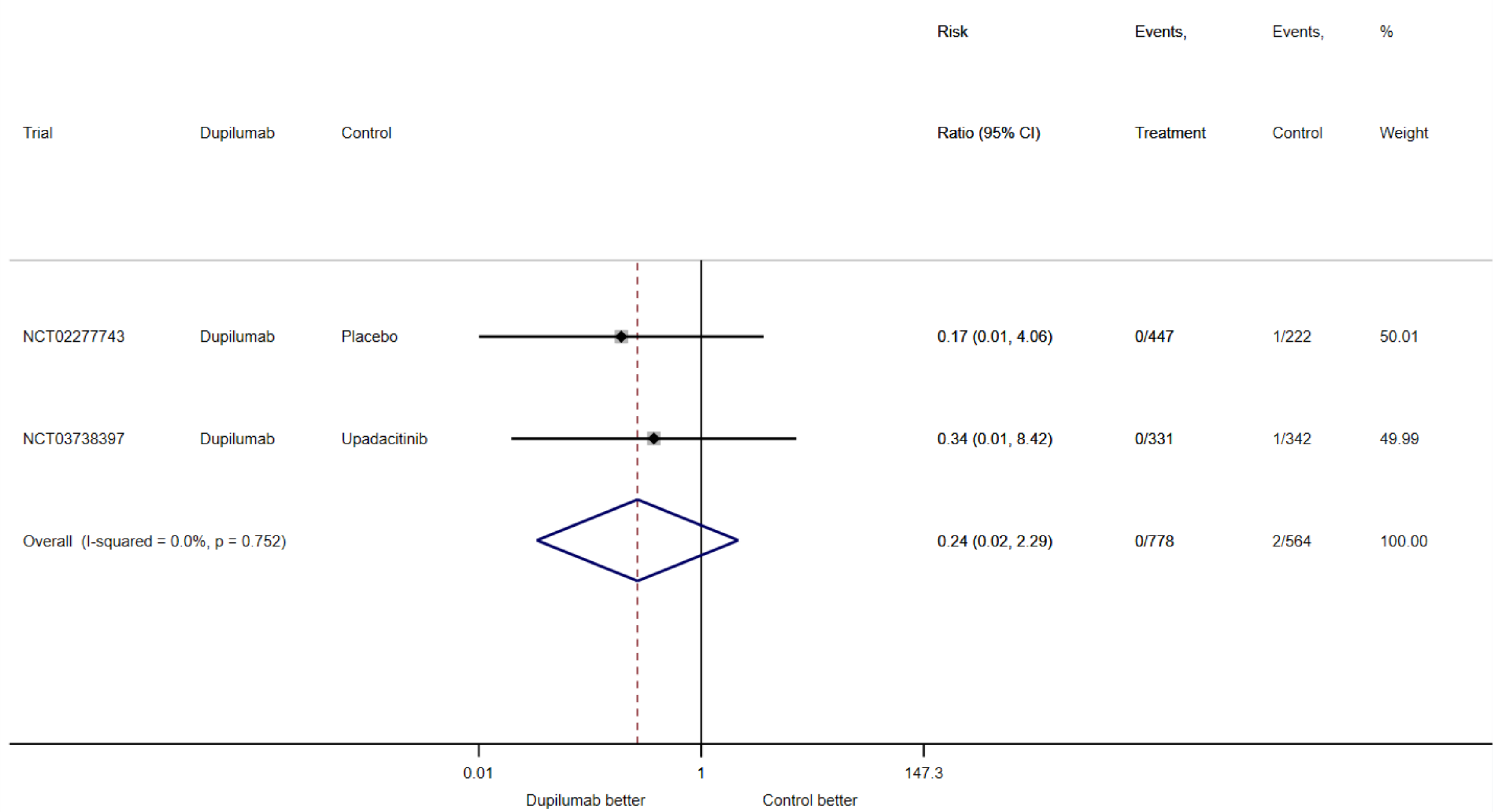

11_Metabolism And Nutrition Disorders-Figure S1 Meta-analysis of Dupilumab and Hyperglycaemia

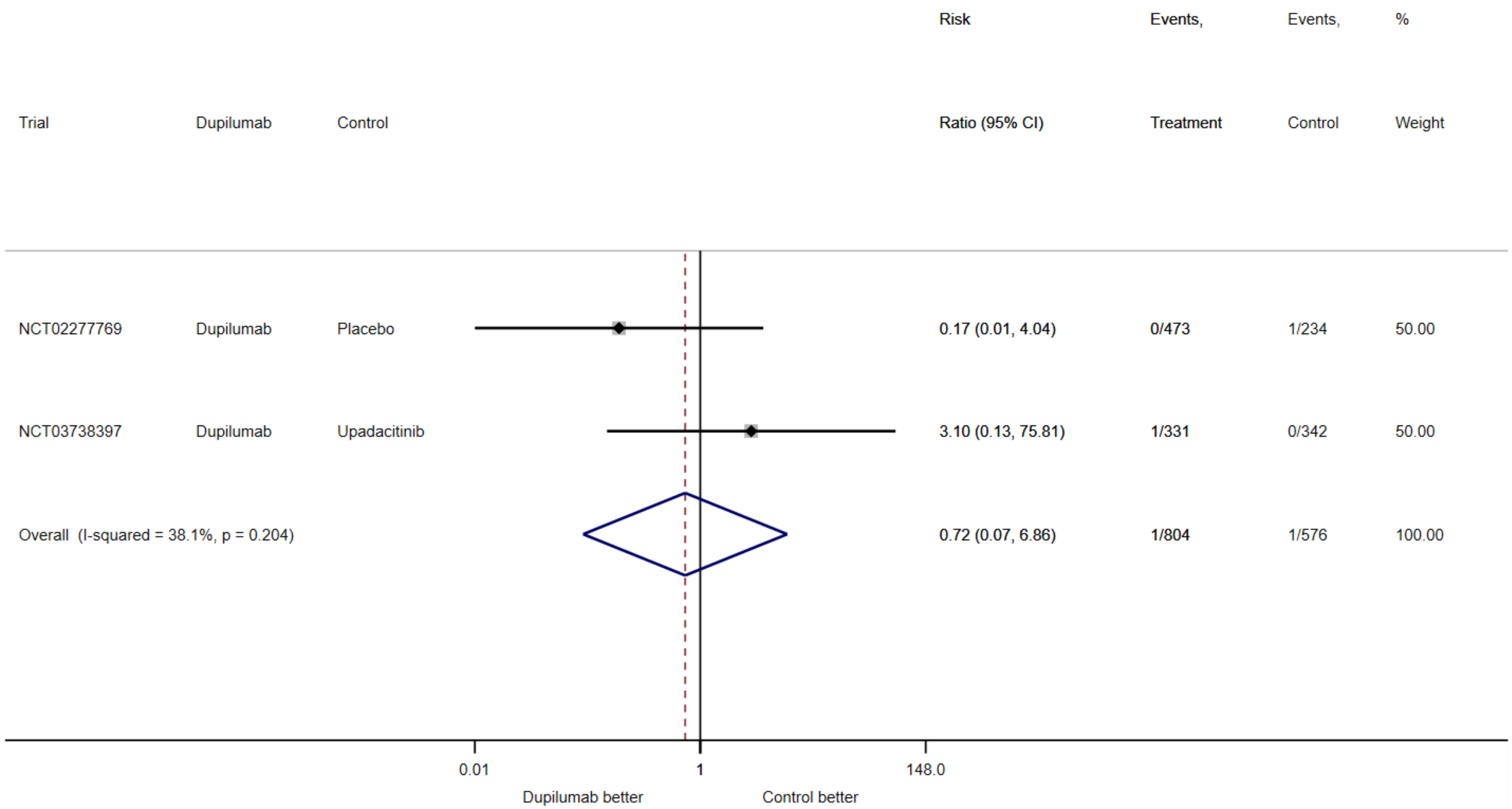

12_Musculoskeletal And Connective Tissue Disorders-Figure S1 Meta-analysis of Dupilumab and Bursitis

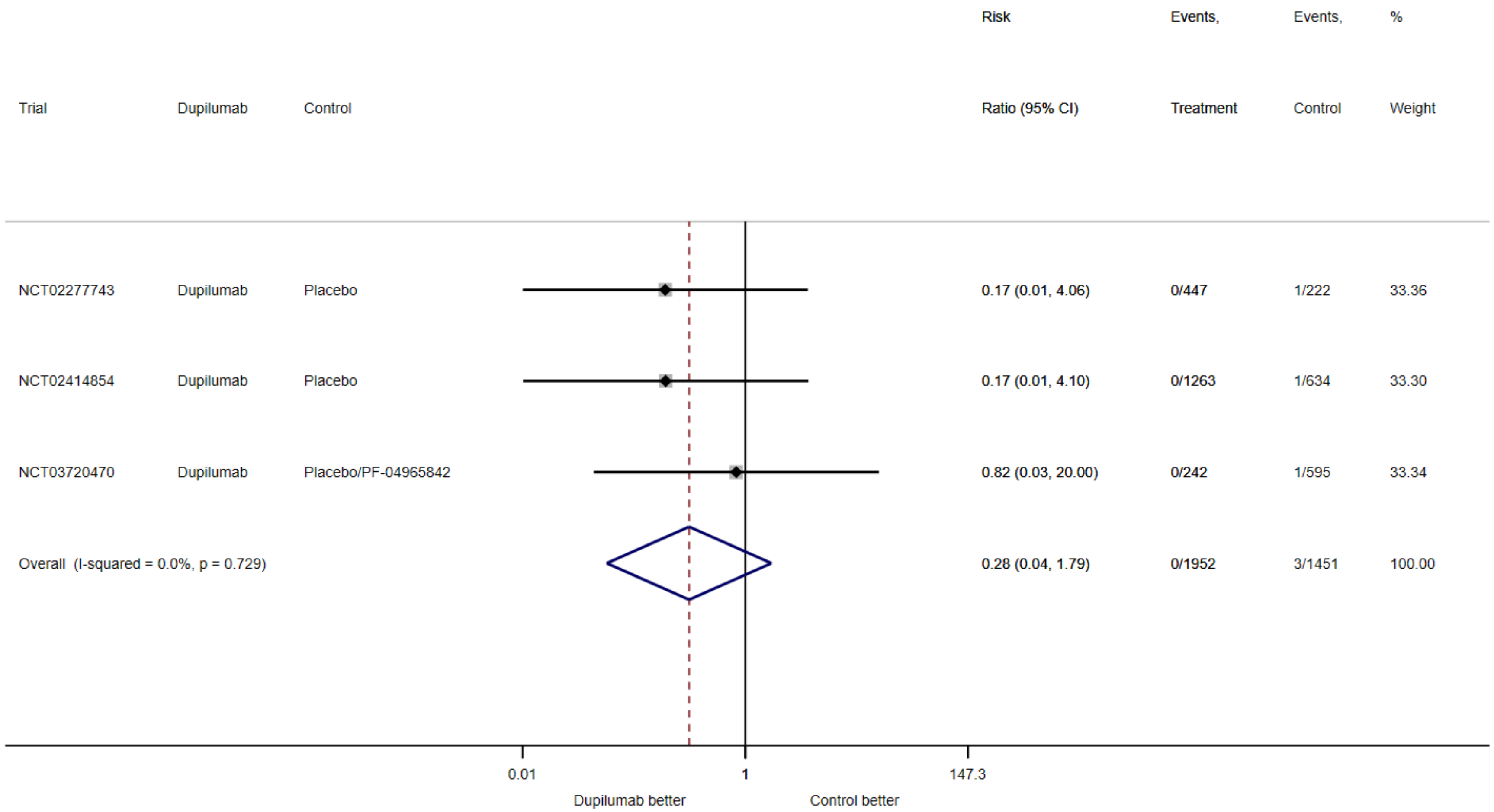

12_Musculoskeletal And Connective Tissue Disorders-Figure S2 Meta-analysis of Dupilumab and Intervertebral disc protrusion

Trial	Dupilumab	Control	Risk Ratio (95% CI)	Events, Treatment	Events, Control	% Weight
NCT02414854	Dupilumab	Placebo	0.50 (0.03, 8.01)	1/1263	1/634	57.12
NCT04345367	Dupilumab	Abrocitinib	2.98 (0.12, 72.80)	1/365	0/362	42.88
Overall (I-squared = 0.0%, p = 0.410)			1.08 (0.13, 8.74)	2/1628	1/996	100.00

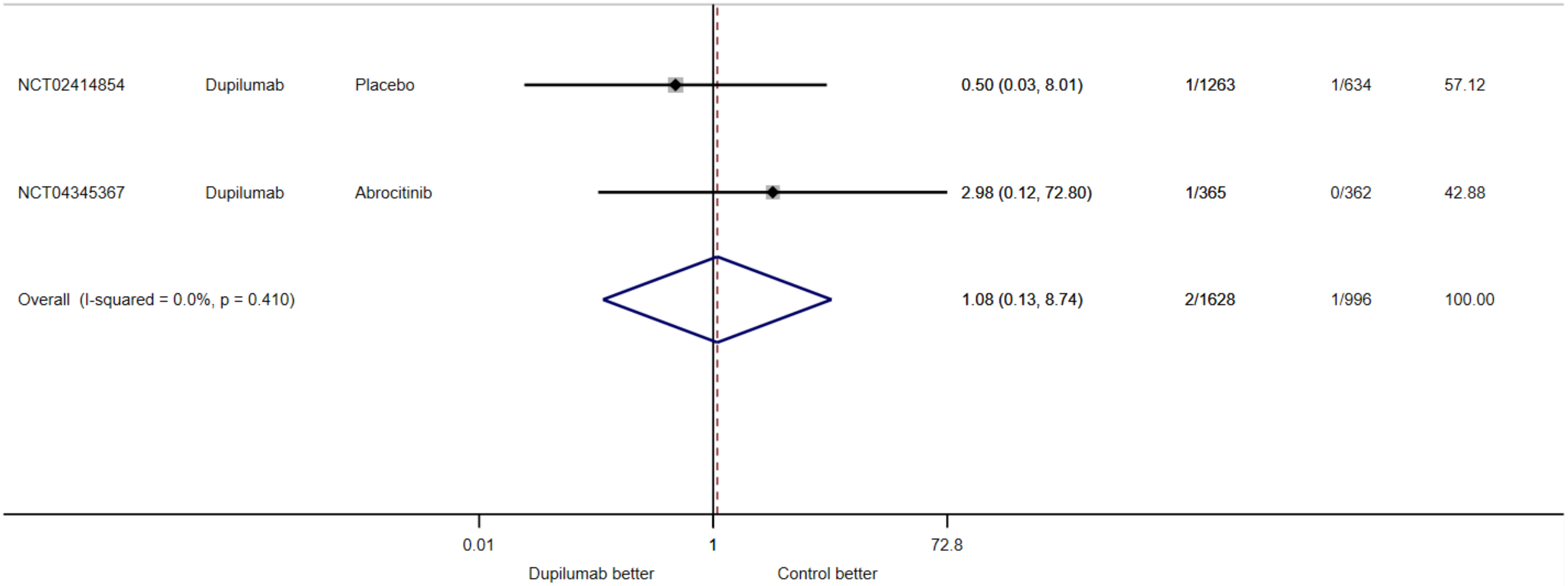

12_Musculoskeletal And Connective Tissue Disorders-Figure S3 Meta-analysis of Dupilumab and Musculoskeletal chest pain

Trial	Dupilumab	Control	Risk Ratio (95% CI)	Events, Treatment	Events, Control	% Weight
NCT02260986	Dupilumab	Placebo	2.23 (0.09, 54.45)	1/425	0/315	19.22
NCT02414854	Dupilumab	Placebo	0.33 (0.06, 2.00)	2/1263	3/634	61.54
NCT02898454	Dupilumab	Placebo	1.52 (0.06, 37.09)	1/297	0/150	19.25
Overall (I-squared = 0.0%, p = 0.504)			0.64 (0.16, 2.62)	4/1985	3/1099	100.00

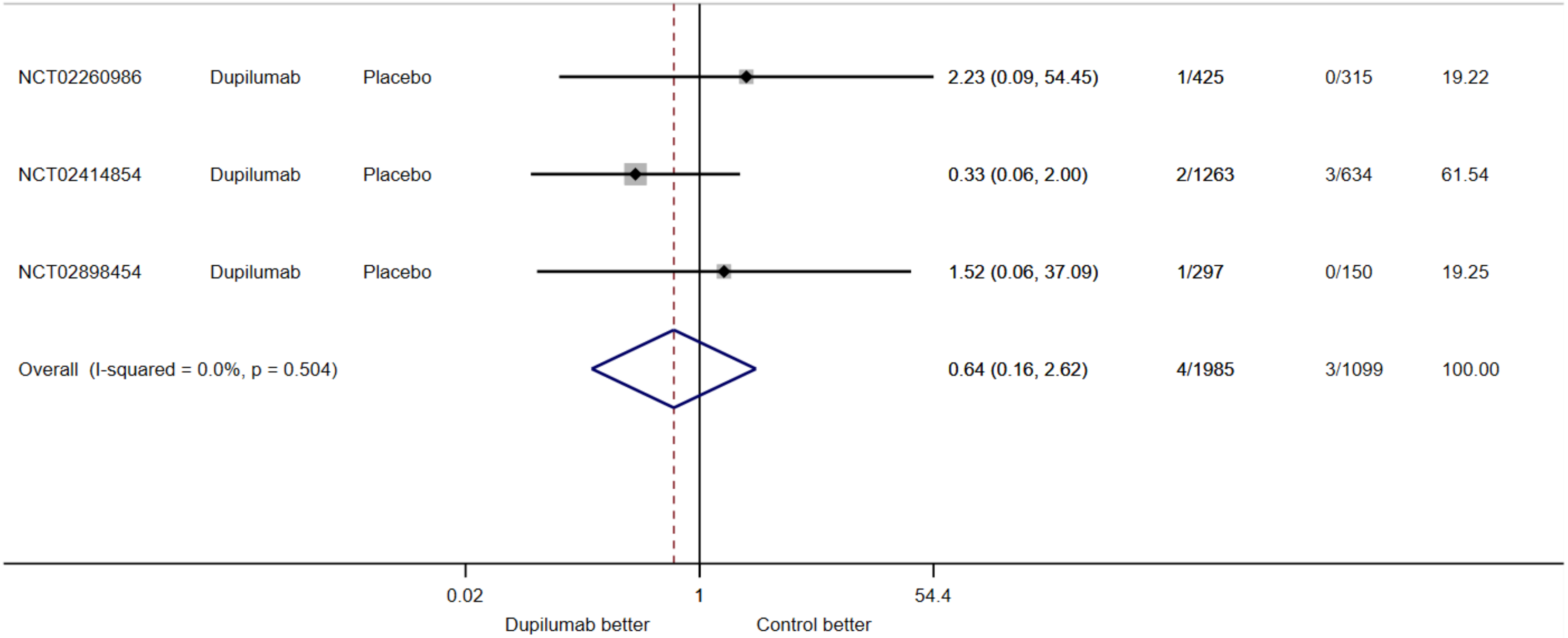

12_Musculoskeletal And Connective Tissue Disorders-Figure S4 Meta-analysis of Dupilumab and Osteoarthritis

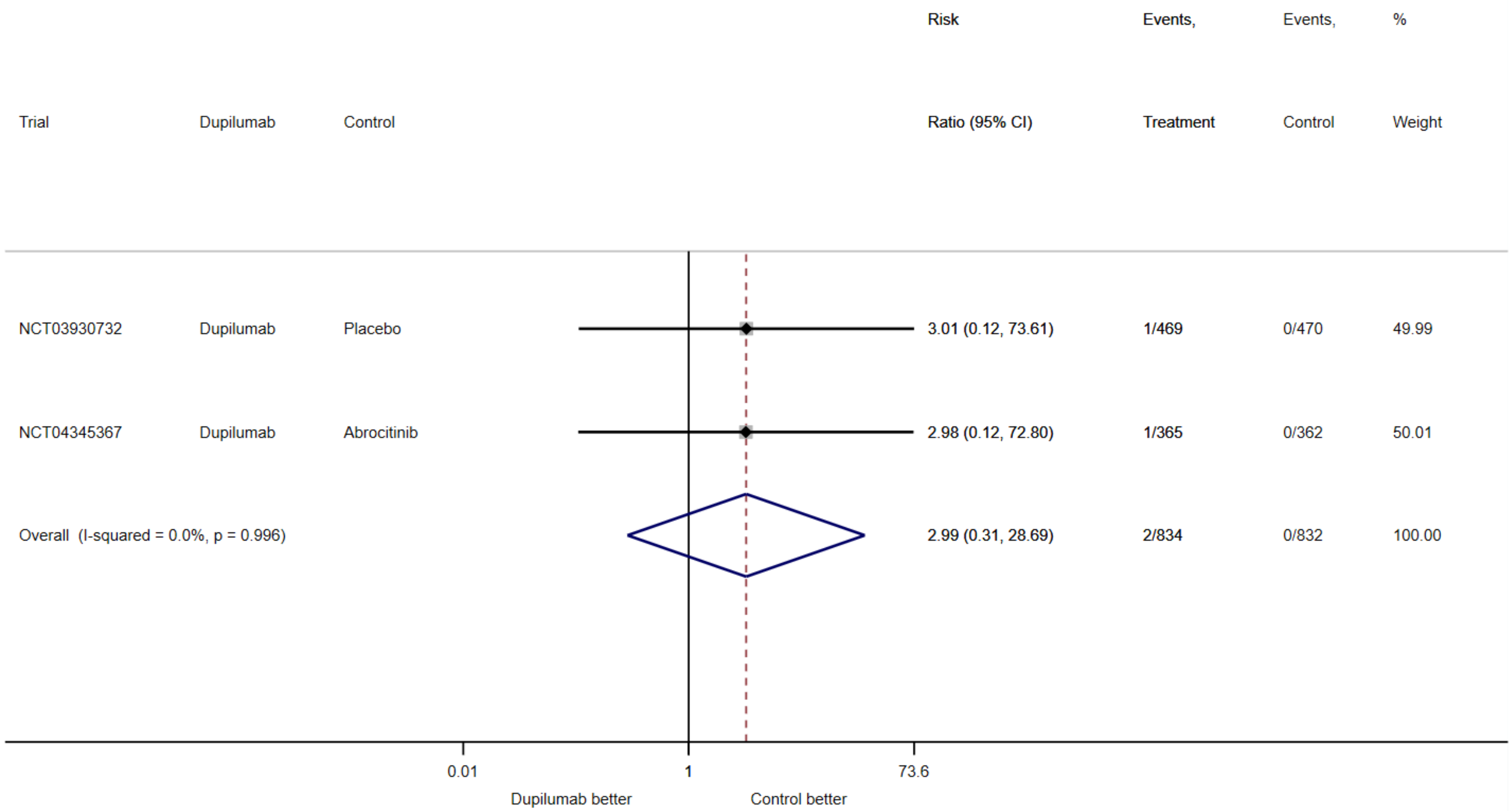

12_Musculoskeletal And Connective Tissue Disorders-Figure S5 Meta-analysis of Dupilumab and Rhabdomyolysis

Trial	Dupilumab	Control	Risk Ratio (95% CI)	Events, Treatment	Events, Control	% Weight
NCT02395133	Dupilumab	Placebo	1.22 (0.06, 25.26)	2/338	0/82	48.91
NCT02414854	Dupilumab	Placebo	3.52 (0.18, 67.98)	3/1263	0/634	51.09
Overall (I-squared = 0.0%, p = 0.625)			2.10 (0.25, 17.43)	5/1601	0/716	100.00

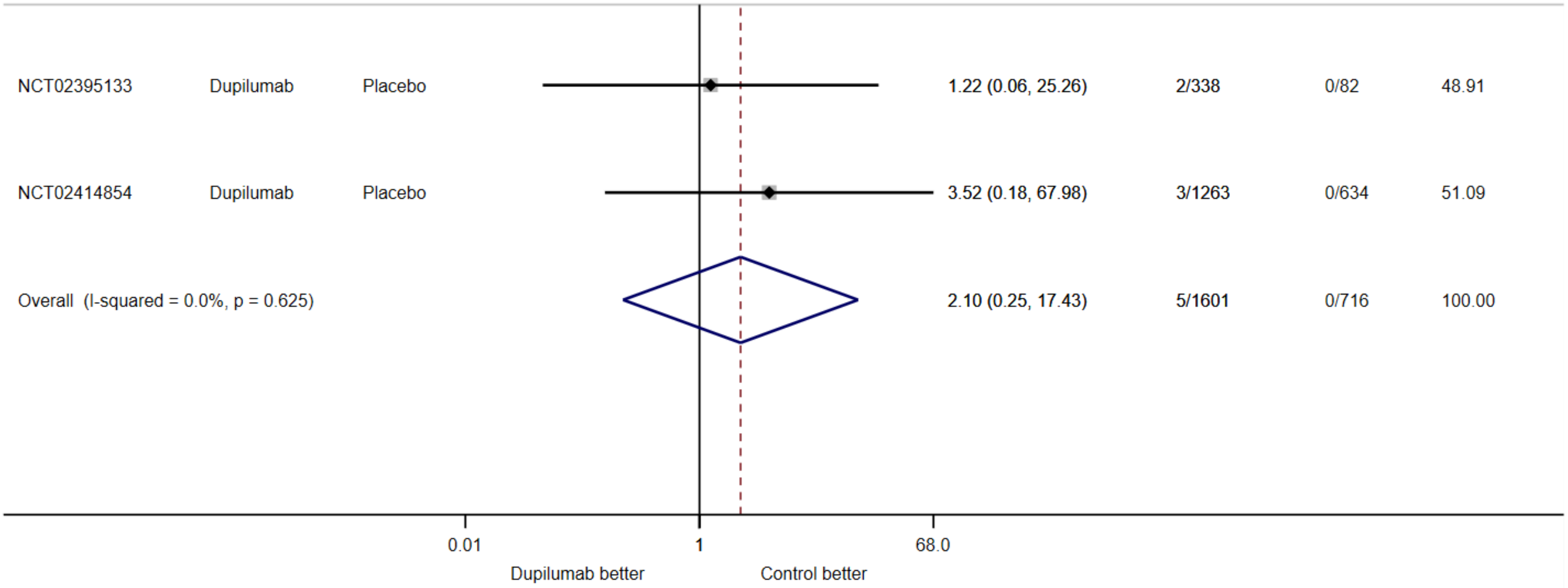

13_Neoplasms Benign, Malignant And Unspecified-Figure S1 Meta-analysis of Dupilumab and Basal cell carcinoma

Trial	Dupilumab	Control	Risk Ratio (95% CI)	Events, Treatment	Events, Control	% Weight
NCT02414854	Dupilumab	Placebo	0.10 (0.00, 2.09)	0/1263	2/634	52.53
NCT03633617	Dupilumab	Placebo	1.74 (0.07, 42.26)	1/203	0/117	47.47
Overall (I-squared = 37.8%, p = 0.205)			0.39 (0.04, 3.50)	1/1466	2/751	100.00

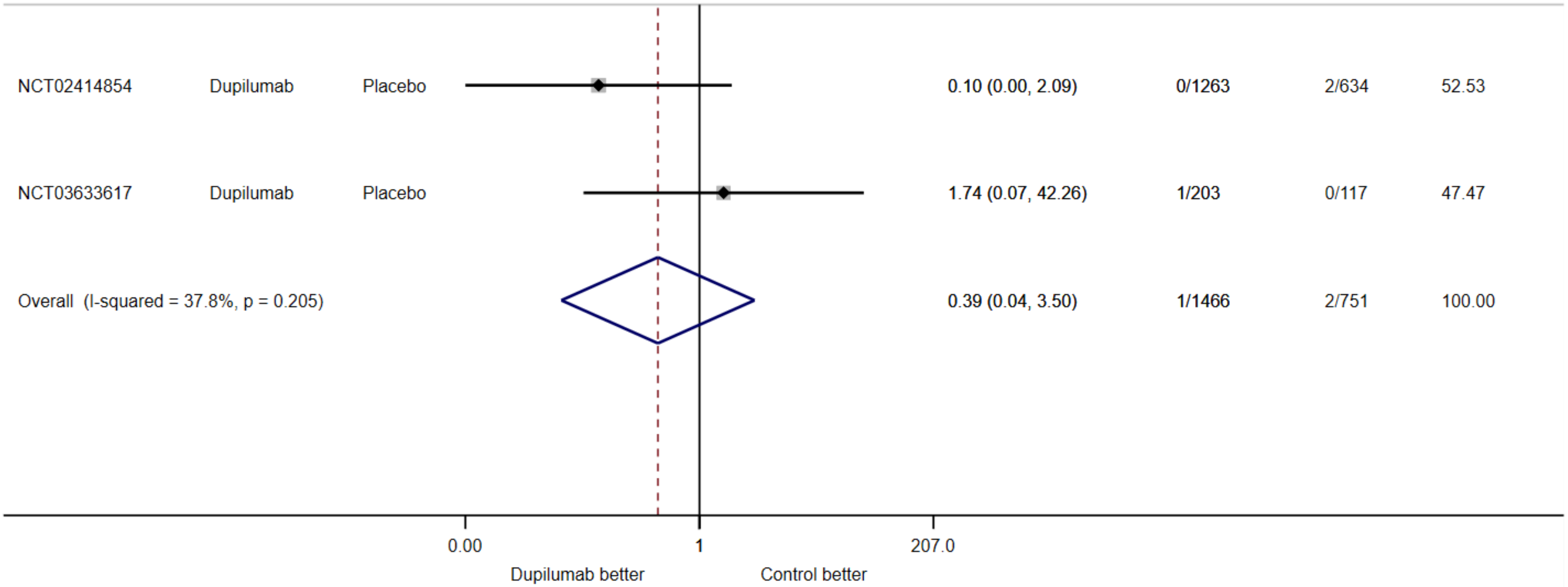

13_Neoplasms Benign, Malignant And Unspecified-Figure S2 Meta-analysis of Dupilumab and Breast cancer

Trial	Dupilumab	Control	Risk Ratio (95% CI)	Events, Treatment	Events, Control	% Weight
NCT02395133	Dupilumab	Placebo	0.73 (0.03, 17.87)	1/338	0/82	50.10
NCT03930732	Dupilumab	Placebo	3.01 (0.12, 73.61)	1/469	0/470	49.90
Overall (I-squared = 0.0%, p = 0.541)			1.48 (0.15, 14.21)	2/807	0/552	100.00

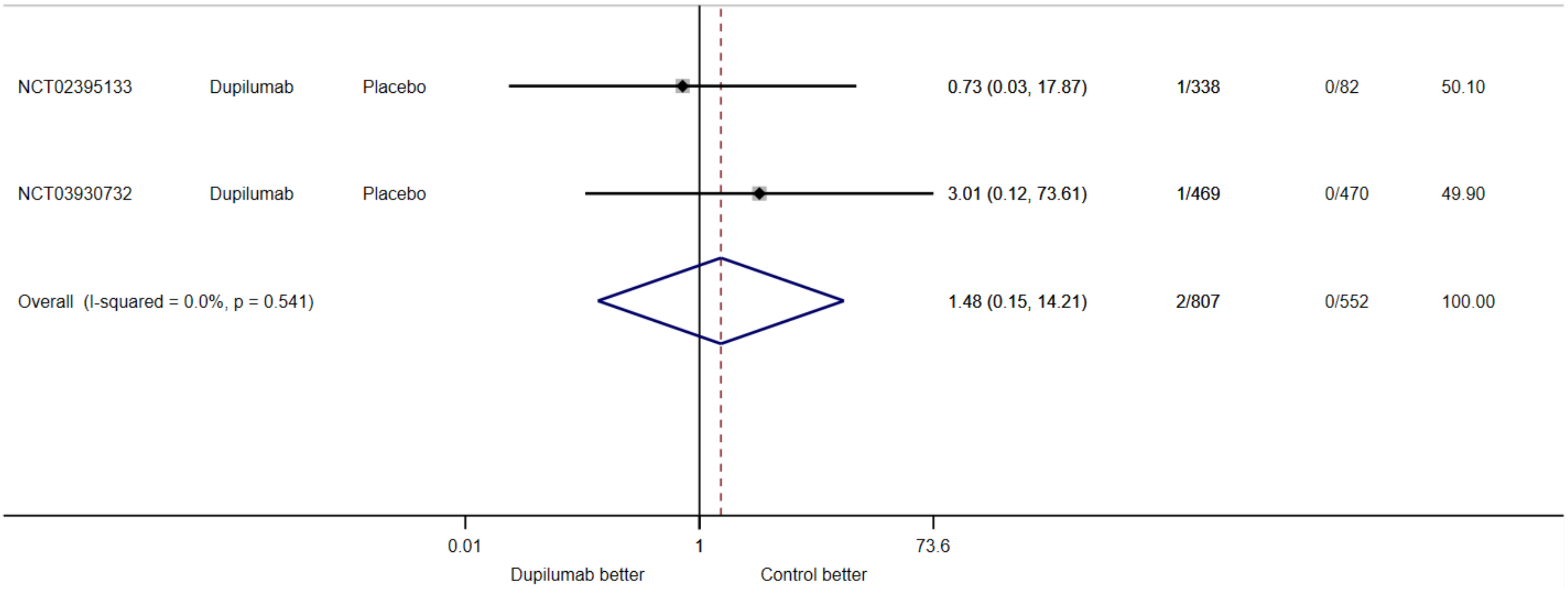

13_Neoplasms Benign, Malignant And Unspecified-Figure S3 Meta-analysis of Dupilumab and Glioblastoma

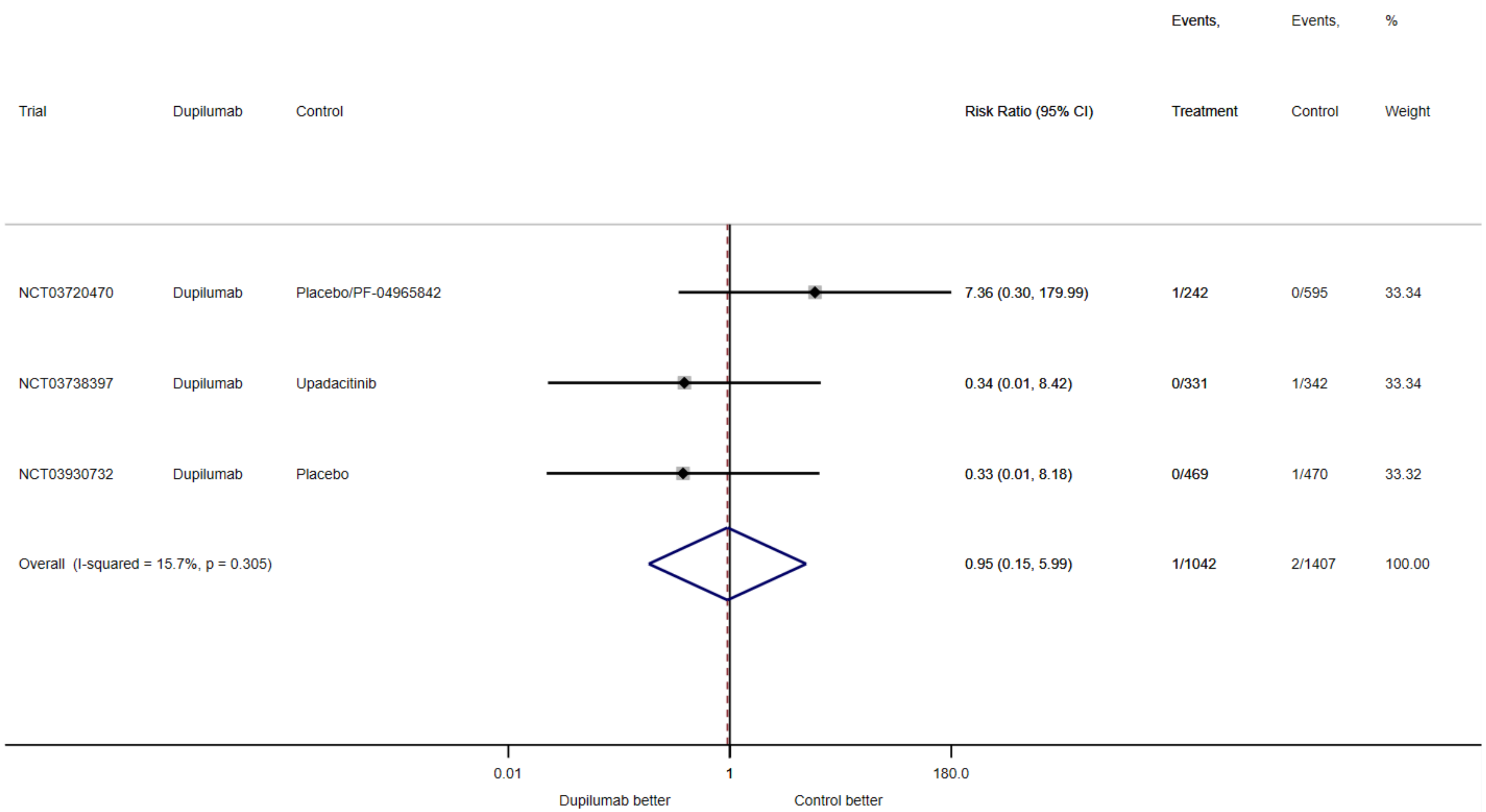

13_Neoplasms Benign, Malignant And Unspecified-Figure S4 Meta-analysis of Dupilumab and Invasive ductal breast carcinoma

Trial	Dupilumab	Control	Risk Ratio (95% CI)	Events, Treatment	Events, Control	% Weight
NCT02260986	Dupilumab	Placebo	3.71 (0.18, 76.99)	2/425	0/315	35.68
NCT02395133	Dupilumab	Placebo	0.73 (0.03, 17.87)	1/338	0/82	32.22
NCT03930732	Dupilumab	Placebo	0.33 (0.01, 8.18)	0/469	1/470	32.09
Overall (I-squared = 0.0%, p = 0.548)			1.02 (0.17, 6.22)	3/1232	1/867	100.00

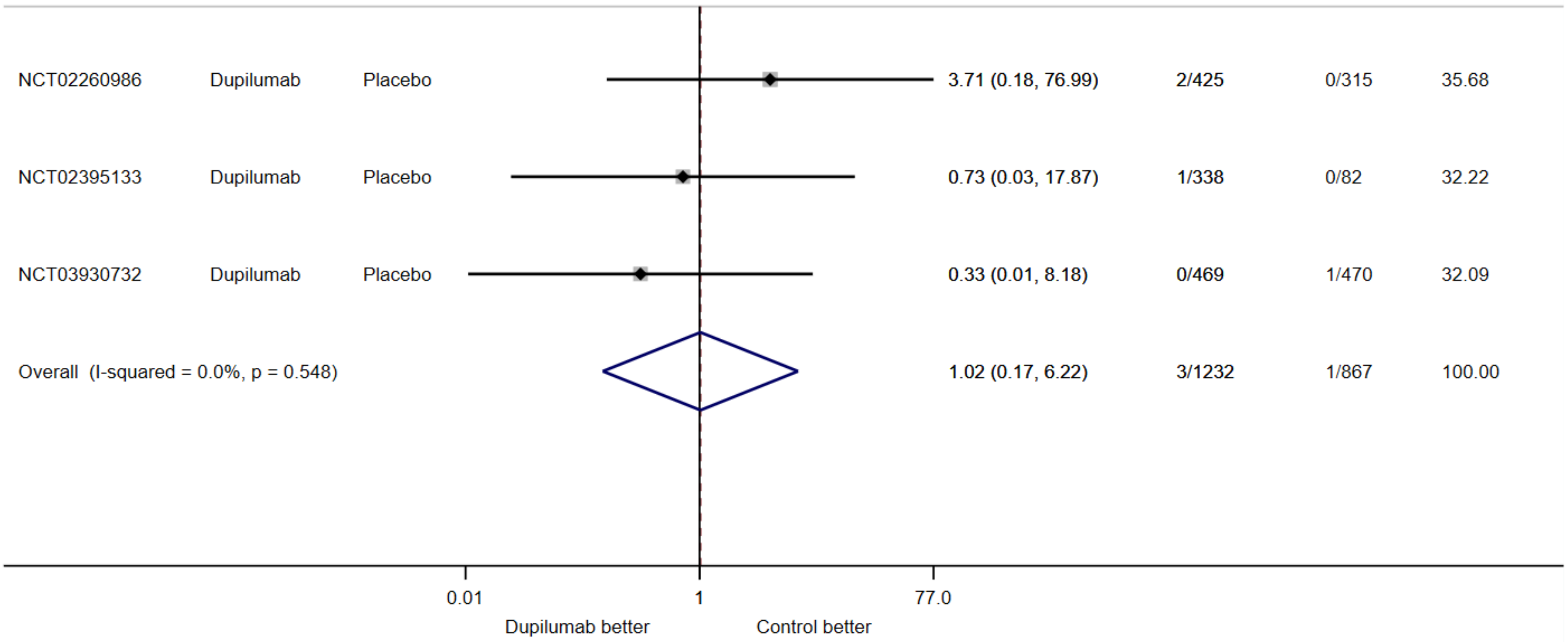

13_Neoplasms Benign, Malignant And Unspecified-Figure S5 Meta-analysis of Dupilumab and Squamous cell carcinoma of skin

Trial	Dupilumab	Control	Risk Ratio (95% CI)	Events, Treatment	Events, Control	% Weight
NCT02260986	Dupilumab	Placebo	0.25 (0.01, 6.05)	0/425	1/315	26.45
NCT02414854	Dupilumab	Placebo	1.00 (0.09, 11.05)	2/1263	1/634	47.01
NCT02755649	Dupilumab	Placebo	1.50 (0.06, 36.52)	1/217	0/108	26.54
Overall (I-squared = 0.0%, p = 0.705)			0.77 (0.15, 3.99)	3/1905	2/1057	100.00

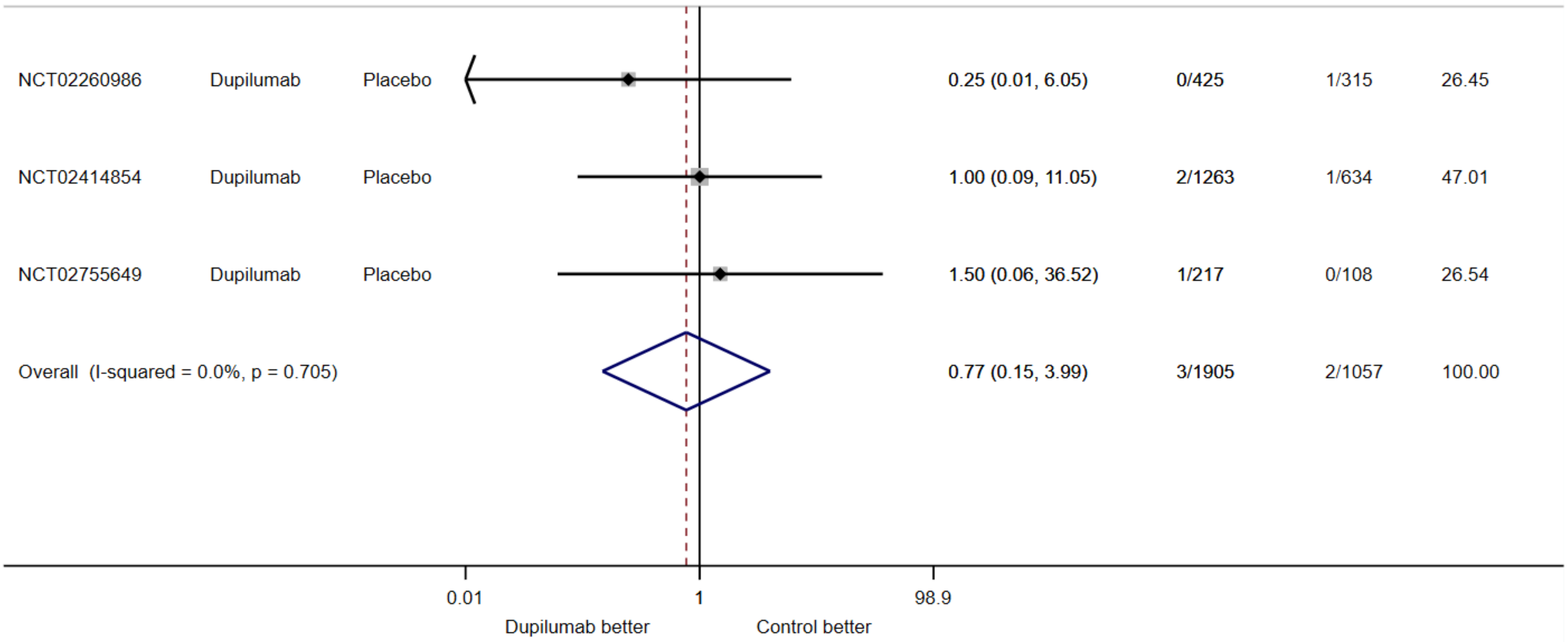

13_Neoplasms Benign, Malignant And Unspecified-Figure S6 Meta-analysis of Dupilumab and Uterine leiomyoma

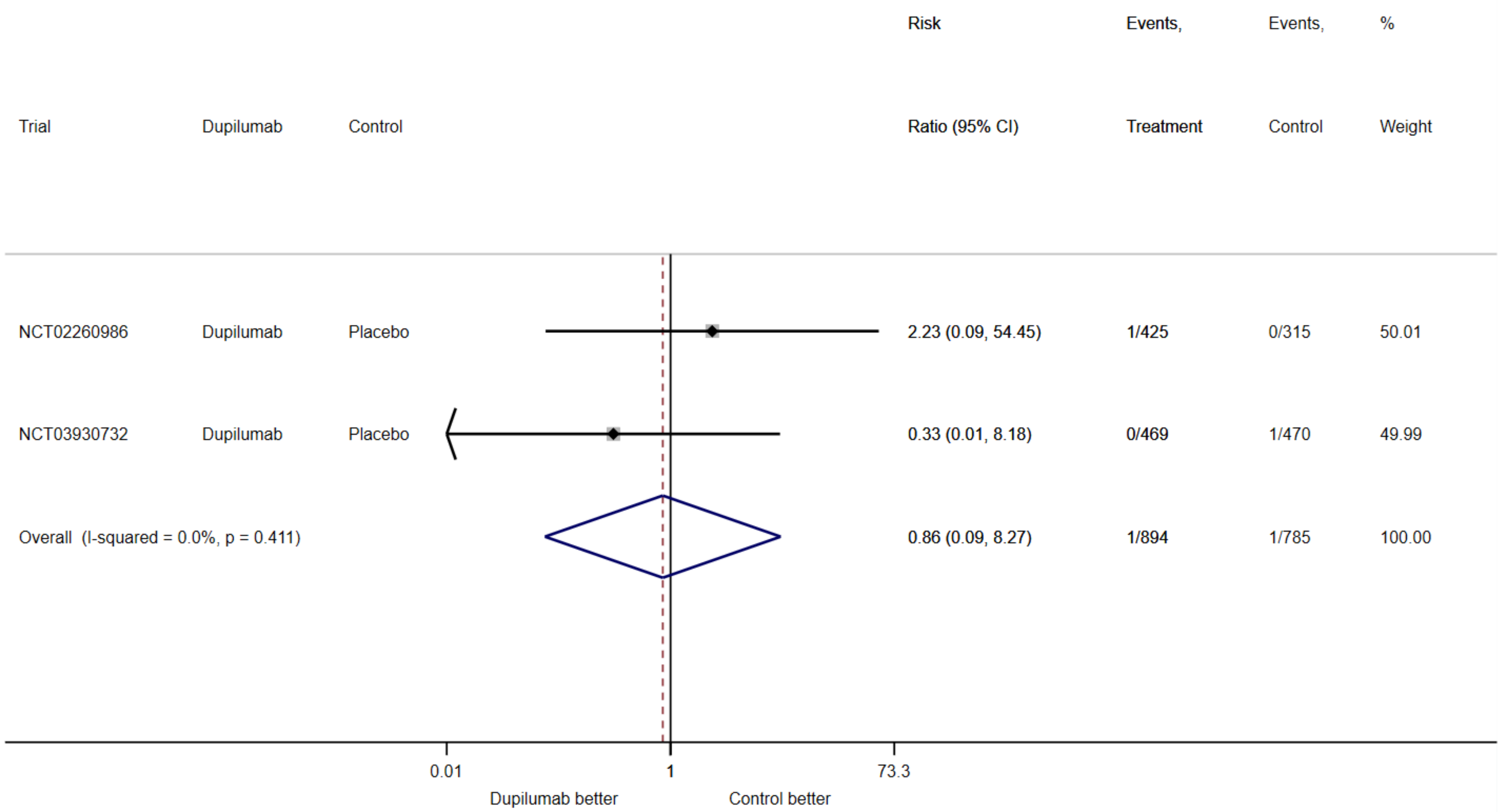

14_Nervous System Disorders-Figure S1 Meta-analysis of Dupilumab and Cerebral infarction

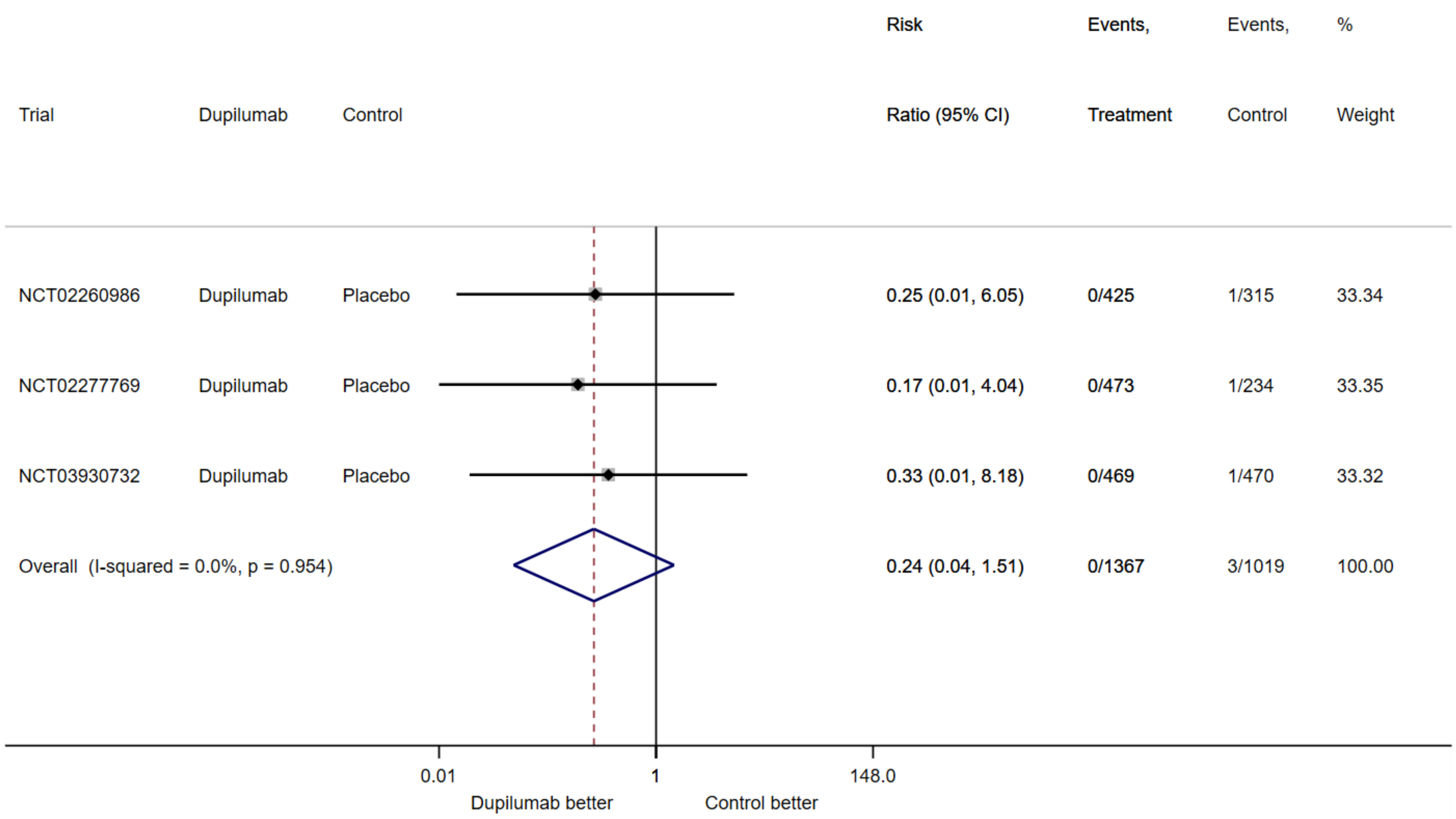

14_Nervous System Disorders-Figure S2 Meta-analysis of Dupilumab and Cerebrovascular accident

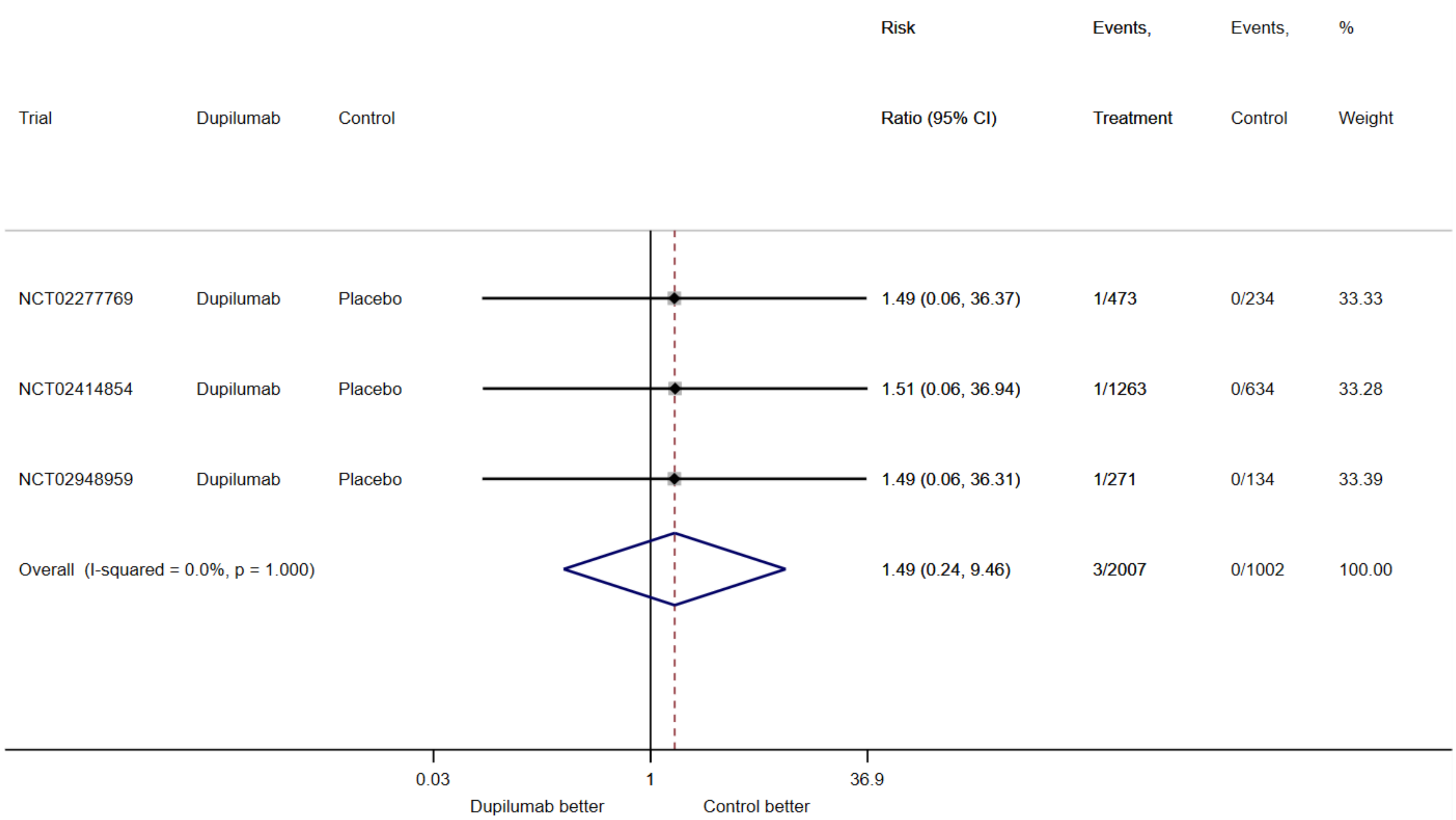

14_Nervous System Disorders-Figure S3 Meta-analysis of Dupilumab and Headache

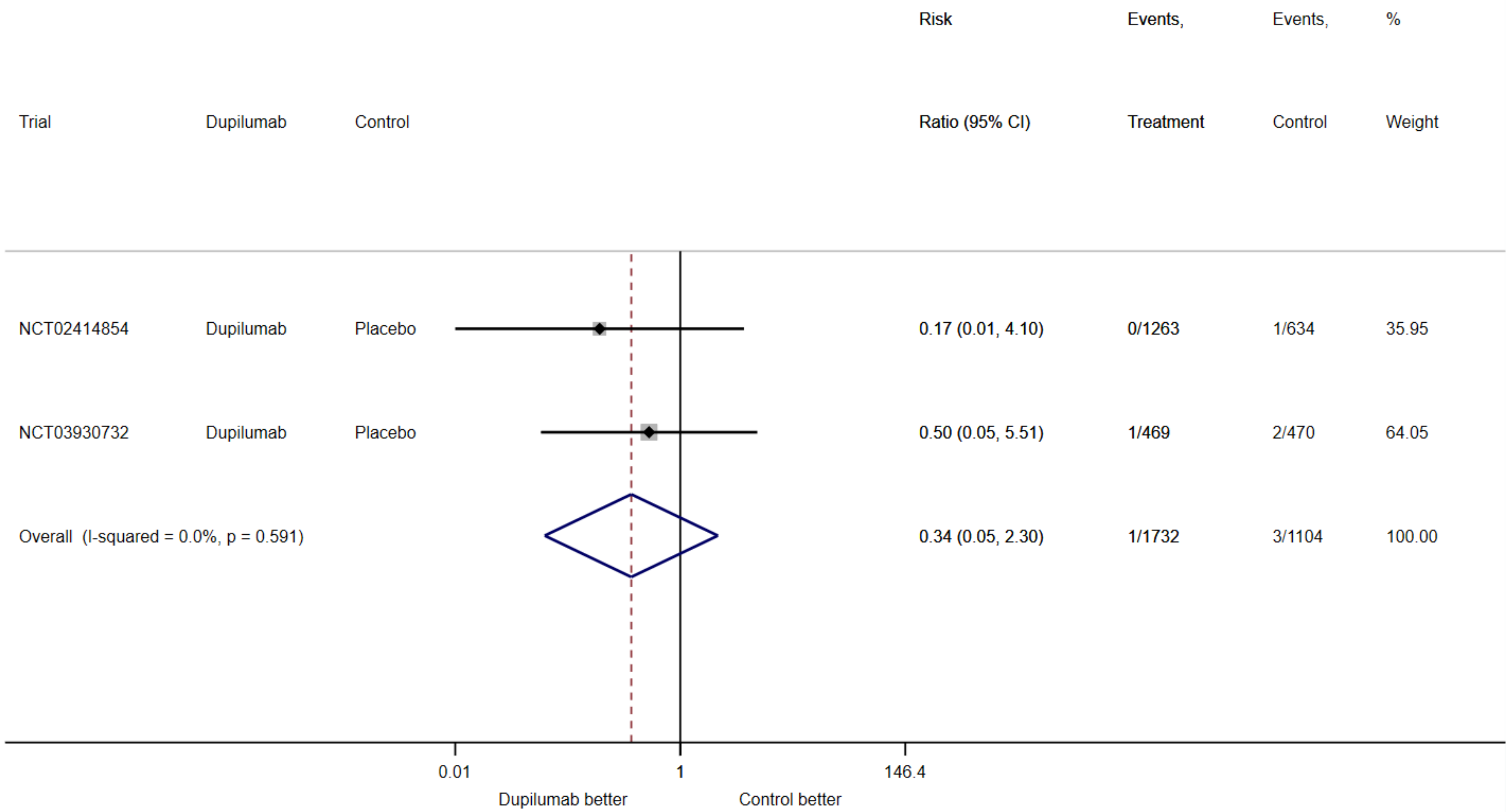

14_Nervous System Disorders-Figure S4 Meta-analysis of Dupilumab and Ischaemic stroke

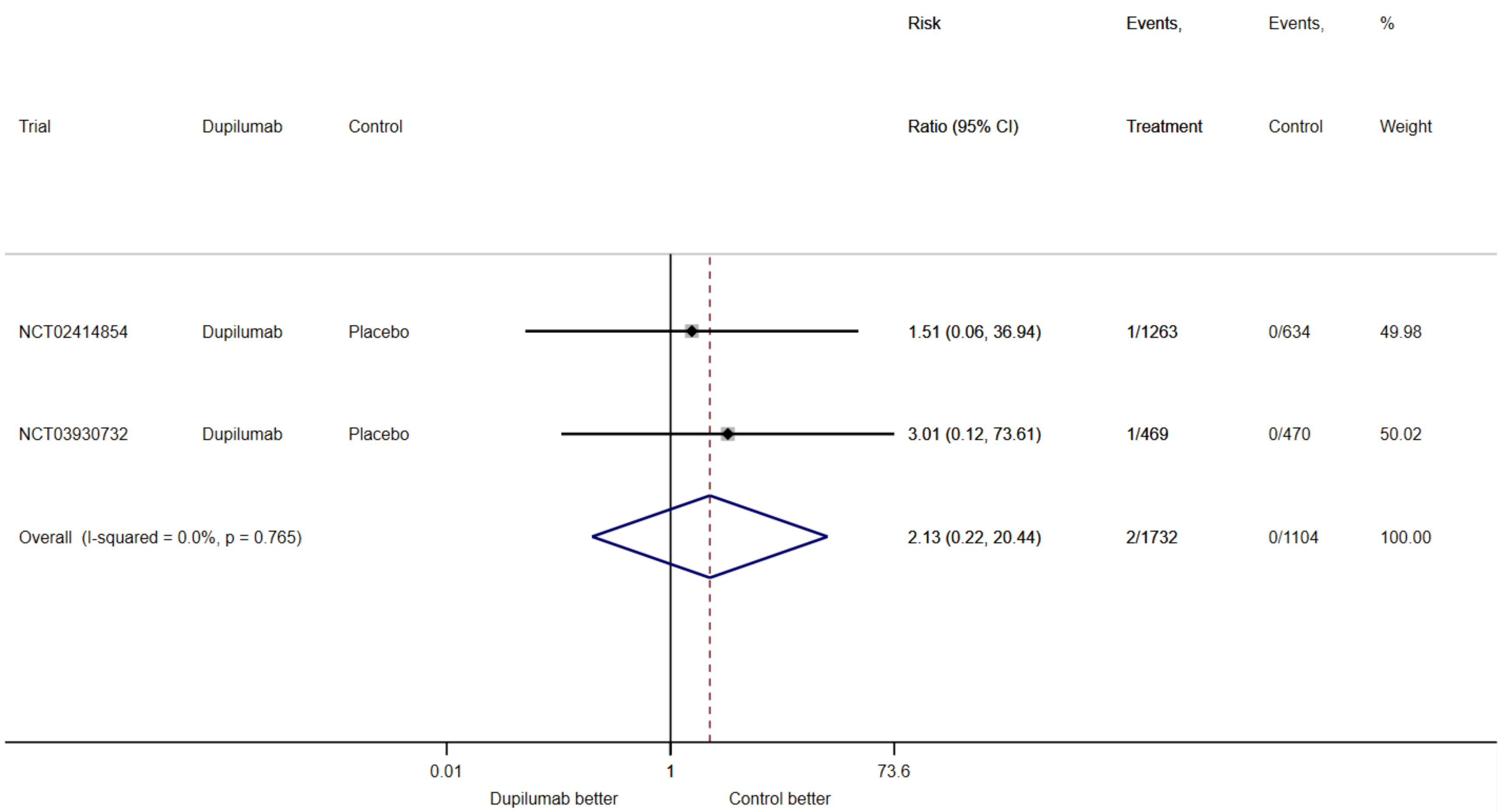

14_Nervous System Disorders-Figure S5 Meta-analysis of Dupilumab and Seizure

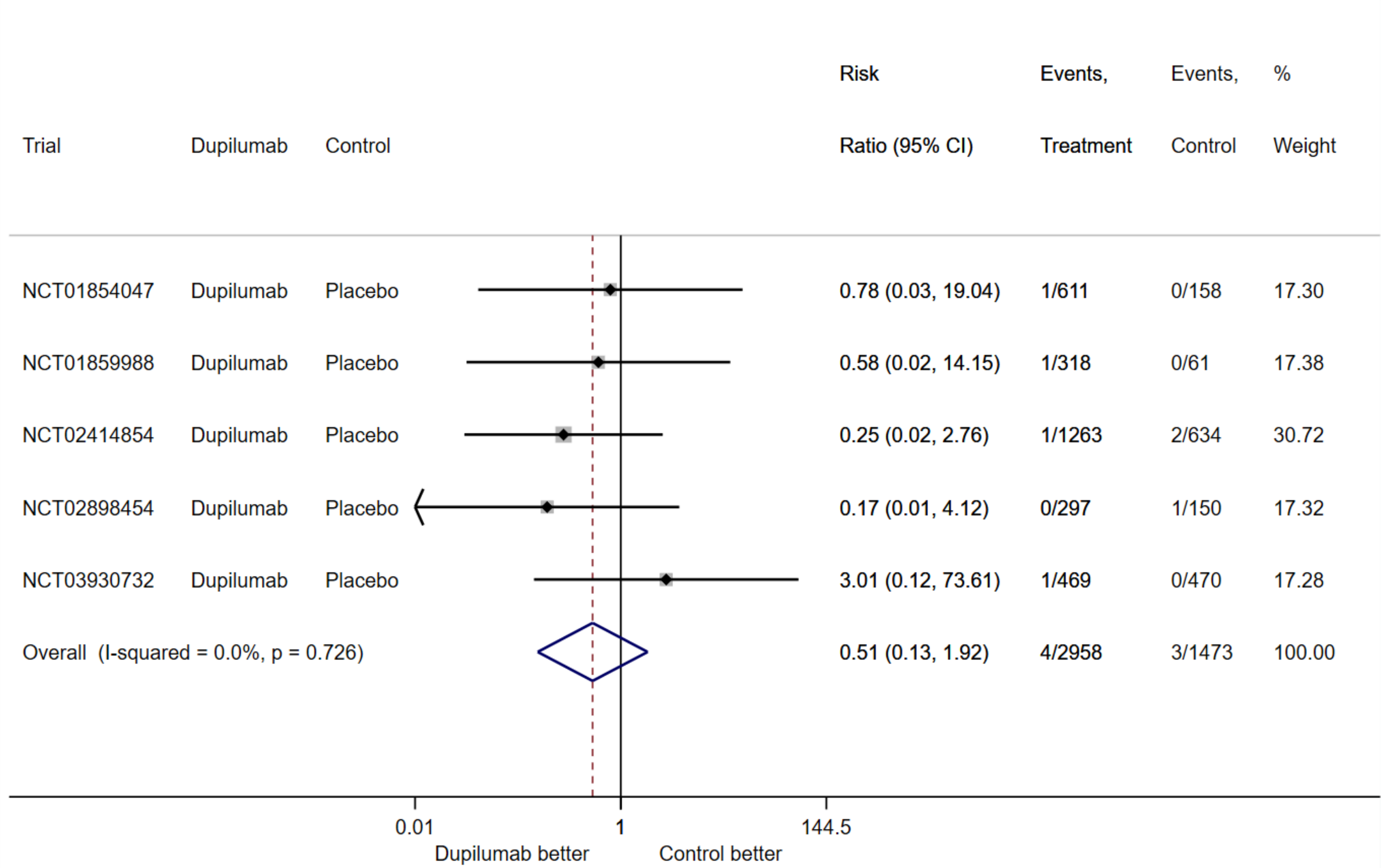

14_Nervous System Disorders-Figure S6 Meta-analysis of Dupilumab and Syncope

Trial	Dupilumab	Control	Risk Ratio (95% CI)	Events, Treatment	Events, Control	% Weight
NCT01854047	Dupilumab	Placebo	1.82 (0.09, 35.03)	3/611	0/158	36.84
NCT02277769	Dupilumab	Placebo	1.49 (0.06, 36.37)	1/473	0/234	31.54
NCT03633617	Dupilumab	Placebo	0.19 (0.01, 4.70)	0/203	1/117	31.62
Overall (I-squared = 0.0%, p = 0.549)			0.84 (0.14, 5.05)	4/1287	1/509	100.00

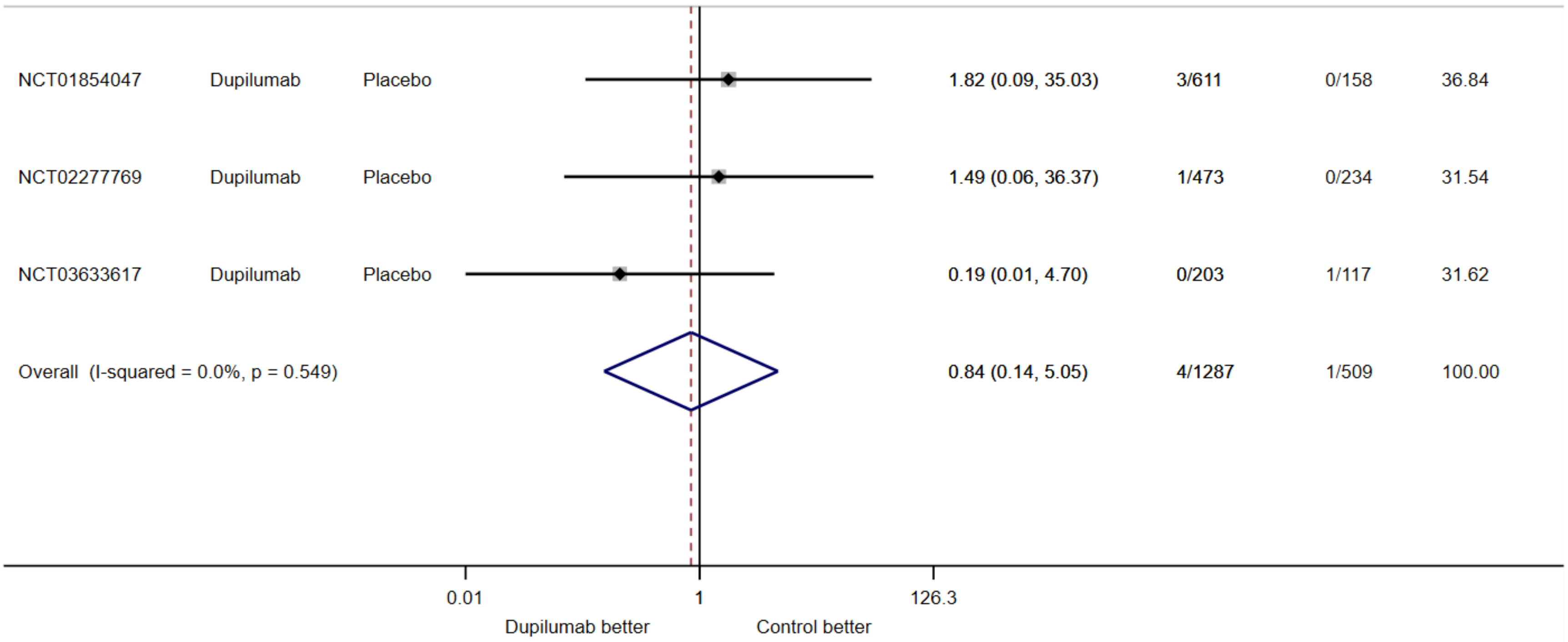

15_Pregnancy, Puerperium And Perinatal Conditions-Figure S1 Meta-analysis of Dupilumab and Abortion spontaneous

Trial	Dupilumab	Control	Risk Ratio (95% CI)	Events, Treatment	Events, Control	% Weight
NCT01854047	Dupilumab	Placebo	0.78 (0.03, 19.04)	1/611	0/158	50.05
NCT02414854	Dupilumab	Placebo	1.51 (0.06, 36.94)	1/1263	0/634	49.95
Overall (I-squared = 0.0%, p = 0.775)			1.08 (0.11, 10.39)	2/1874	0/792	100.00

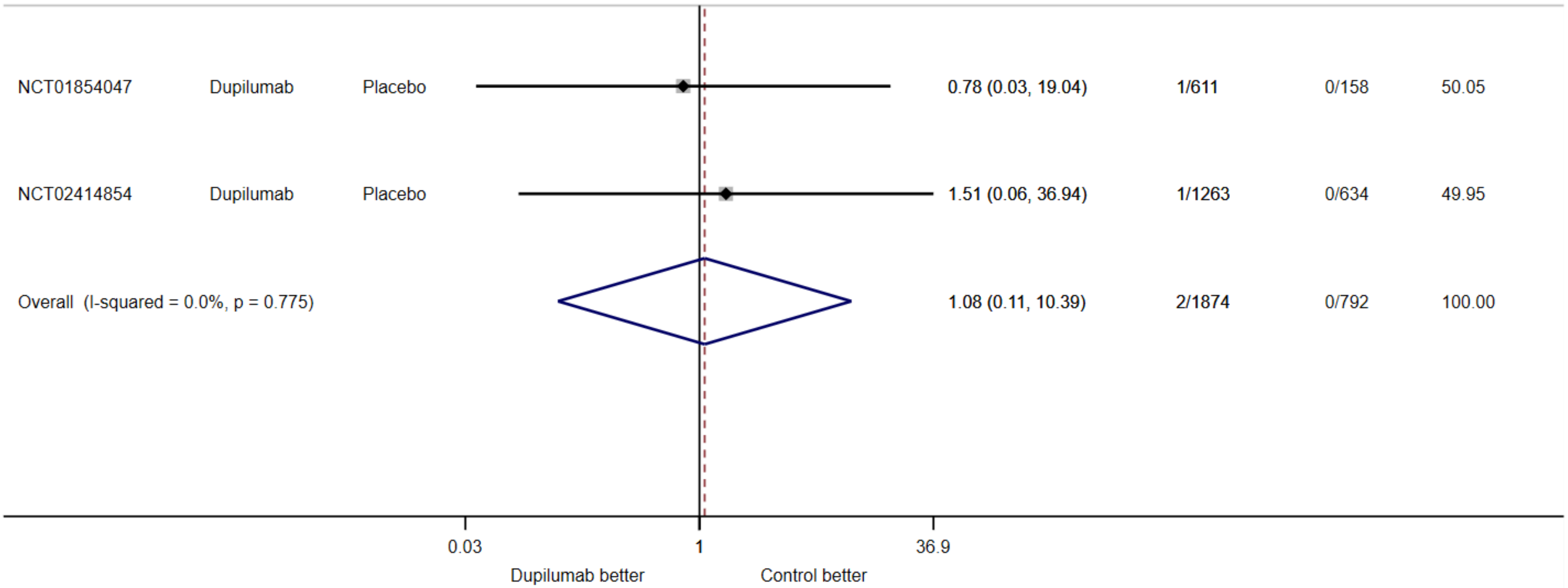

15_Pregnancy, Puerperium And Perinatal Conditions-Figure S2 Meta-analysis of Dupilumab and Pregnancy

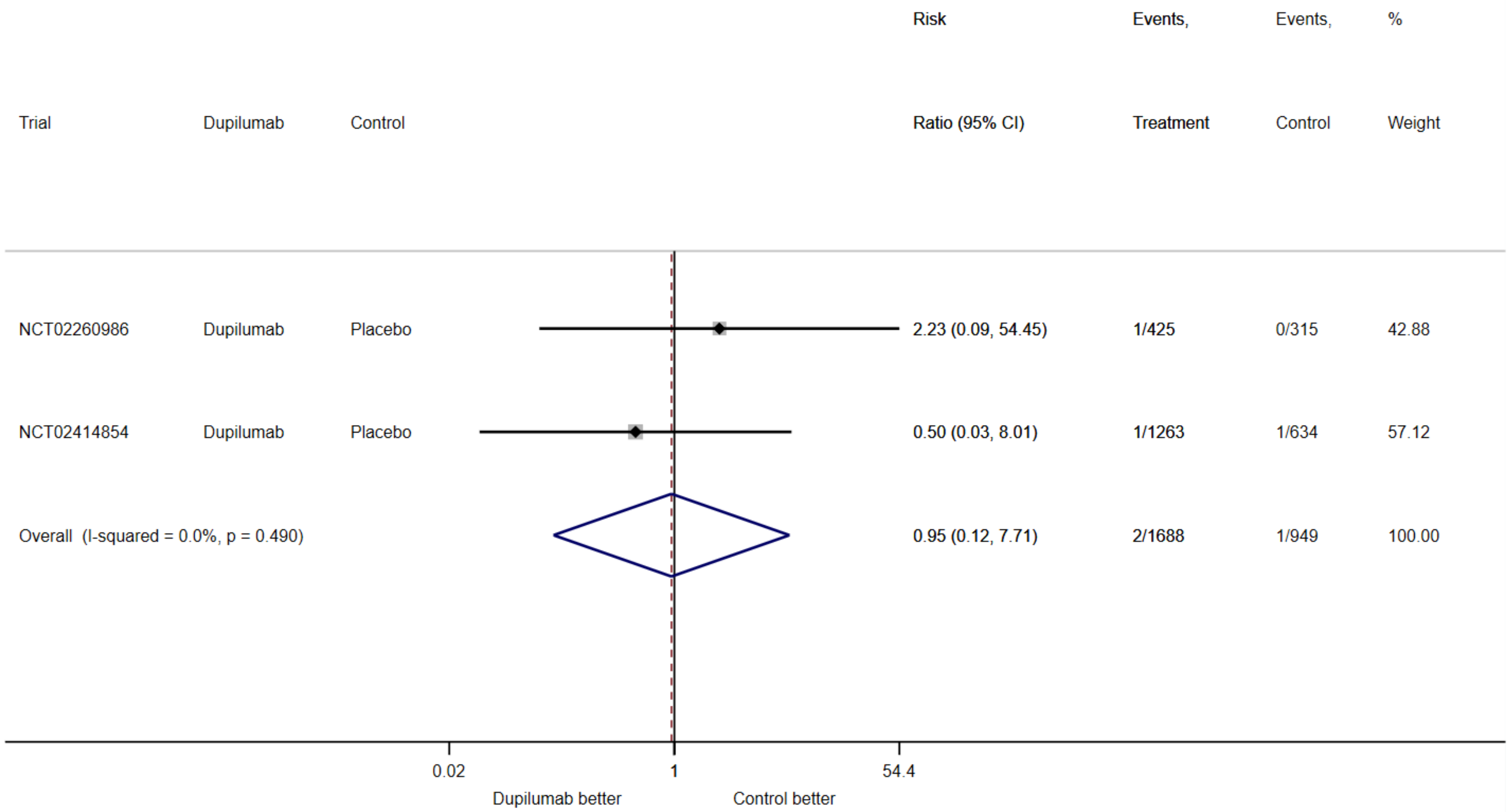

16_Psychiatric Disorders-Figure S1 Meta-analysis of Dupilumab and Anxiety

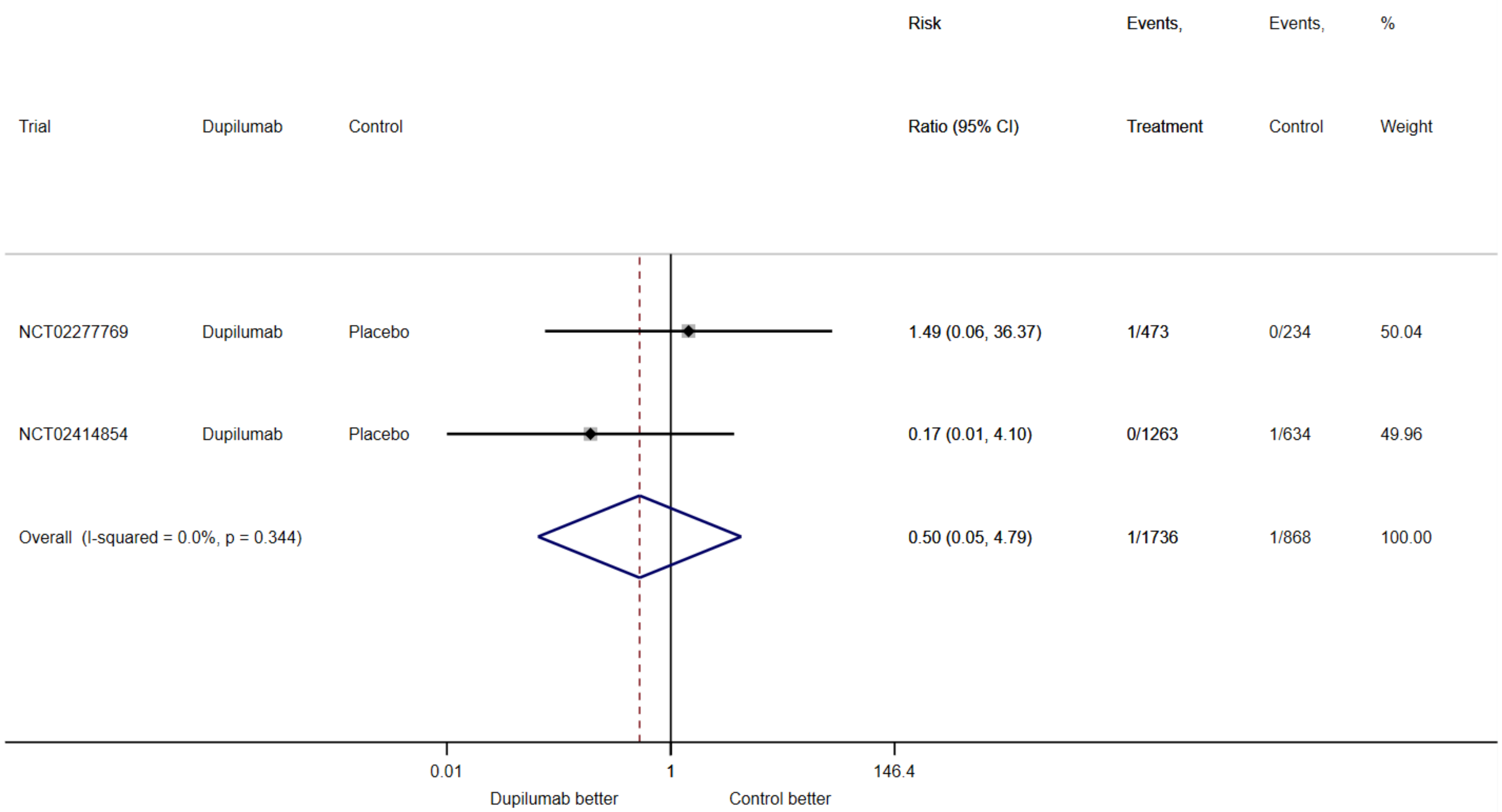

16_Psychiatric Disorders-Figure S2 Meta-analysis of Dupilumab and Completed suicide

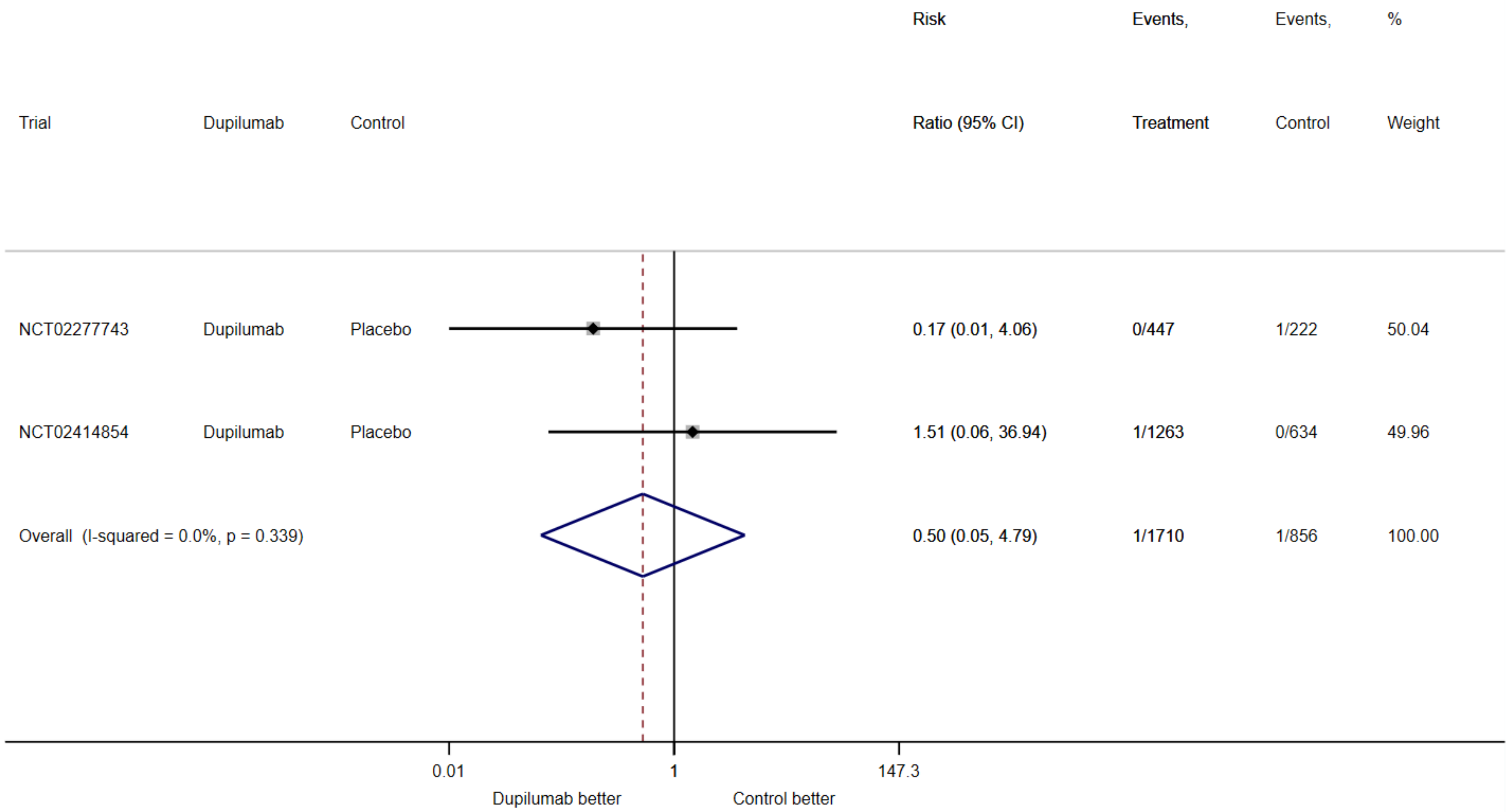

16_Psychiatric Disorders-Figure S3 Meta-analysis of Dupilumab and Depression

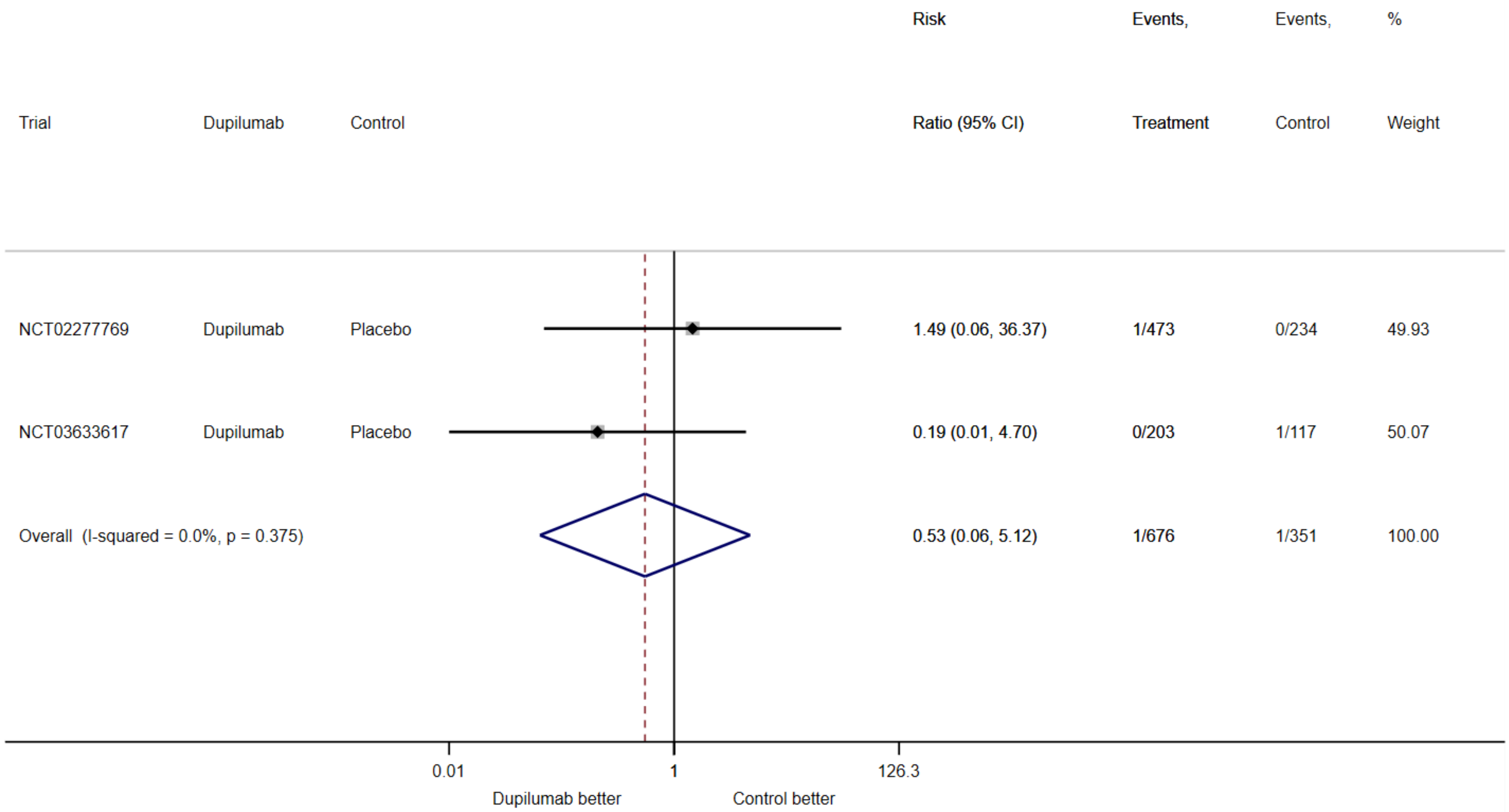

16_Psychiatric Disorders-Figure S4 Meta-analysis of Dupilumab and Mental status changes

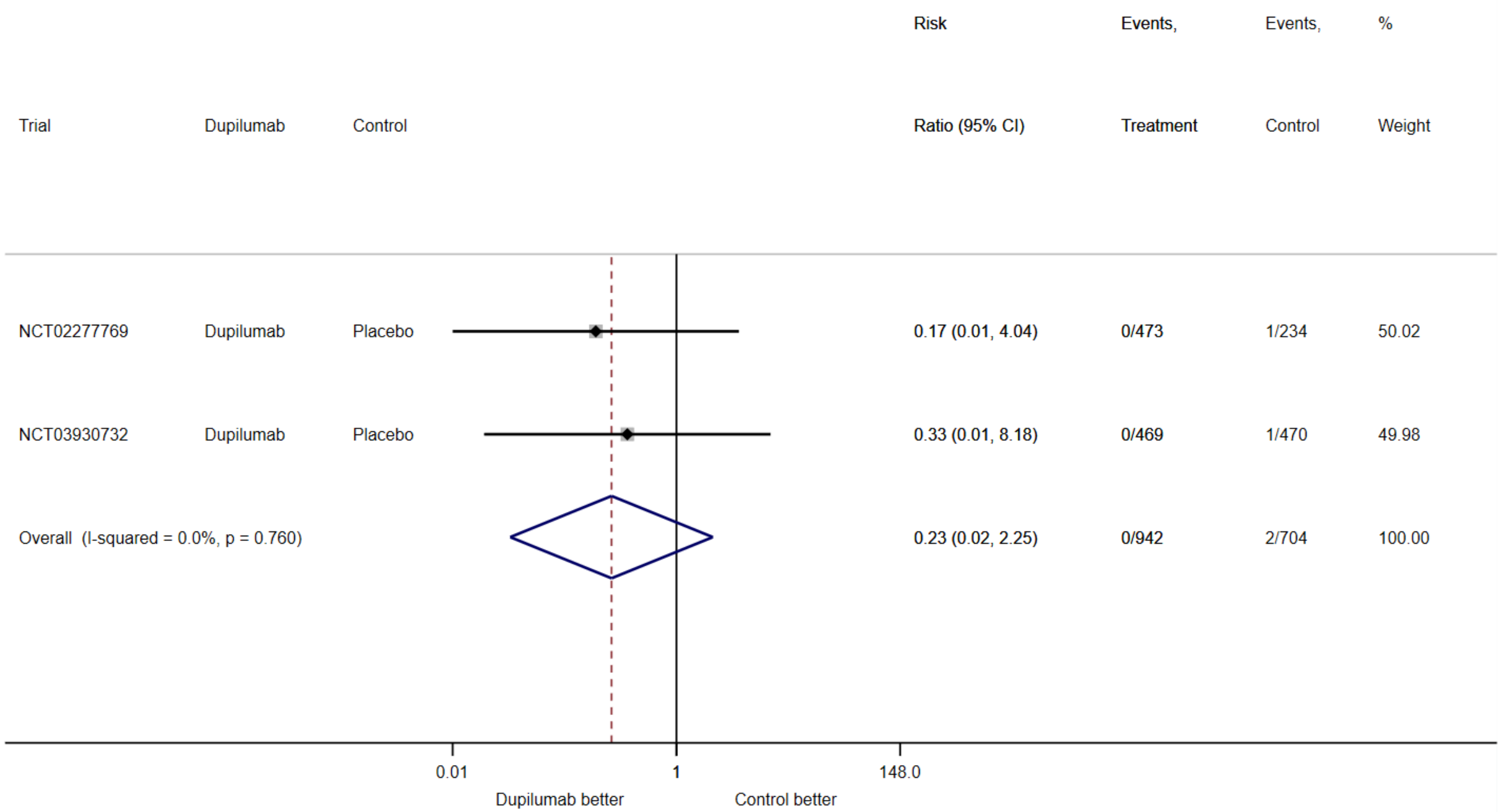

16_Psychiatric Disorders-Figure S5 Meta-analysis of Dupilumab and Psychotic disorder

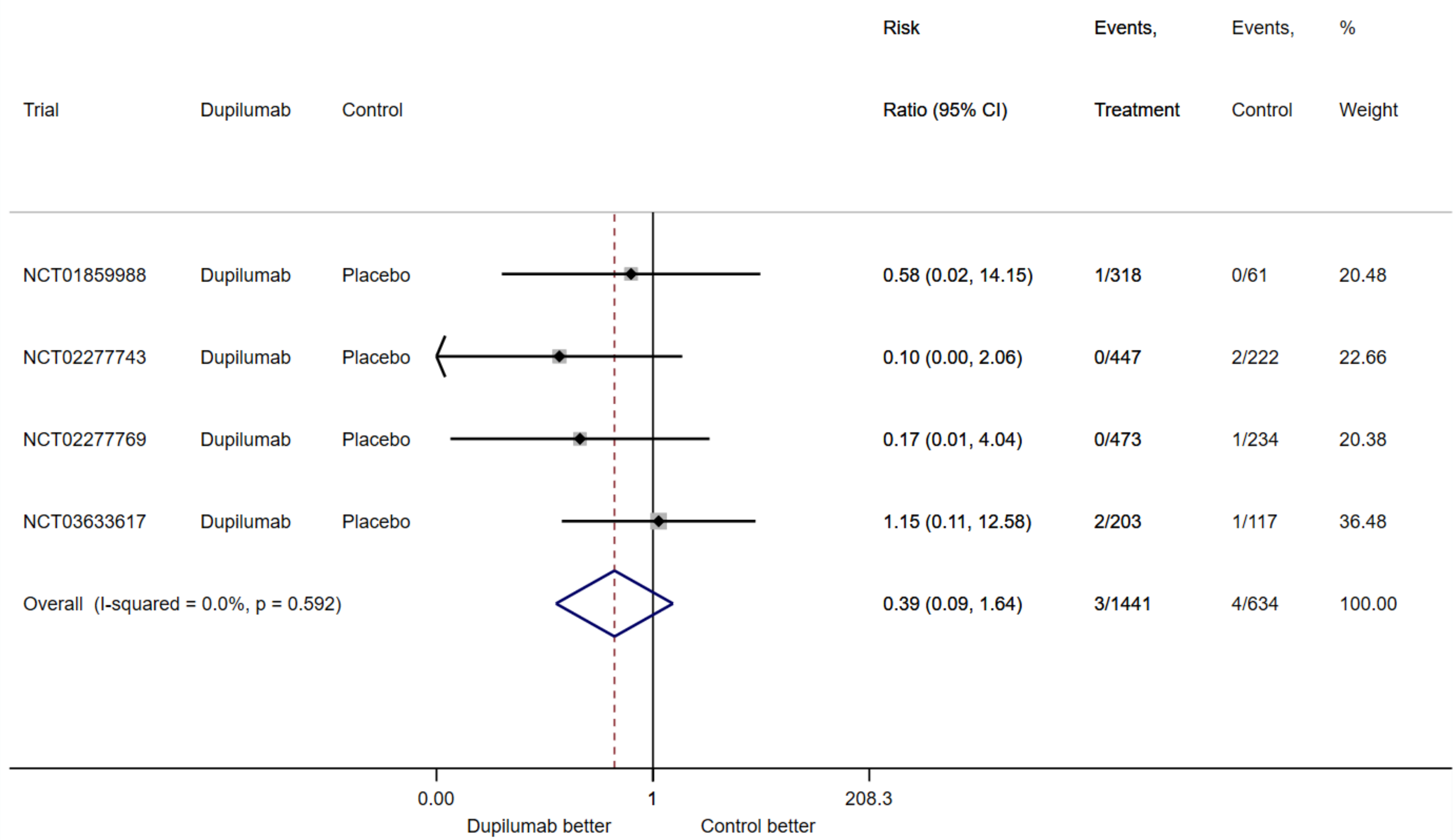

16_Psychiatric Disorders-Figure S6 Meta-analysis of Dupilumab and Suicidal ideation

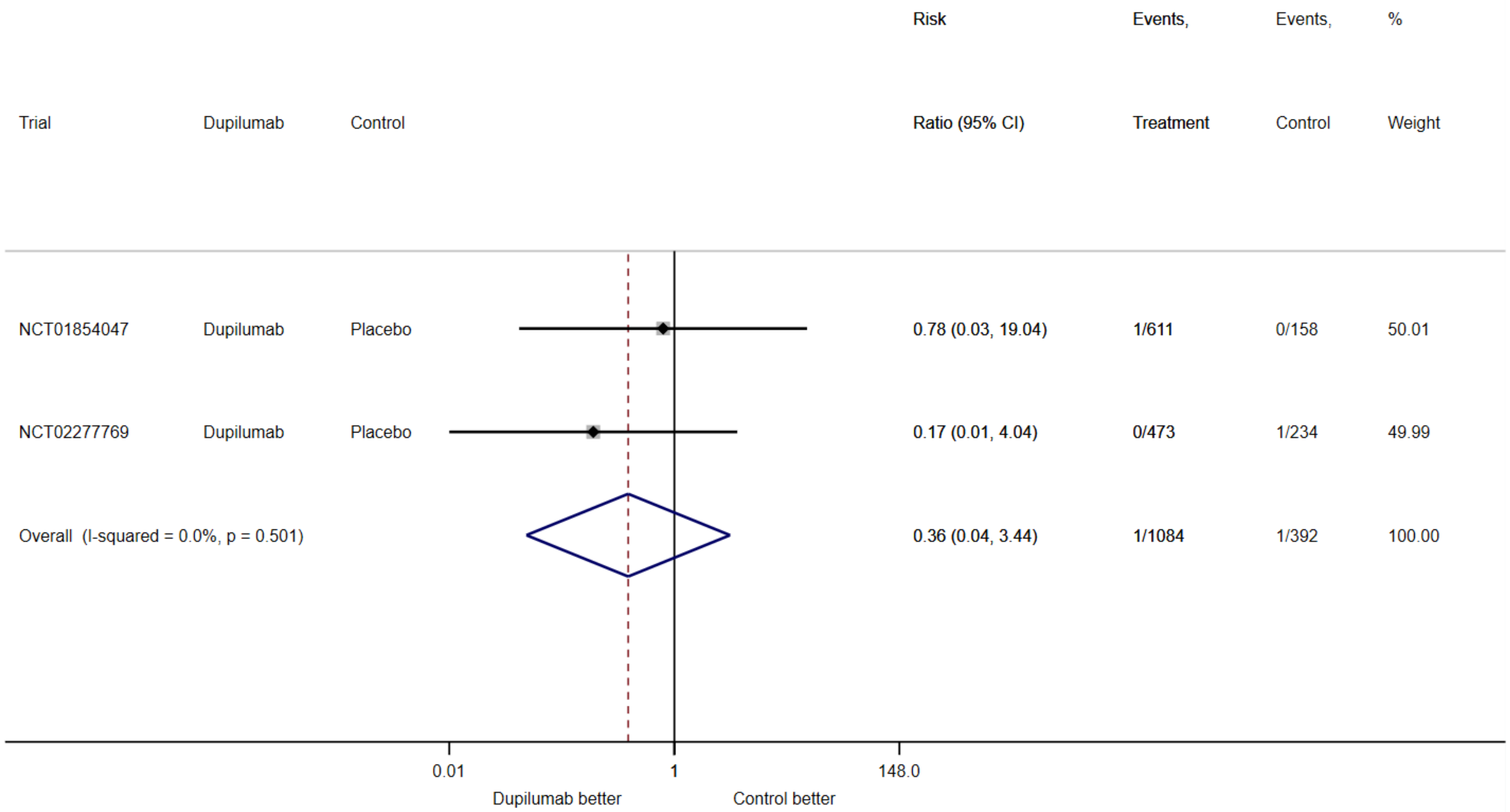

16_Psychiatric Disorders-Figure S7 Meta-analysis of Dupilumab and Suicide attempt

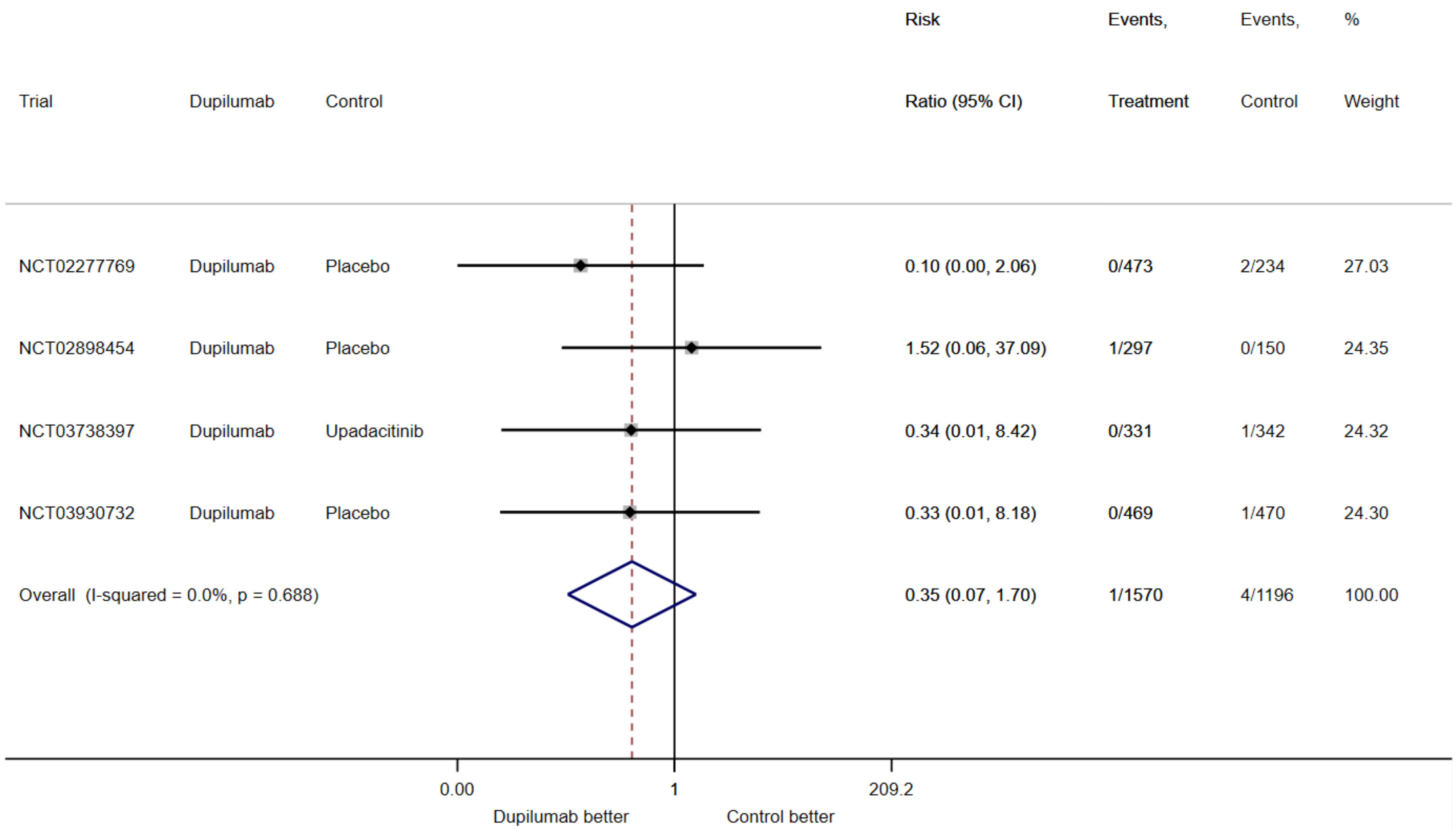

17_Renal And Urinary Disorders-Figure S1 Meta-analysis of Dupilumab and Acute kidney injury

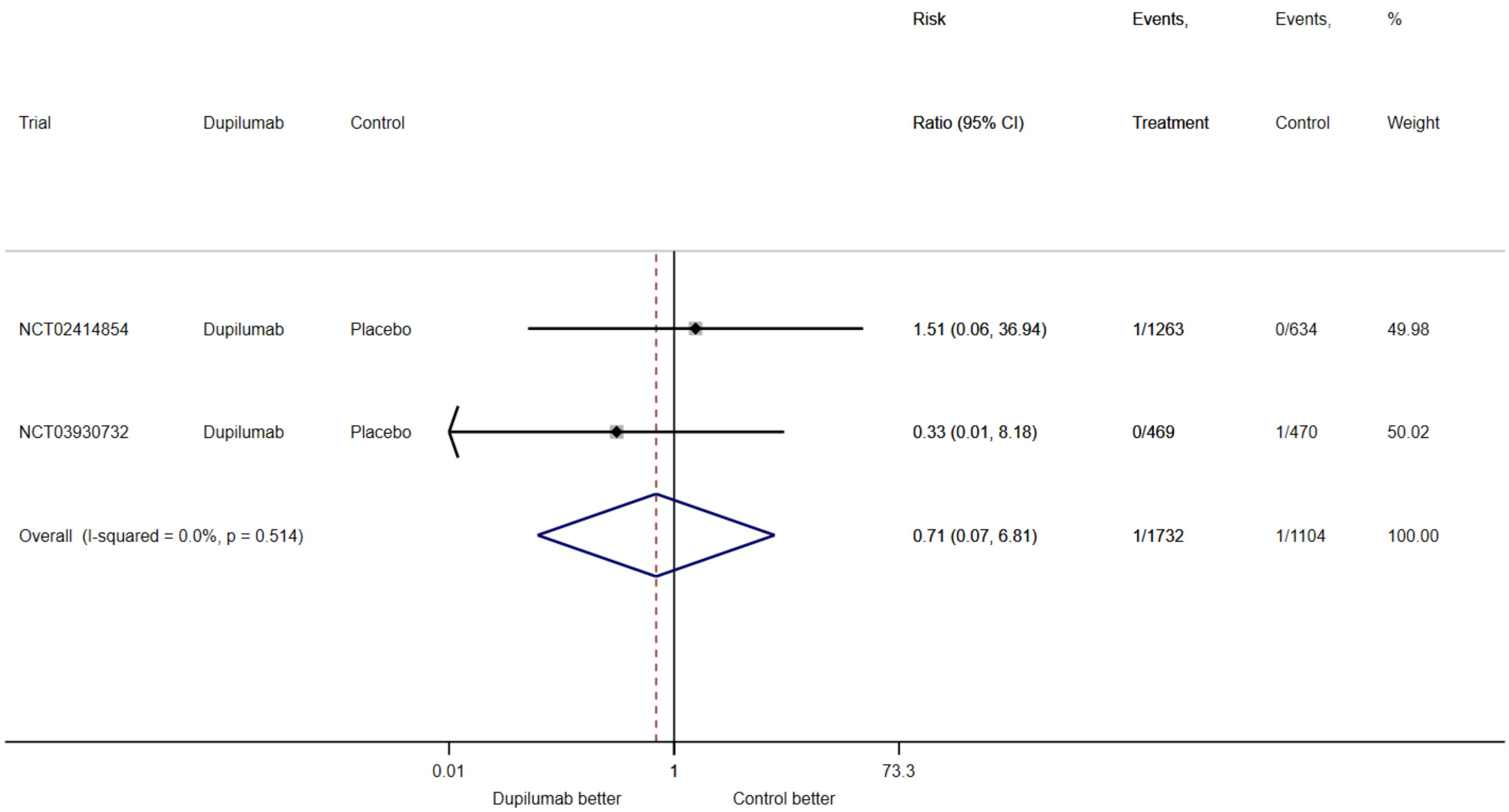

18_Reproductive System And Breast Disorders-Figure S1 Meta-analysis of Dupilumab and Ovarian cyst

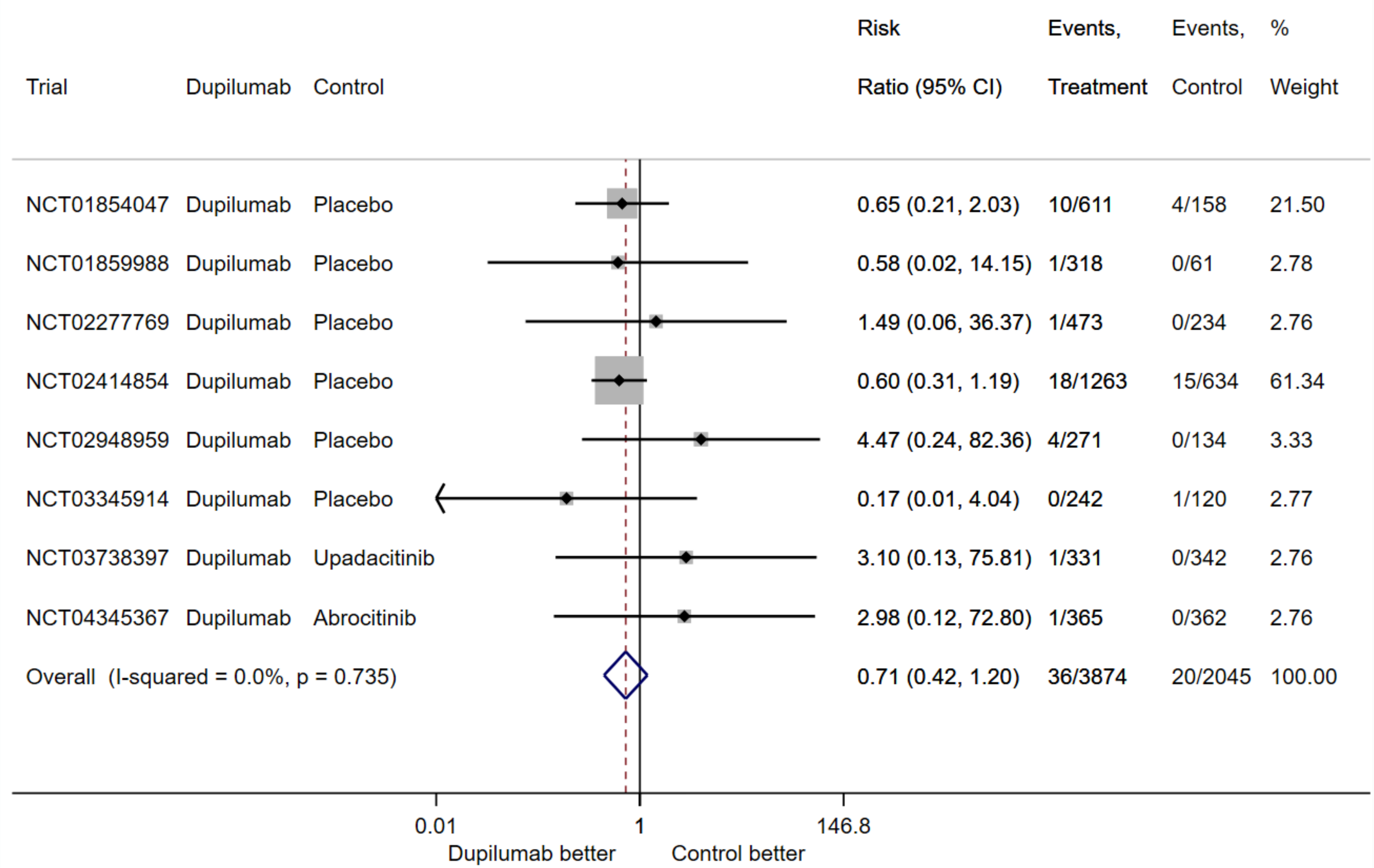

19_Respiratory, Thoracic And Mediastinal Disorders-Figure S1 Meta-analysis of Dupilumab and Asthma

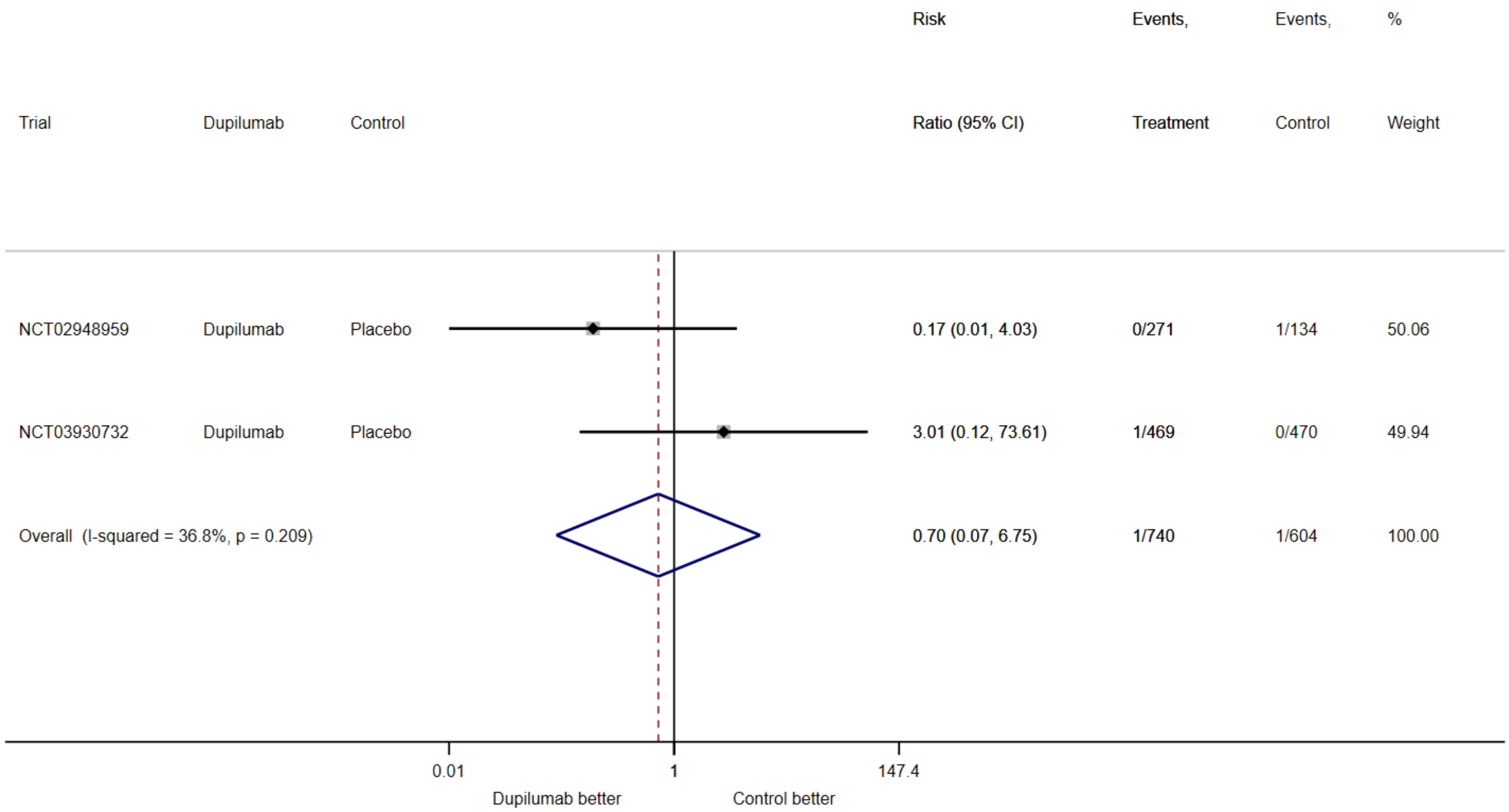

19_Respiratory, Thoracic And Mediastinal Disorders-Figure S2 Meta-analysis of Dupilumab and Atelectasis

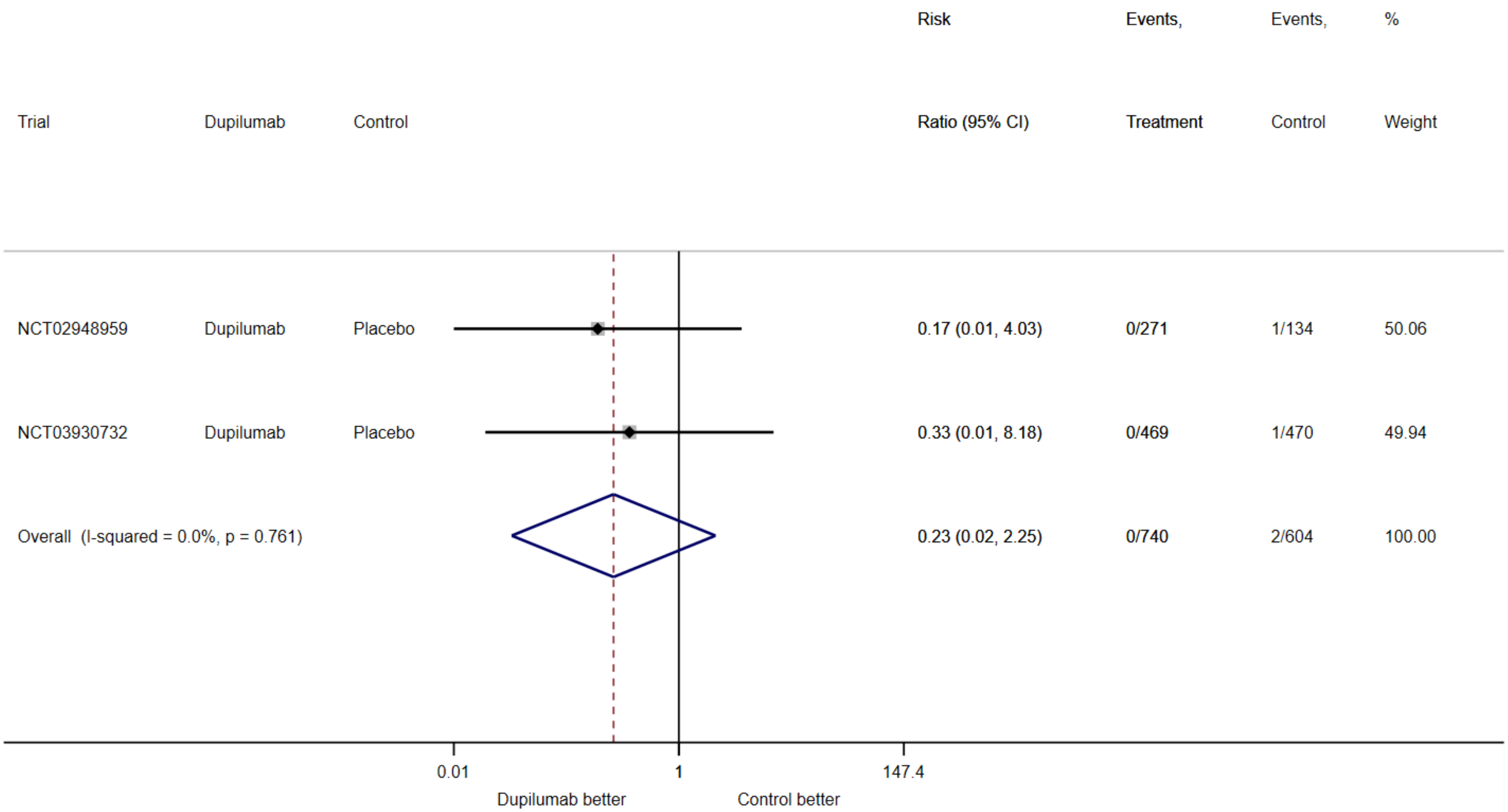

19_Respiratory, Thoracic And Mediastinal Disorders-Figure S3 Meta-analysis of Dupilumab and Bronchospasm

Trial	Dupilumab	Control	Risk Ratio (95% CI)	Events, Treatment	Events, Control	% Weight
NCT02414854	Dupilumab	Placebo	**1.51 (0.06, 36.94)**	**1/1263**	0/634	49.97
NCT03720470	Dupilumab	Placebo/PF-04965842	**0.82 (0.03, 20.00)**	**0/242**	1/595	50.03
Overall (I-squared = 0.0%, p = 0.791)			**1.11 (0.12, 10.65)**	**1/1505**	1/1229	100.00

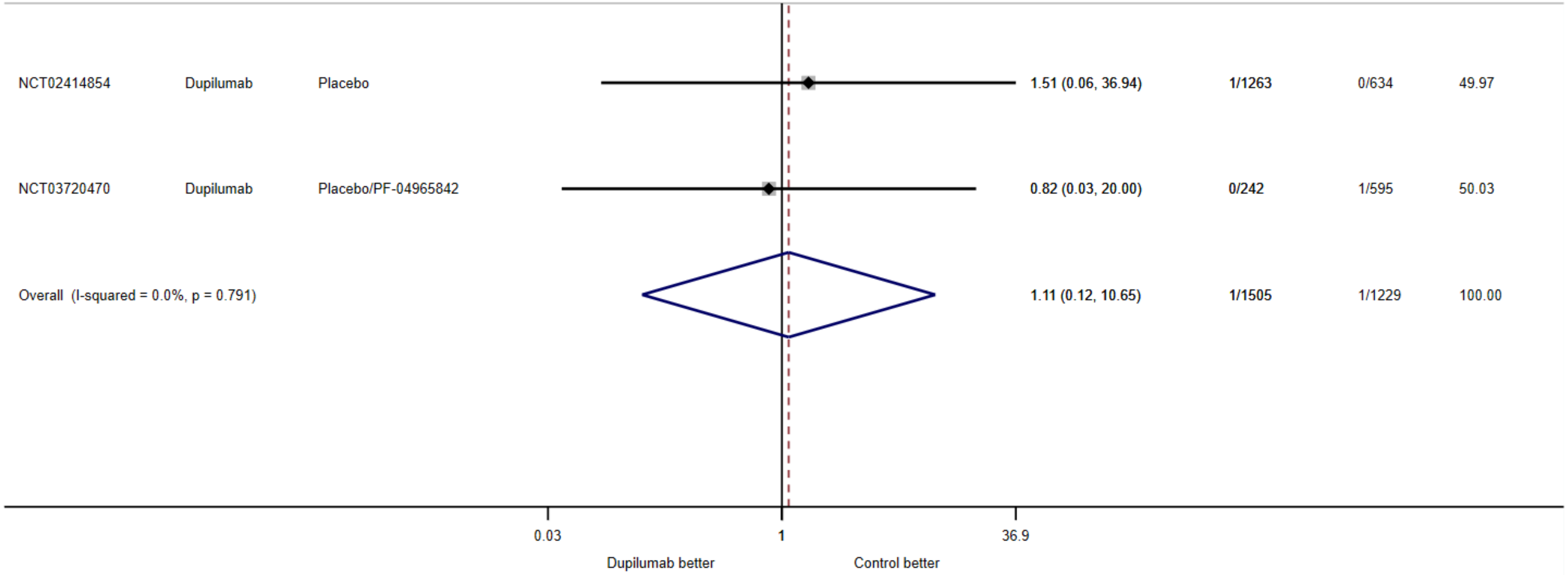

19_Respiratory, Thoracic And Mediastinal Disorders-Figure S4 Meta-analysis of Dupilumab and Dyspnoea

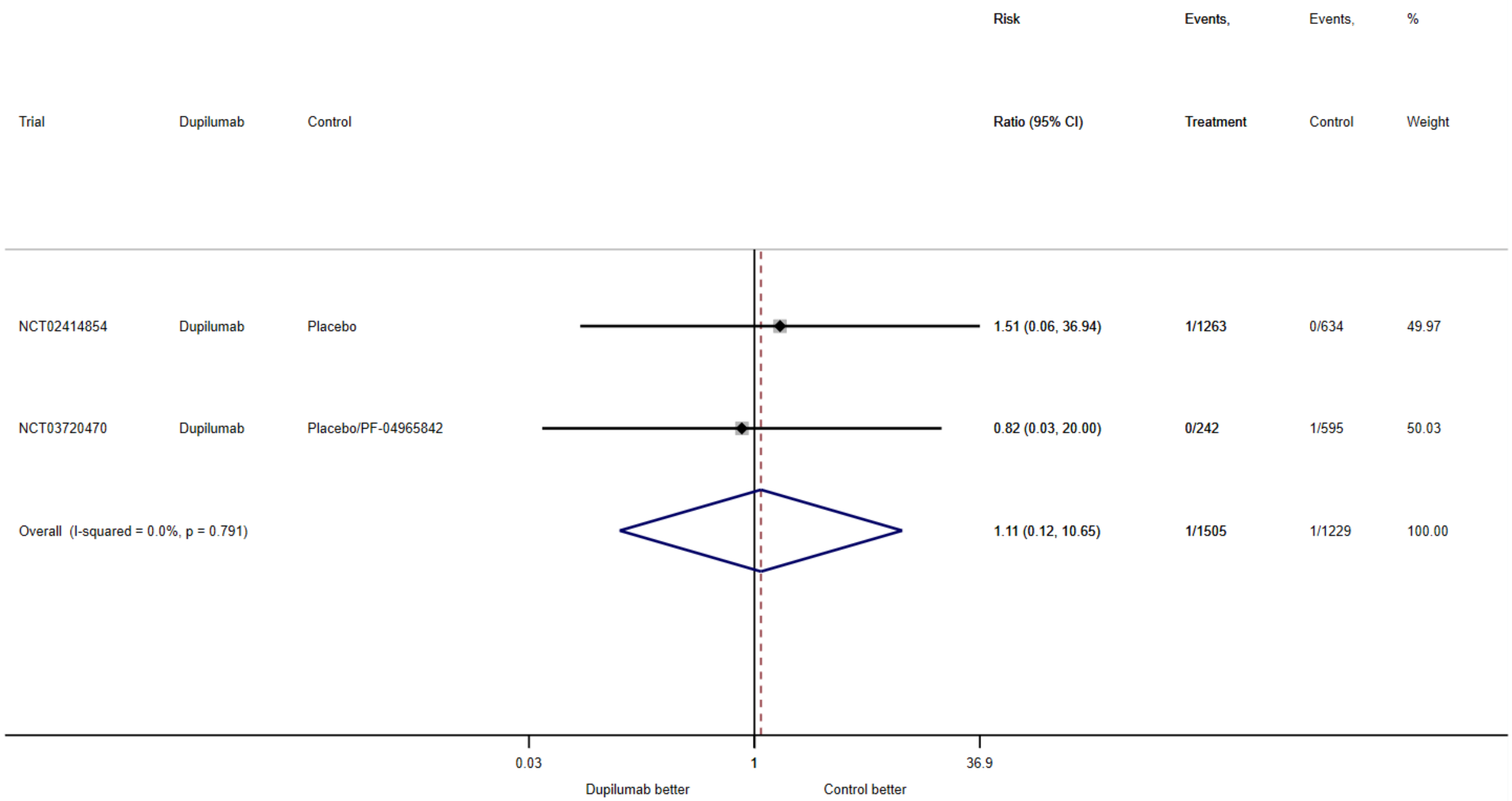

19_Respiratory, Thoracic And Mediastinal Disorders-Figure S5 Meta-analysis of Dupilumab and Interstitial lung disease

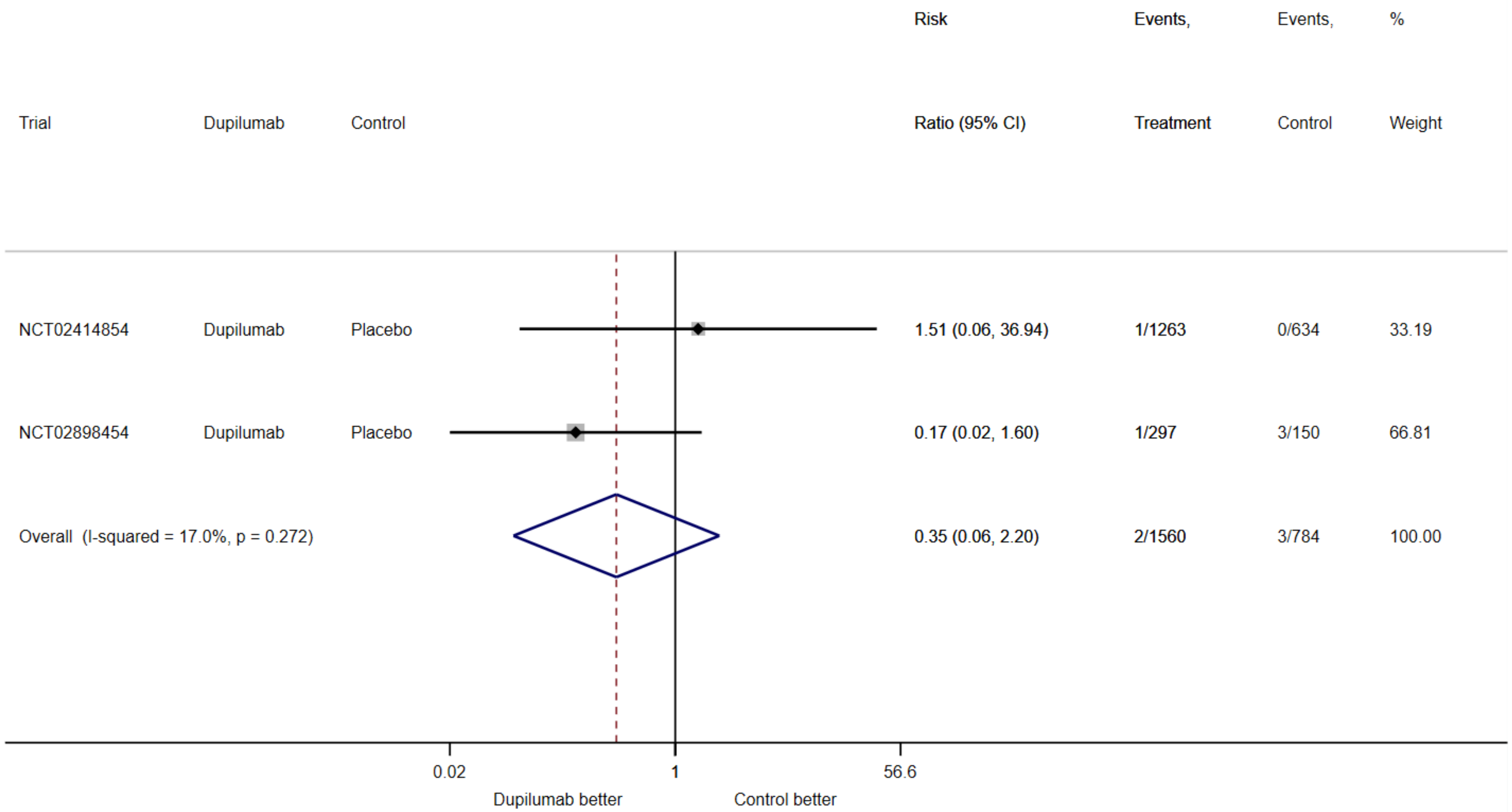

19_Respiratory, Thoracic And Mediastinal Disorders-Figure S6 Meta-analysis of Dupilumab and Nasal polyps

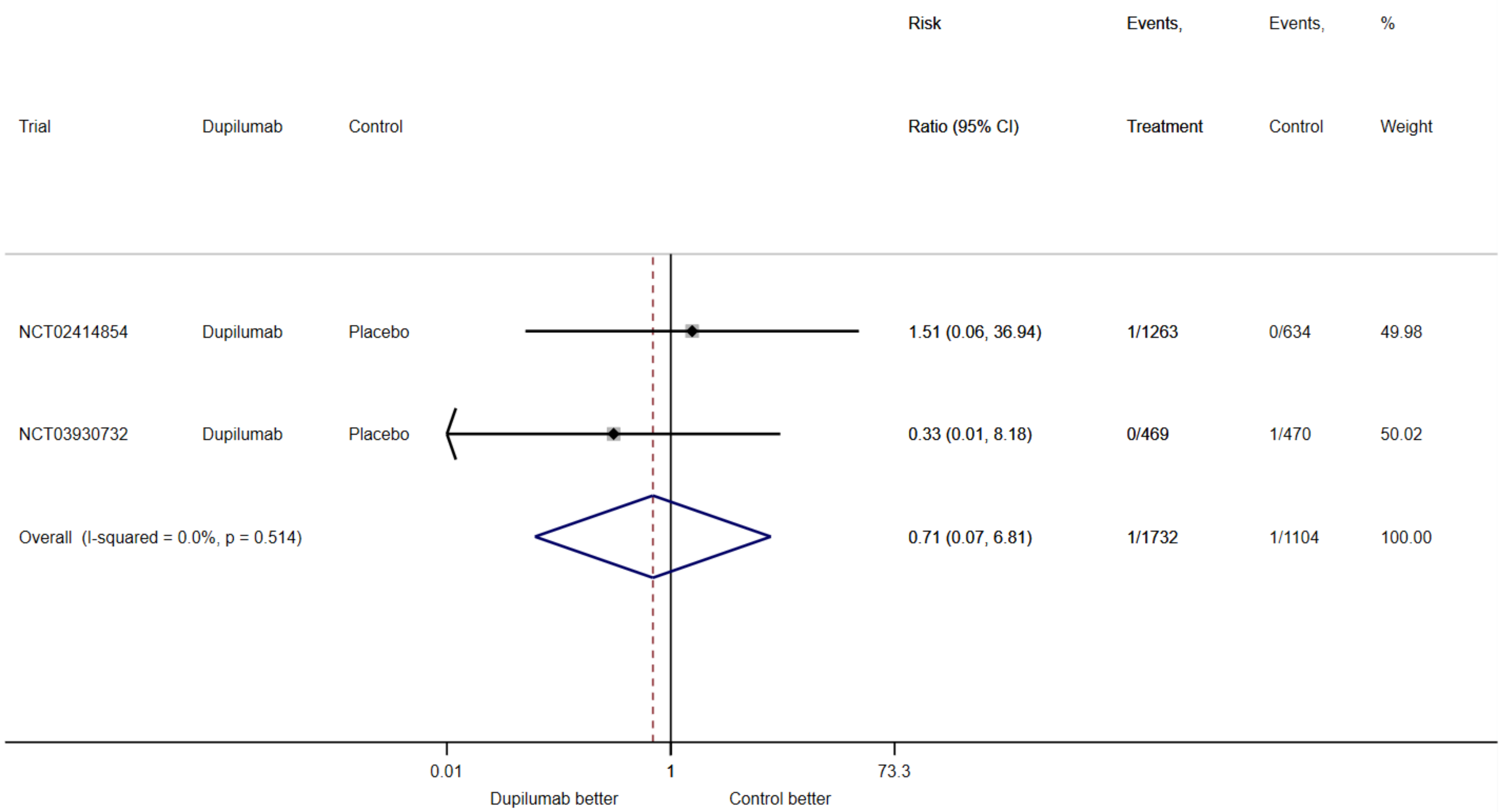

19_Respiratory, Thoracic And Mediastinal Disorders-Figure S7 Meta-analysis of Dupilumab and Pneumothorax spontaneous

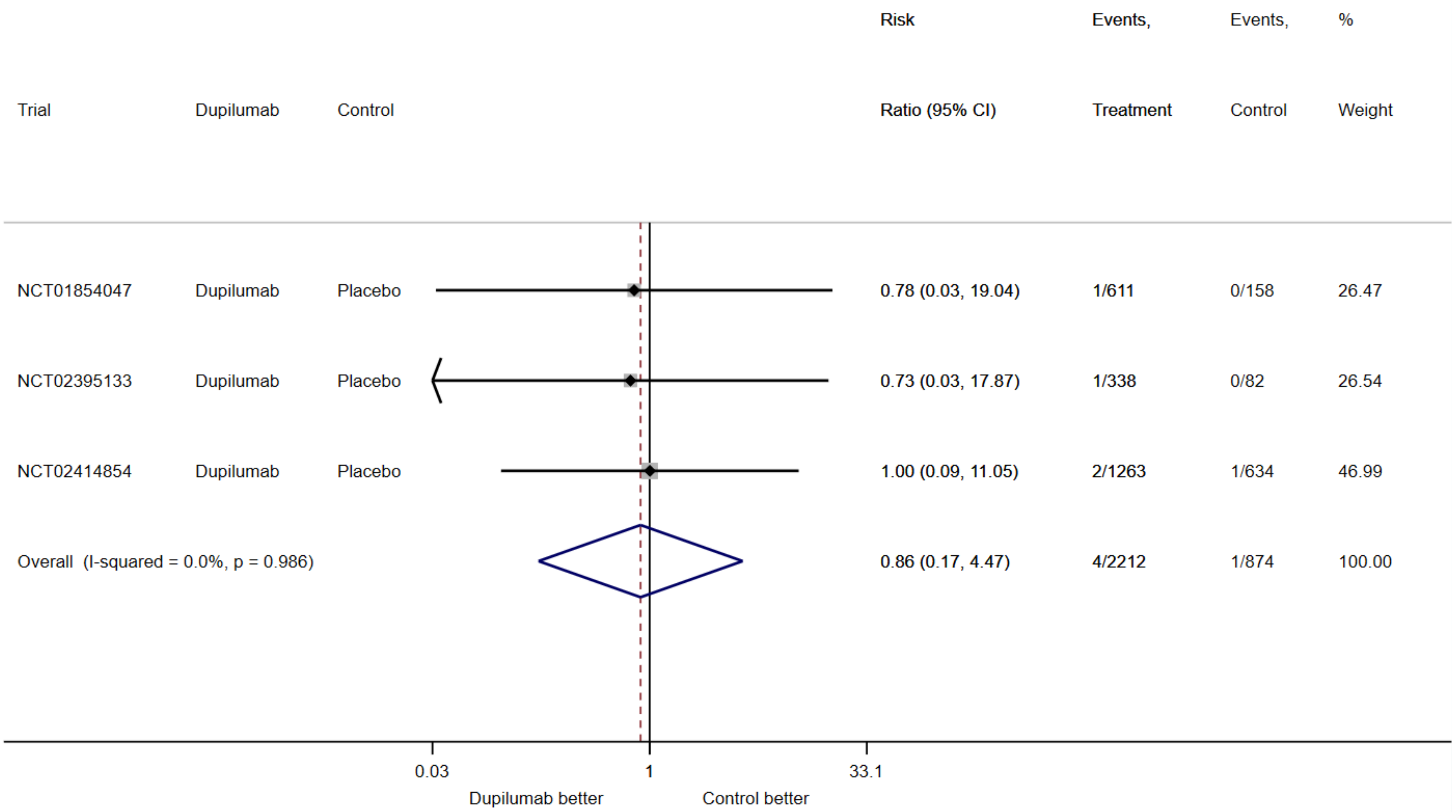

19_Respiratory, Thoracic And Mediastinal Disorders-Figure S8 Meta-analysis of Dupilumab and Pulmonary embolism

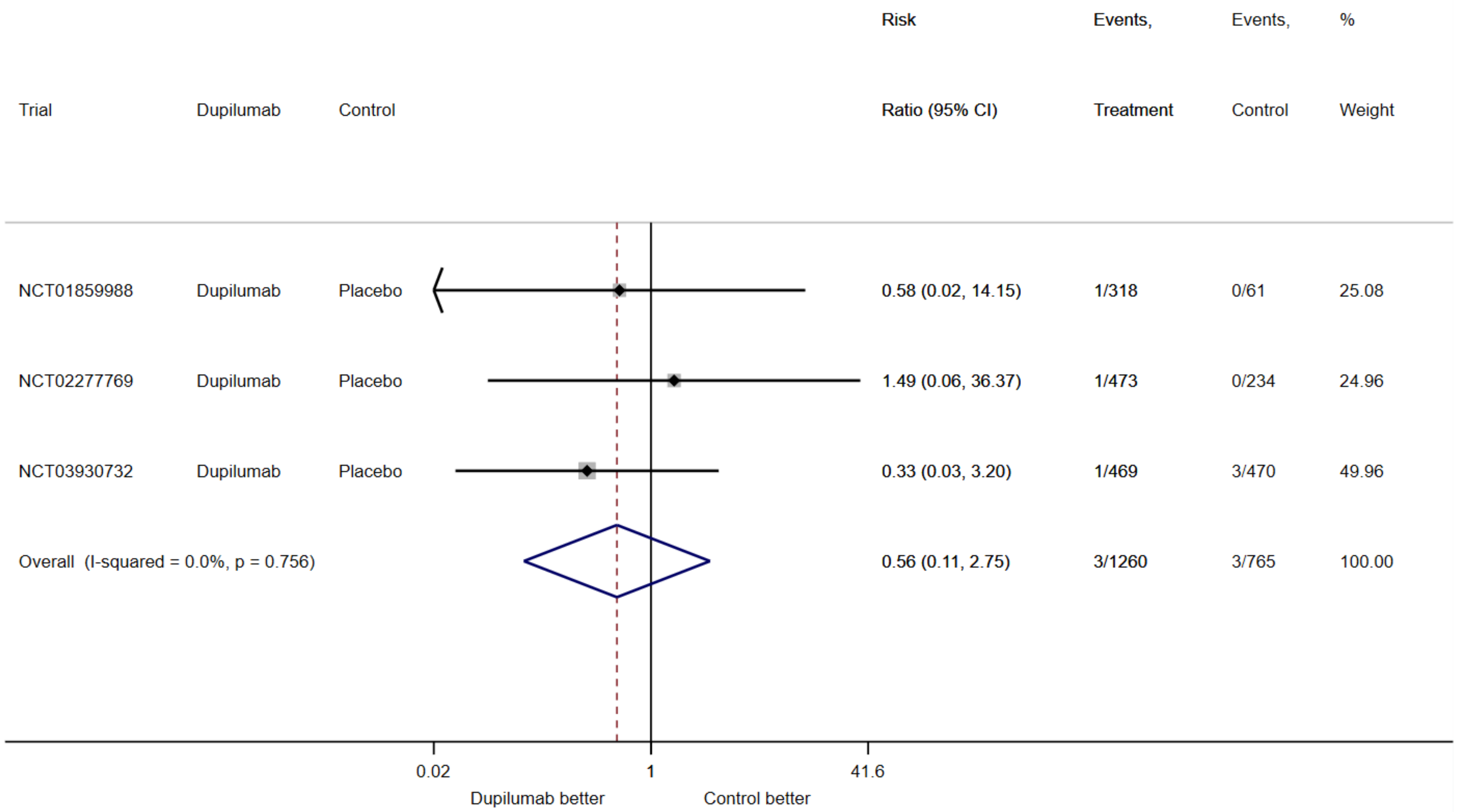

19_Respiratory, Thoracic And Mediastinal Disorders-Figure S9 Meta-analysis of Dupilumab and Respiratory failure

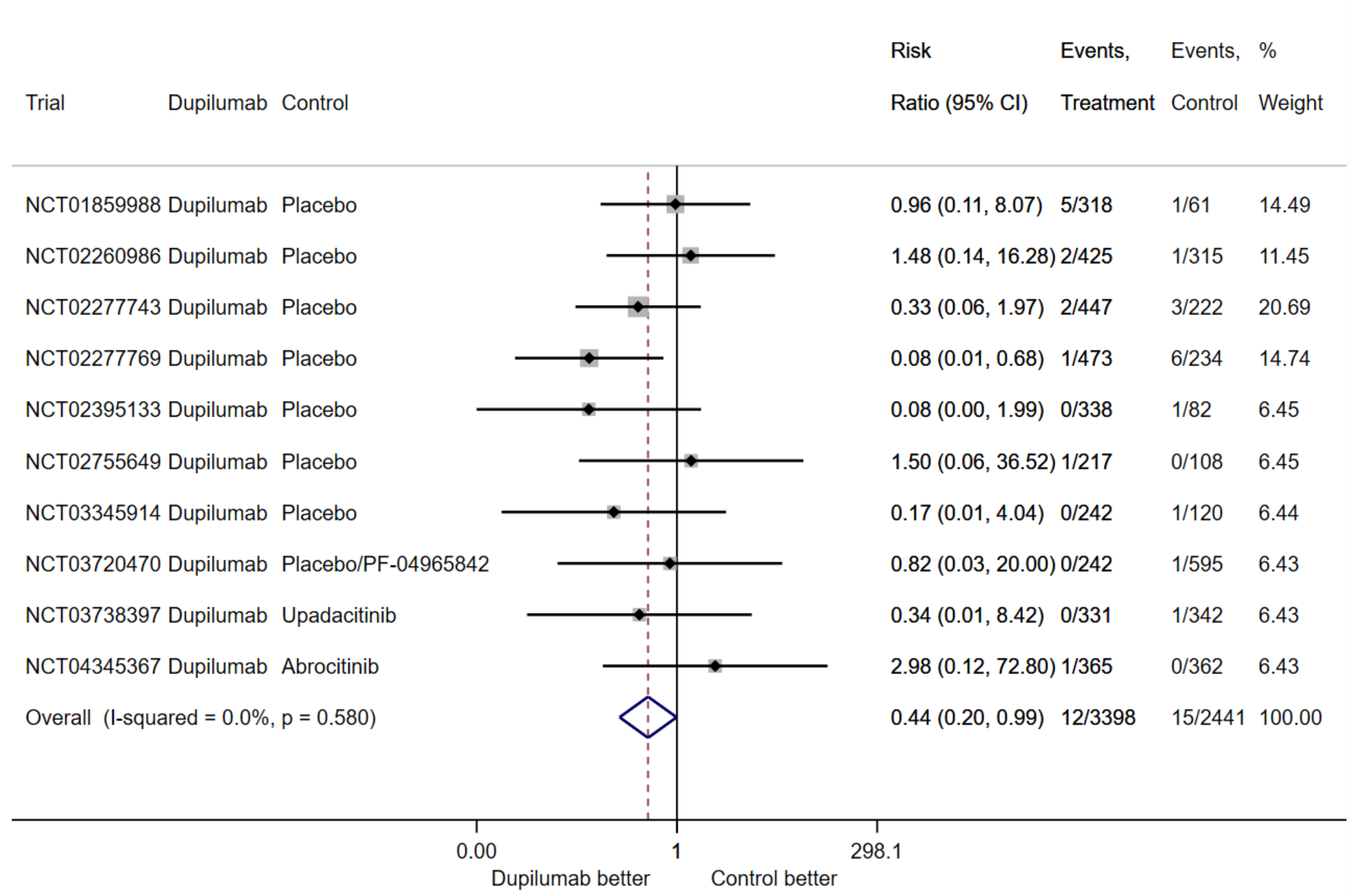

20_Skin And Subcutaneous Tissue Disorders-Figure S1 Meta-analysis of Dupilumab and Dermatitis atopic

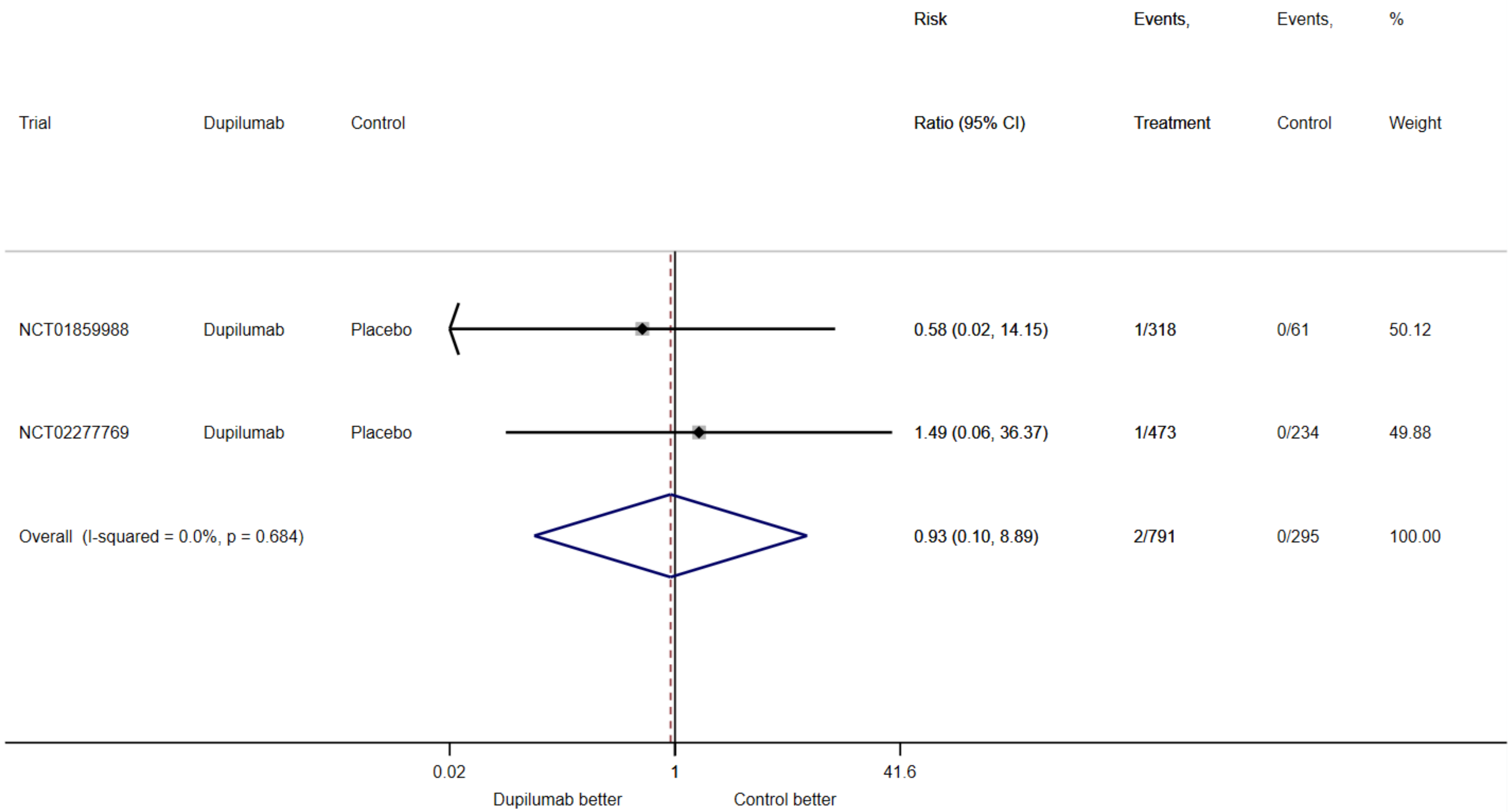

20_Skin And Subcutaneous Tissue Disorders-Figure S2 Meta-analysis of Dupilumab and Dermatitis exfoliative

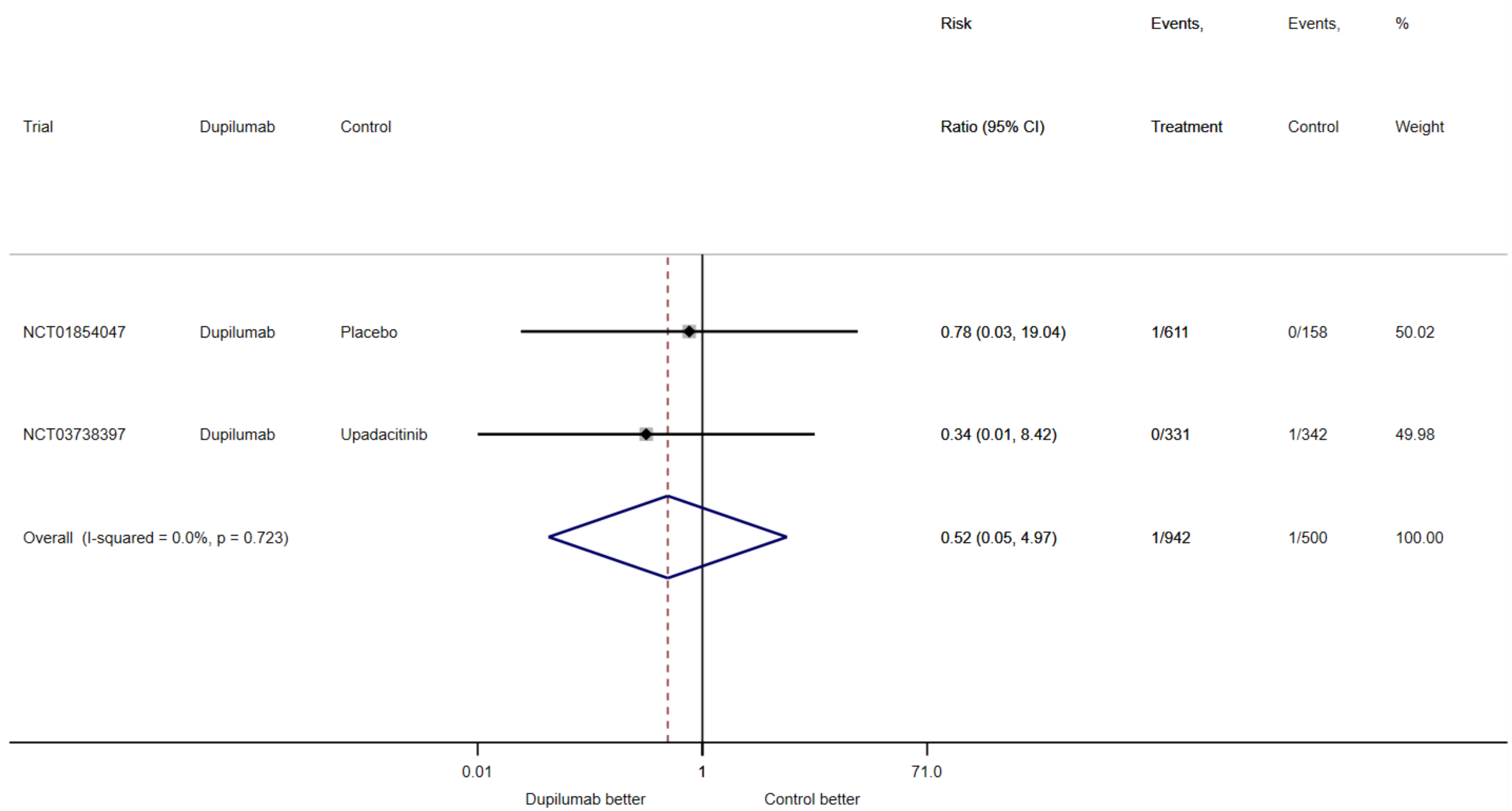

20_Skin And Subcutaneous Tissue Disorders-Figure S3 Meta-analysis of Dupilumab and Eczema

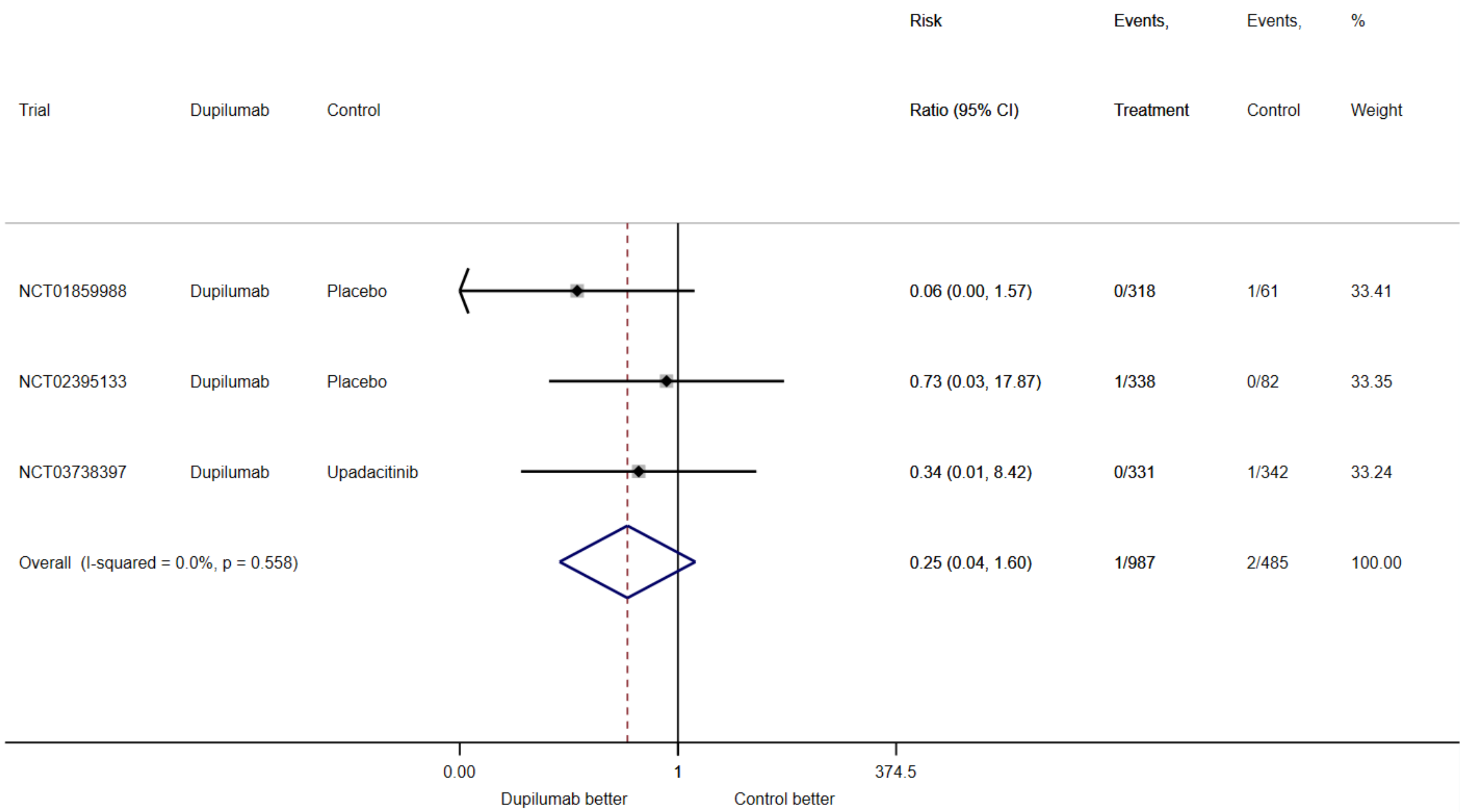

21_Surgical And Medical Procedures-Figure S1 Meta-analysis of Dupilumab and Abortion induced

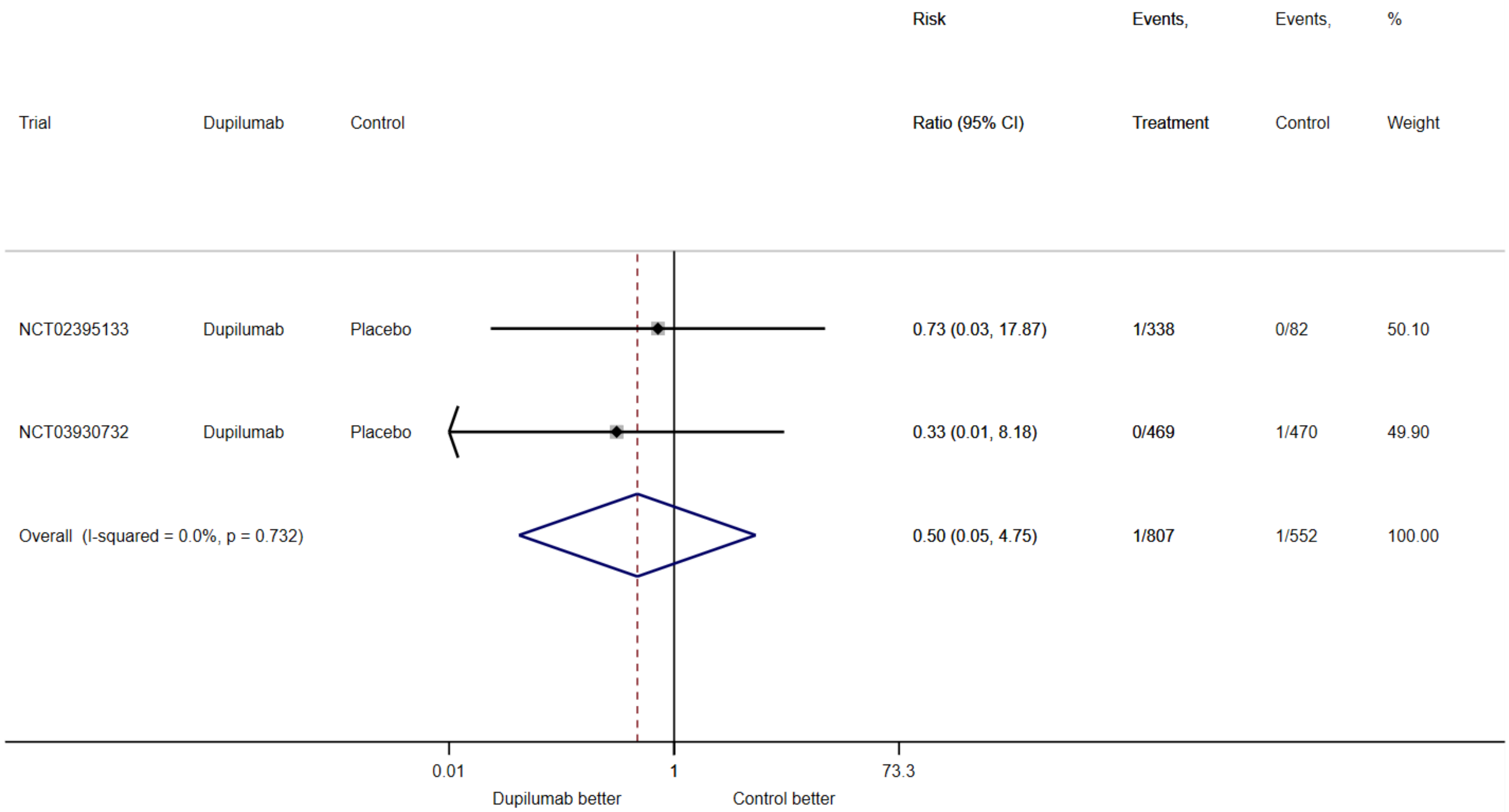

22_Vascular Disorders-Figure S1 Meta-analysis of Dupilumab and Deep vein thrombosis

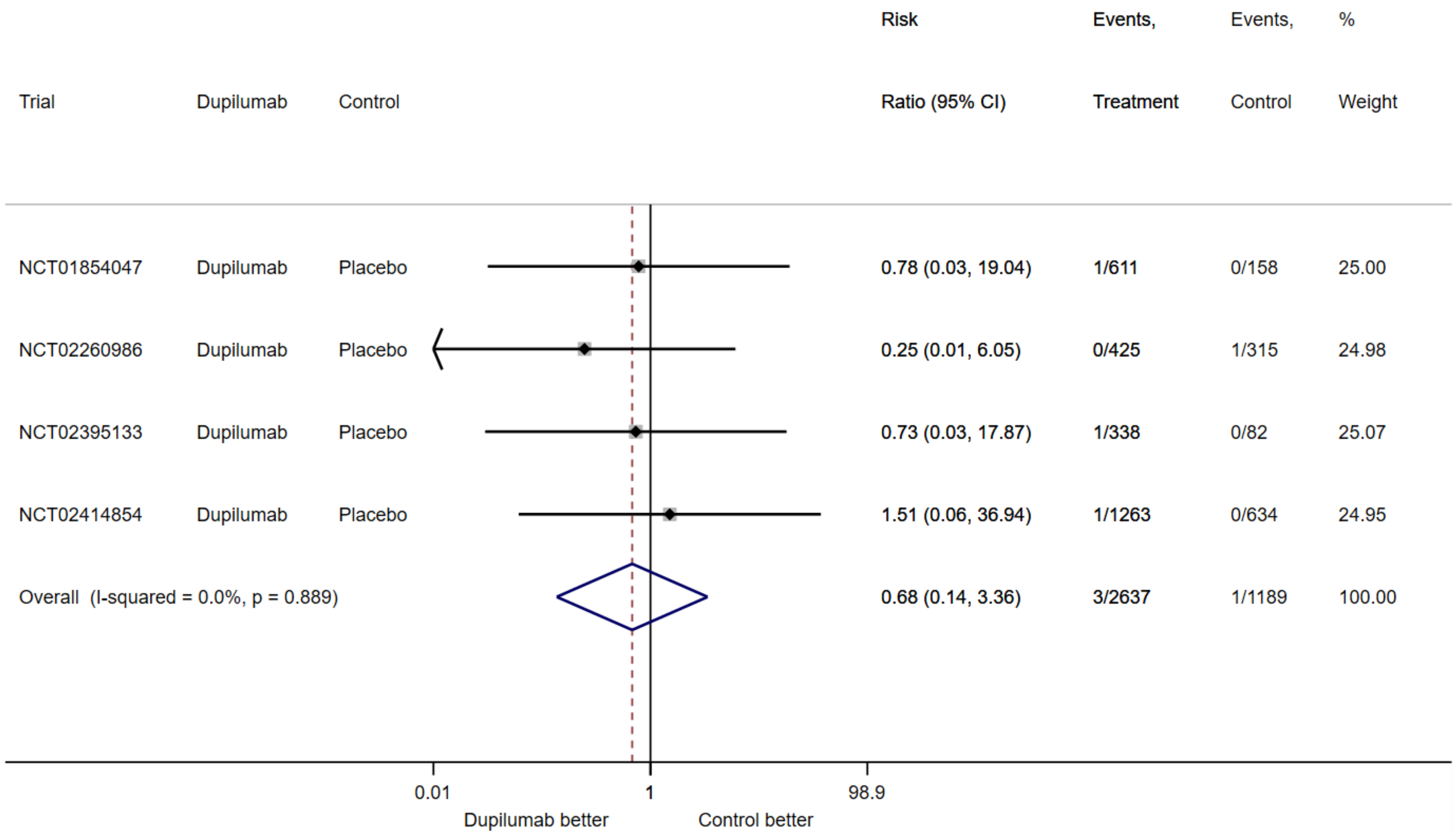

22_Vascular Disorders-Figure S2 Meta-analysis of Dupilumab and Hypertension
