# Supplementary figures and images for: A comprehensive analysis on the safety of two biologics dupilumab and omalizumab
Source: Front Med (Lausanne). 2024 Aug 8;11:1435370. doi: 10.3389/fmed.2024.1435370 (PMC11338893; doi:10.3389/fmed.2024.1435370)

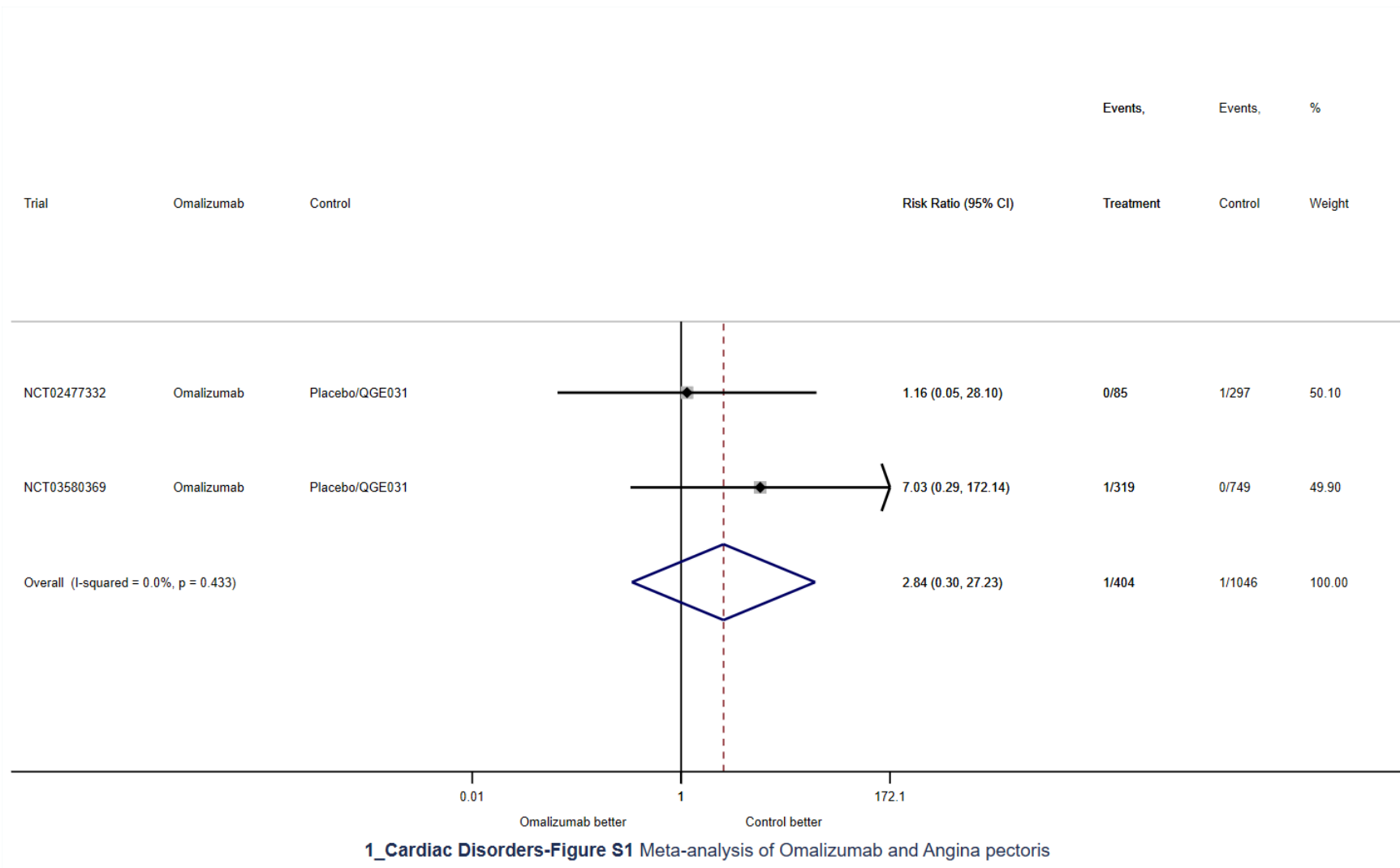

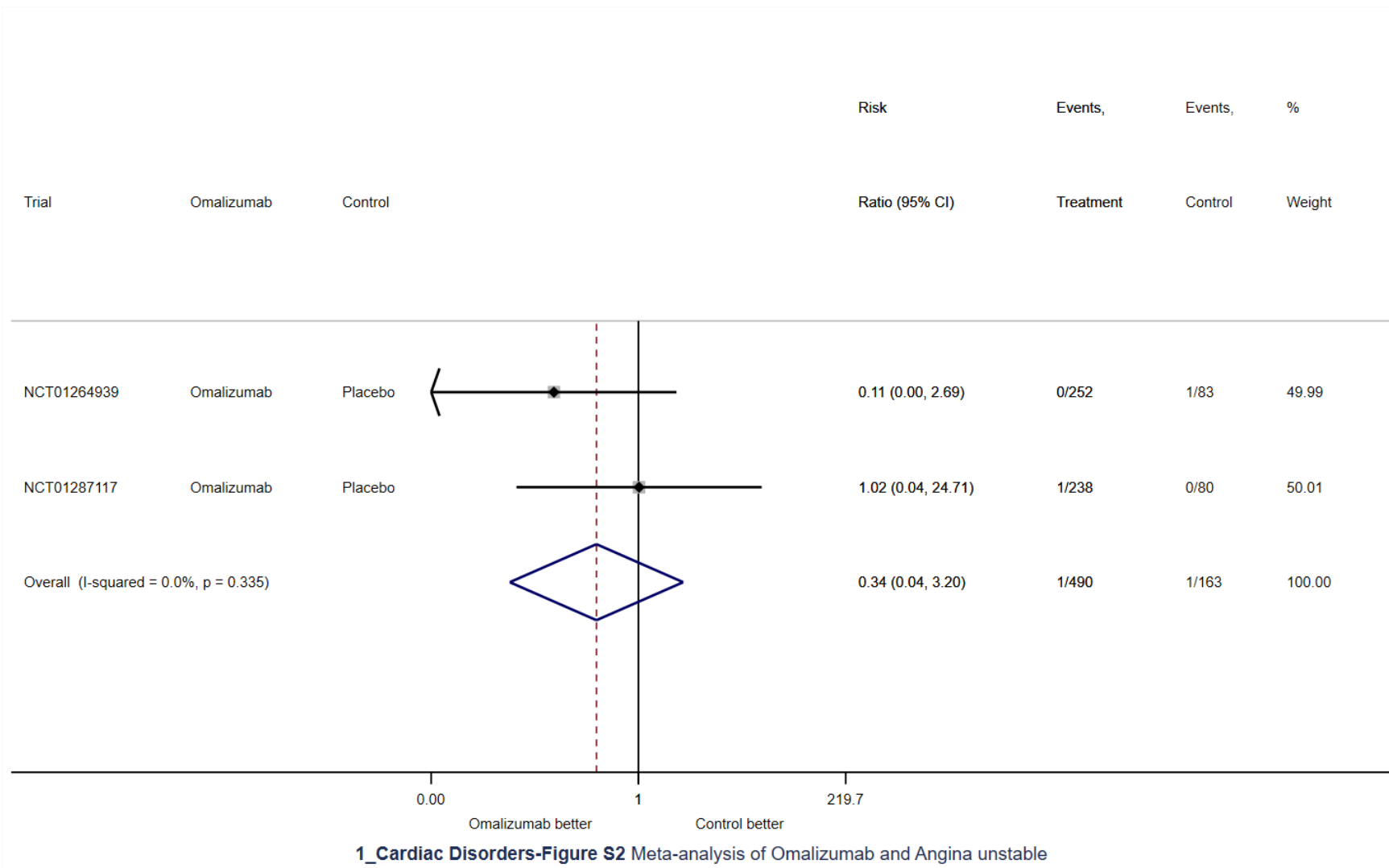

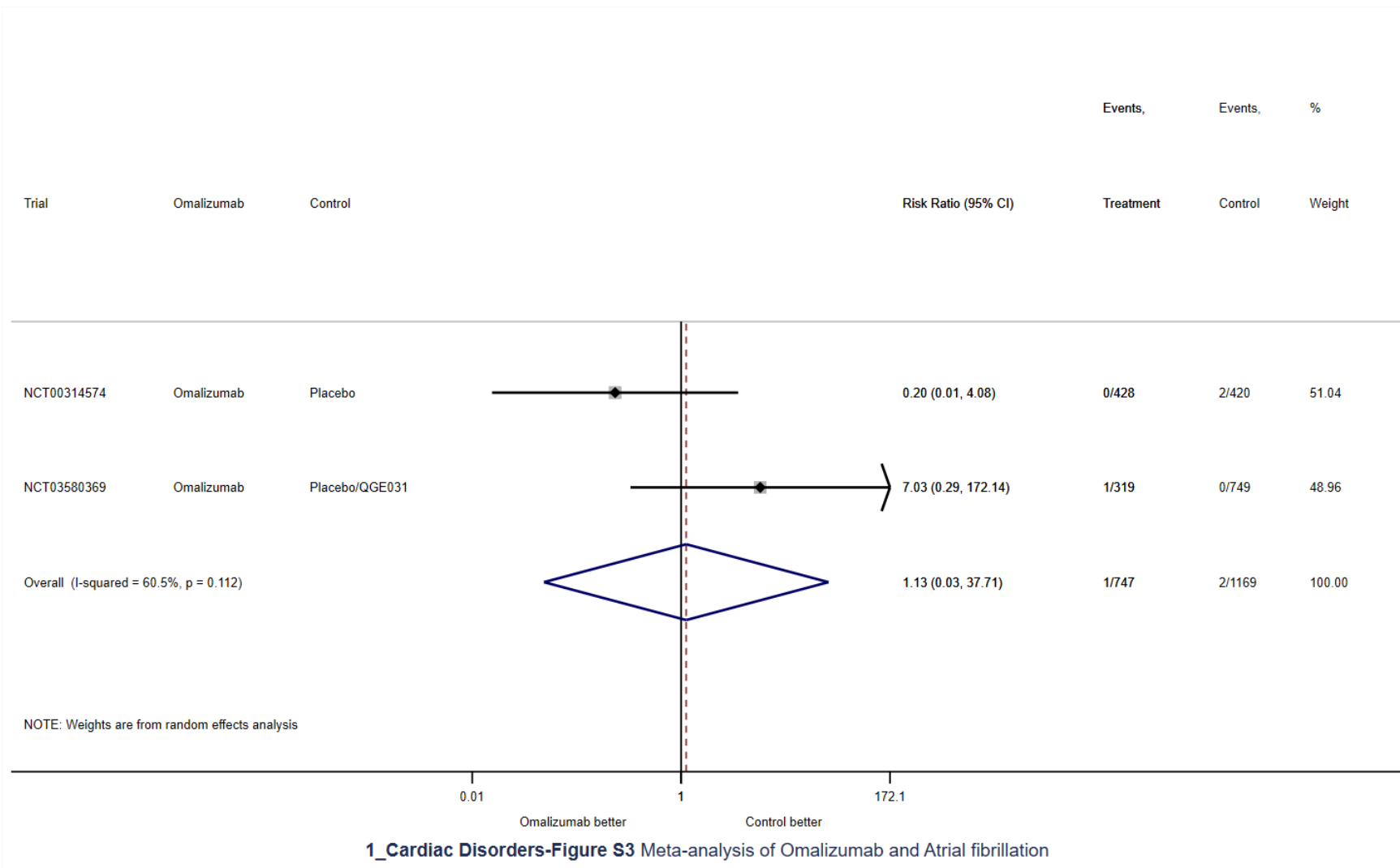

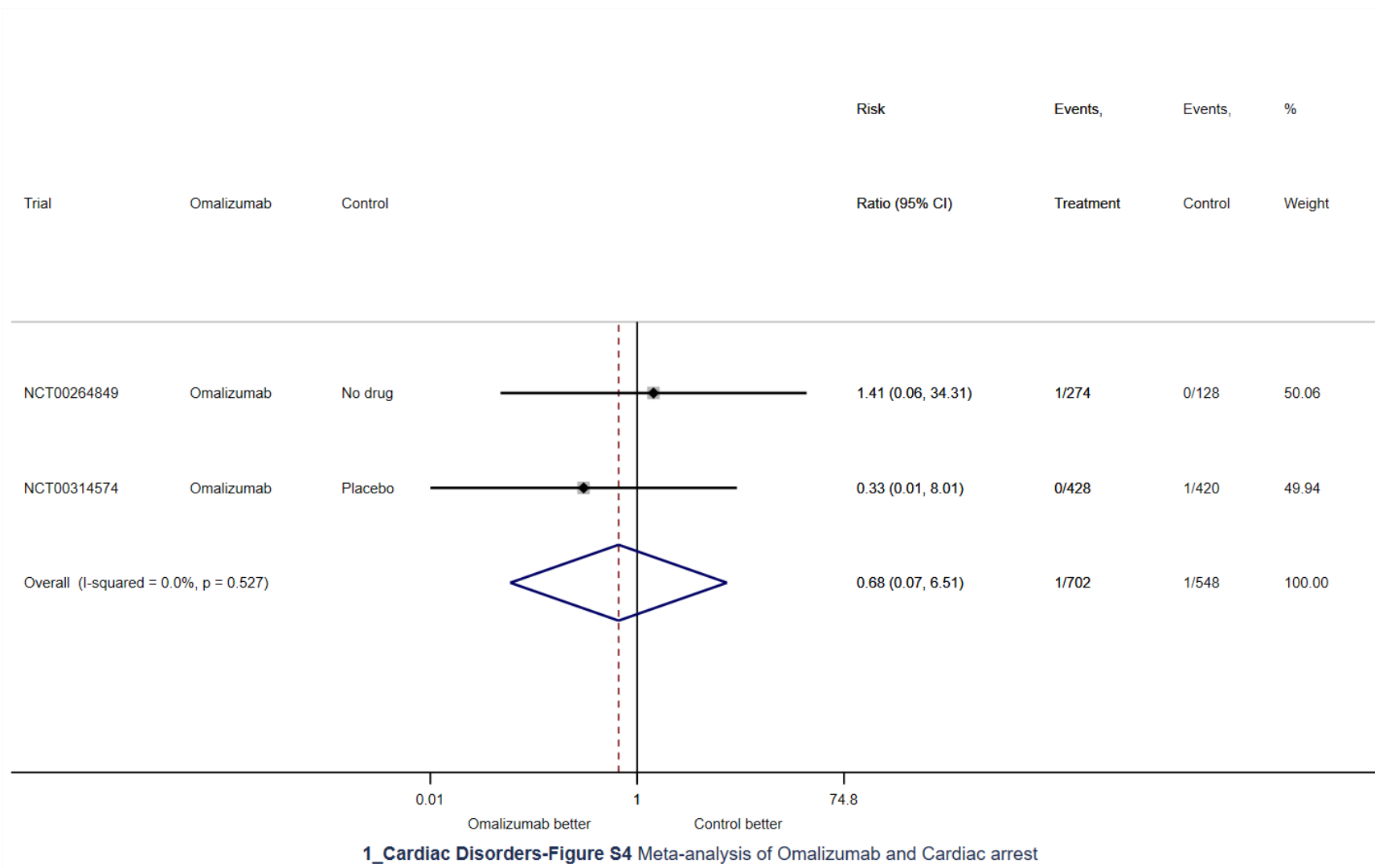

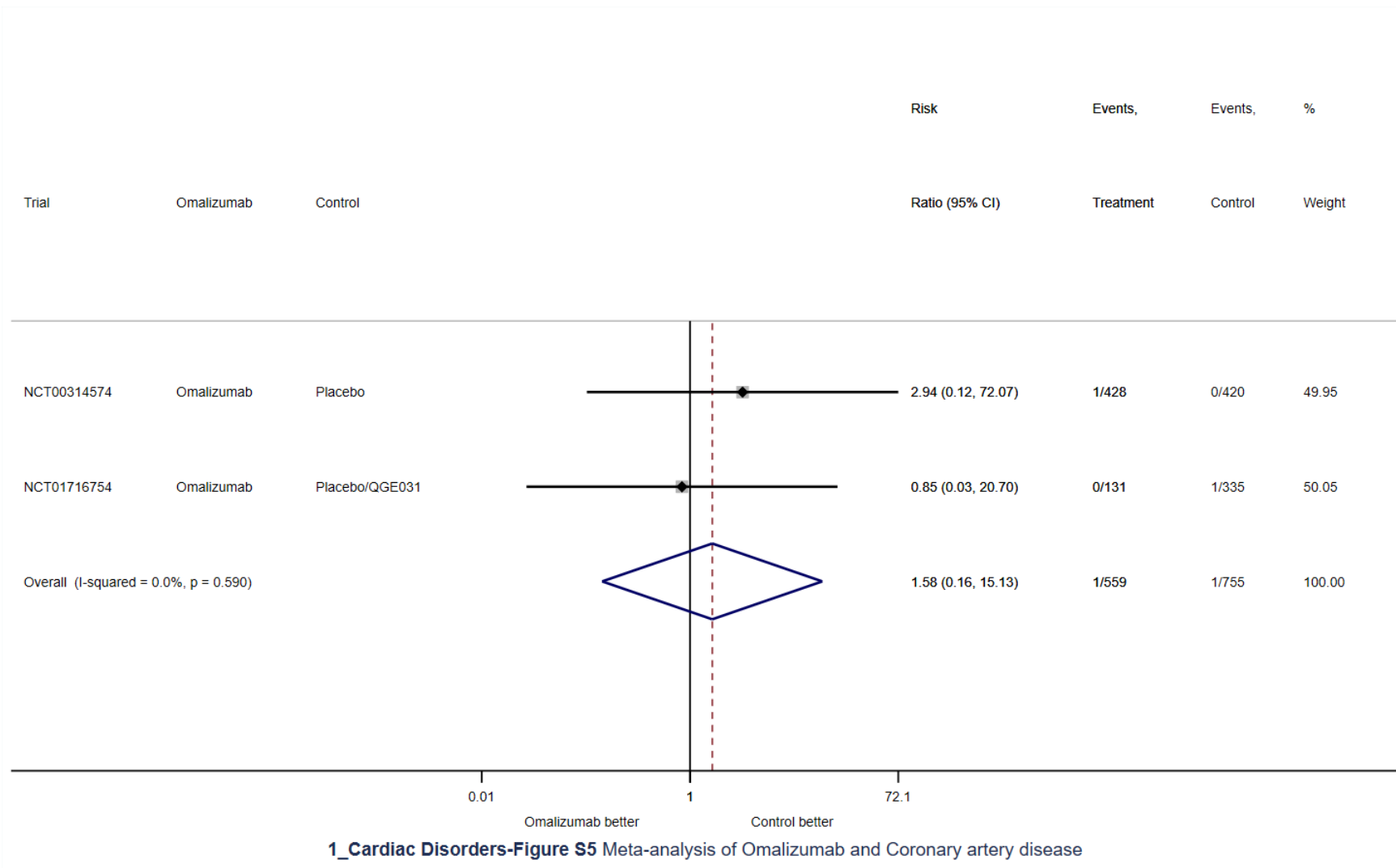

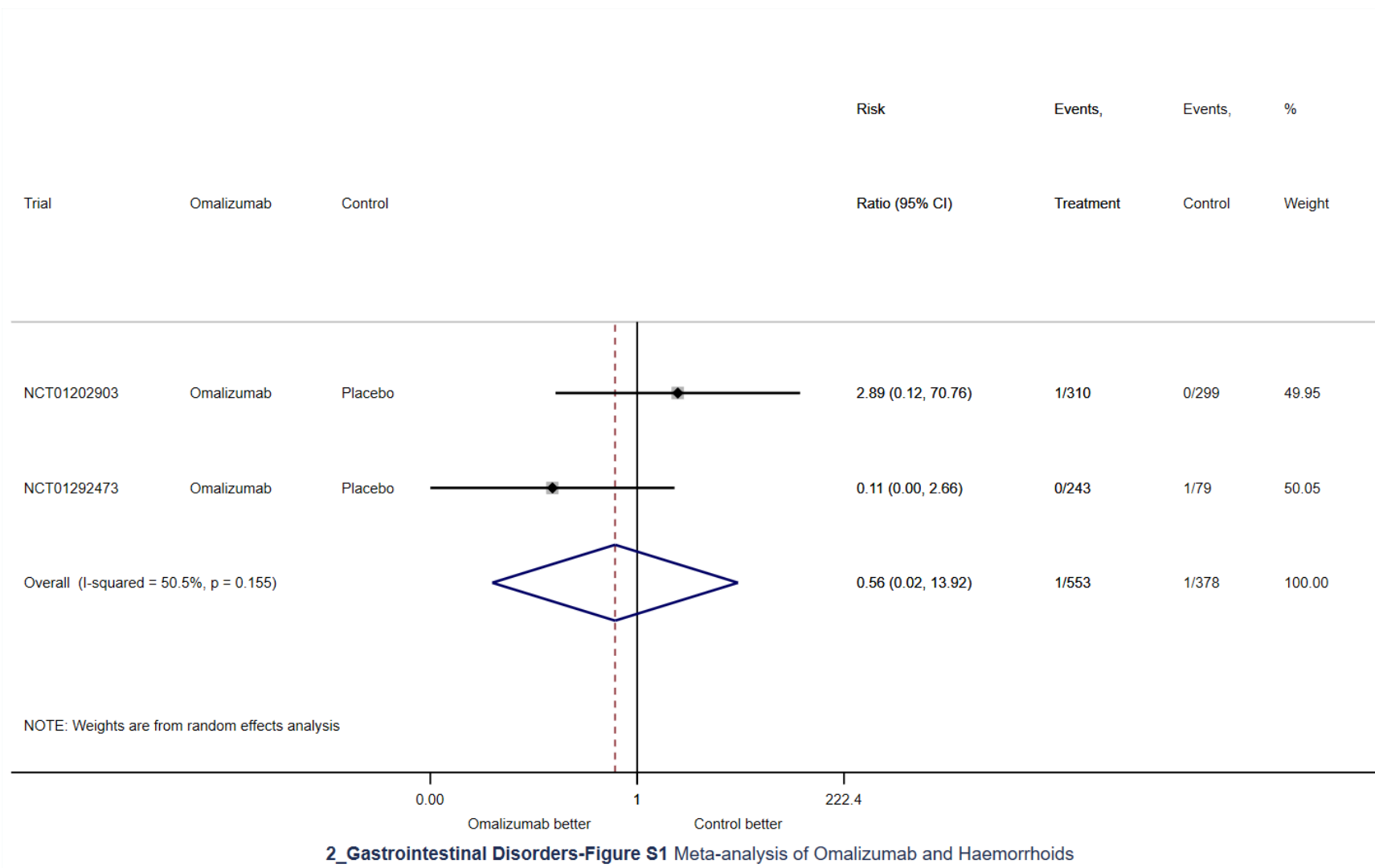

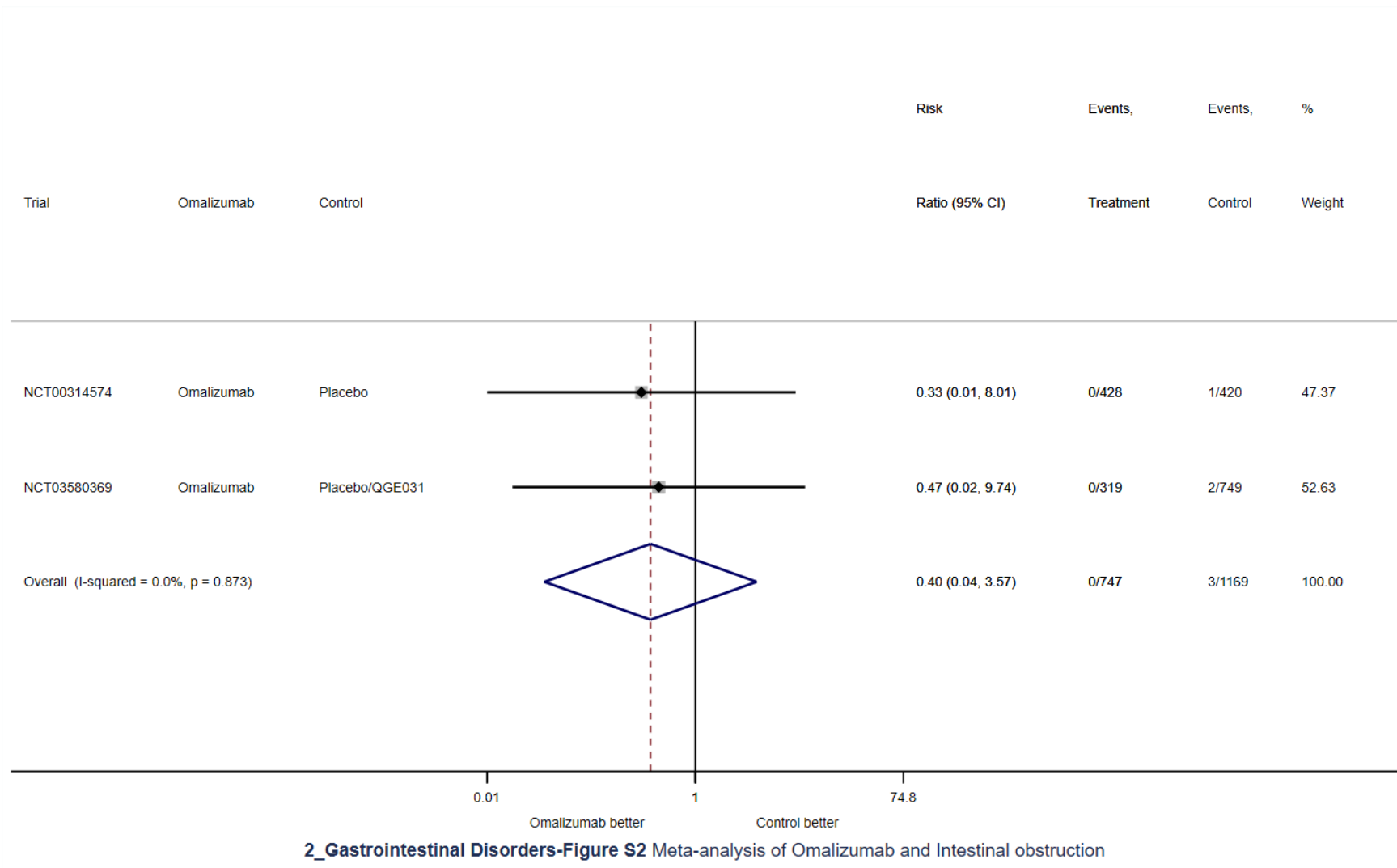

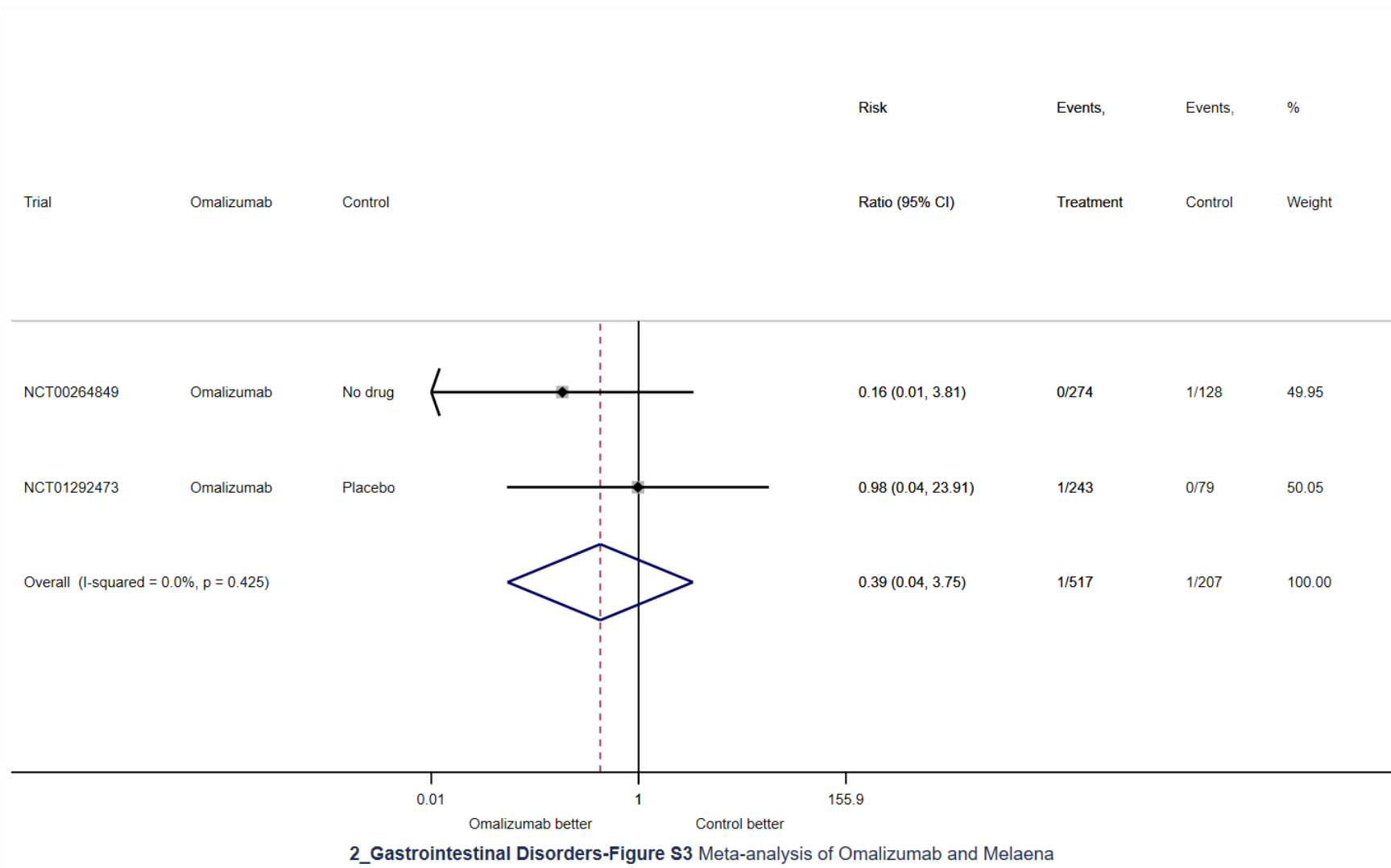

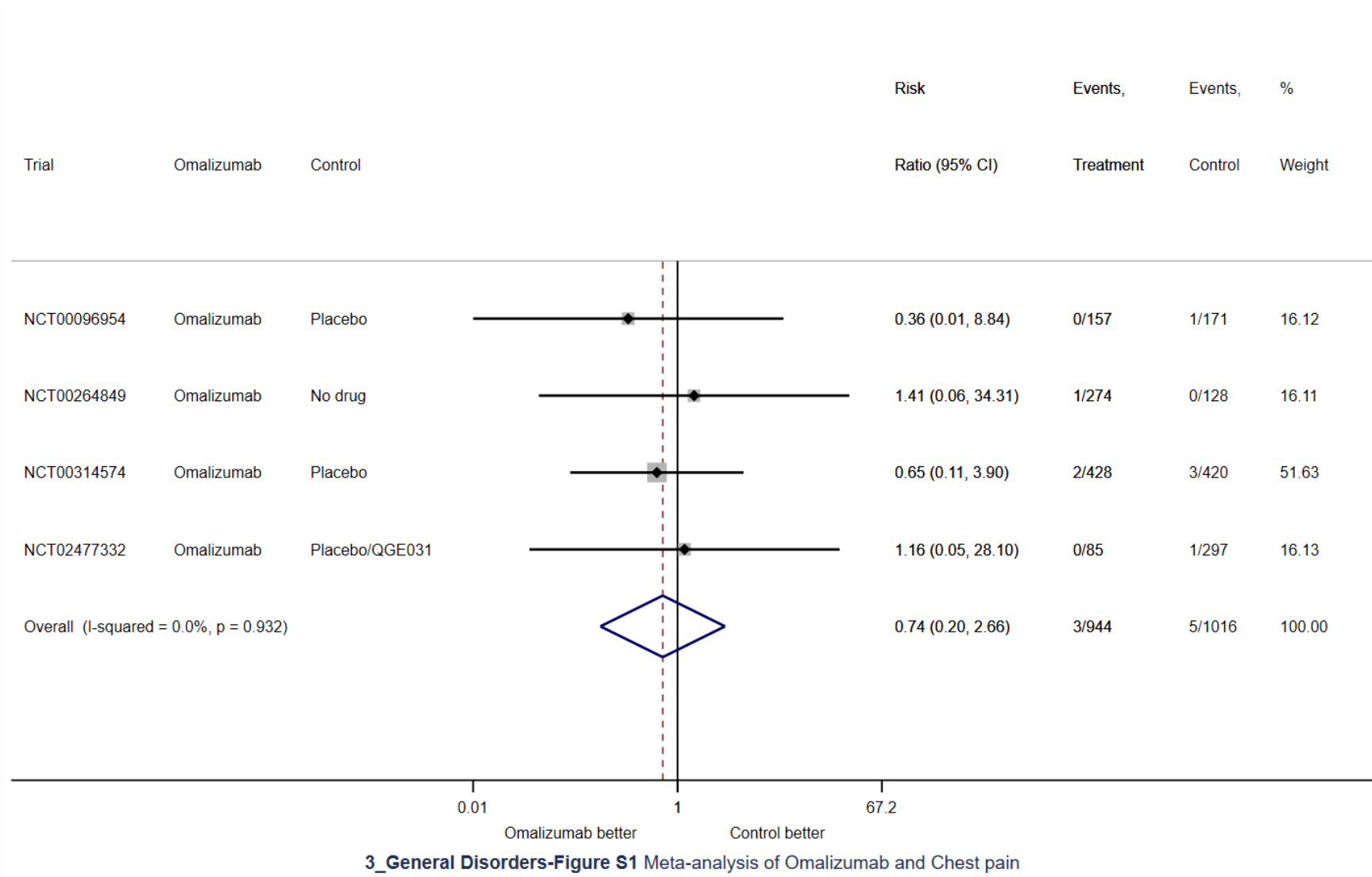

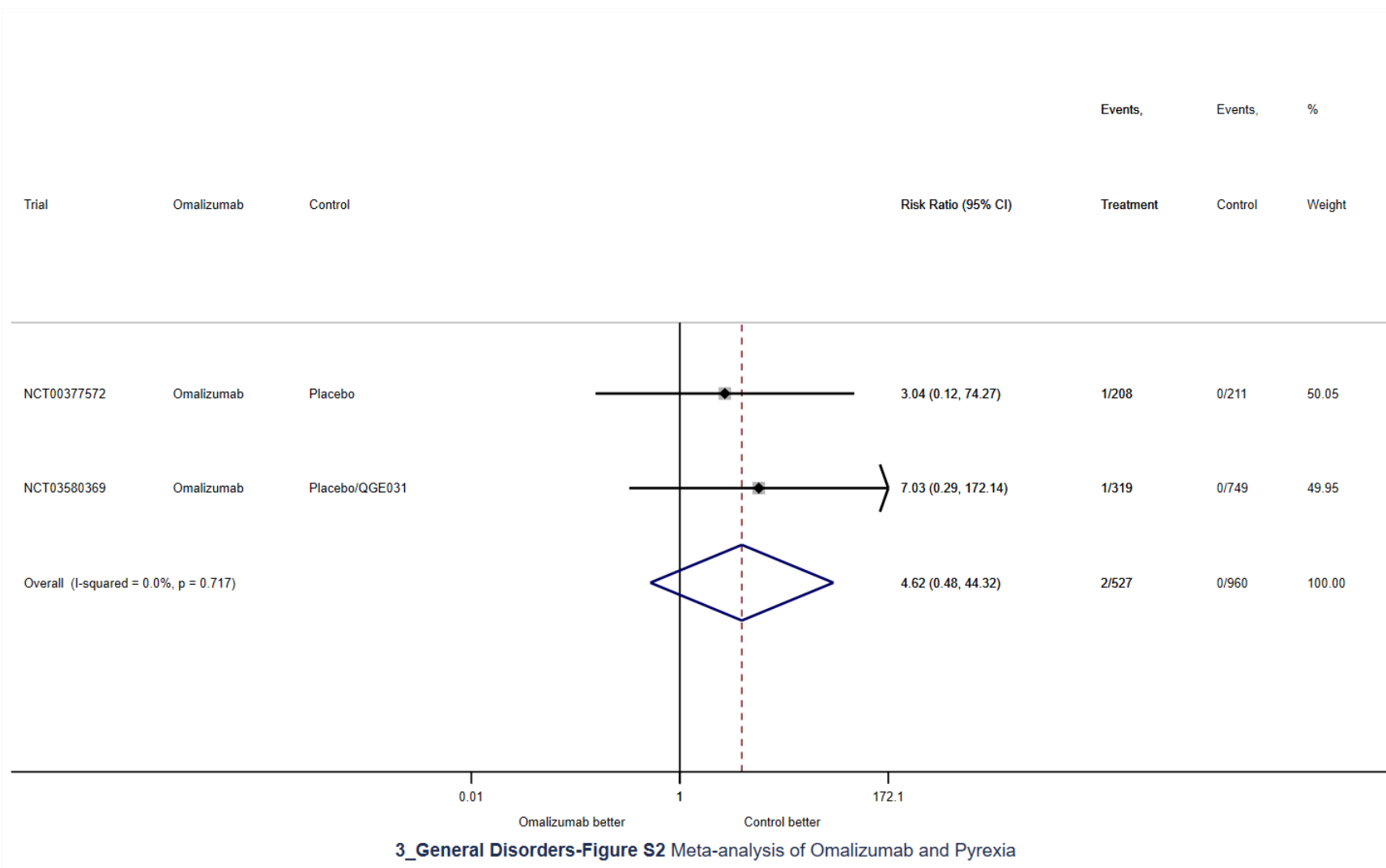

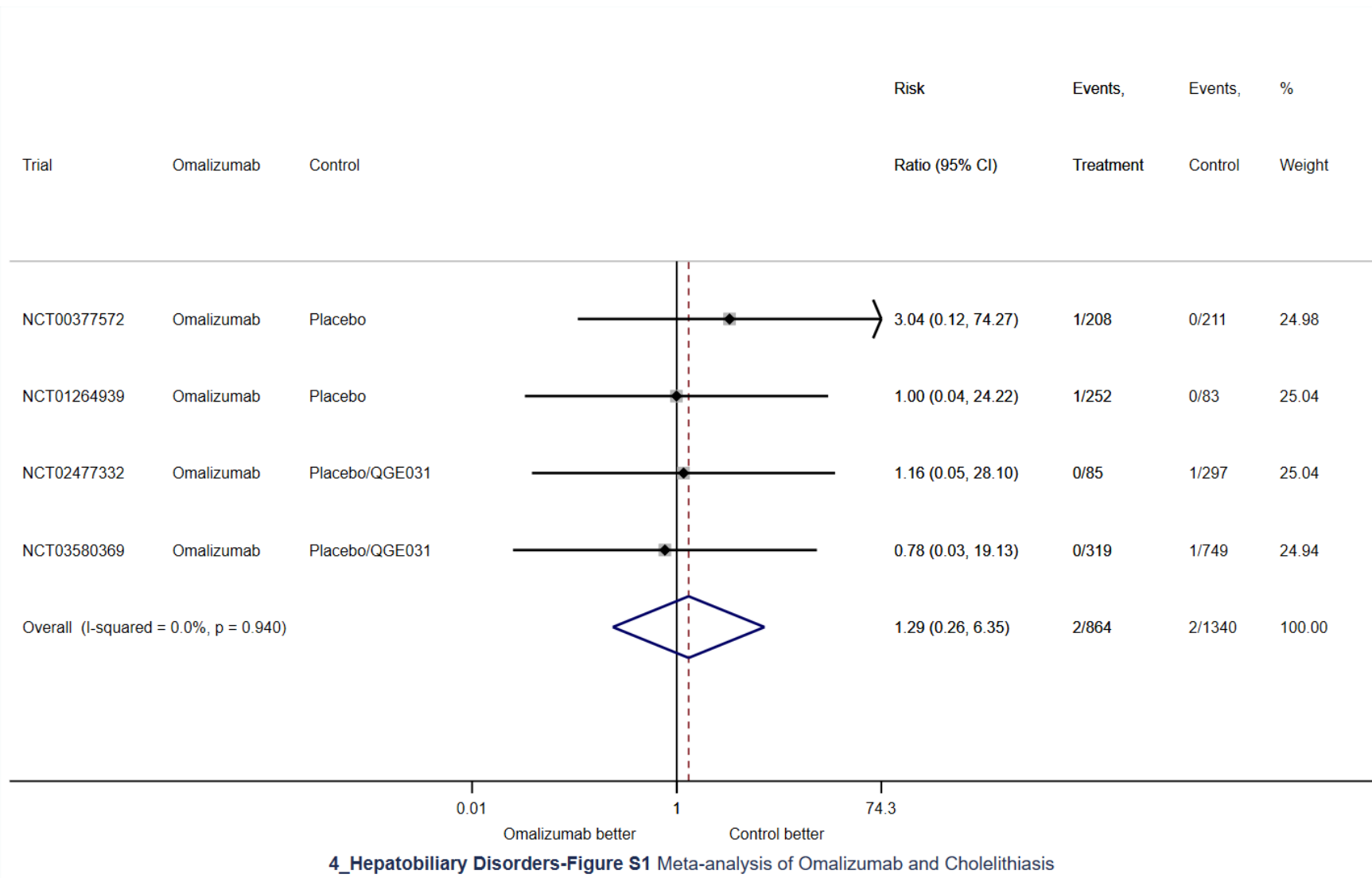

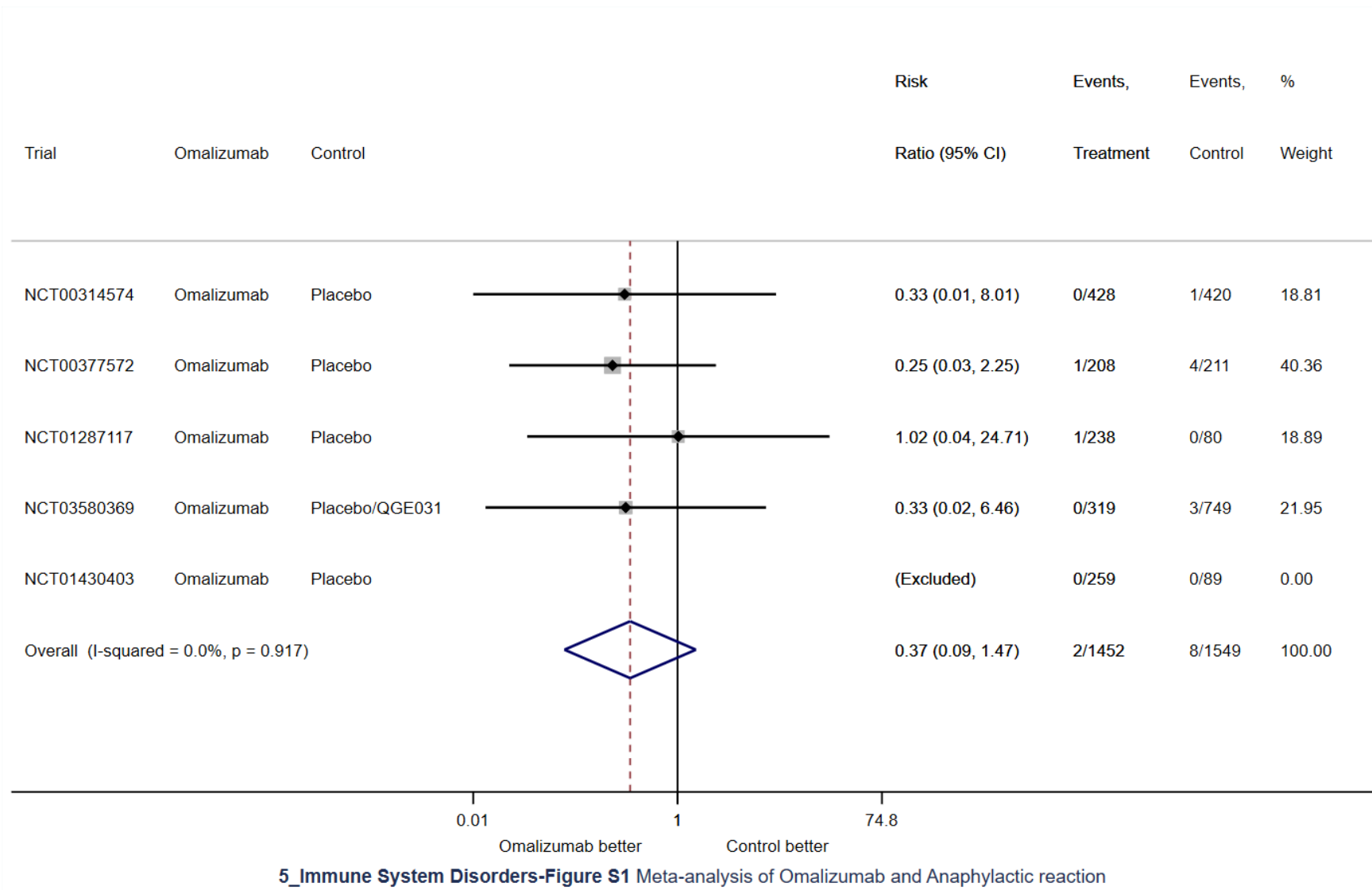

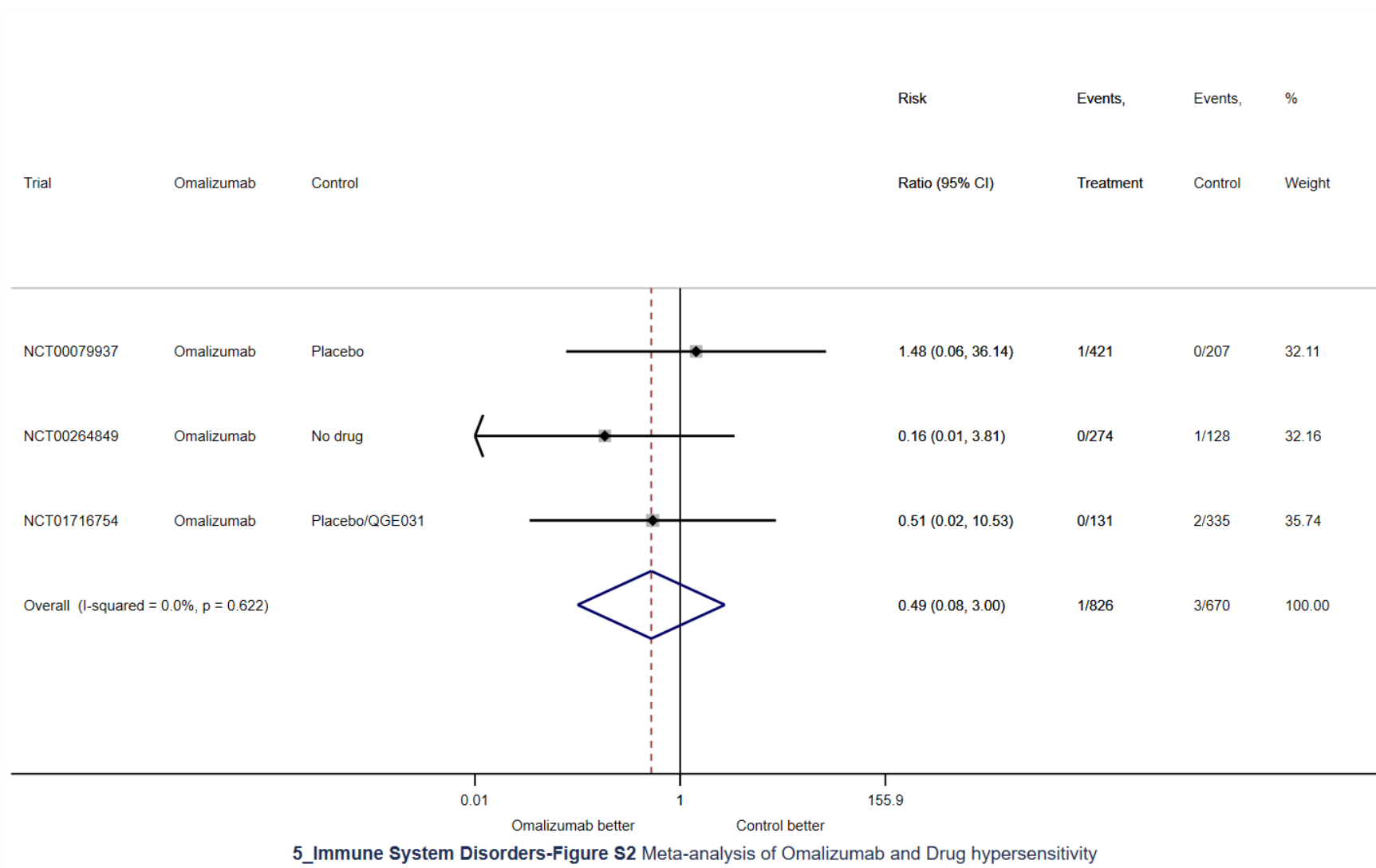

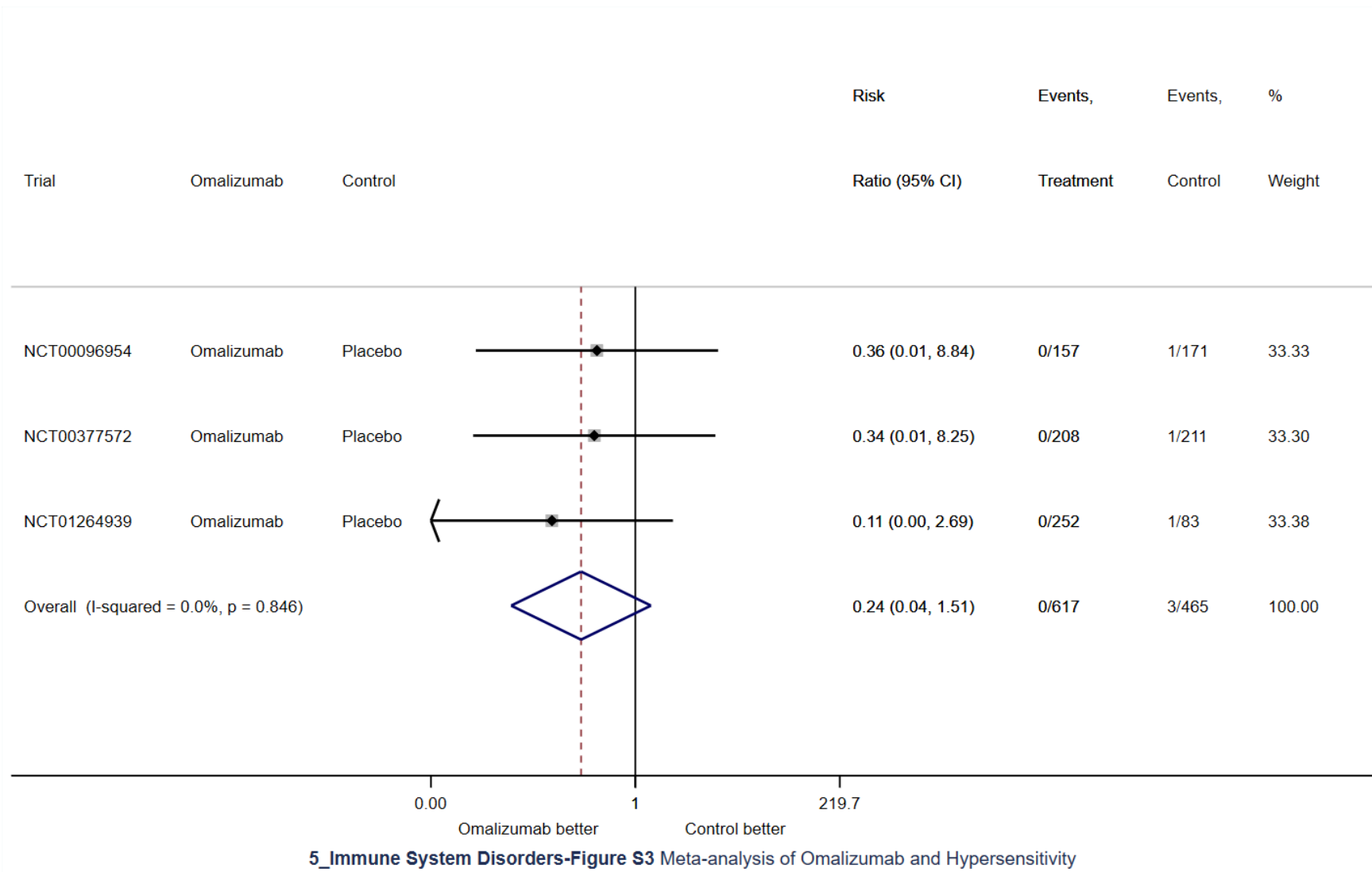

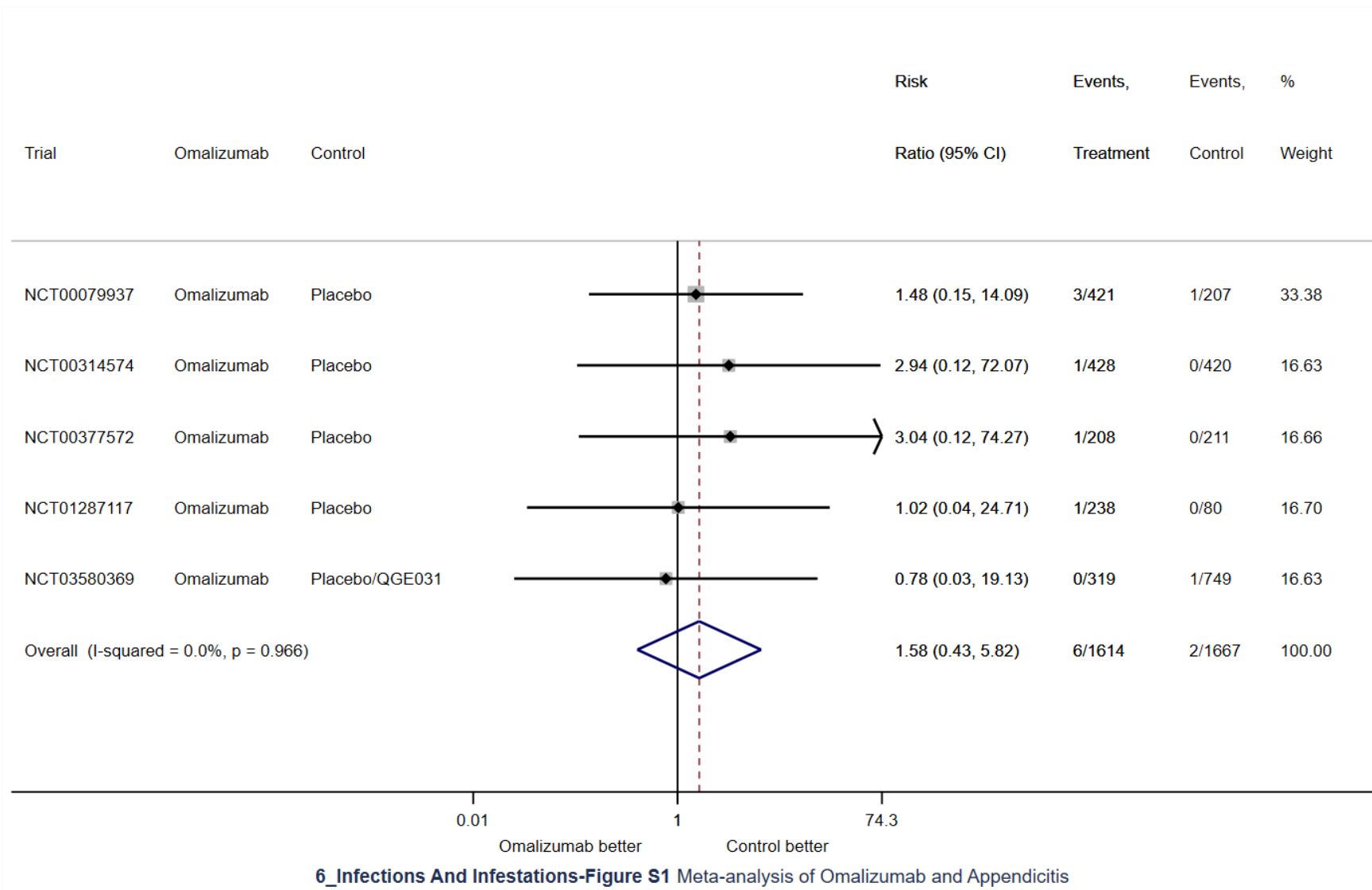

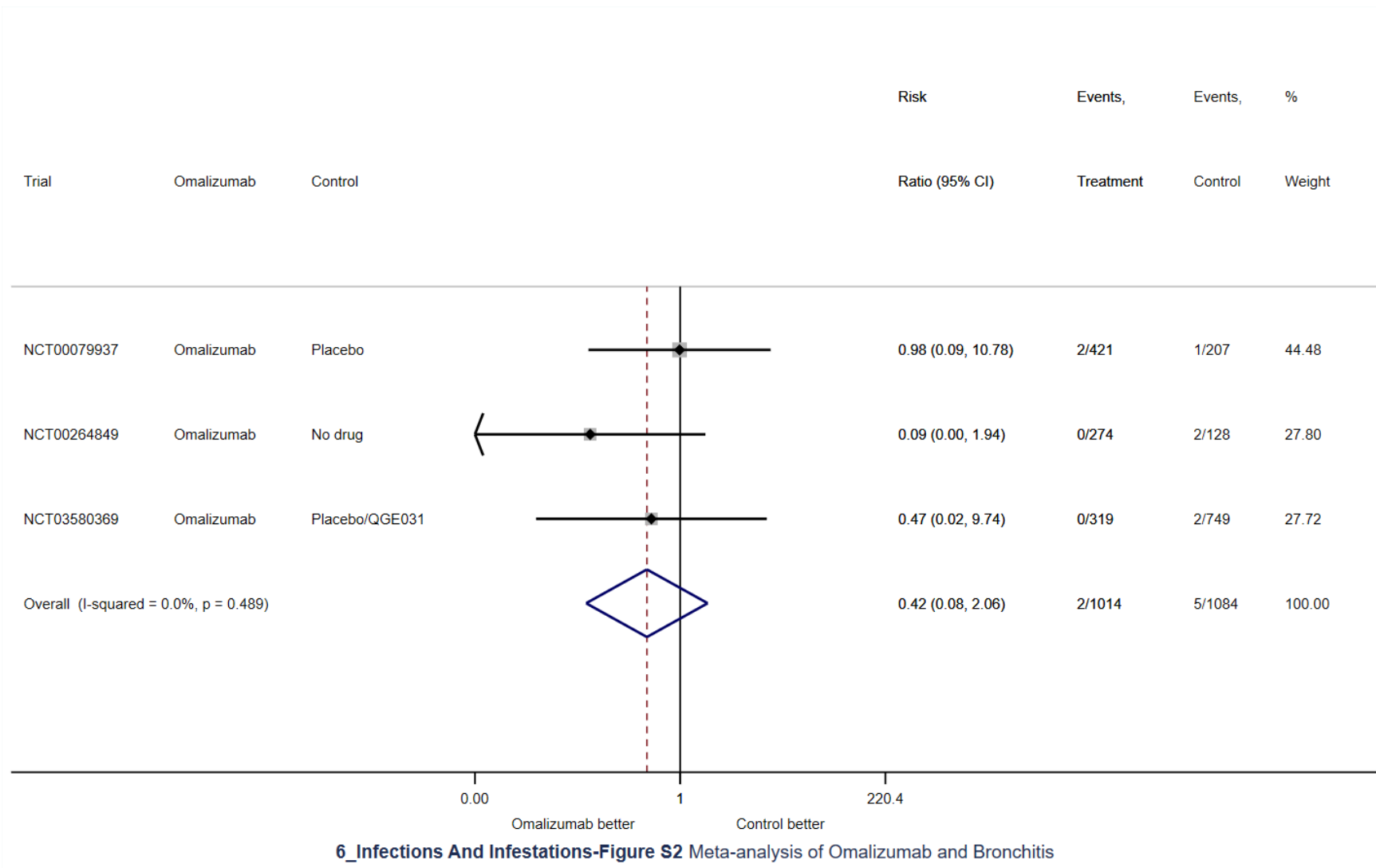

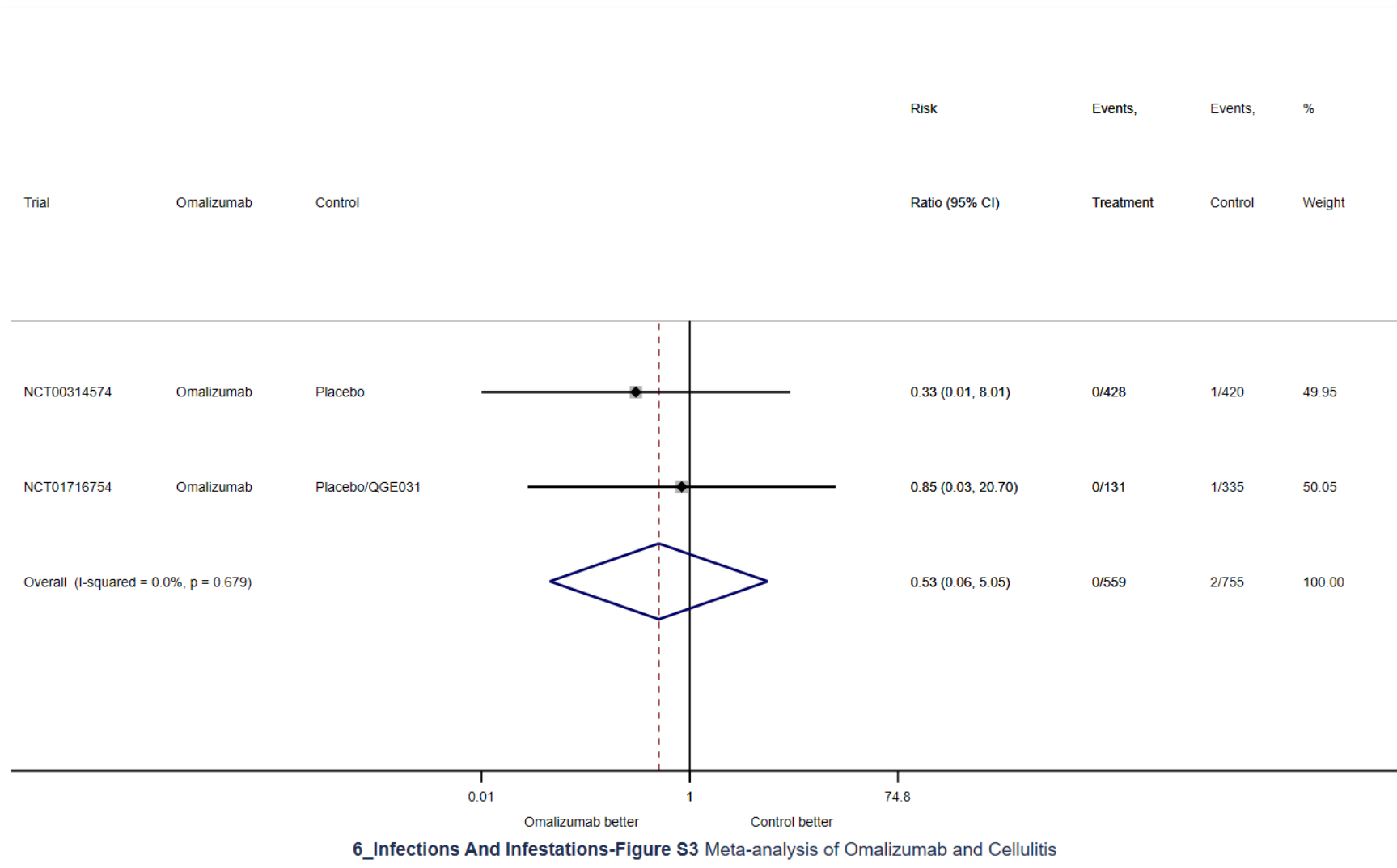

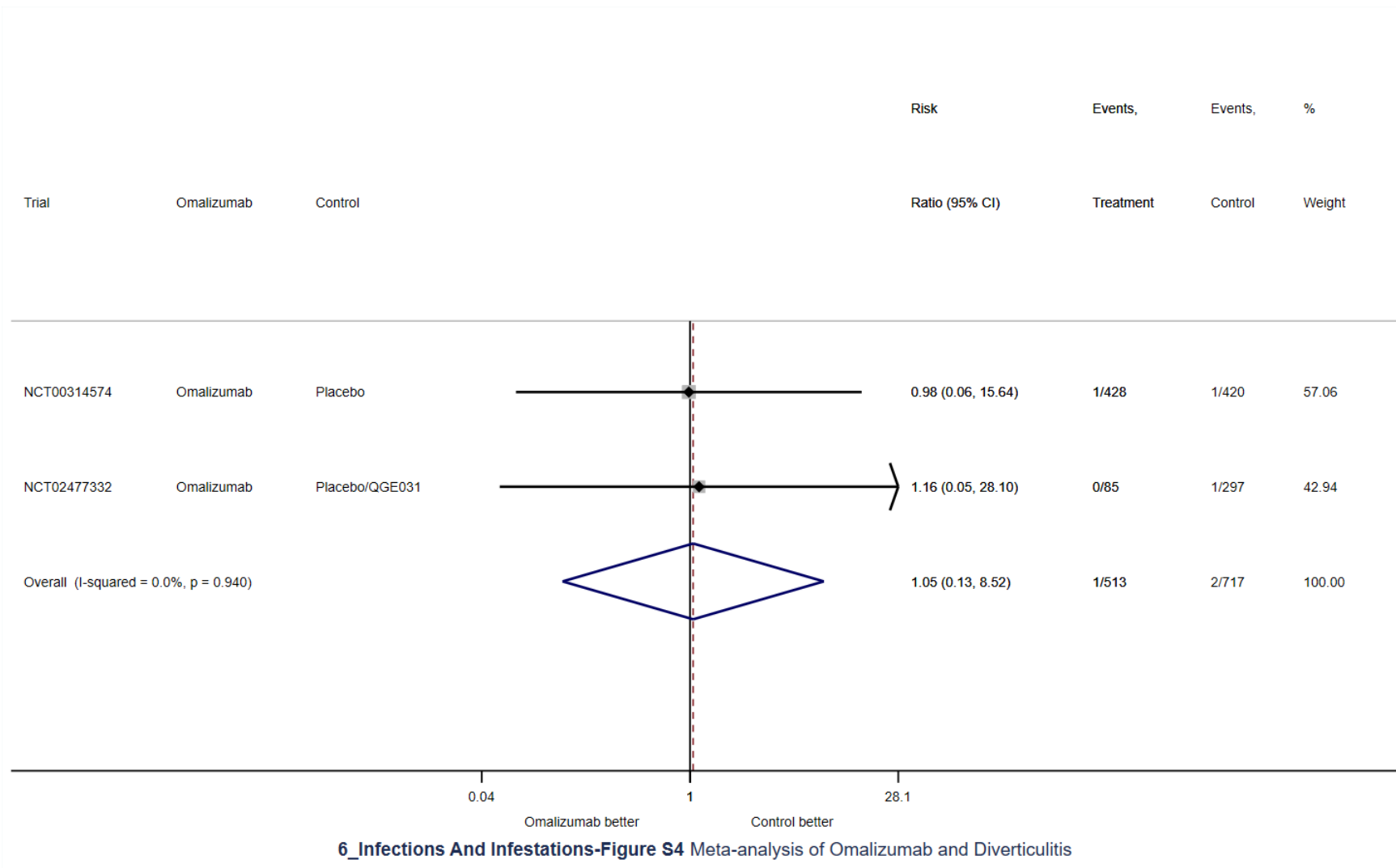

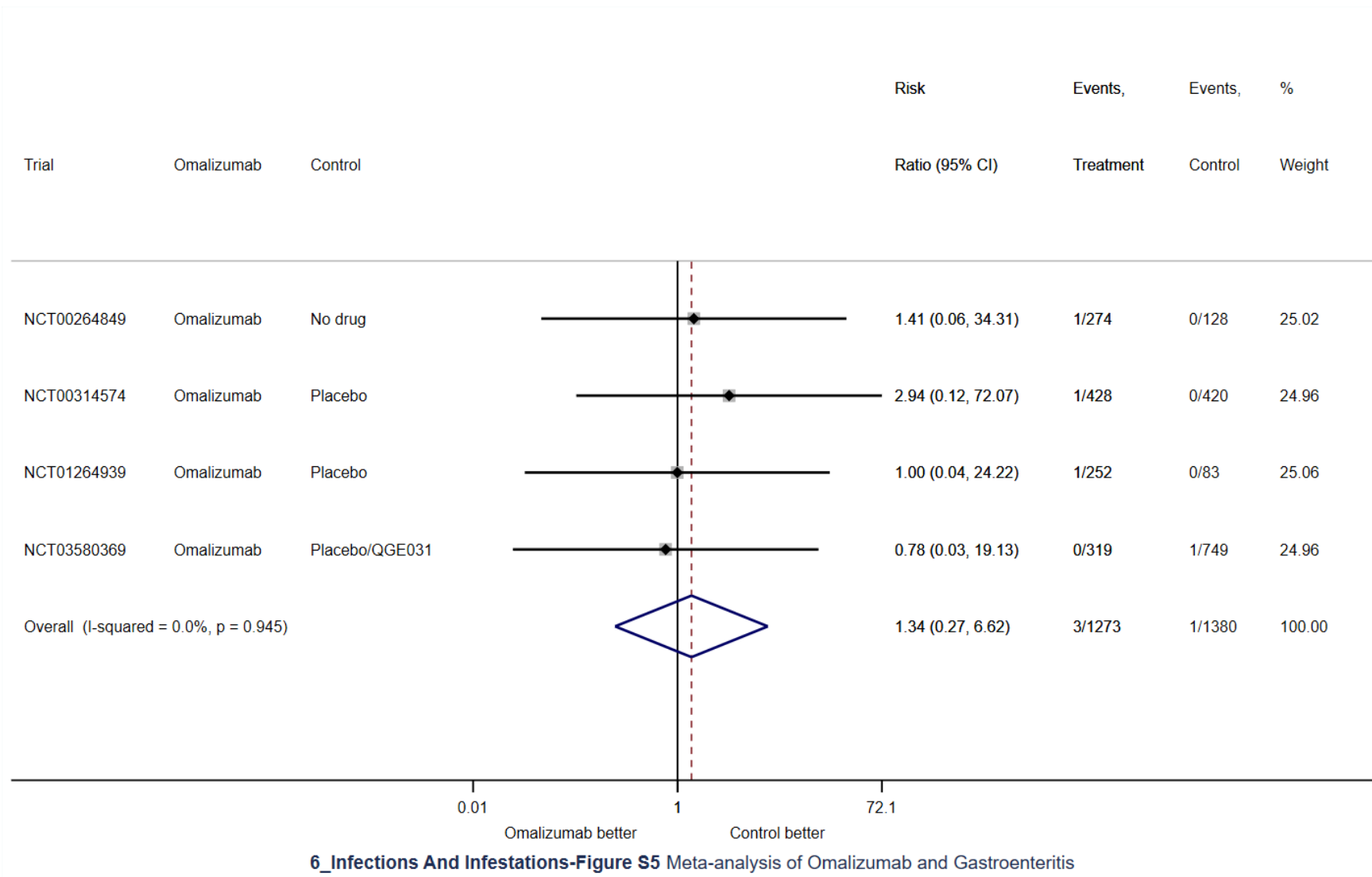

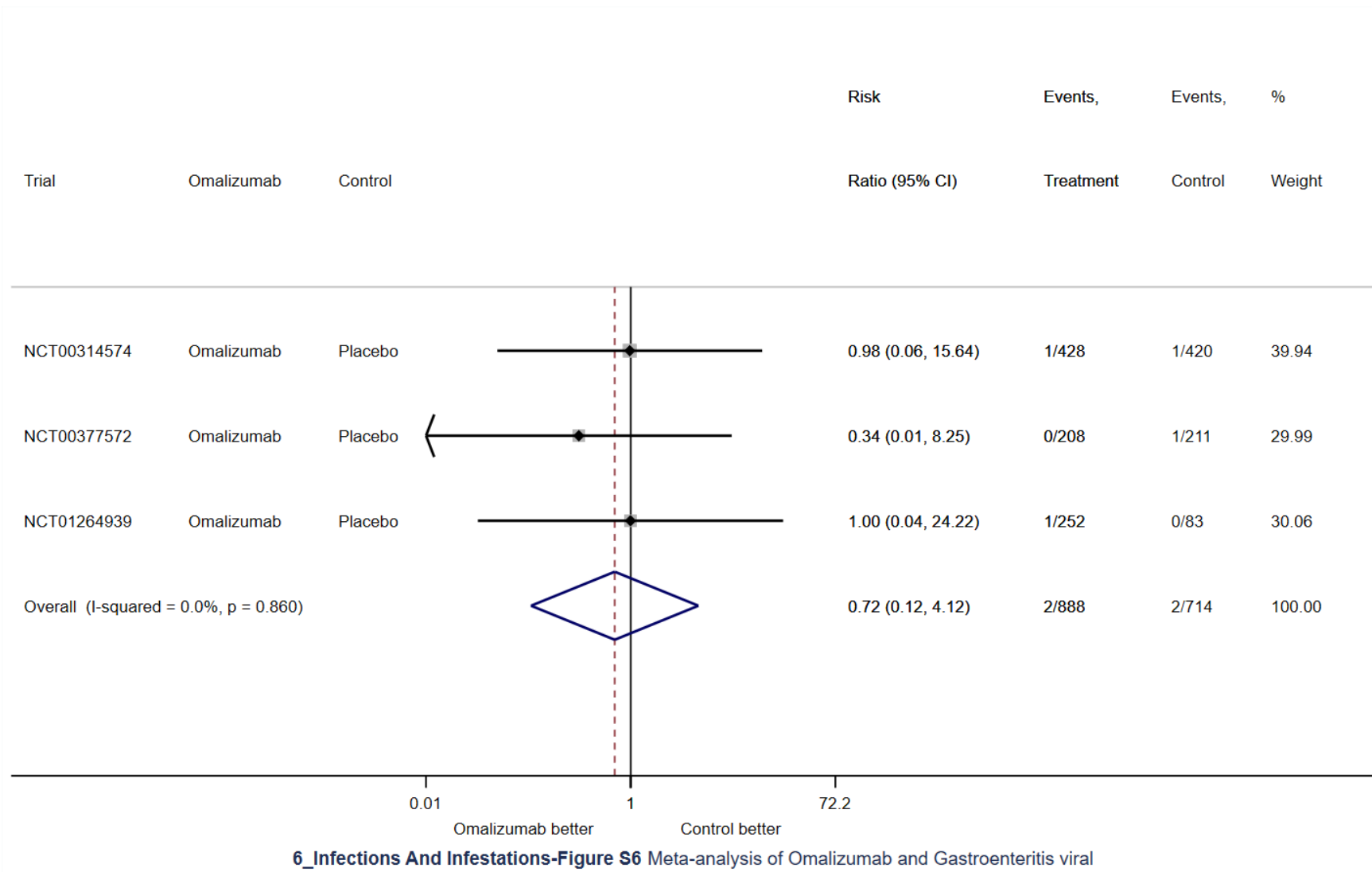

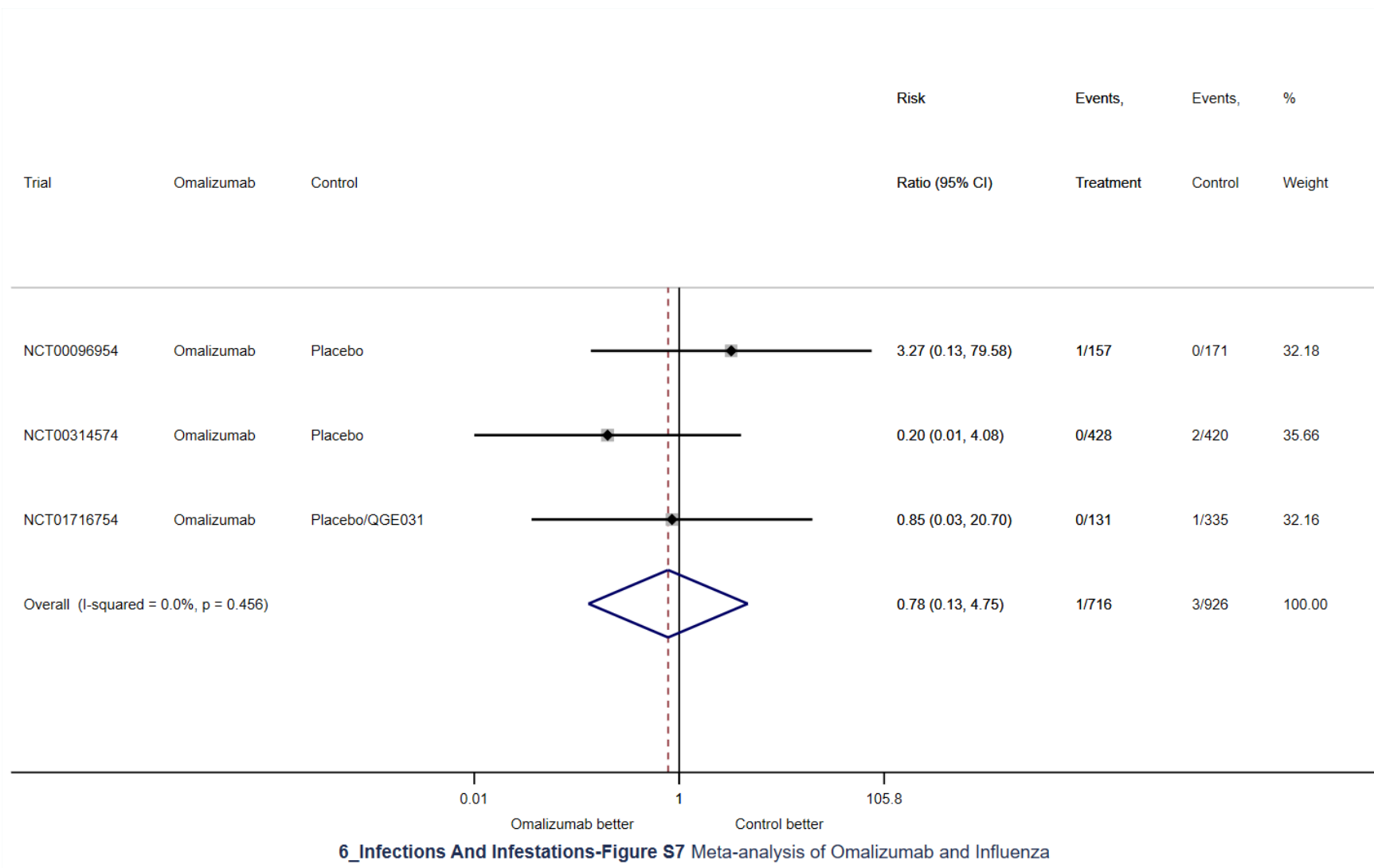

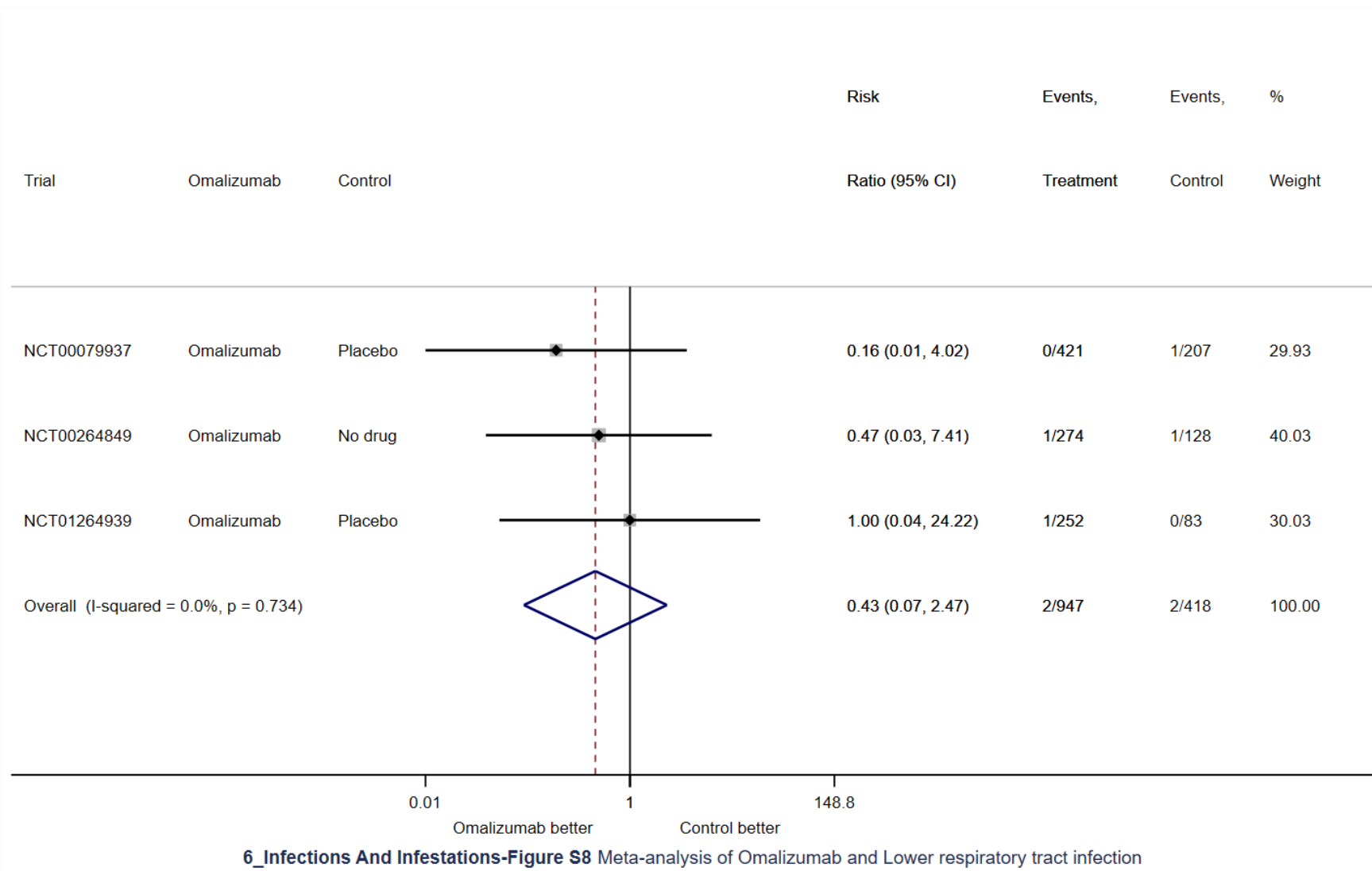

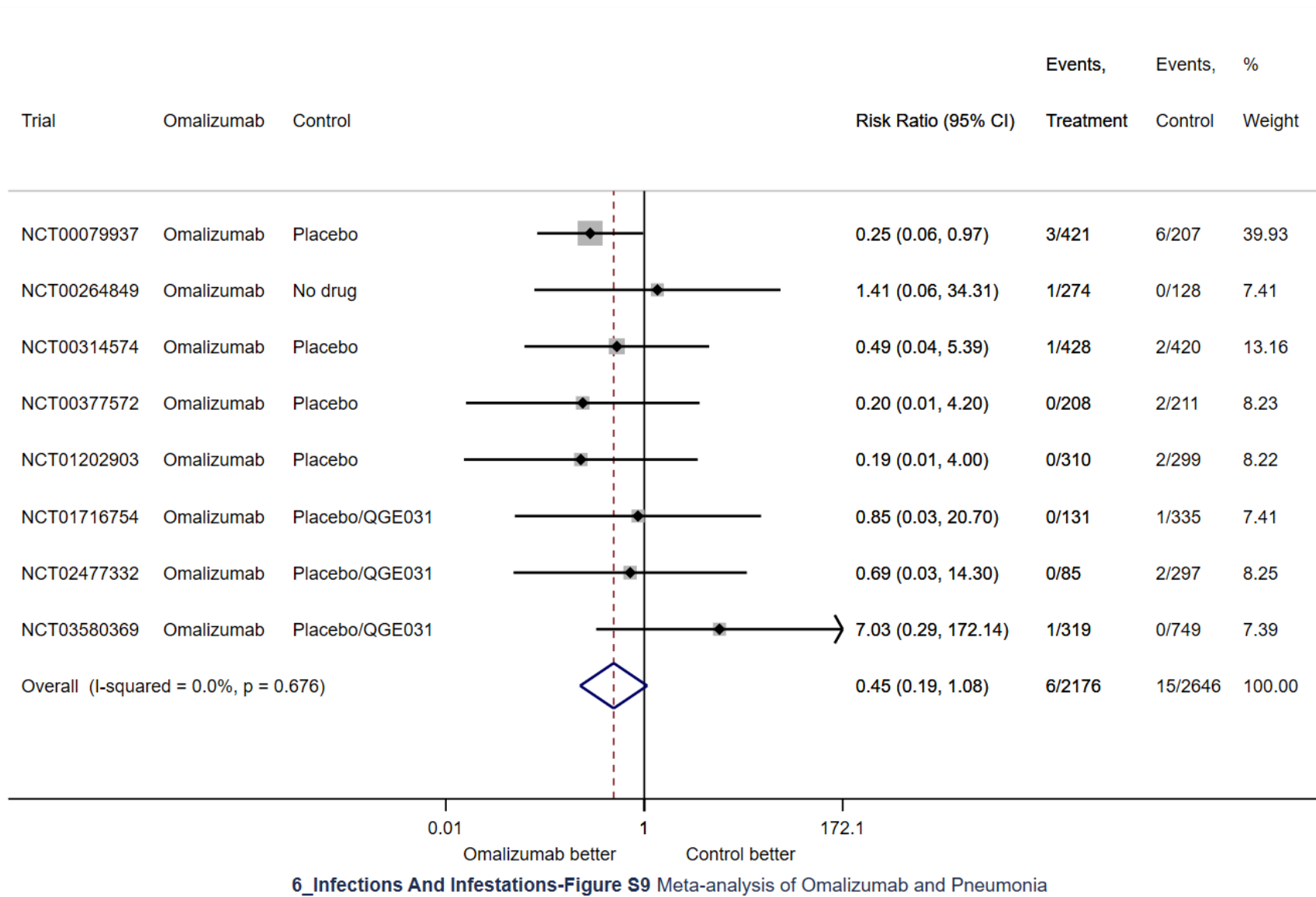

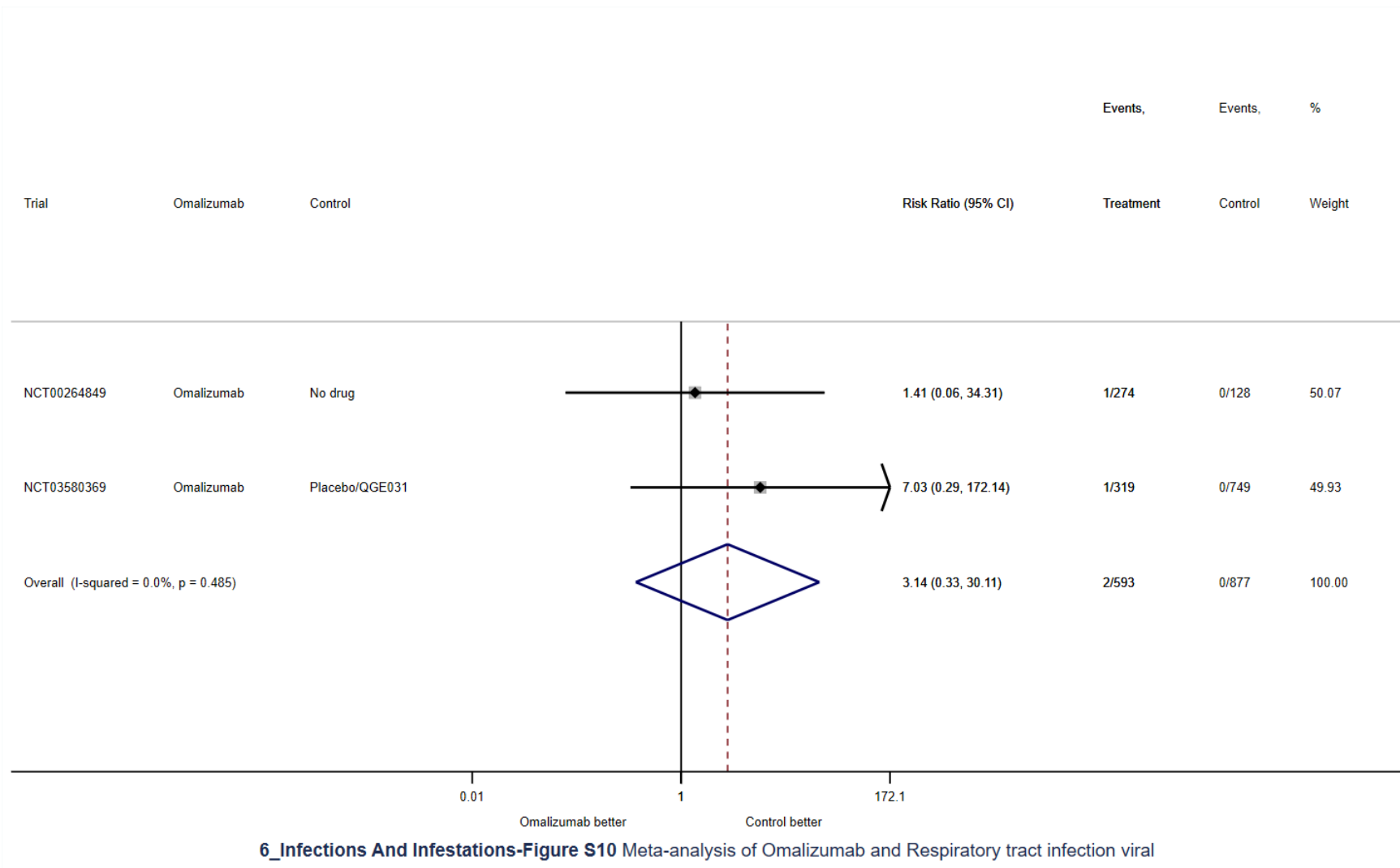

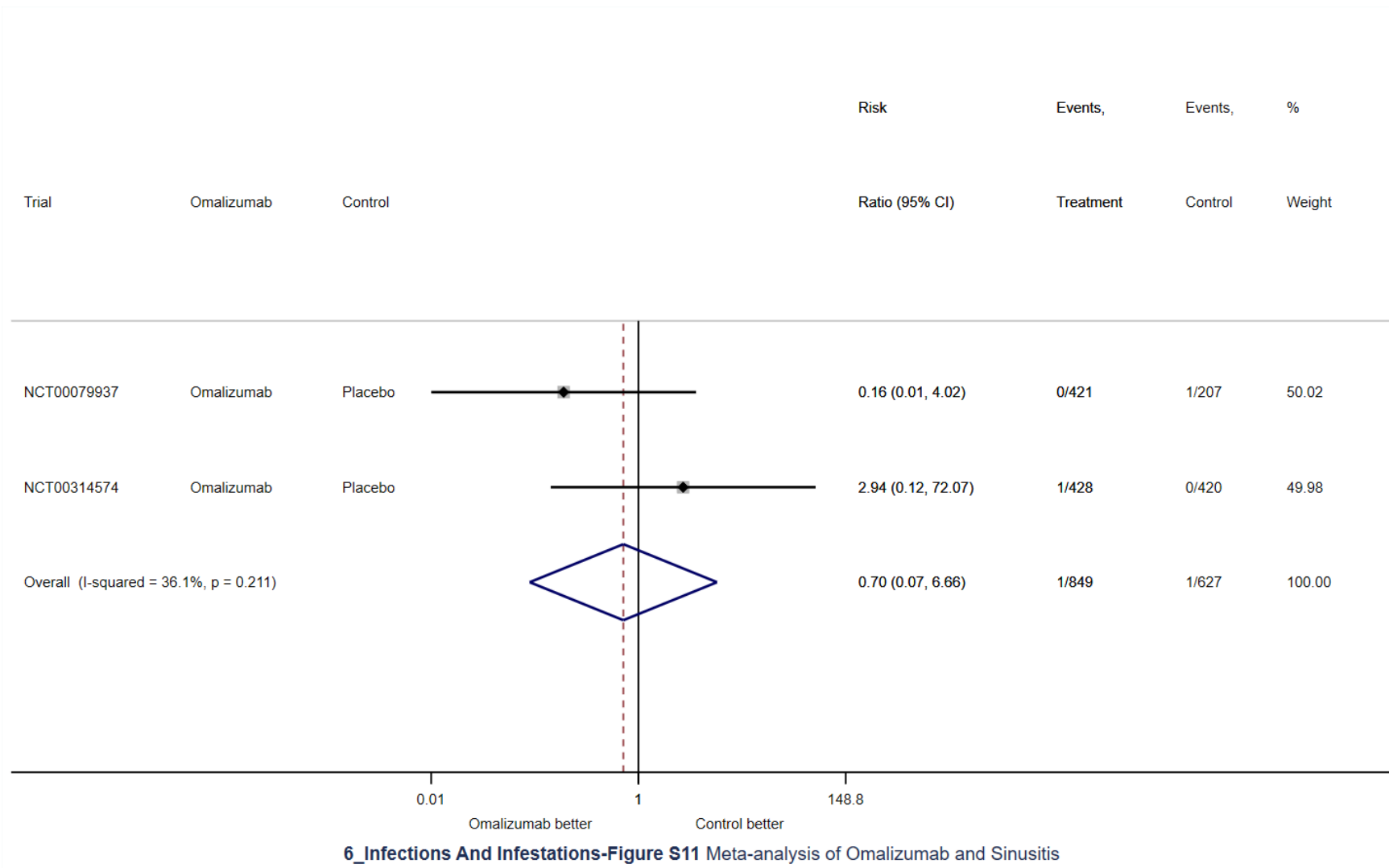

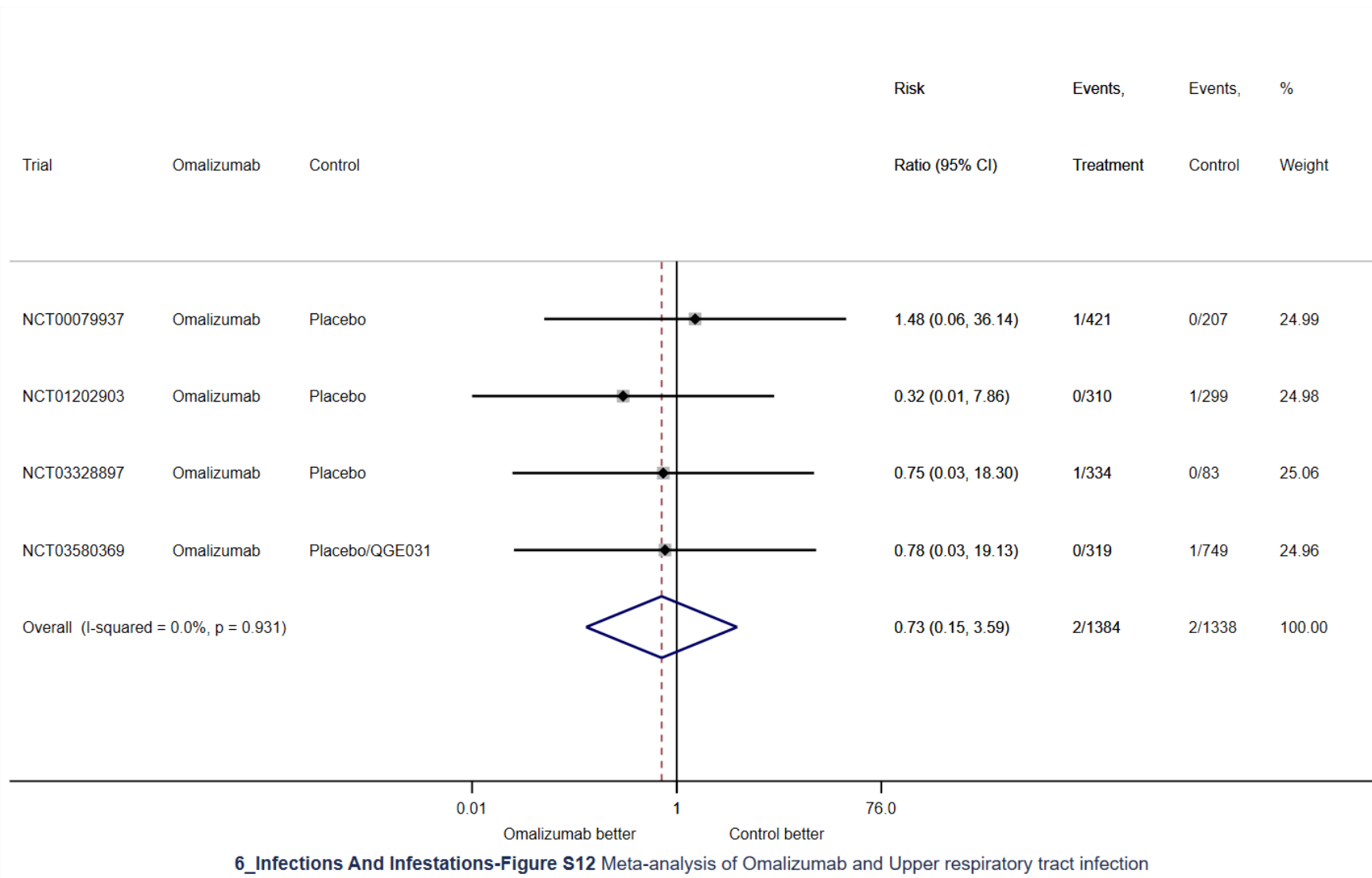

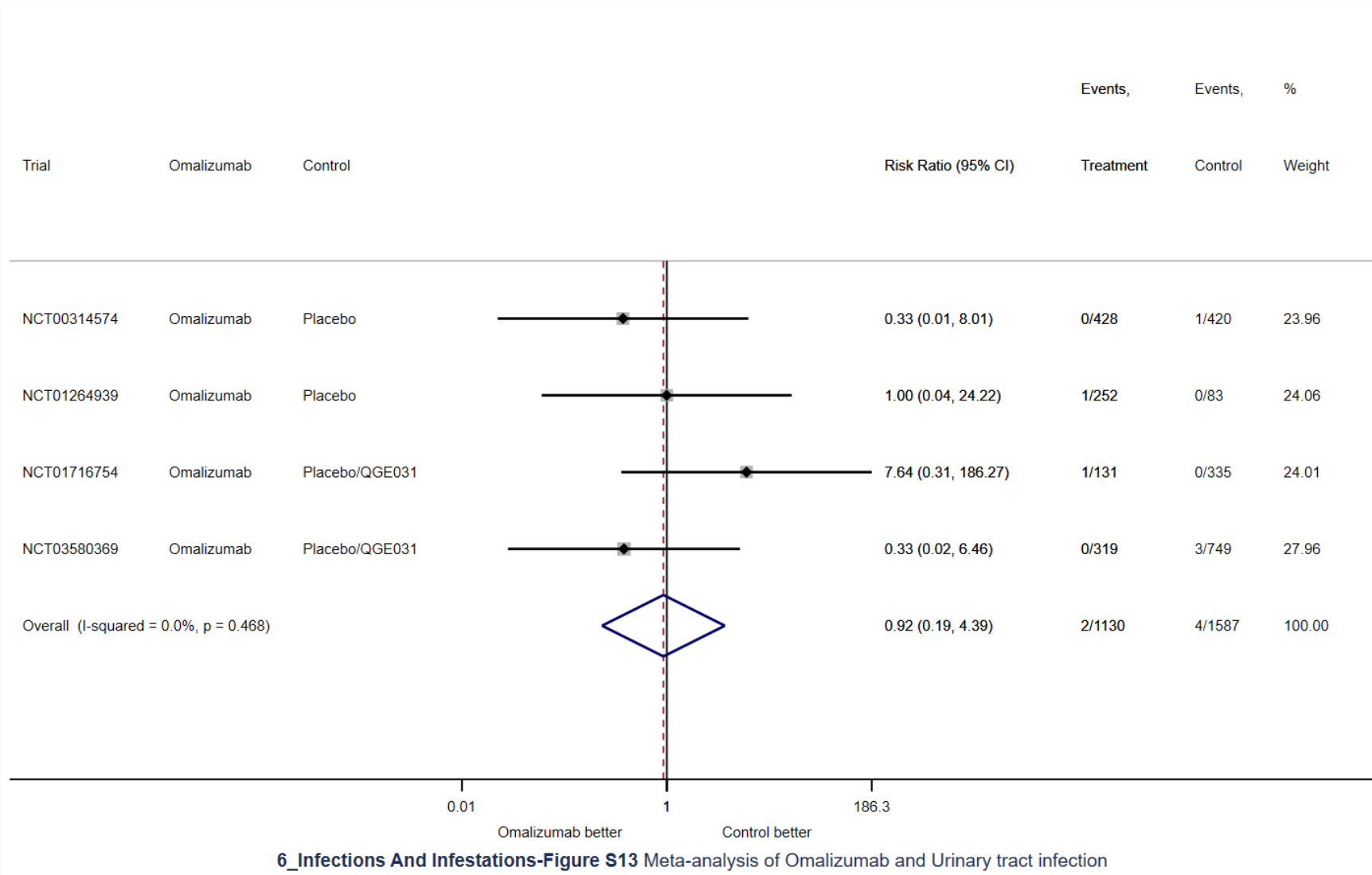

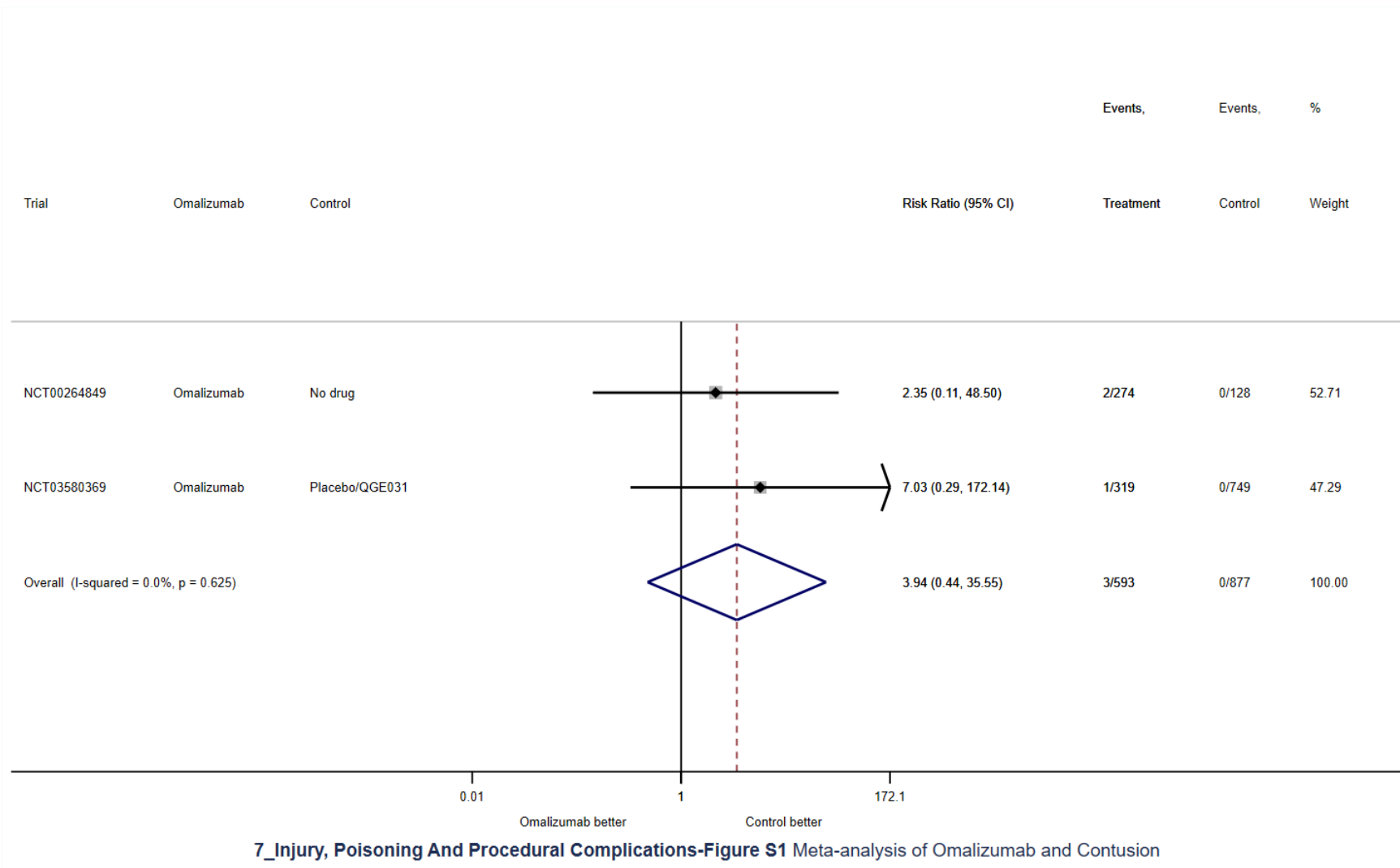

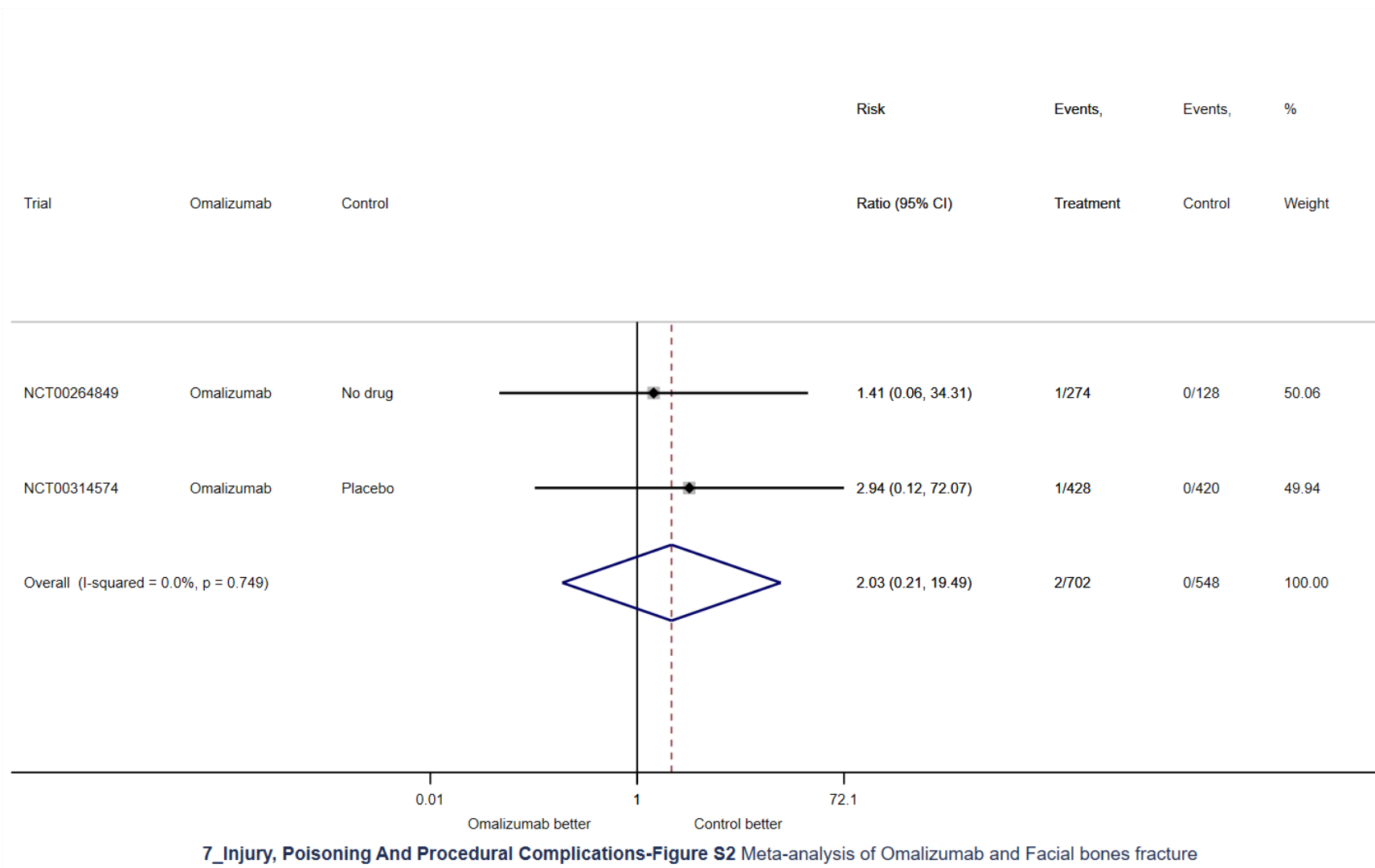

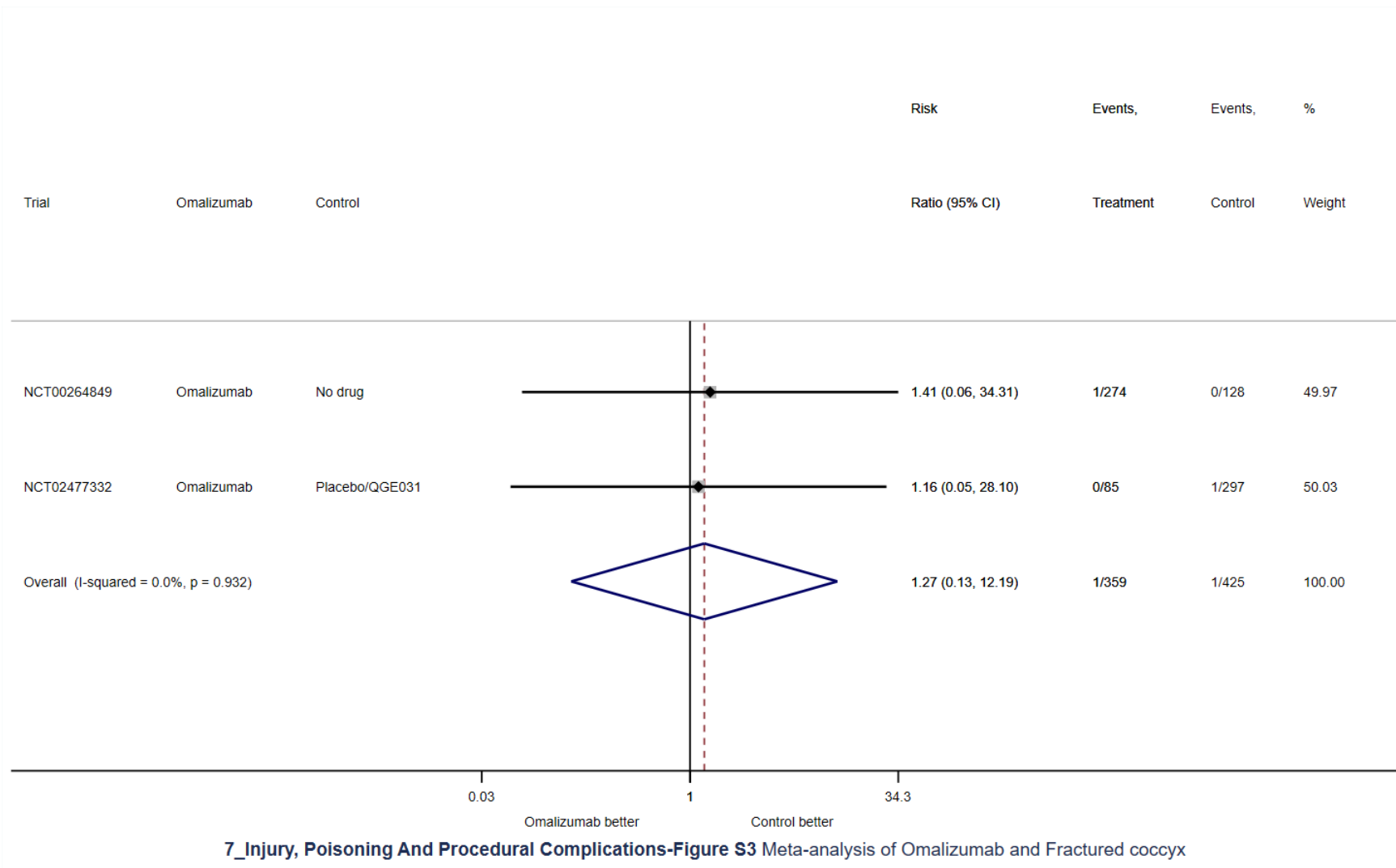

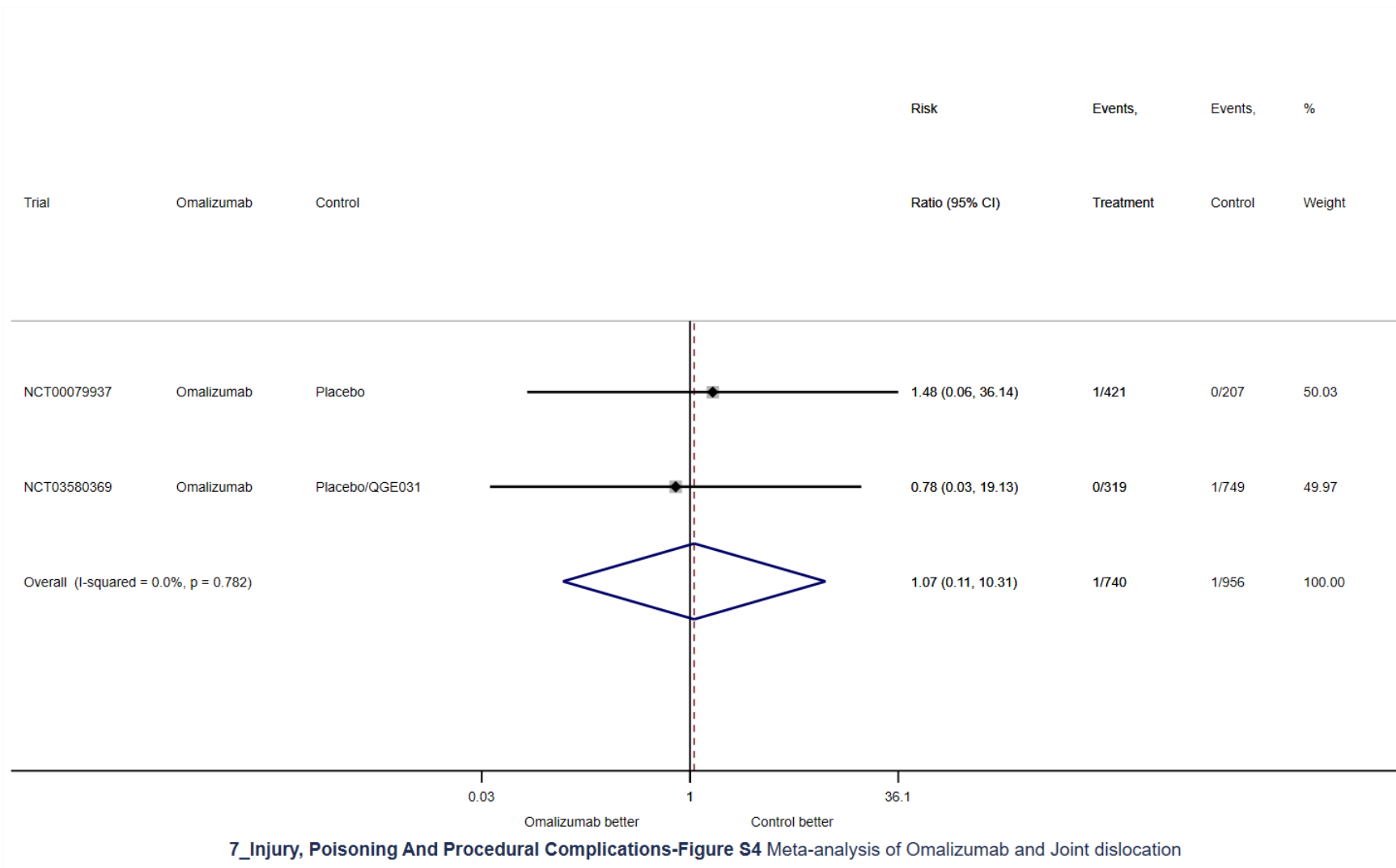

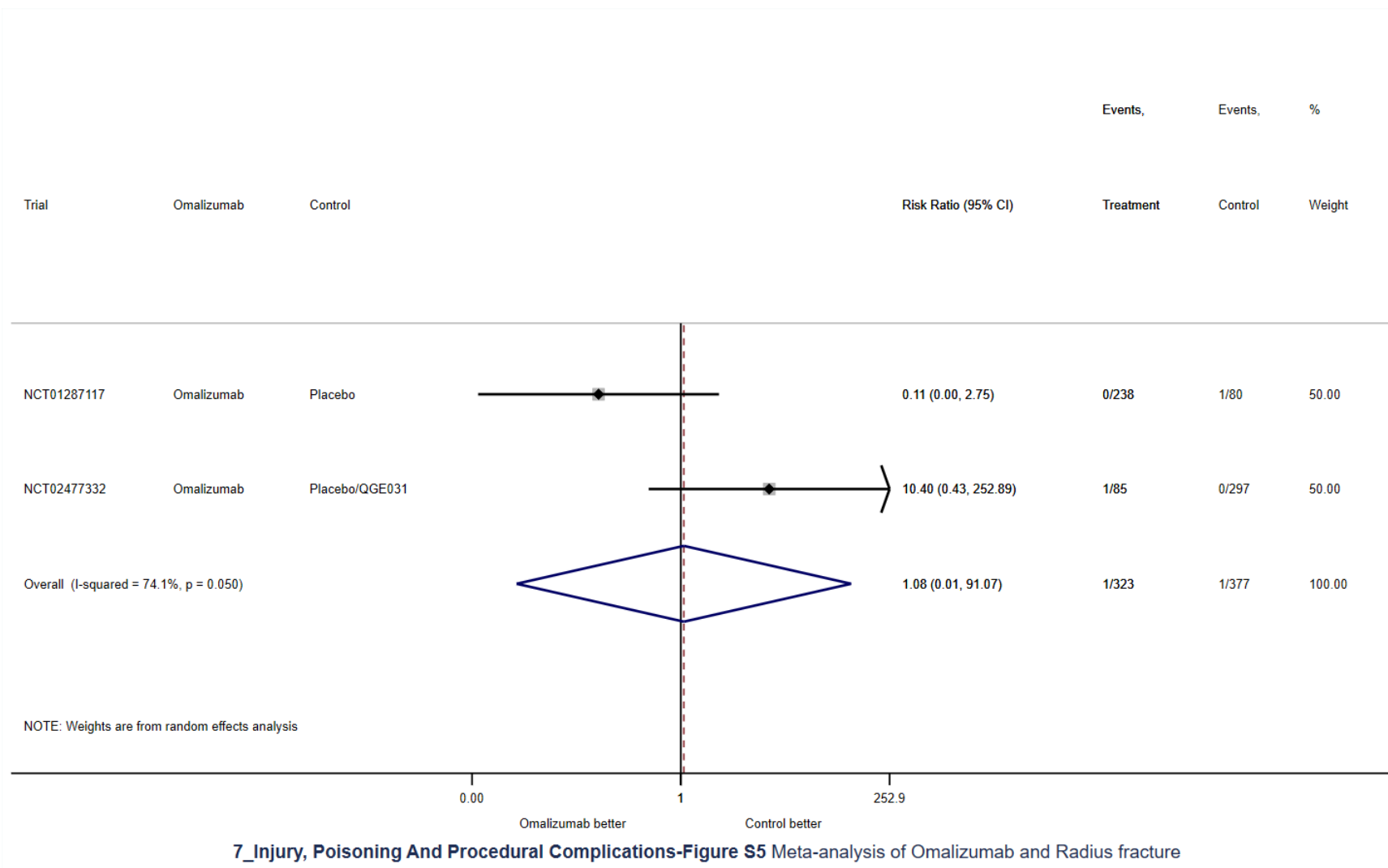

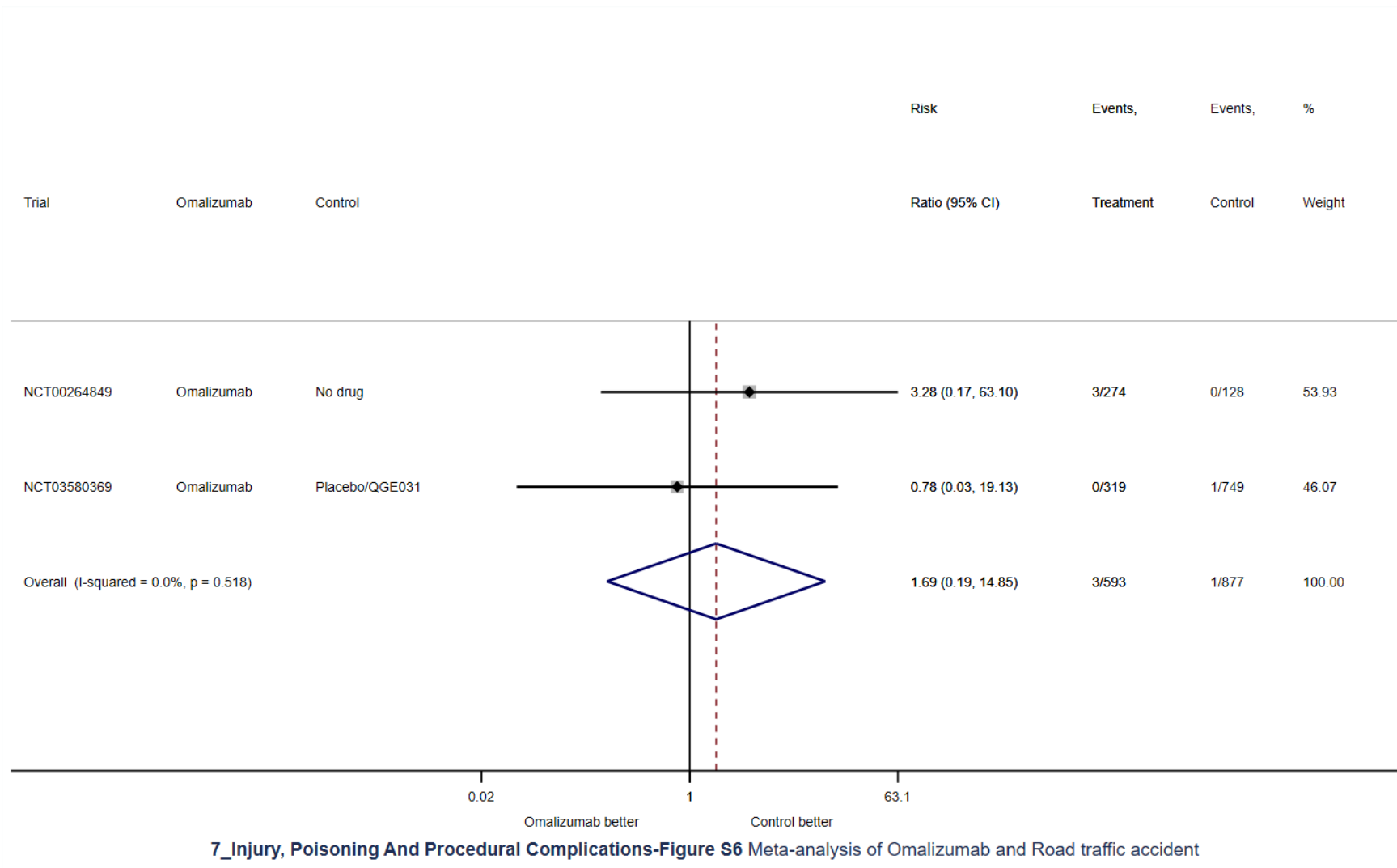

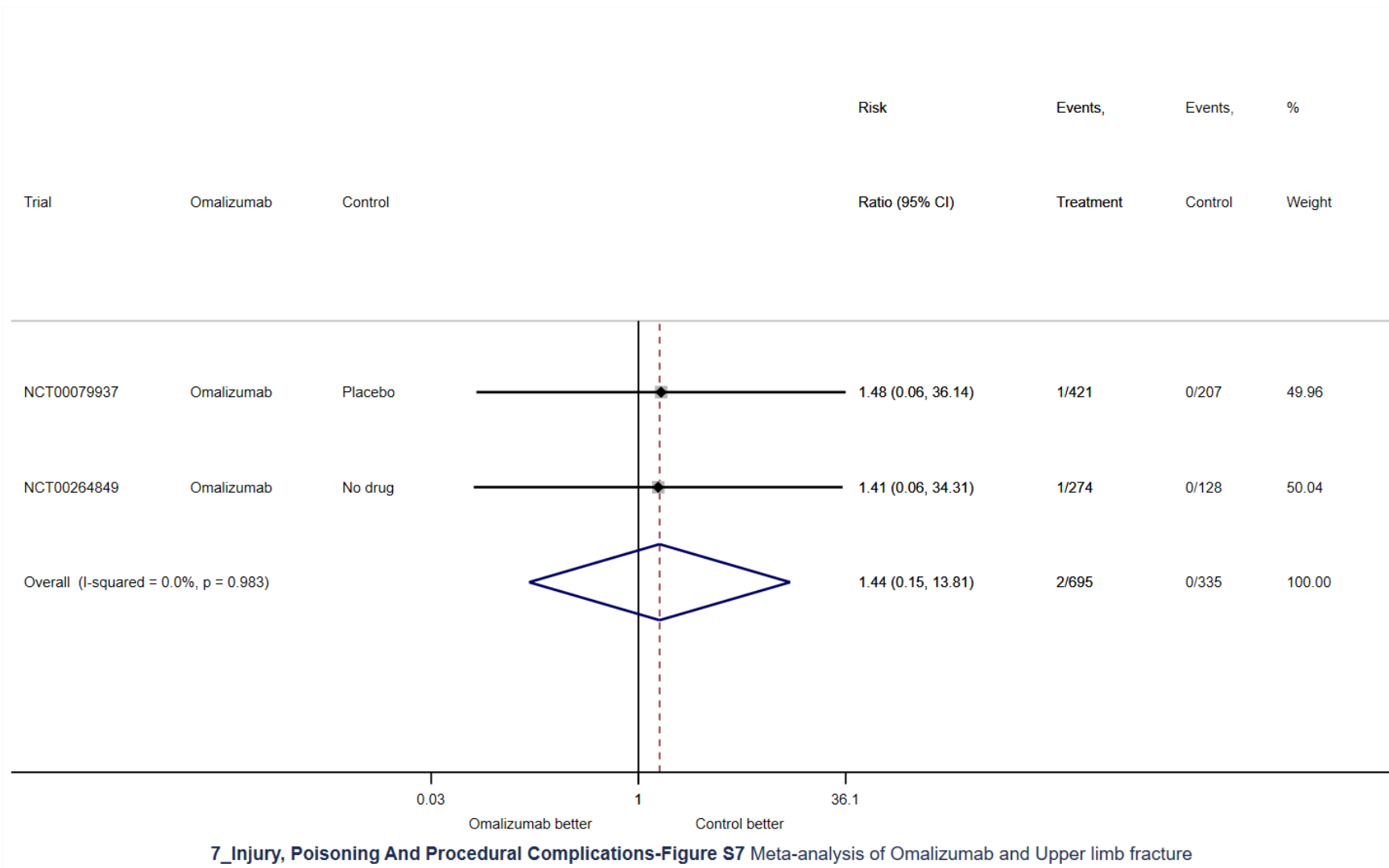

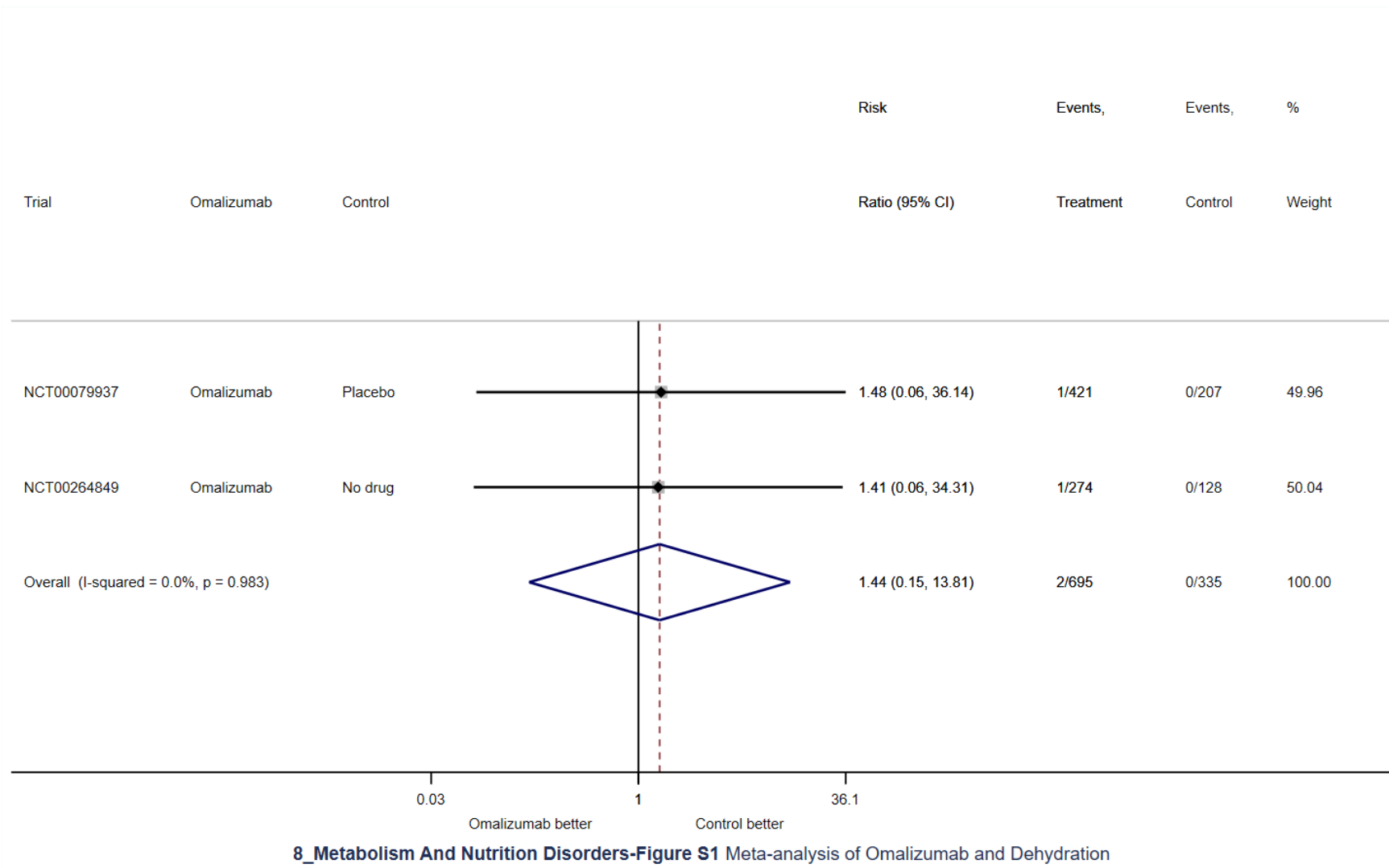

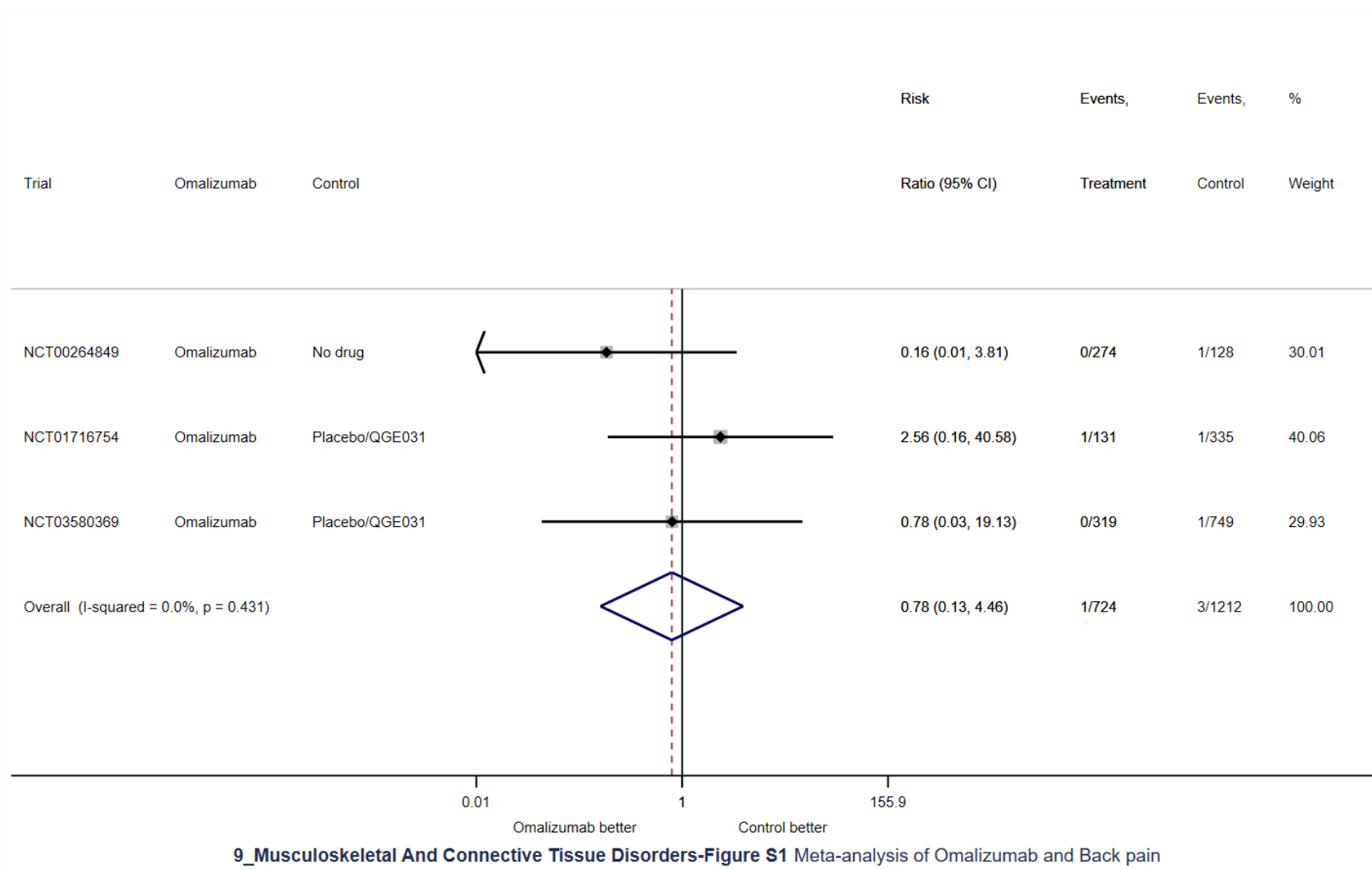

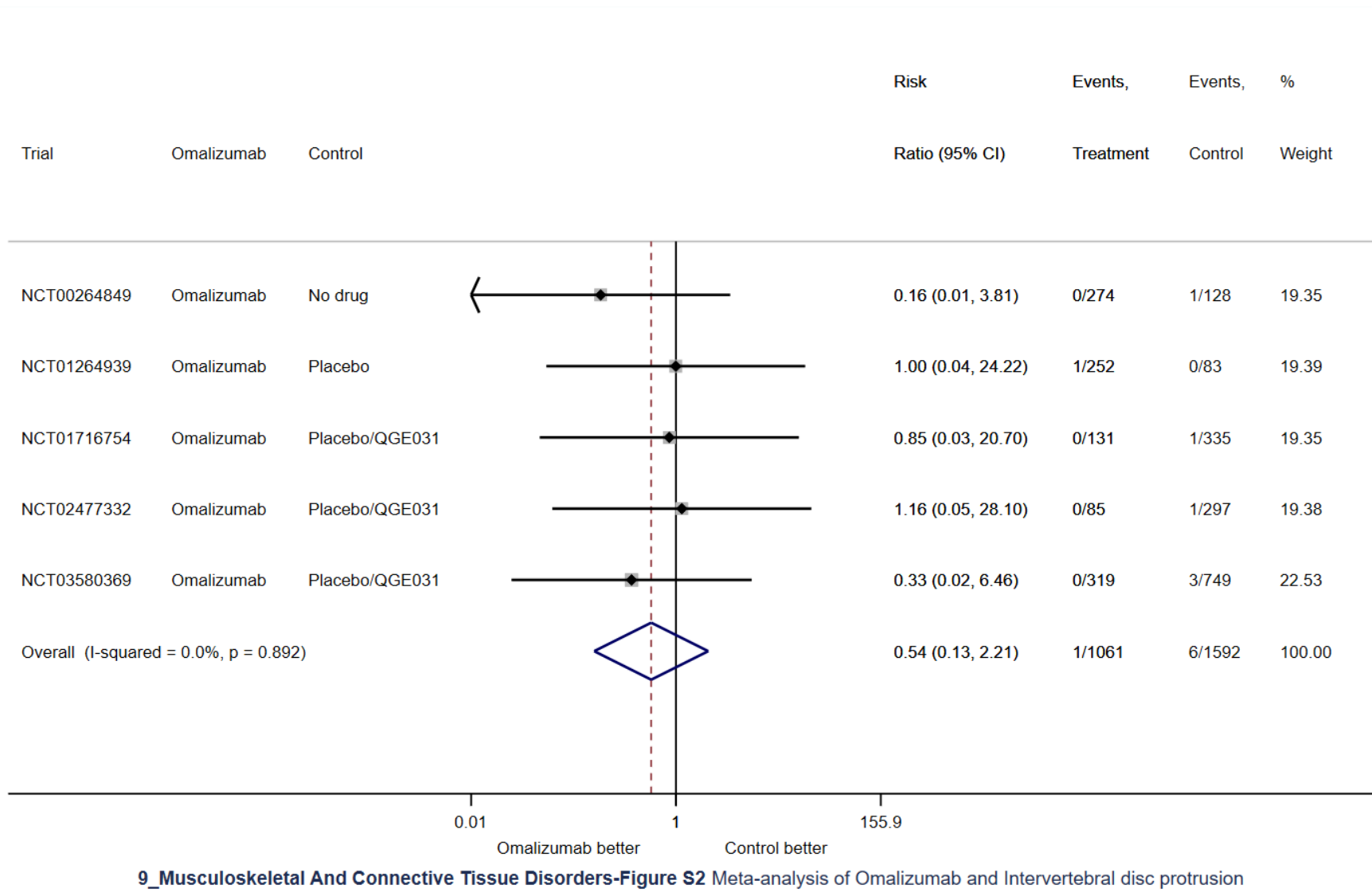

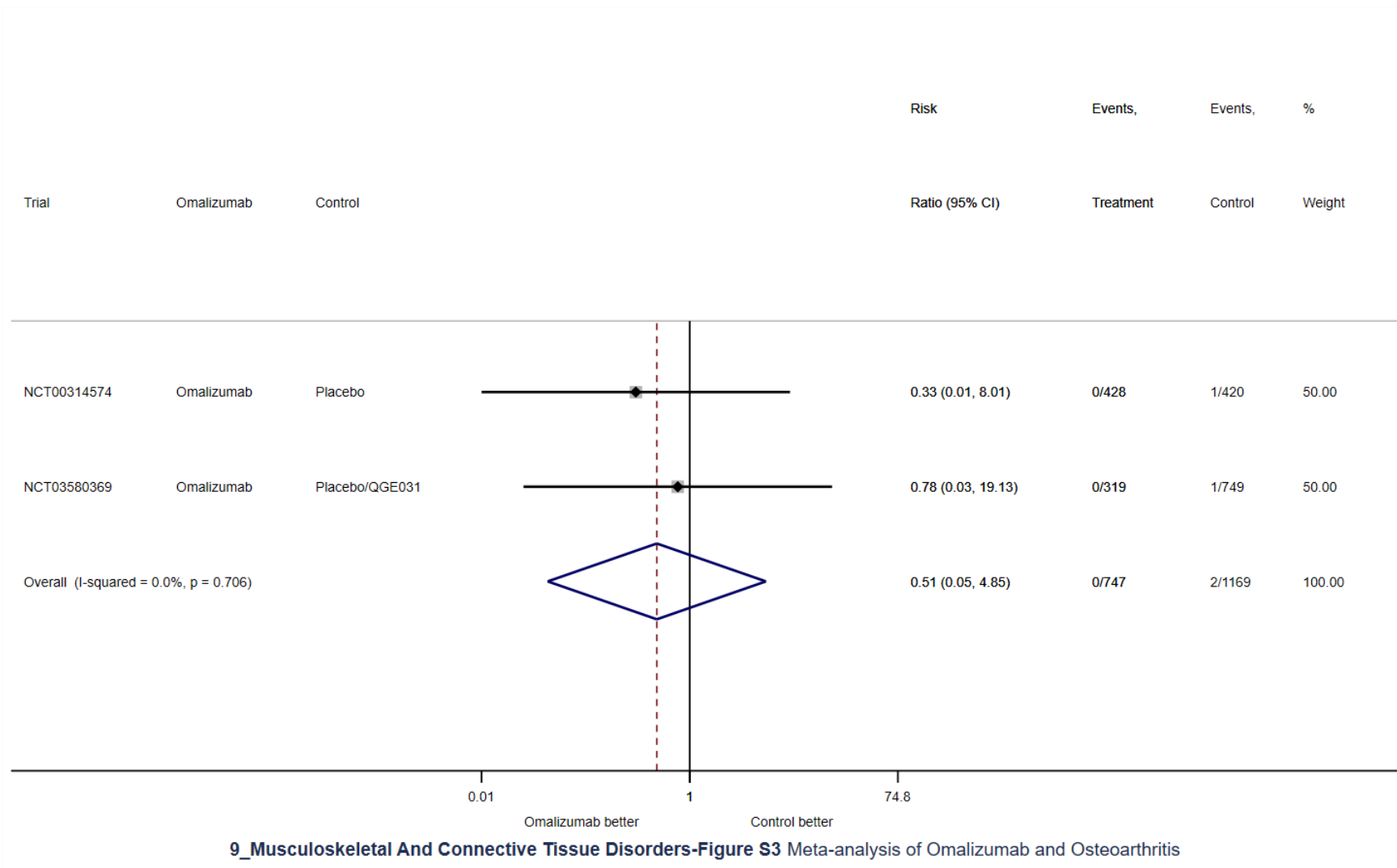

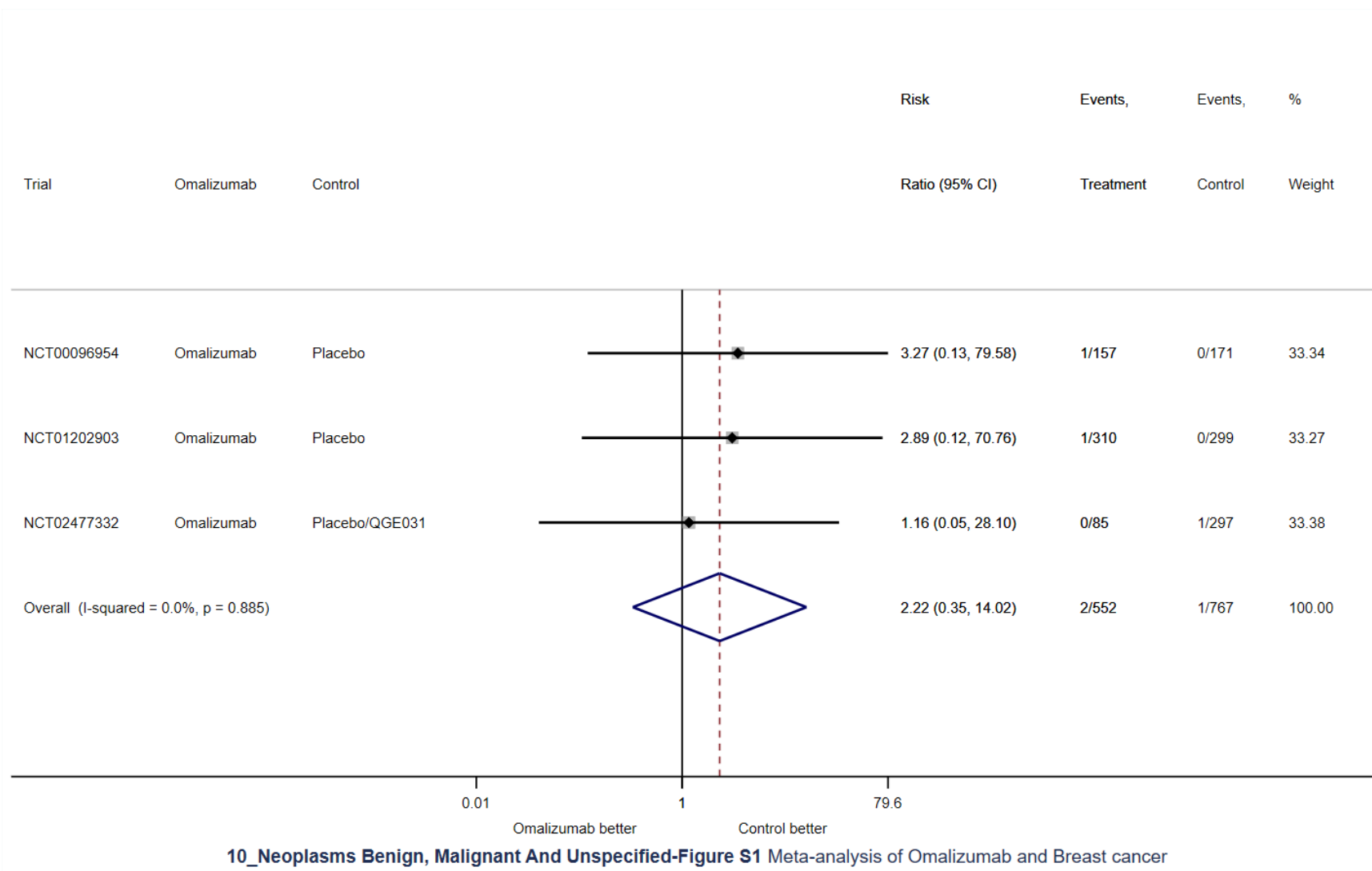

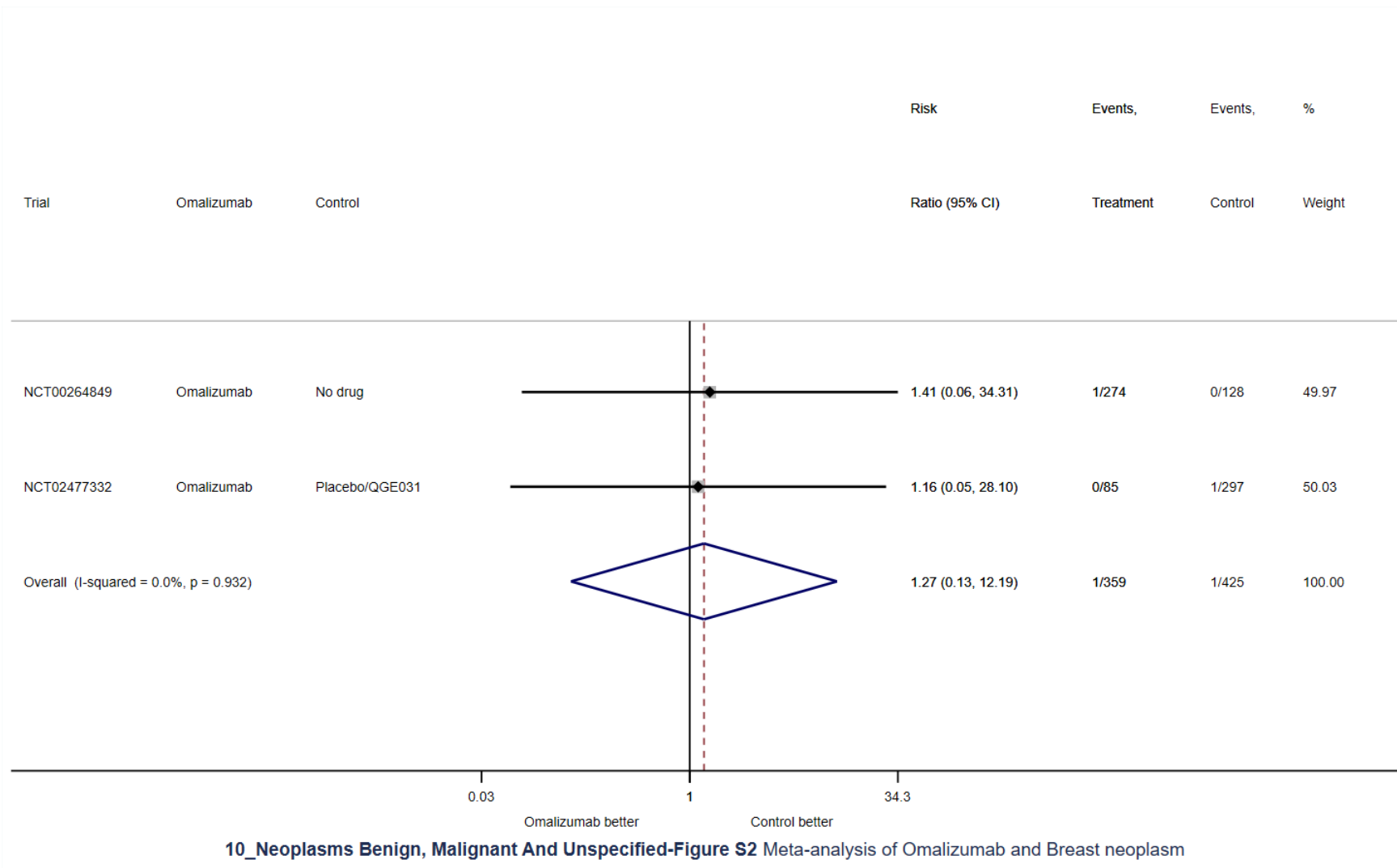

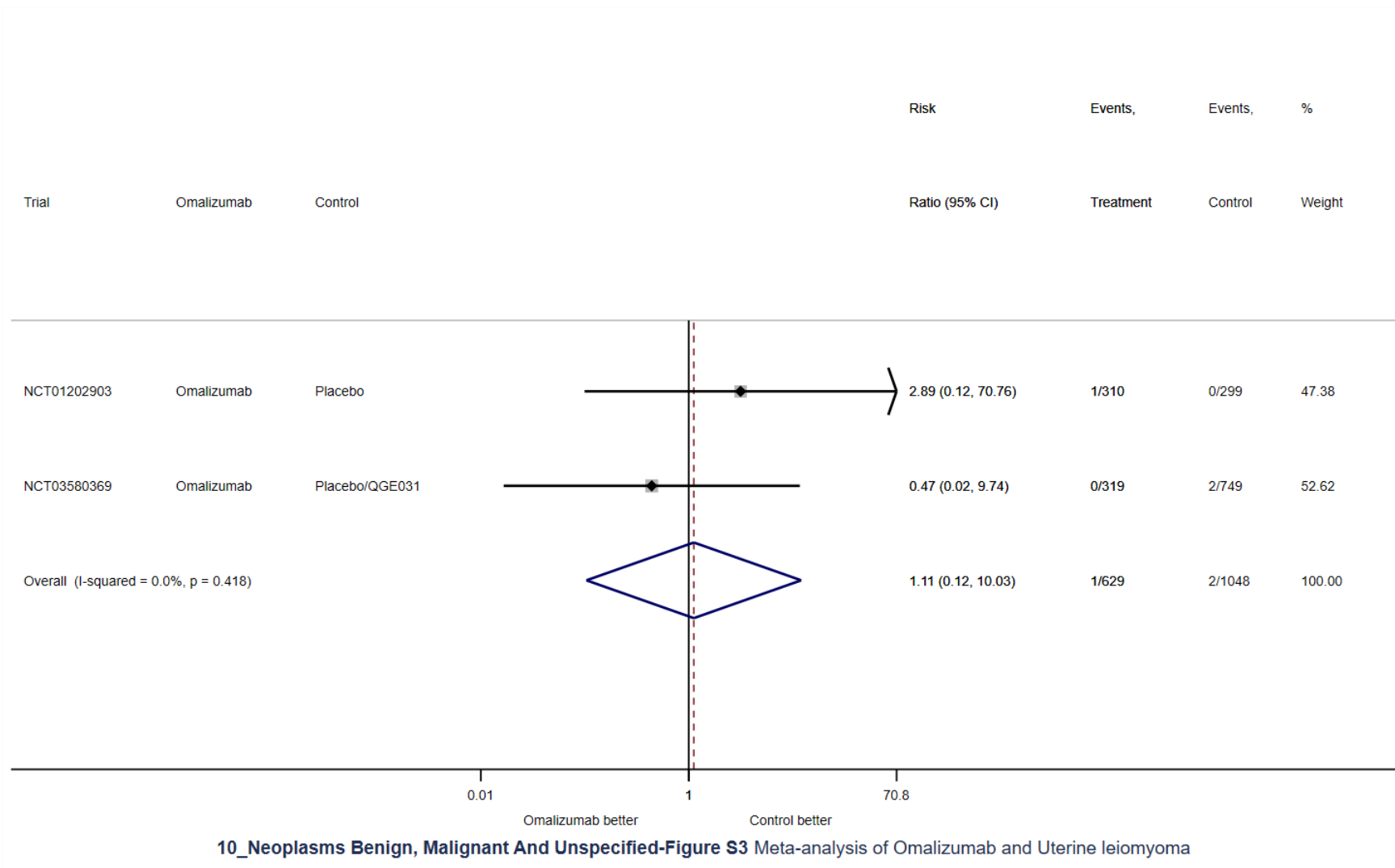

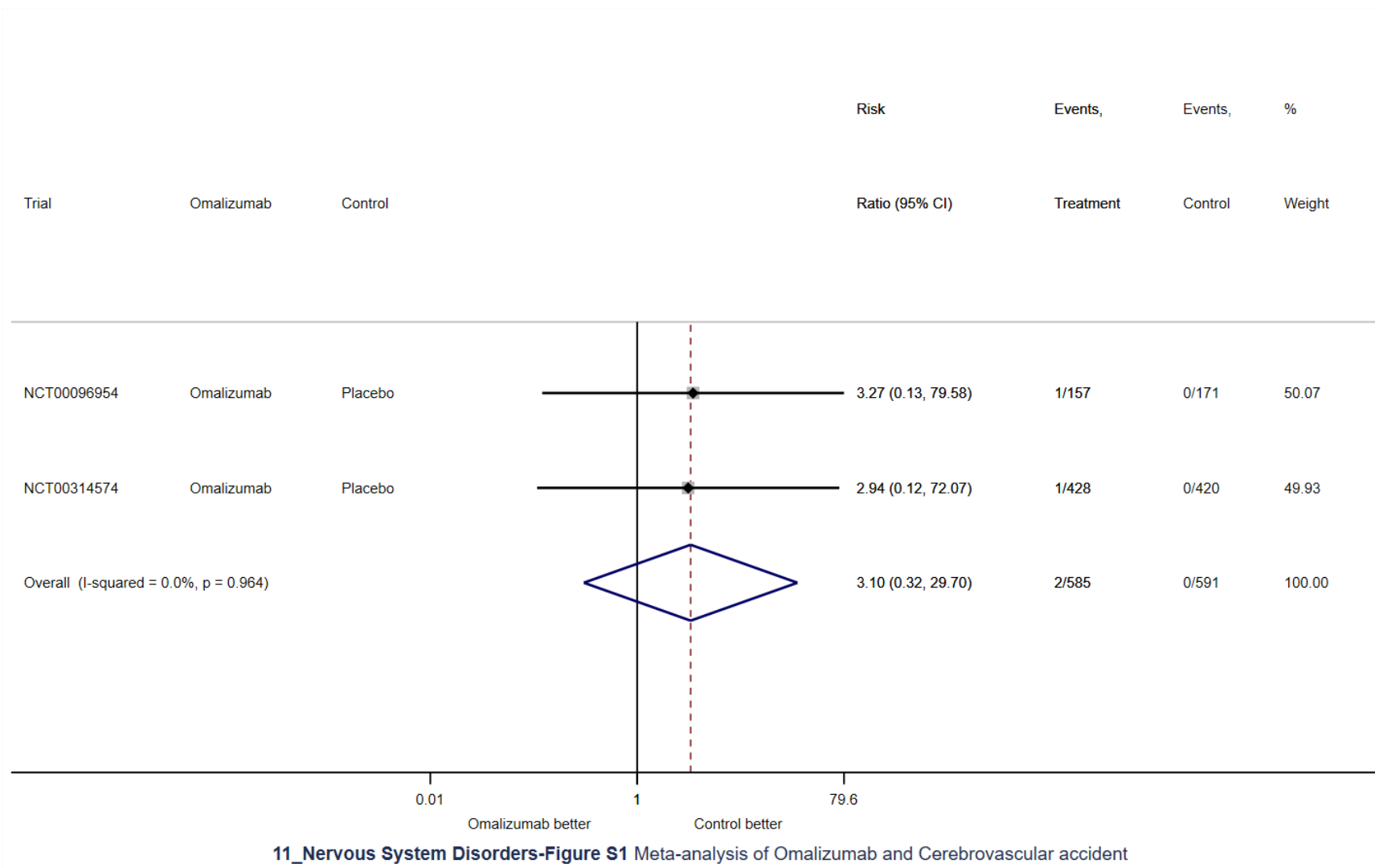

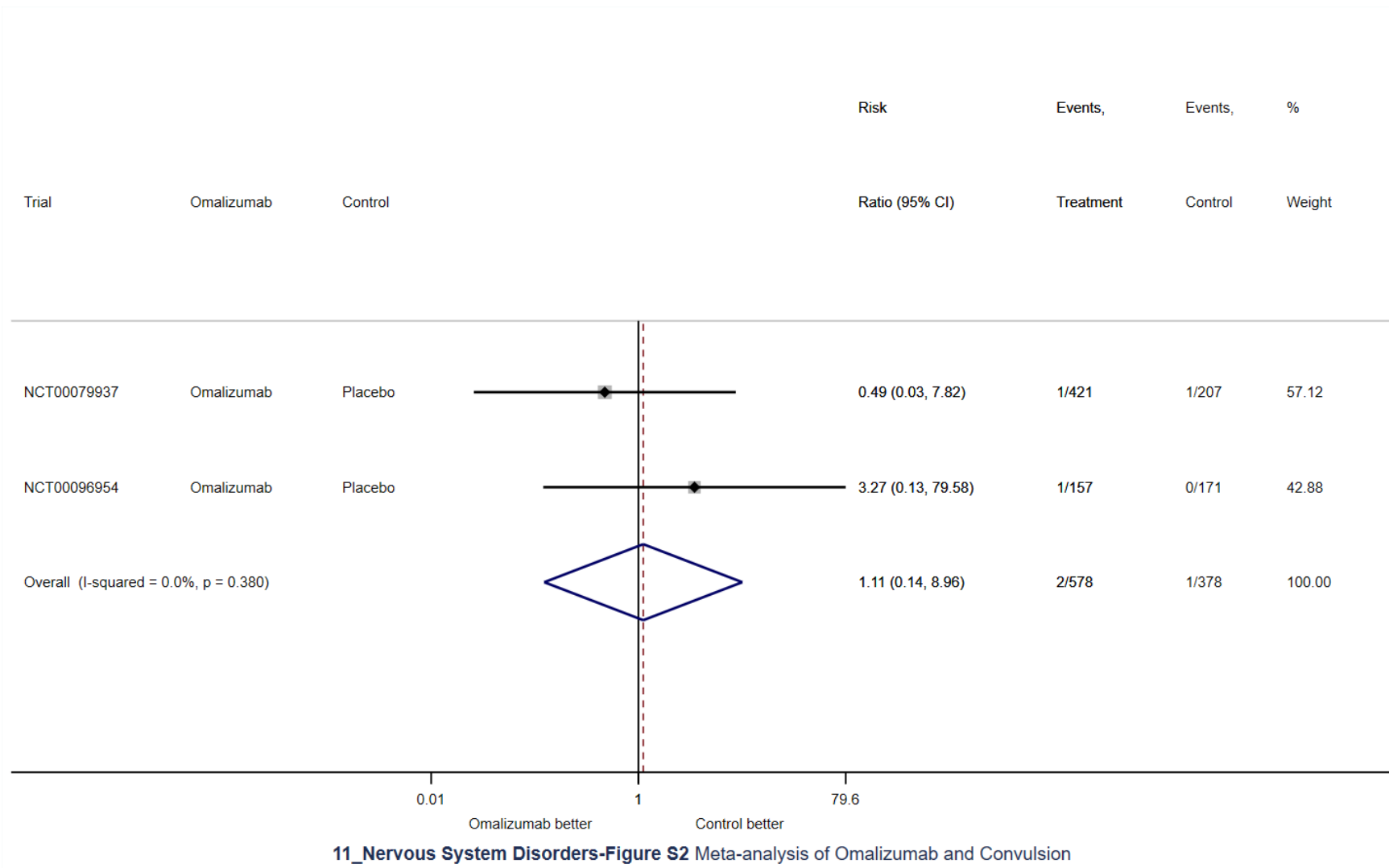

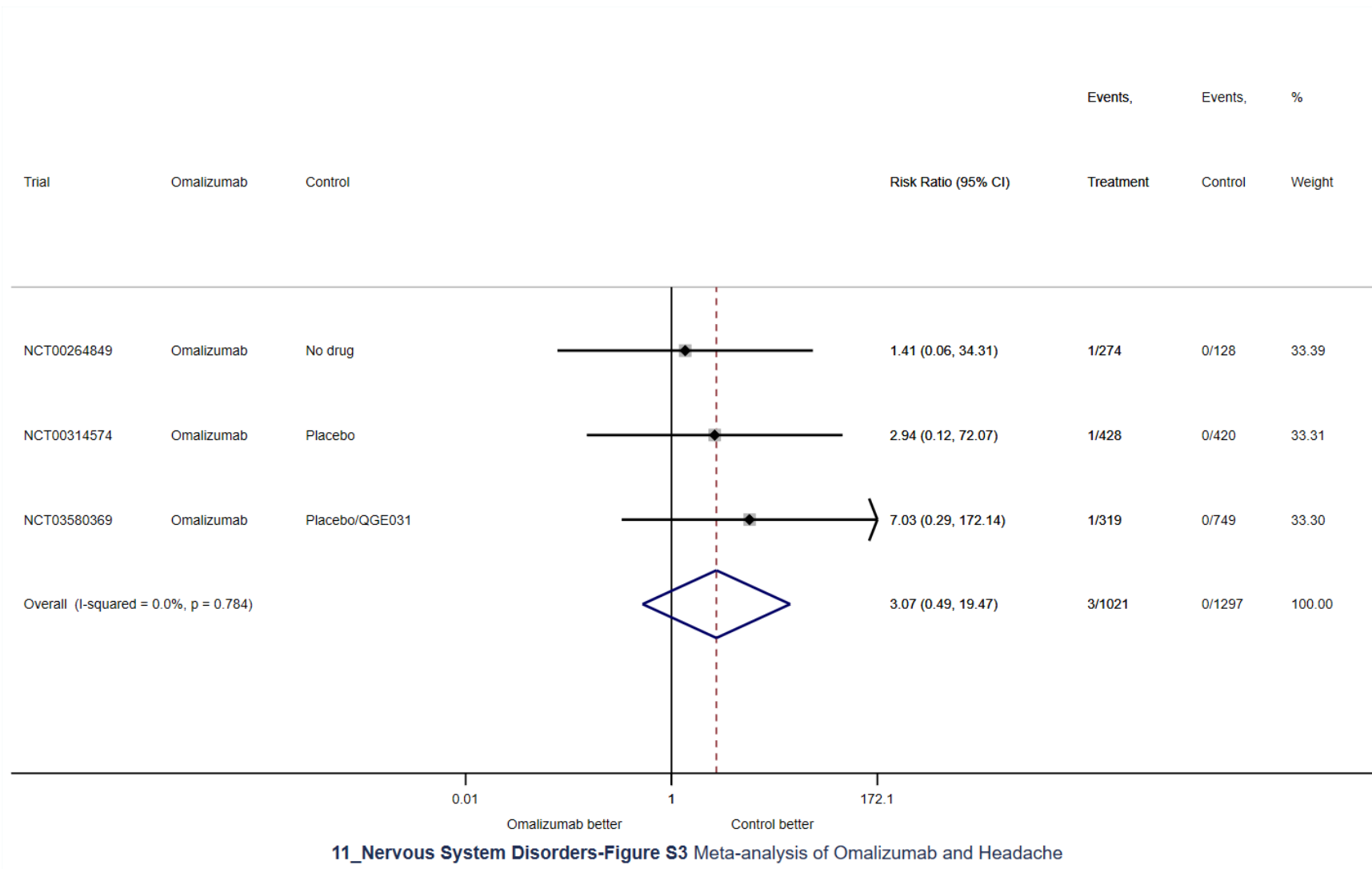

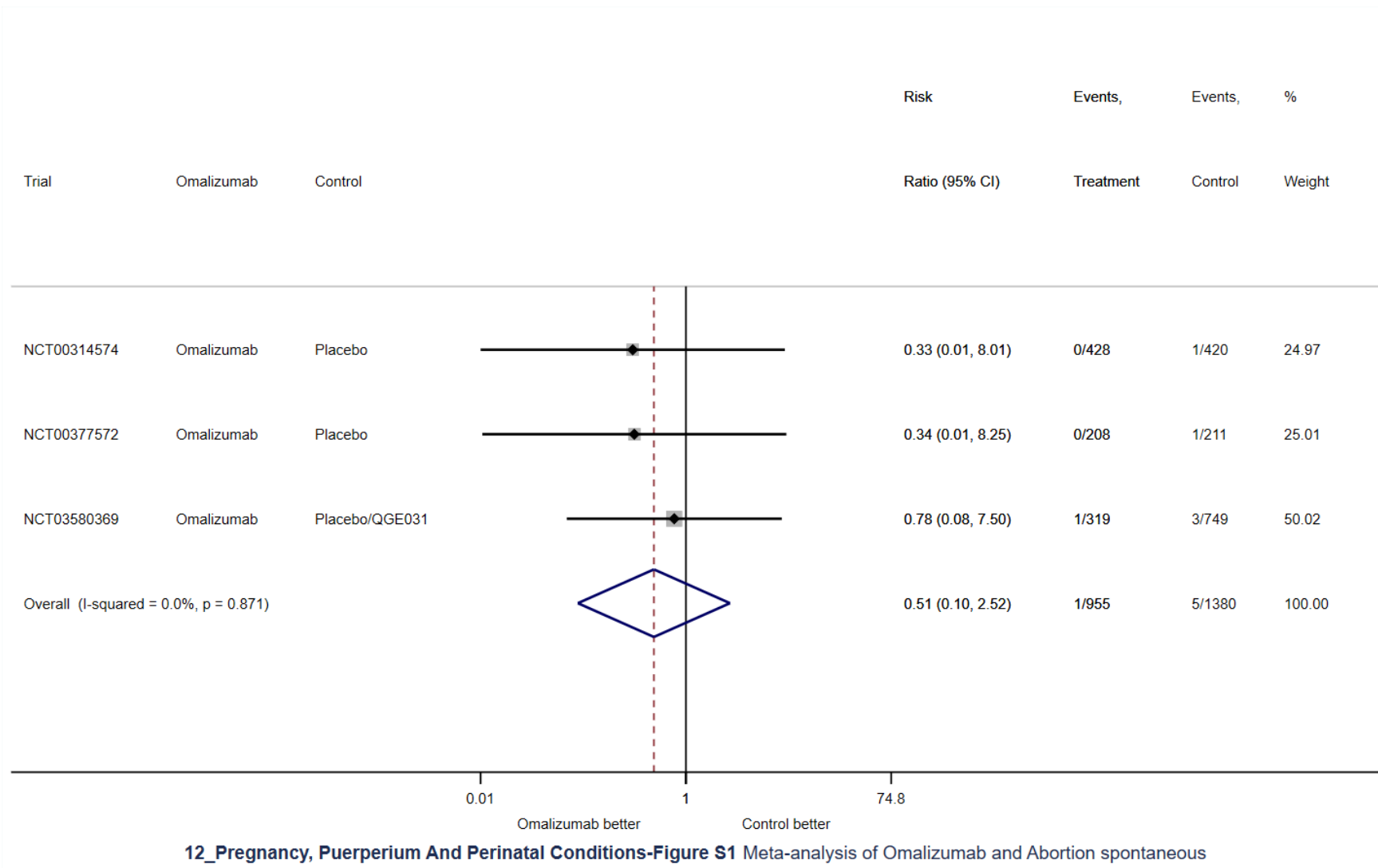

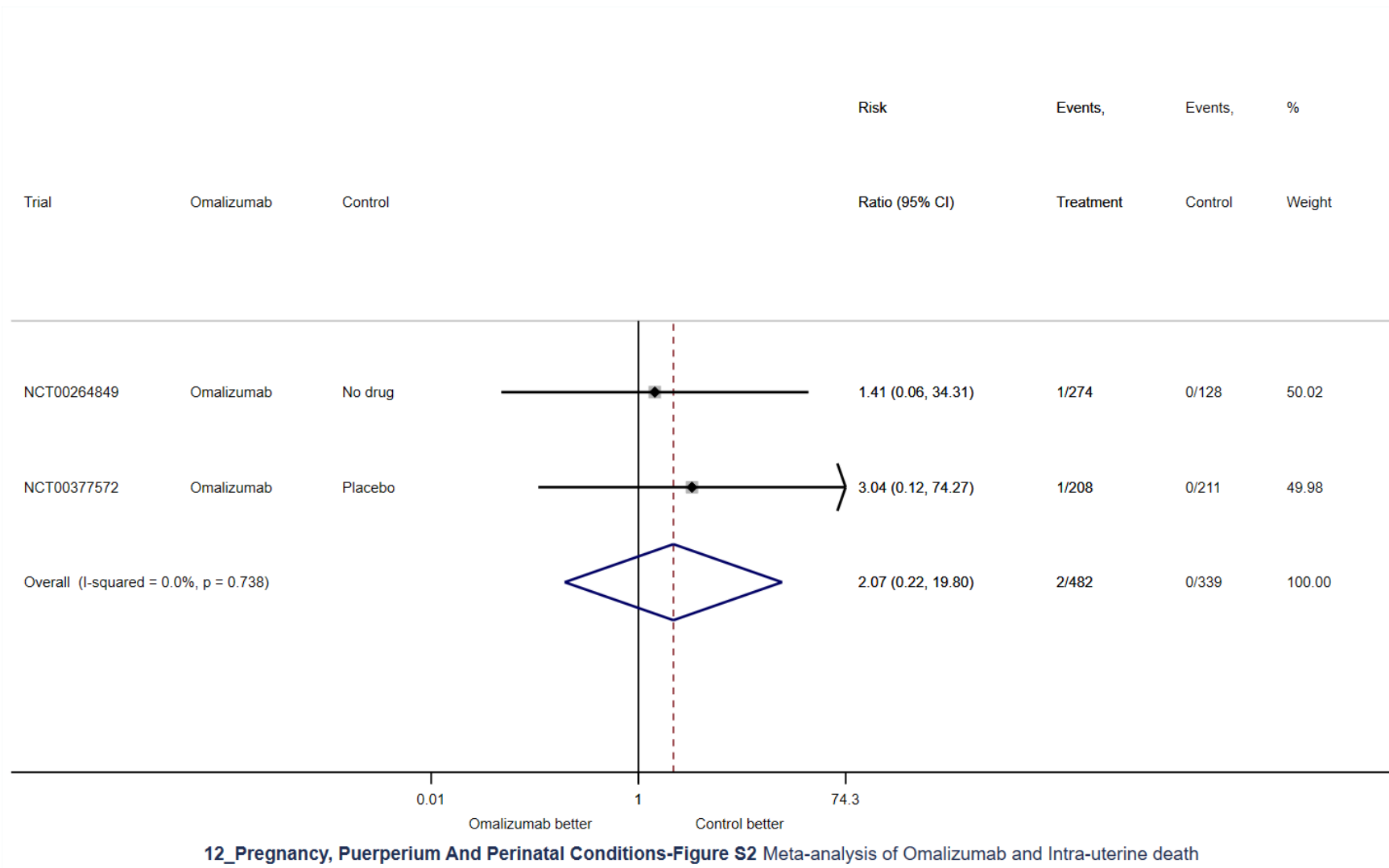

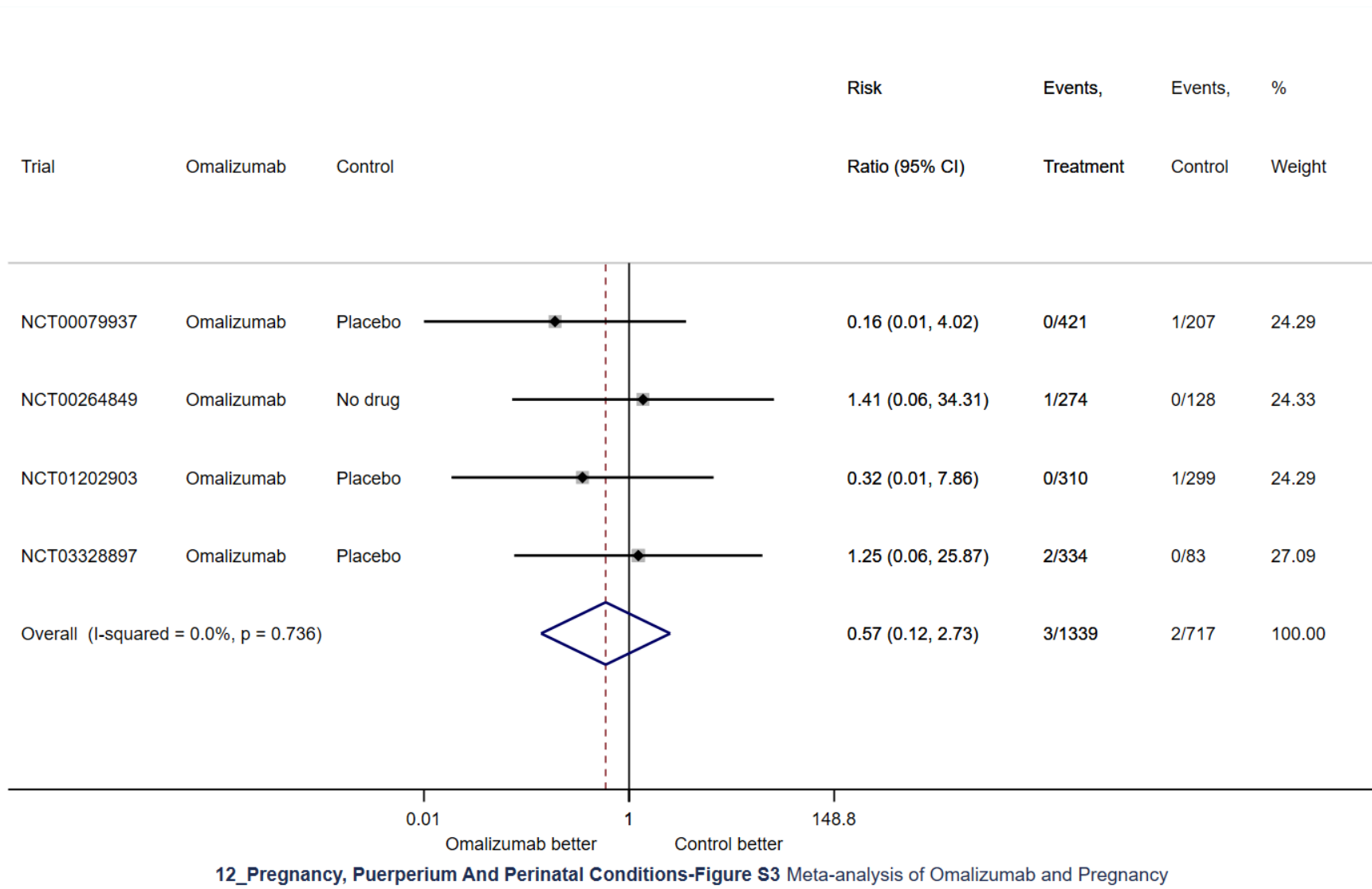

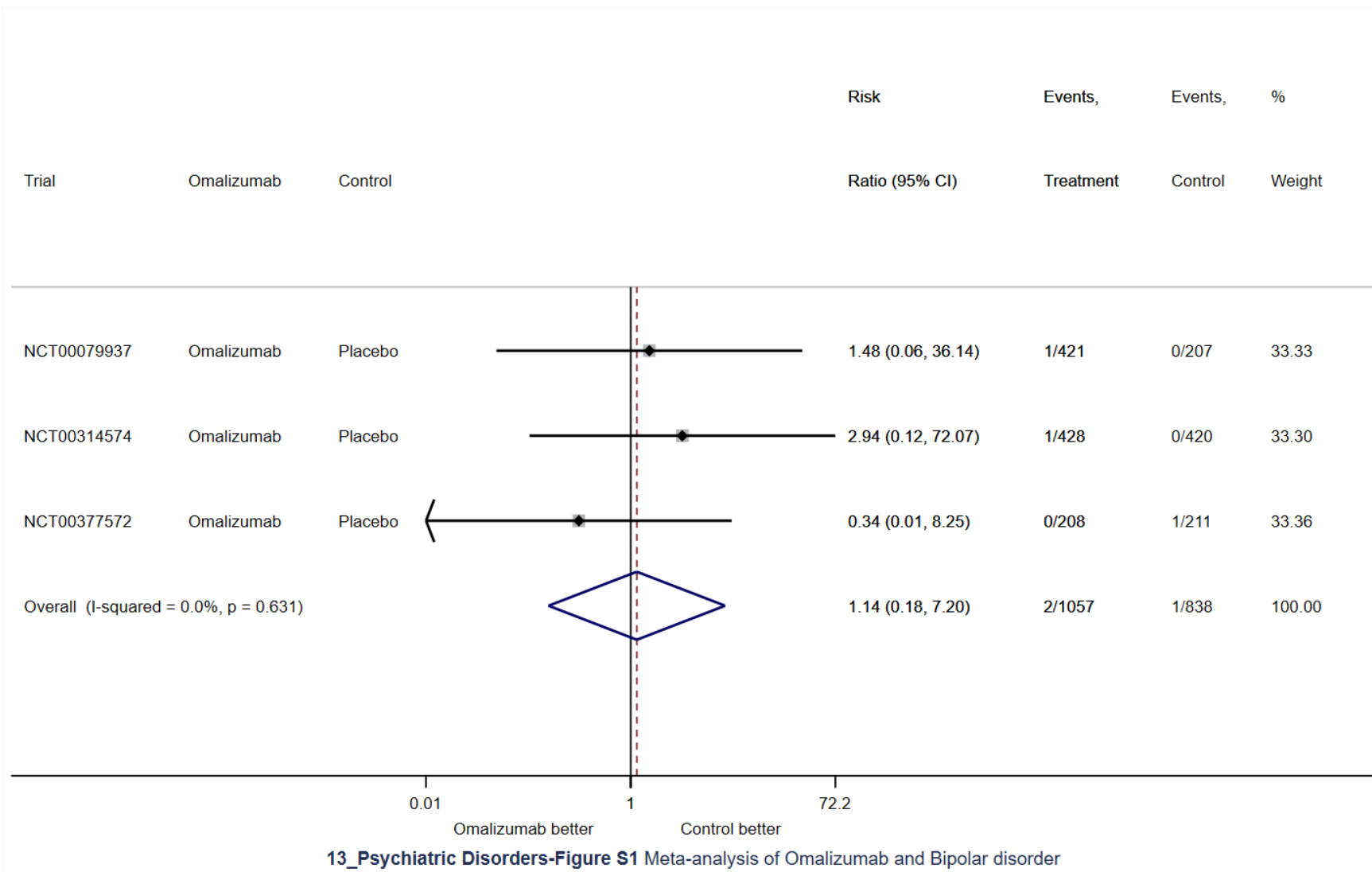

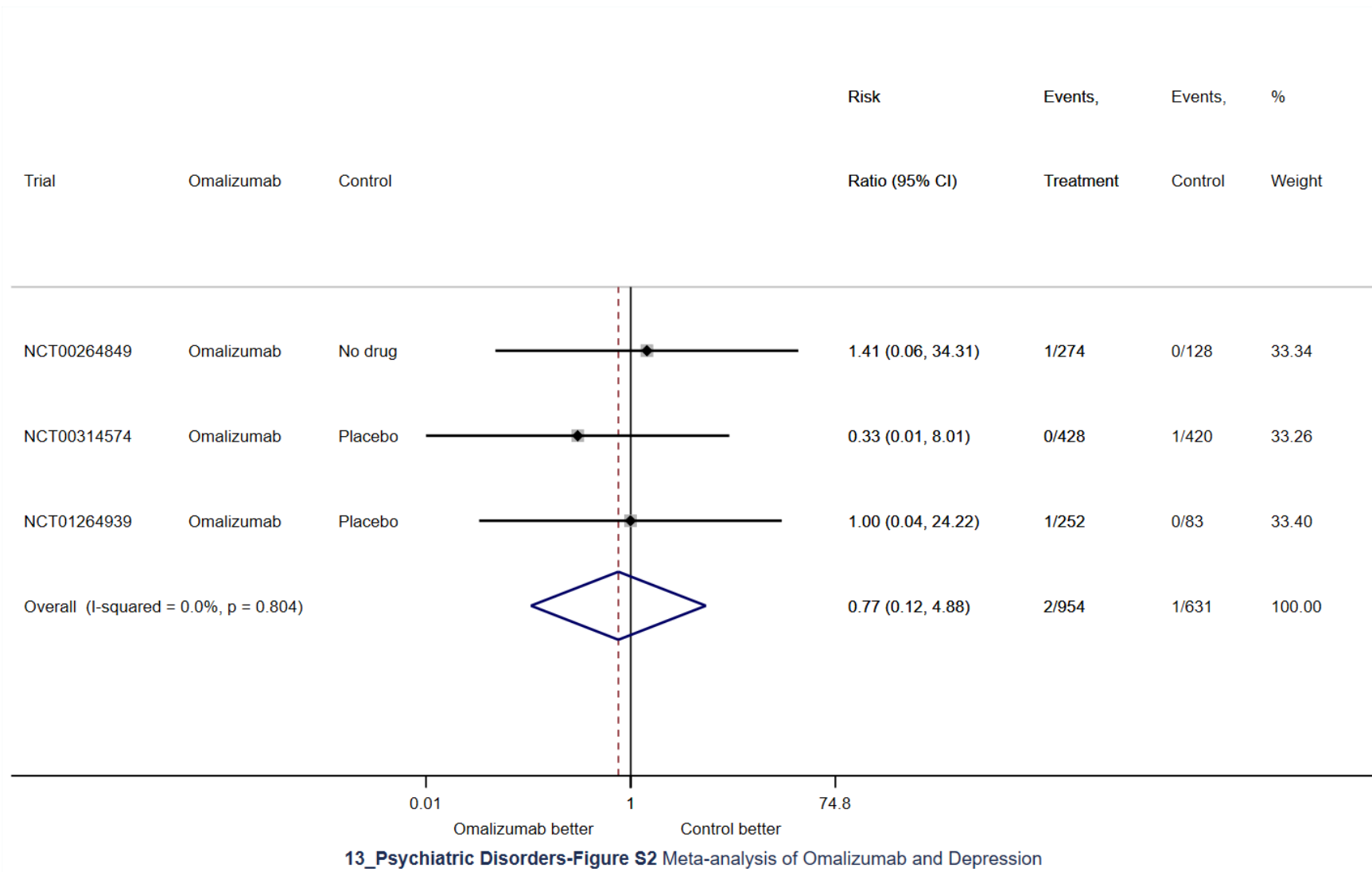

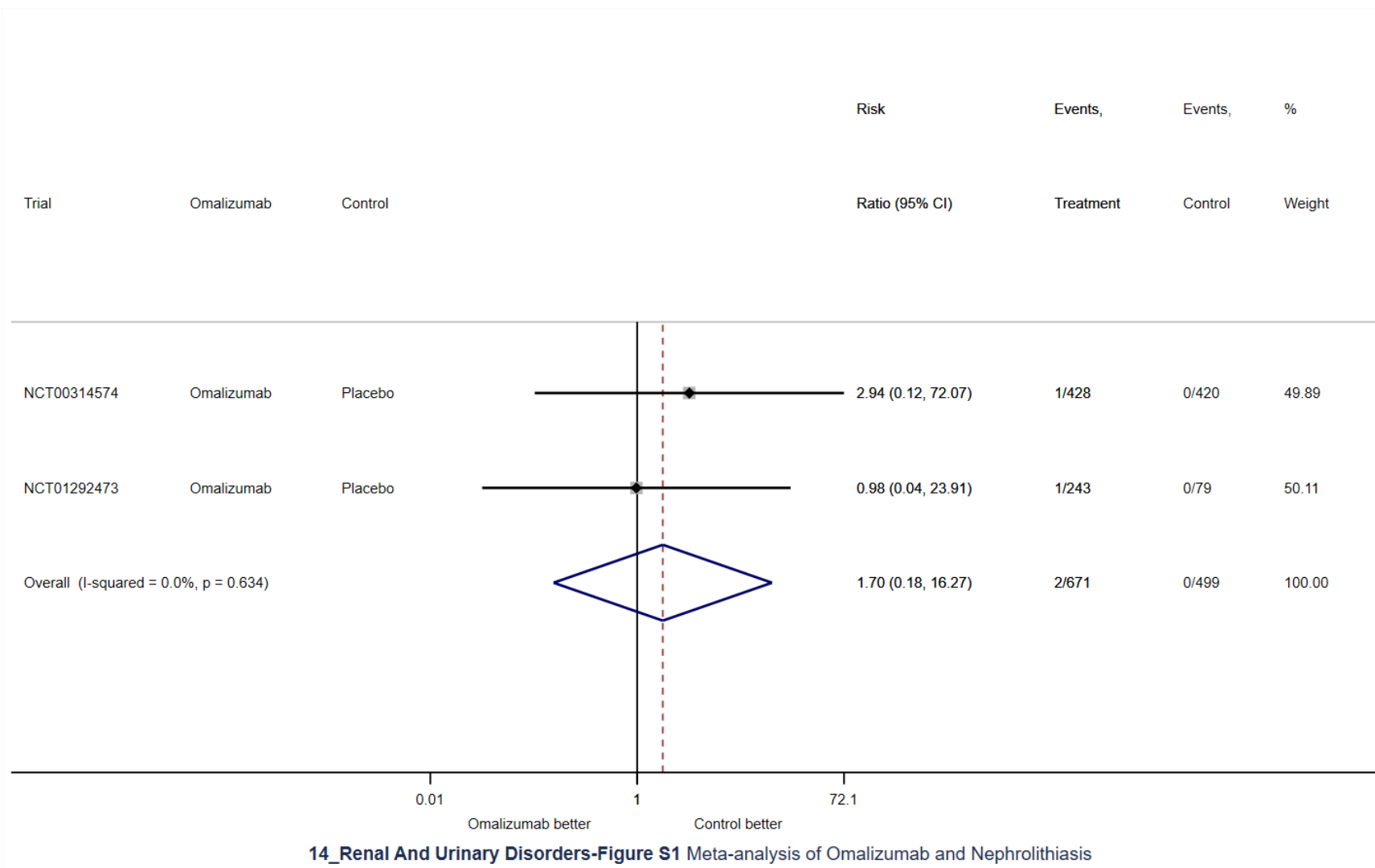

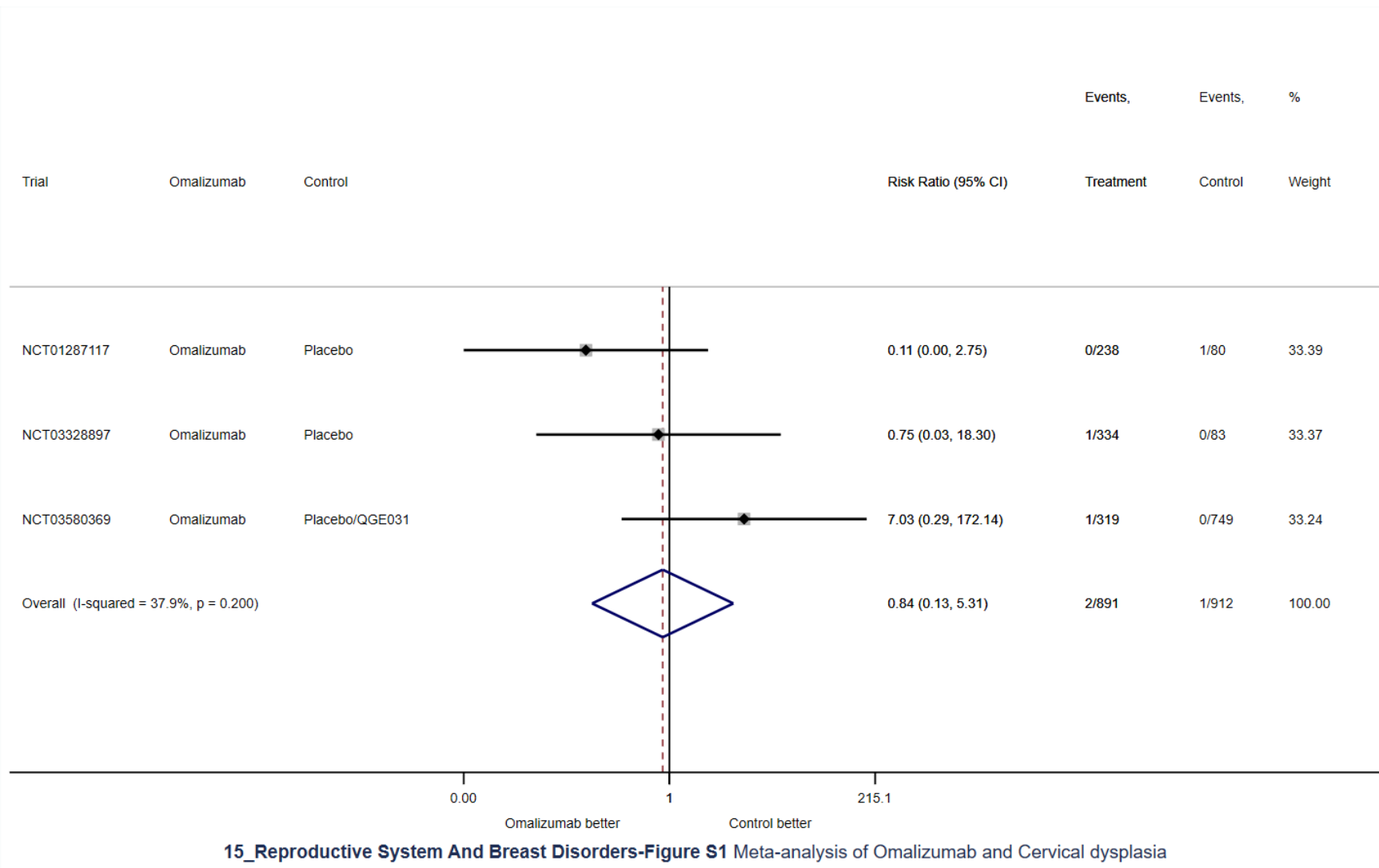

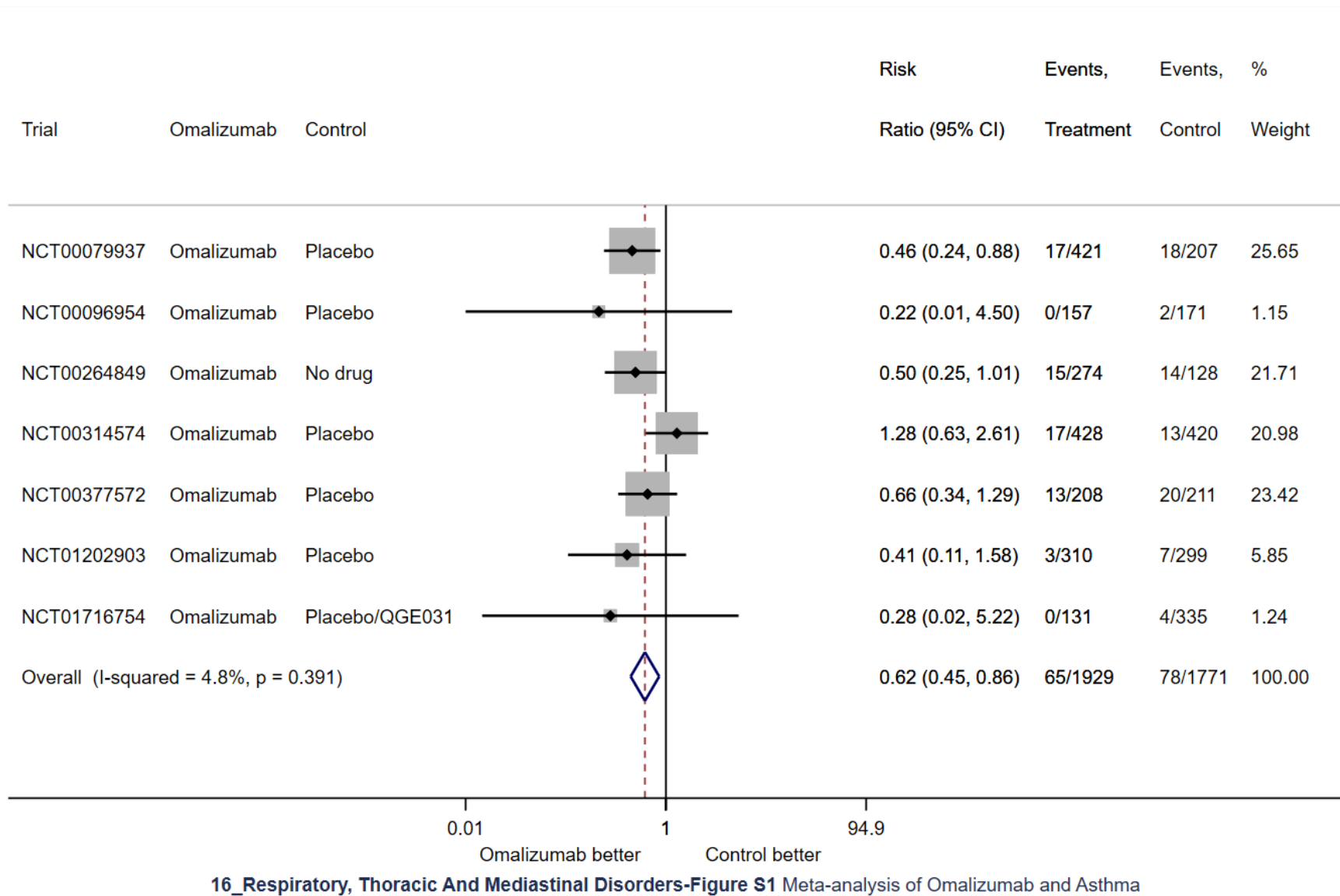

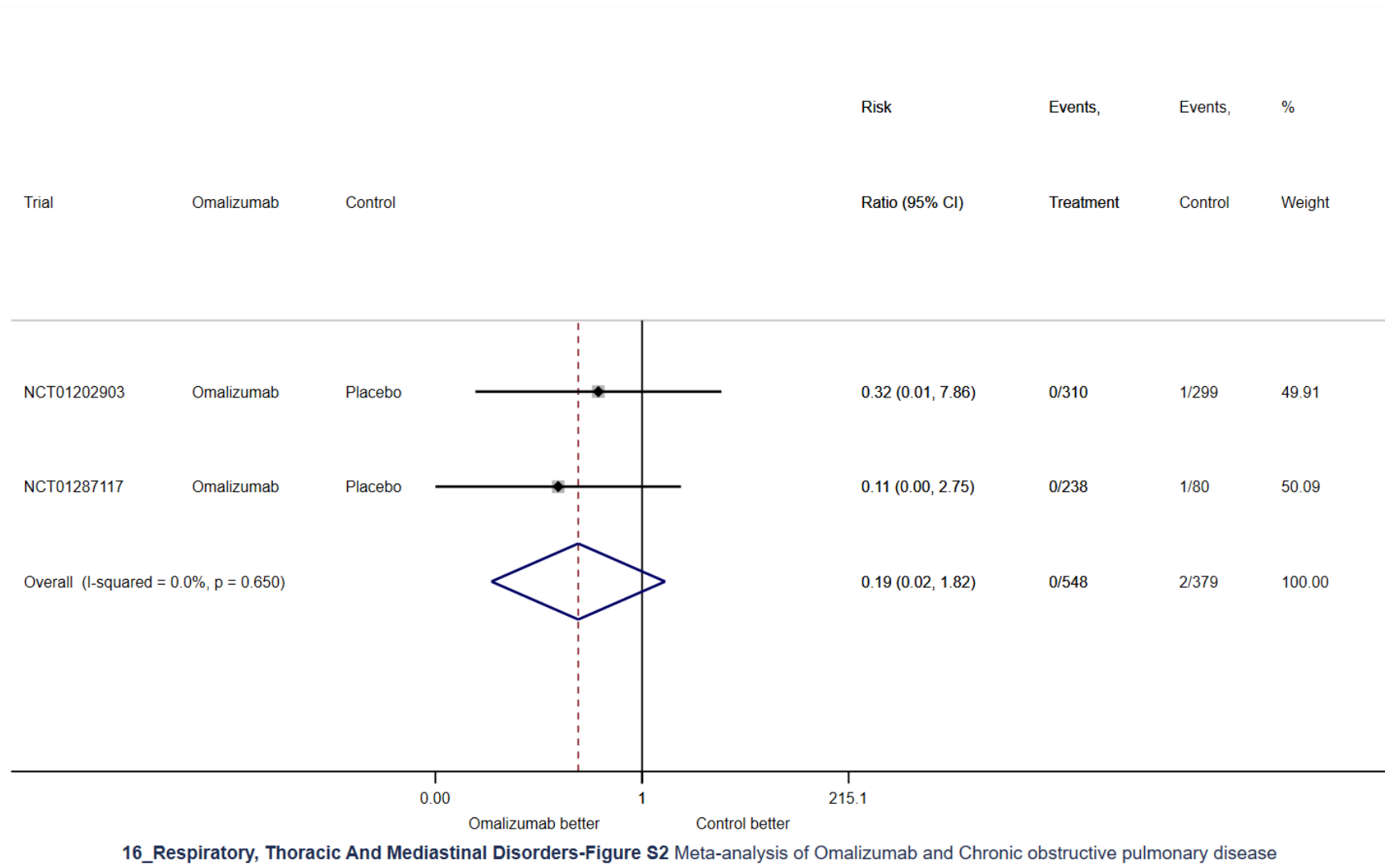

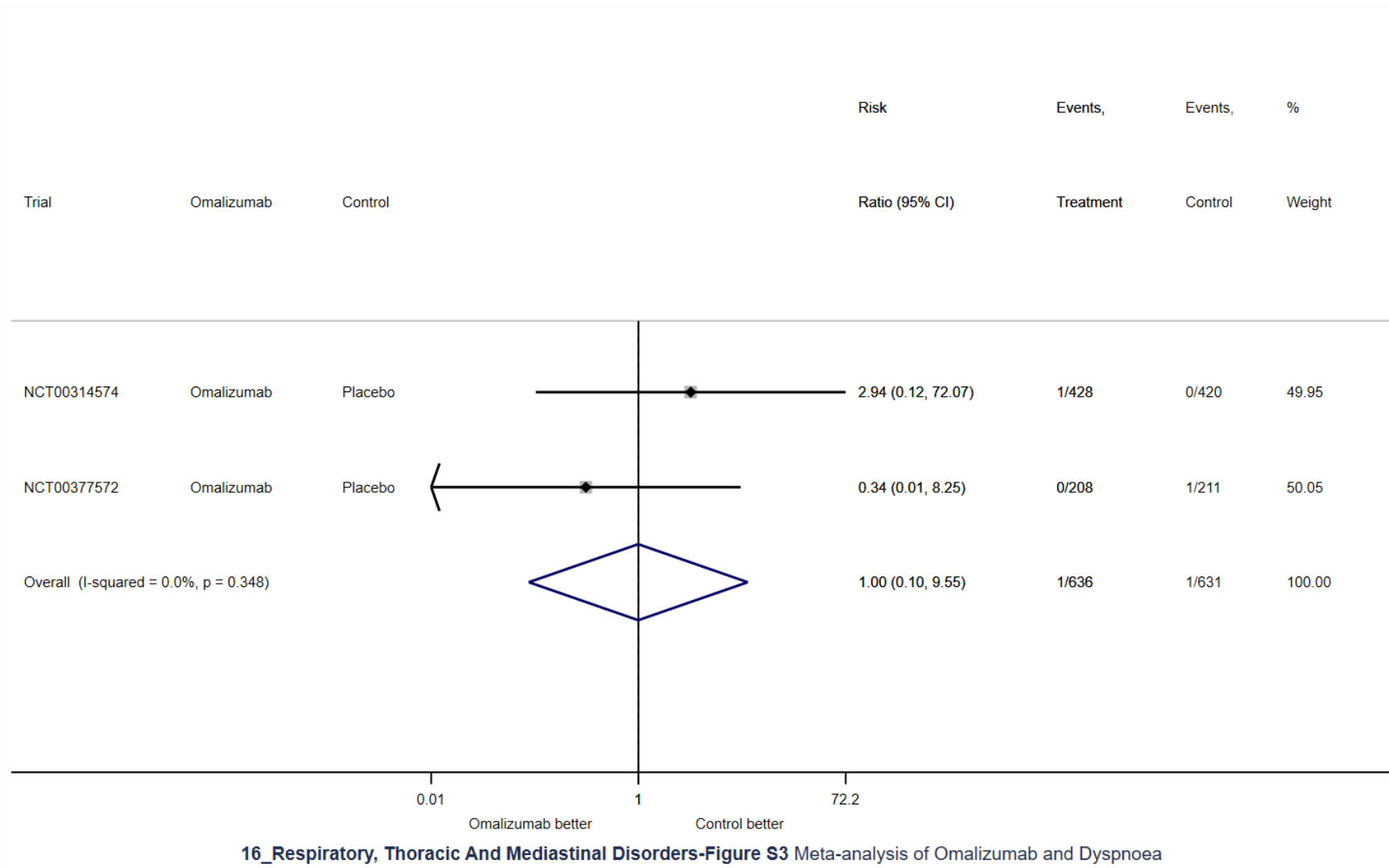

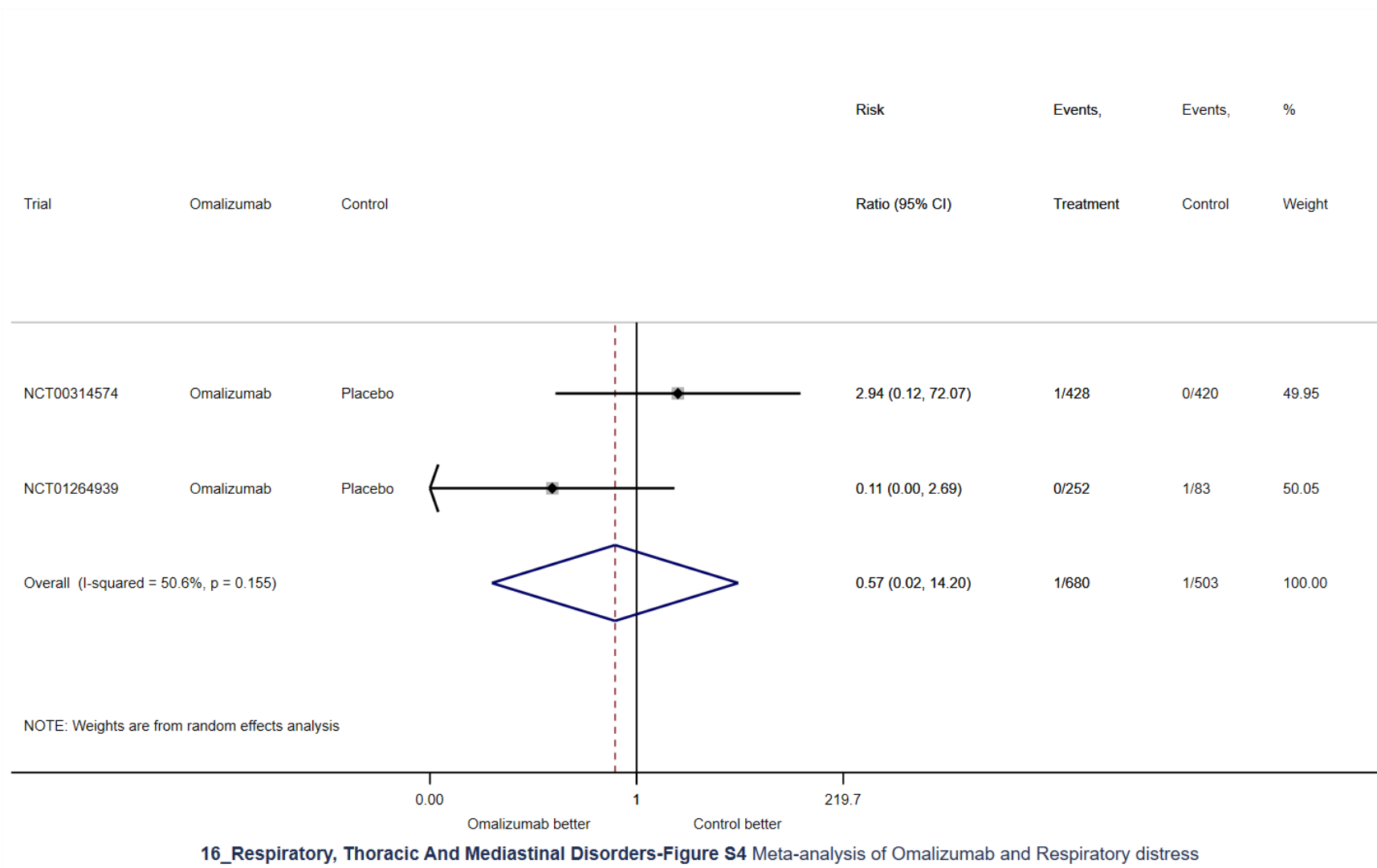

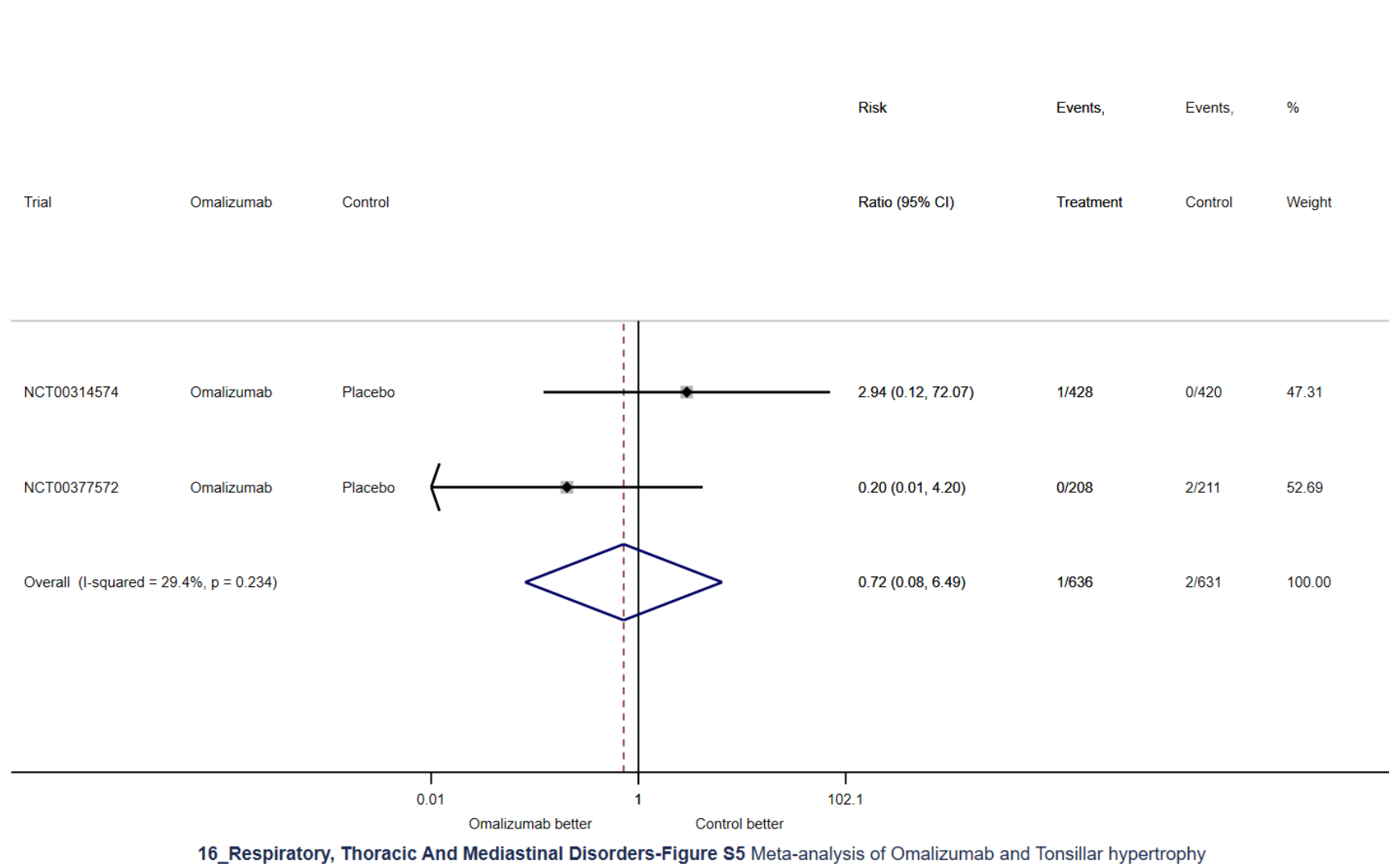

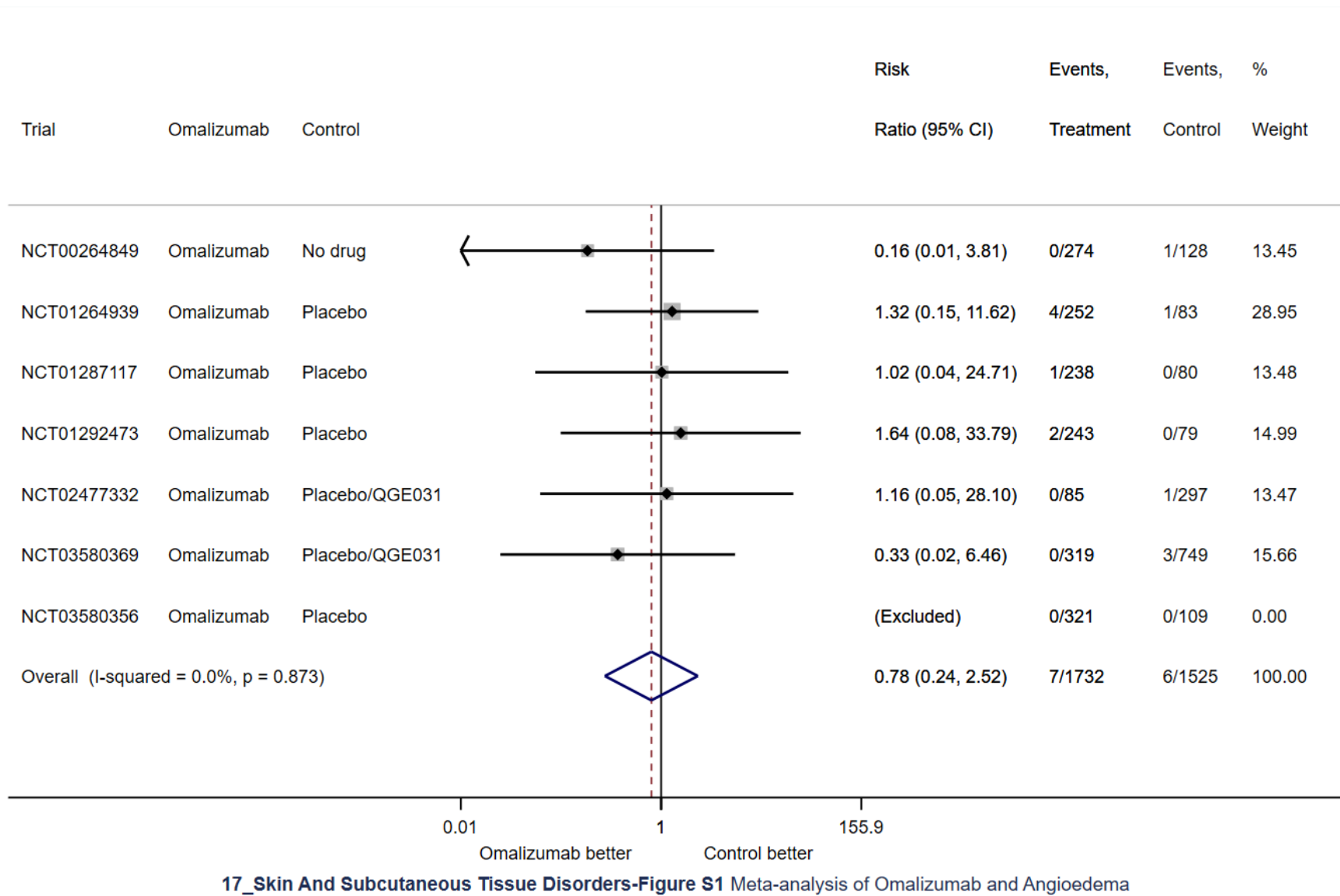

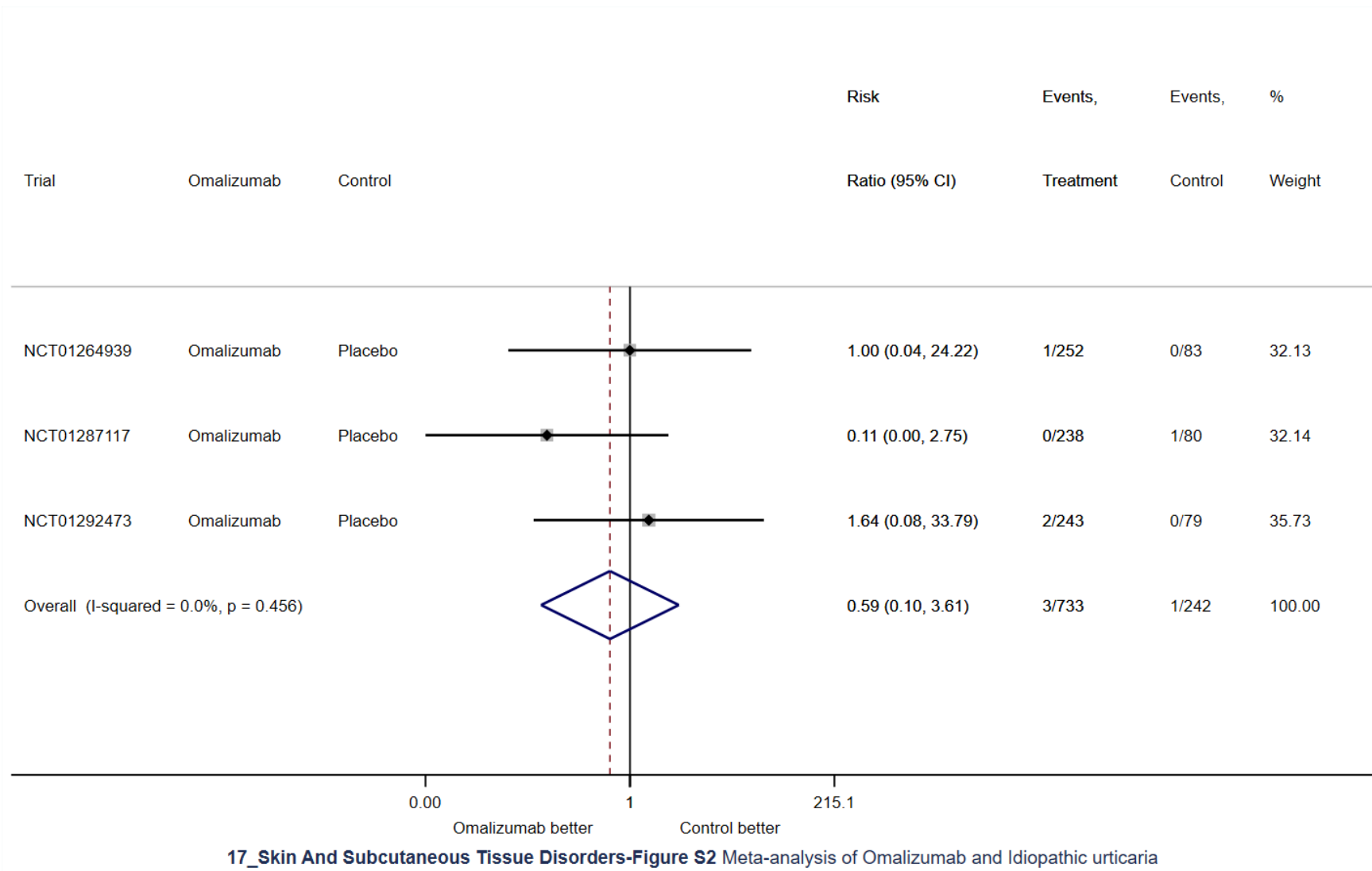

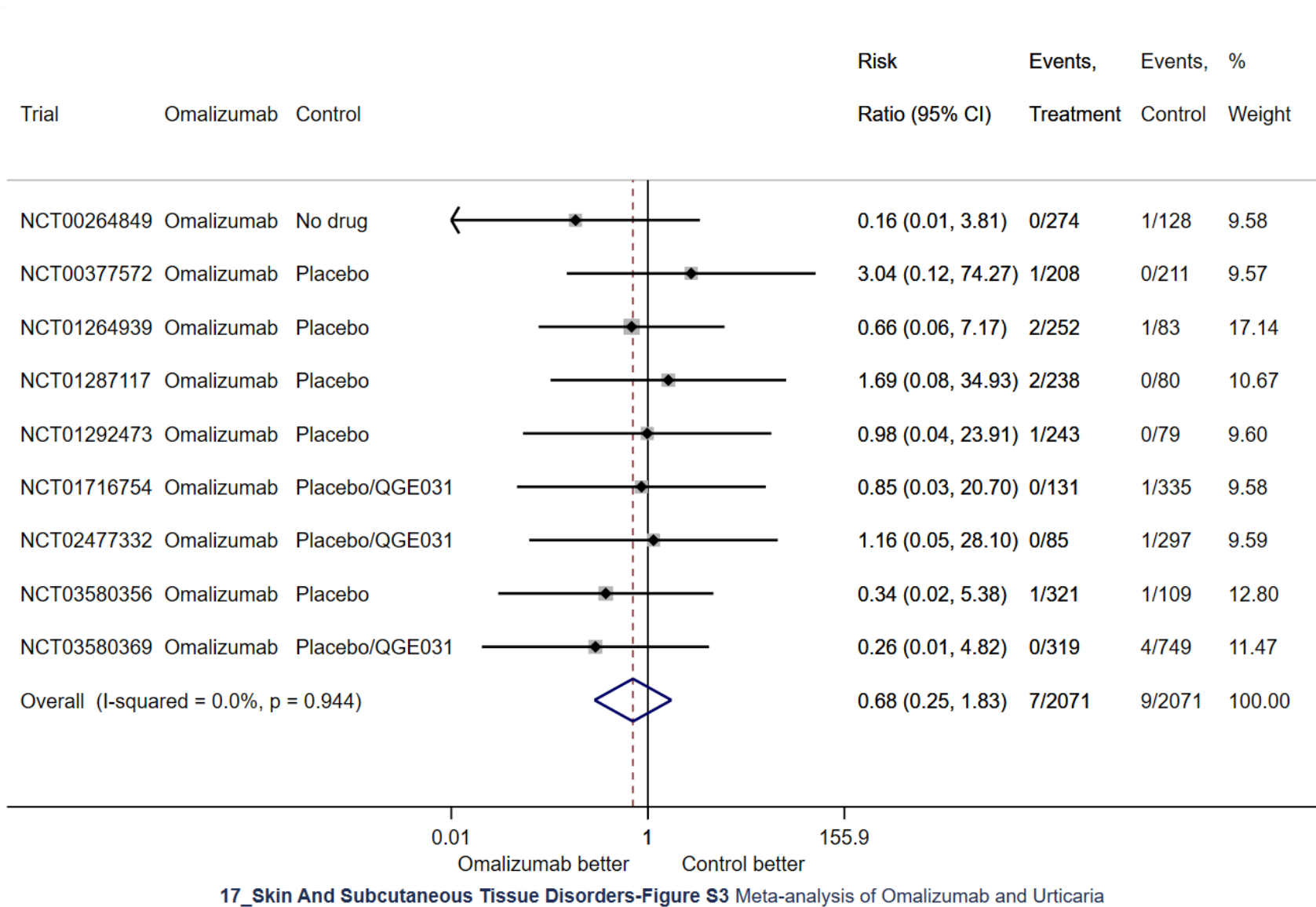

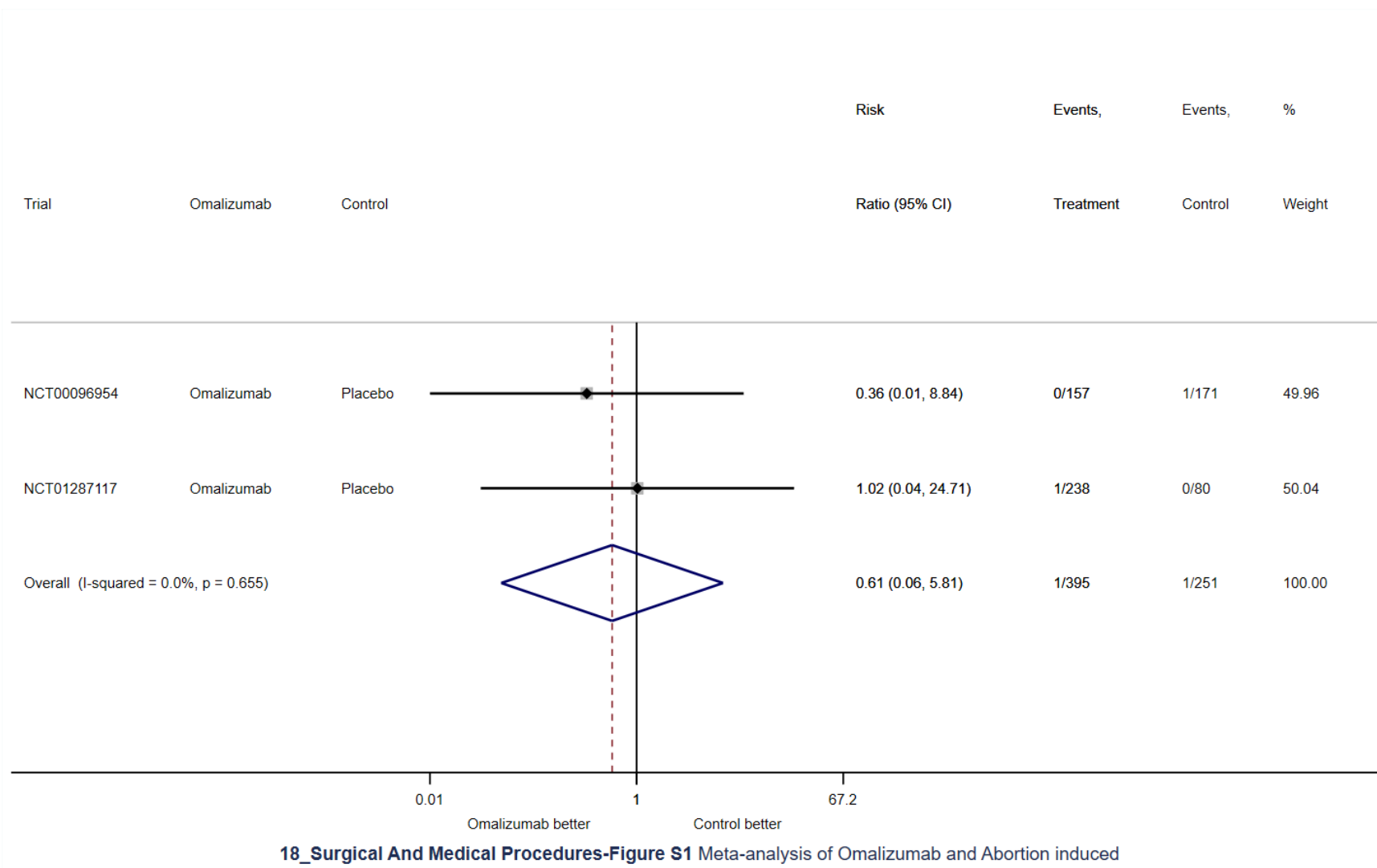

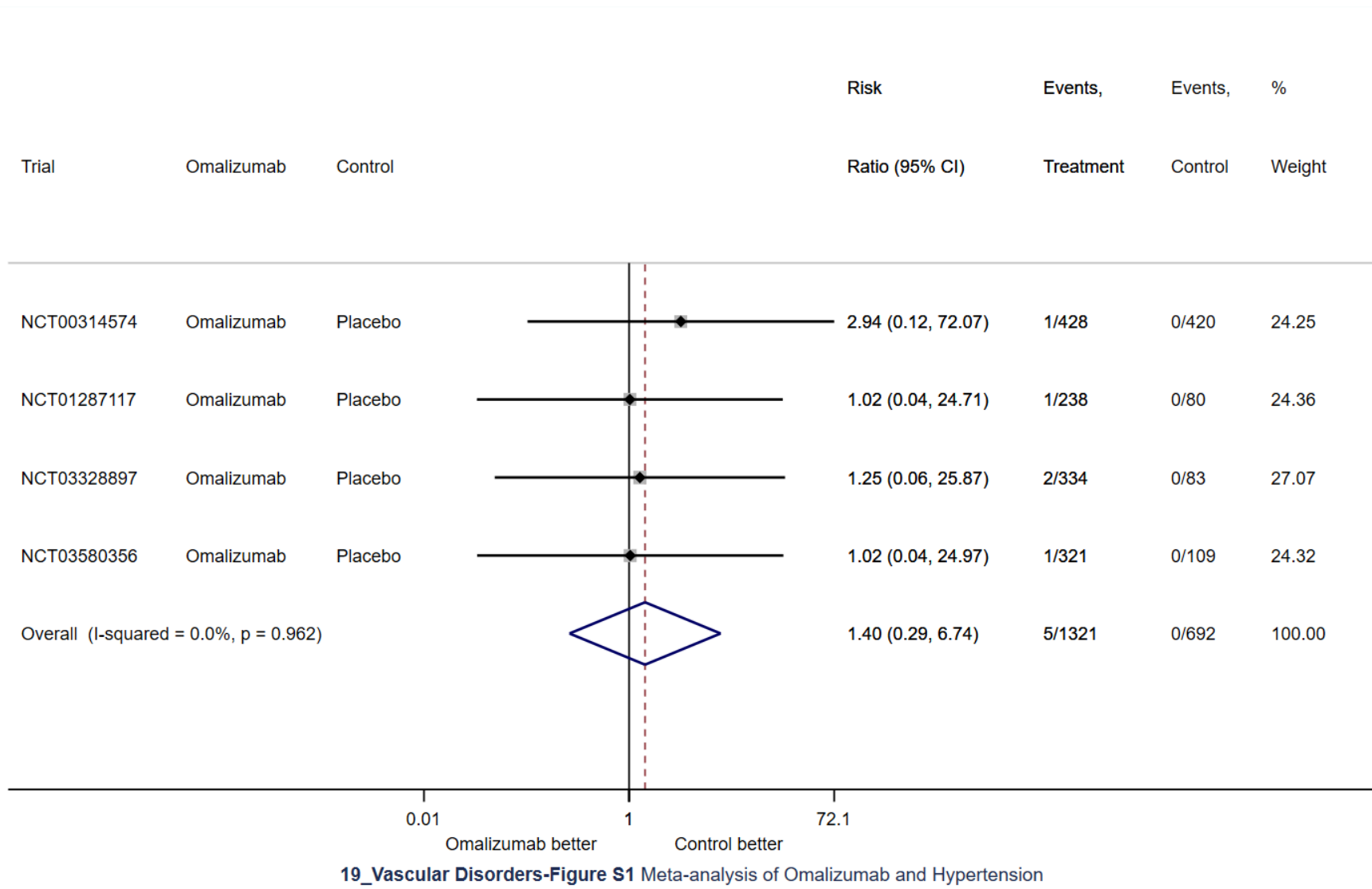

Supplement: Supplementary Figure 2 — Forest plots of omalizumab. [file Image_2.PDF]
